# Persistence drives gene clustering in bacterial genomes

**DOI:** 10.1186/1471-2164-9-4

**Published:** 2008-01-07

**Authors:** Gang Fang, Eduardo PC Rocha, Antoine Danchin

**Affiliations:** 1 Génétique des Génomes Bactériens, Institut Pasteur, 28 rue du Dr. Roux, 75724 Paris Cedex 15, France; 2 Atelier de Bioinformatique, Université Pierre et Marie Curie-Paris 6, 12, rue Cuvier, 75005 Paris, France

## Abstract

**Background:**

Gene clustering plays an important role in the organization of the bacterial chromosome and several mechanisms have been proposed to explain its extent. However, the controversies raised about the validity of each of these mechanisms remind us that the cause of this gene organization remains an open question. Models proposed to explain clustering did not take into account the function of the gene products nor the likely presence or absence of a given gene in a genome. However, genomes harbor two very different categories of genes: those genes present in a majority of organisms – persistent genes – and those present in very few organisms – rare genes.

**Results:**

We show that two classes of genes are significantly clustered in bacterial genomes: the highly persistent and the rare genes. The clustering of rare genes is readily explained by the selfish operon theory. Yet, genes persistently present in bacterial genomes are also clustered and we try to understand why. We propose a model accounting specifically for such clustering, and show that indispensability in a genome with frequent gene deletion and insertion leads to the transient clustering of these genes. The model describes how clusters are created via the gene flux that continuously introduces new genes while deleting others. We then test if known selective processes, such as co-transcription, physical interaction or functional neighborhood, account for the stabilization of these clusters.

**Conclusion:**

We show that the strong selective pressure acting on the function of persistent genes, in a permanent state of flux of genes in bacterial genomes, maintaining their size fairly constant, that drives persistent genes clustering. A further selective stabilization process might contribute to maintaining the clustering.

## Background

Made of DNA, a complex chemical substrate duplicated using a complex machinery, and submitted to all kinds of chemical aggressions and accidents, bacterial genome sequences are subject to many processes leading to sequence alteration, such as point mutations, rearrangements, gene duplications, gene deletions, lateral transfer of genes, etc. [1]. The availability of a rapidly increasing number of completely sequenced bacterial genomes makes it possible to explore gene order conservation in related and distant species. Gene order is preserved extensively in closely related species, but fades away in distantly related organisms [2,3]. Comparing different species, the conservation of gene order varies in parallel with the nature of the different selection pressures imposed upon genome stability [4]. Most studies of genome rearrangements have shown a marked preference for highlighting the fluidity of the bacterial chromosomes organization [5-8]. In contrast, the fact that conserved genes are not uniformly distributed but organized into clusters is a feature of the genome of *Escherichia coli *shared with many other bacteria [9]. This clustering property has long been used to predict gene function through the annotations of its neighborhoods, with the assumption that conservation of gene proximity is coupled with their functional relevance [9-11].

Hypotheses accounting for the clustering of genes in genomes basically break into three main categories. 1) Gene clusters are formed in situ as the consequence of gene duplication followed by divergence, and the conserved gene clusters are evolutionary relics allowing investigators to trace back their origins [12,13]. However, the constant rearrangement of chromosomes requires selection pressures to maintain the genes clustered along large evolutionary periods [4]. Furthermore, gene duplication happens much less frequently in prokaryotes than that in eukaryotes, while genes' clustering is much stronger in the former [14]. 2) Genes display a "selfish" behavior, aggregating into clusters to increase their chances of propagating through horizontal transfer into other genomes [15]. Briefly, this hypothesis is accounted for by a model describing the repeated loss and gain of batches of contiguous genes grouped together in a section of DNA. During this process, genes within batches coding for coupled functions will have a higher chance of increasing the organism fitness, and thus their own, than uncoupled genes, which would require pre-existence of the interacting partners in the chromosome. This provides a mechanism allowing gradual aggregation of functionally related genes among genes that are frequently laterally transferred. While the authors showed that this model works well for genes submitted to "weak selection pressures", they found that it did not hold for genes contributing to fitness at each generation, predicting that essential genes should not be organised into clusters in prokaryotic genomes [15]. This is in sharp contrast with the observation that, compared to non-essential genes, essential genes are significantly clustered in bacterial genomes [16-18]. 3) Finally, there is a large variety of works emphasizing some of the selective advantages that stabilize gene clusters in chromosomes, which interpret these advantages as the cause of clustering [10,11,19,20]. The nature of those selective advantages was generally discussed along two major lines: gene co-transcription and functional coupling. The role of co-transcription, which is at the core of the concept of operon [21,22], is supported by several lines of evidence [23-25]. The selection pressure for co-transcription is naturally gene co-expression. Conservation of bi-directionally transcribed gene pairs, which are not coded on the same mRNA molecule, was also associated with coupled functional properties [26]. Because many genes correspond to complex functions requiring the simultaneous presence of several components, the need for protein complexes cooperating in a given cellular function was therefore suggested as a selective driving force for gene clustering and formation and/or maintenance of operons [23,27]. A variety of parallel studies of the "uber-operon", a concept proposed to account for the clustering of several transcription units together, observed that in most cases genes are united by consistent functional themes [28,29].

However, some genes within uber-operons have no apparent functional relation with their neighbors. Their conservation has been attributed to "genomic hitch-hiking" suggesting that the genes' presence might simply reflect selection for stable expression at levels controlled by their neighbors [30]. Furthermore, rules leading to gene order conservation may be associated to chromosome organization and distribution in the cell, as shown by strong alteration of the bacterial growth observed upon some genome minimization attempts [31].

Conservation of gene proximity is useful to infer protein interactions or functional links [10], but some quantitative evaluations show that this is insufficient to explain the observed level of gene clustering. As a case in point, for *Mycoplasma genitalium*, gene clustering could only account for 37% of the functional interactions [32]. A program designed to predict gene function by building gapped local alignment of genome contexts between prokaryotic genomes, followed by studying the conserved gene strings provided significant predictions, yet it could not cover the majority of genes either [33]. These studies indicate that correlations between functional cooperativity and gene clustering could be lower than expected, depending on different datasets which reflect different evolutionary histories. Moreover, the existence of correlations indicates a relationship between two features as observed by analysis of the current bacterial genomes. If we aim at discovering the mechanism producing gene clustering, not its subsequent association with other events, the underlying causality is not as straightforward as it may seem: if functional coupling could stabilize clustering, clusters need to exist in the first place. Cluster formation could well be the initial process that allows selection and then stabilization of clusters displaying a strong contribution to fitness. In this sense, clustering could be a driving force for the creation of interactions. Even if co-transcription is the only major selective force that stabilizes favored gene clusters in bacterial genomes, the creation of essential gene clusters is yet to be addressed.

Considering the controversial explanations proposed to account for gene clustering, we tried to explore the concrete mechanisms that could result in clustering essential genes together, trying to avoid any type of teleological explanation. The systematic gene inactivation programs defined gene essentiality as whether a gene's inactivation leads to a dead end or not under laboratory growth conditions [34,35]. Remarkably, in our previous analysis of the conservation of experimentally identified essential genes in bacterial genomes we observed a further category of genes that persist in the course of evolution while they are not "laboratory essential" [16]. Many of the latter code for functions that considerably increase the fitness of the organism in natural environments, managing in particular the maintenance of essential functions. Thus, we proposed that gene persistence is the relevant representative of gene essentiality in an evolutionary perspective [16]. In this work, we restricted the analysis of gene clustering to persistent genes. We propose a model driving step-by-step the clustering of persistent genes, mainly based on two common evolutionary processes in bacterial genomes – lineage-specific genes loss and insertion. We discovered that to better survive from the random deletion process, persistent genes, which are under inherent high purifying selection pressure, organized as clusters. However, while clustering of persistent gene provided a significant opportunity to allow the genes to be inherited and spread, these clusters could also be destroyed by inevitable random gene insertion. We therefore explored a scenario where gene deletion and gene insertion would affect gene clustering. We subsequently measured the relative contribution of known co-transcription, protein-protein interaction and protein functional coupling to persistent gene clustering. We suggest that they operate as a stabilization force that maintains gene clustering after gene clusters have been formed in genomes.

## Results

### Persistent genes are organized into clusters and gene persistence is associated with their propensity to cluster together

**Figure 1:**
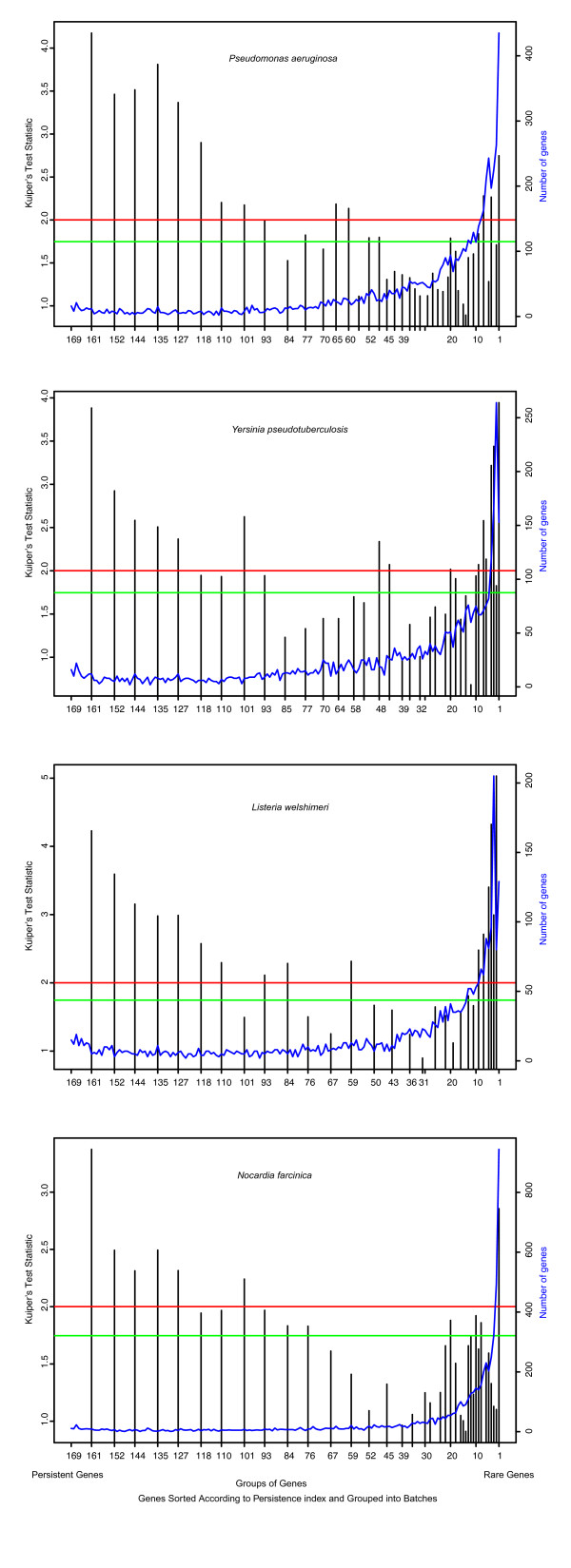
Gene clustering in specimen bacteria. The X-axis represents the persistence index, i.e. the number of orthologs found for a given gene among the 169 genomes. The blue curve shows the number of genes with identical persistence index, which is indicated by the Y-axis on the right. Genes with similar persistence are assigned into groups and tested for their distribution using Kuiper's test. The statistic for each group is shown as a vertical line. The two horizontal lines show the critical values given by Kuiper's test. The red one at y = 2.001 (resp. green one at y = 1.747) shows the critical value at the significant level of alpha = 0.01 (resp. alpha = 0.05). Both the persistent genes and the rare genes are significantly clustered along the chromosome, whereas the genes in between are not. Note that groups are not equally spaced along the X-axis to homogenize the number of genes in each group (from 100 to 200 genes). The gene persistence range for groups on the left side is 10%: e.g. the first group includes genes present in 90% to all of the bacteria; the 2nd group includes genes present in between 85% to 95% of the bacteria, and so on. Gene groups on the right side are assigned to groups with much narrower ranges, due to the fact that there is more rare genes than persistent genes in most bacteria.

A first indication that persistent genes cluster together can be found in the work of Martin et al, who observed that highly conserved genes in *E. coli *are organized into clusters [9]. This work, however, did not explore to what extent genes' conservation was coupled to clustering. Therefore, for each of the 169 bacterial genomes retained in the present study (see Additional file 1), we measured the deviation from uniformity in circular distributions (the Kuiper's test, see Method) to examine the distribution of genes in groups ordered following their frequency in genomes. We defined a Persistence Index (PI), as the percentage of bacteria containing a given gene. Figure 1 shows examples of the association between the genes' tendency of clustering and their PI (see Additional file 2 for the analysis of all bacteria).

**Figure 2:**
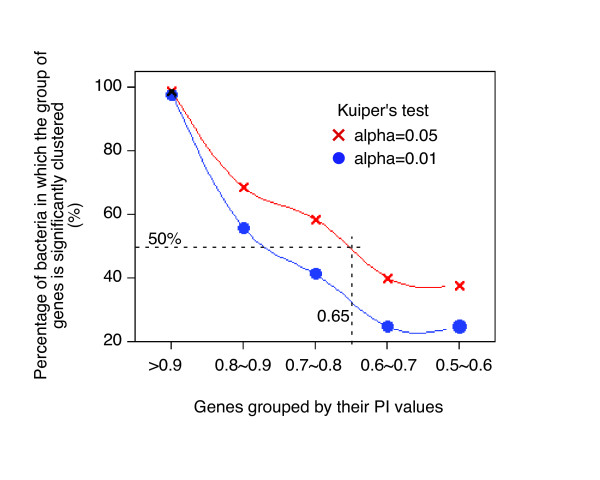
Distribution of groups of genes according to their persistence index. Genes were grouped according to their PI and examined each for its distribution in each of the 169 bacteria of interest. This figure shows the summary of the distributions of genes present in more than 50% (PI > 0.5) of the bacteria. The X-axis indicates gene groups: specifically, for each bacterium, genes with PI > 0.9 are assigned into the first group; genes with PI ≤ 0.9 and >0.8 are assigned into the second group, and so on. These genes groups were then examined by Kuiper's test in each bacterium for their distribution. The Y-axis shows the percentage of bacteria in which a group of genes was clustered. The red (resp. blue) curve is tested at alpha = 0.05 (resp. 0.01). Gene's persistence index is determined by the gene's presence in the 169 bacteria we studied.

As shown in Figure 1, genes are distributed into three categories: persistent genes – genes present in a majority of organisms, rare genes – those present in very few organisms, and genes in between. It is worth noticing that both persistent and rare genes form clusters, while the genes of the intermediary category do not cluster. The rarest genes display the strongest clustering tendency, and this is in agreement with the selfish gene hypothesis [15] and with the major processes of lateral gene transfer (conjugation, bacteriophage infection and transformation). In this work we will explore the mechanism leading to the clustering of persistent genes. This clustering shows three remarkable features (see also Additional file 2): i) The genes with PI >=65% (around 400 genes in each bacterium) are significantly clustered together in most bacteria (Figure 2 and Additional file 3). ii) The most persistent genes have the strongest tendency to cluster together, and as their persistence decreases, genes tend to become more uniformly distributed (Figure 2). Hence, there is a correlation between persistence and clustering. iii) A few bacteria do not follow the general trend, *viz. *Cyanobacteria, in that their persistent genes are fairly uniformly distributed in the genome (Additional file 2).

### Estimation of the length of batches of contiguous genes indels

**Figure 3:**
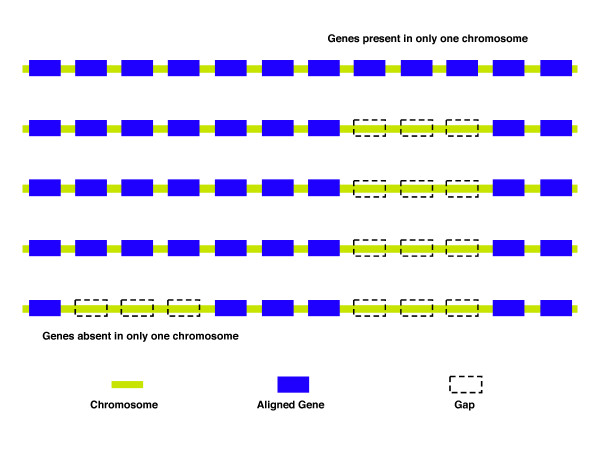
Deletion and insertion of batches of contiguous genes in closely related bacterial clades.

Previous studies suggested that genes are deleted from genomes in batches of contiguous genes [36-38]. To substantiate this observation, we made multiple alignments of gene contexts among 9 clades including 33 closely related genomes (see Methods). A batch of contiguous genes indel is a gap in the genomes alignment due to the presence or absence of a group of genes in only one strain (see Method, as illustrated in Figure 3). We found that the length of batches of contiguous genes indels ranges from 2 to more than 10 genes, with an average batch of approximately 3 genes (Additional file 4).

### A model featuring only passive selection groups persistent genes into clusters

**Figure 4:**
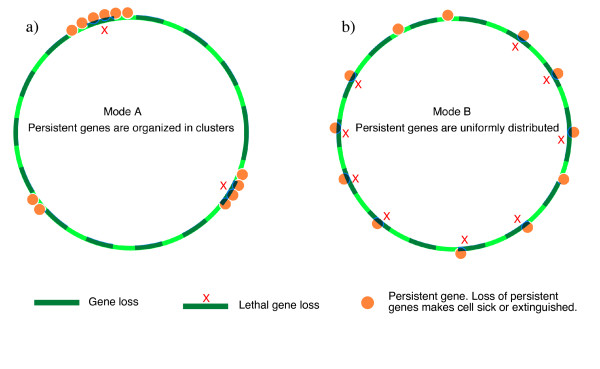
Two modes symbolizing persistent genes distributions along chromosomes. Under mode A, persistent genes are organized into clusters. Supposing that 15 gene deletions occurred, only 2 lead to the deletion of persistent genes. Under mode B persistent genes are uniformly distributed and 9 out of the same 15 gene deletions involve persistent genes.

As discussed previously, deletion of a persistent gene will much diminish bacterial fitness and prevent formation of a significant progeny [16]. Since deletions are very frequent in bacterial genomes we modeled the effect of deletion in batches of genes on the clustering of persistent genes. If genes are clustered (Figure 4.a), a small number of deletions will affect one or more persistent genes, while most deletions will affect none. The consequence of the first case is that the cell will have no progeny, whereas in the second it will not be much affected. In the second extreme gene distribution mode (Figure 4.b) persistent genes are uniformly distributed. As a consequence many deletions will include one, rarely more, persistent genes. All these will prevent the cell to have a significant progeny. Thus, under these very simplifying conditions, the clustering of persistent genes is adaptive because it renders the genome more robust to deletions.

To explore the validity of the model, we performed simulations using the following assumptions: 1) fixed population size; 2) fixed genome length; 3) fixed number of persistent genes; 4) genes are deleted and inserted in batches of contiguous genes, and this can happen randomly at any position. If the deletion involves a persistent gene, the cell has no progeny; 5) deletion of non-persistent genes has no fitness effect. In our simulations, we regarded the genome as made of a string of genes as the basic units, and ignored the structure of intergenic and coding regions. Deletion and insertion simply meant the removal and addition of genes along the string succession of genes. In real cases, intergenic regions are much shorter than coding regions in bacterial genomes, thus foreign genes would probably insert within a coding region. However, this event is basically identical to a process assuming first a gene deletion followed by an insertion at the same place.

**Figure 5:**
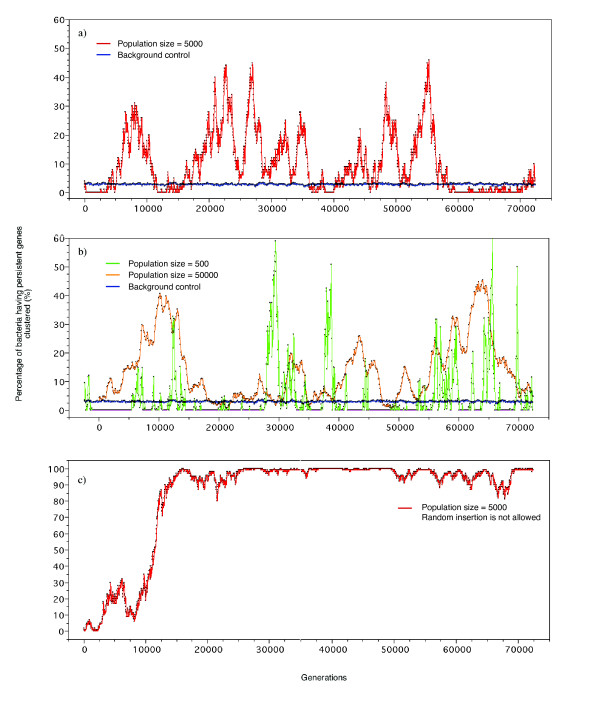
The percentage of genomes having persistent genes clustered over the time course of our simulation. The Y-axis shows the percentage of cells in which persistent genes are significantly not uniformly distributed (Kuiper's test, alpha = 0.05). a) Insertion is allowed at any position in a population of 5 000 cells. The red curve is under the hypothesis that deletion of persistent genes significantly affect the cell's multiplication, and the blue curve is the control supposing that persistent genes are indistinguishable from the other genes; b) Comparison of different population sizes. The green curve shows the simulation with a population size of 500 cells, and the orange curve is for a population of 50 000 cells. The blue curve is the control; c) Insertion is not allowed within clusters of persistent genes. In panel a and b, areas calculated by integral beneath the green, red and orange curves are 6290, 14033 and 19892, respectively for population sizes of 500, 5 000 and 50 000.

We ran the model on 5000 chromosomes, each with randomly distributed 3600 dispensable and 400 persistent genes (see Methods). We tested at each generation the clustering of persistent genes (Kuiper's test, alpha = 0.05). The percentage of bacteria with significant clustering of persistent genes fluctuates widely (red curve in Figure 5.a). Several peaks appear during the simulation and the analysis of the maximal peak showed long clusters of 5 to 7 persistent genes. As intuitively predicted, this simulation shows that the conflict between gene indispensability (at the basis of persistence) and deletion of batches of contiguous genes tends to group persistent genes into clusters. We repeated this simulation for 10 times, with similar results (Additional file 5.a). We also made simulations for smaller (500) and larger (50 000) population sizes (resp. green and orange curve in Figure 5.b). This showed that the clustering effect increases steadily with population size, as expected from a trait under weak selection. We further made a control of persistent gene's indispensability during the simulation: supposing that deletion of persistent gene(s) had no fitness effect (while inserting the deleted gene batch back into the chromosome at a randomly chosen place), persistent genes clustering constantly appeared in just 2 ~ 3% chromosomes, and did not vary over generations (blue curve in Figure 5.a).

For short population sizes the clustering is quickly disrupted by continuation of the insertion/deletion process (see Figure 5.b). In this scenario, clustering is therefore a dynamic process where groups of persistent genes form and vanish in the course of generations but in the absence of other selective forces requires high population sizes. These are not rare among bacteria, but the lack of knowledge of the insertion/deletion frequency in bacterial lineages precludes the development of a more quantitative model. That persistent genes tend to cluster together in many bacterial clades suggests that either population sizes are indeed sufficient to select for this trait or that there exists some sort of selective stabilization pressure superimposed on the process we have tried to mimick.

### Mutually Attracted Gene Pairs

**Figure 6:**
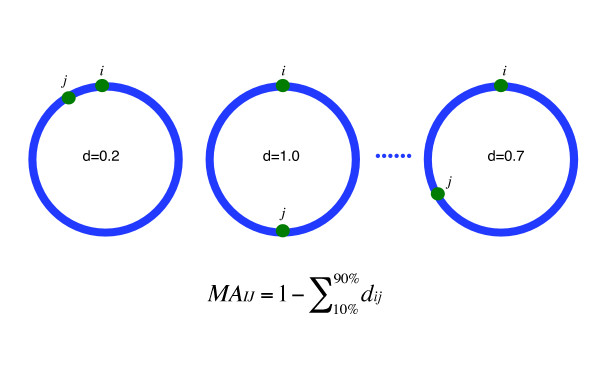
Mutual Attractivity (*MA*) between gene *I *and *J*.

**Figure 7:**
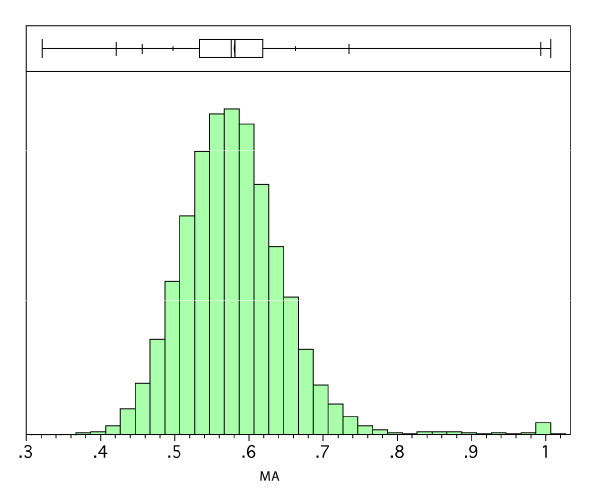
Histogram of the distribution of *MA *between pairs of persistent gene. The abscissa is the *MA *between every pair of genes, and it spans from 0.315 to the maximum of 1.0 (with our definition of *MA *the expected range is 0 to 1). The mean is 0.574.

To understand which factors might stabilize the clusters of persistent genes, we looked for genes staying conservatively close to each other in all the 169 bacterial genomes. To this aim, we introduced the concept of Mutual Attractivity (*MA*) between genes. The *MA *is a measure derived from the average distances between two genes in all the genomes, with shorter distances corresponding to stronger attraction (see Methods, Figure 6). We subsequently analyzed the 384 genes that are persistent among the 169 bacteria (see Methods), looking for pairs of genes that stay consistently clustered together (Figure 7).

If two persistent genes were randomly distributed, their *MA *should follow a normal distribution, with mean 0.5. By contrast, the distribution of *MA *among all pairs of persistent genes is significantly skewed toward the right, with mean 0.569 (Figure 7). This could be explained by the large-scale organization of the bacterial genome that tends to bias the distribution of some genes. For example, highly expressed genes, which often are persistent, cluster near the replication origin in fast growing bacteria [39]. This clustering is due to the location preference in the chromosome, not discriminant for a given pair's association, thus it leads to *MA *values that on average are not necessarily very large but still larger than the average given by a random process, i.e. 0.5. A further remarkable feature of the distribution is that we also find using the Expectation Maximization algorithm (see Methods) a small group of pairs of genes that are grouped at a value close to 1 (Figure 7), corresponding to pairs of genes that are consistently co-localized (see Methods). In summary, isolated from the mixture distribution of 73536 *MA *(see Methods), most gene pairs (over 98%) belong to a class following a normal distribution (average *MA *= 0.569, standard deviation = 0.063) while 1064 pairs form a smaller class of genes located close to each other (*MA *between 0.794 and 1). These 1064 gene pairs (formed by association of 258 genes into specific pairs) from that second class were named Mutually Attracted Gene Pairs (MAGP). Approximately half (506 pairs) of this group was composed of ribosomal proteins genes. The list detailing the 1064 MAGP is supplied in Additional file 6.

### Association between co-transcription and gene clustering

We used the 1064 MAGP to investigate the nature of the "stabilization" forces that might maintain the clusters' integrity. Since most bacterial genes are organized into operons, co-transcription might be regarded as the major force gluing together persistent genes. We firstly estimated the contribution from co-transcription to maintain MAGP. In the absence of a general set of experimental data on co-transcribed genes for all genomes in this study, we used three indicators to segment bacterial chromosomes and predict operons borders: presence of a rho-independent transcription terminator, intergenic regions spanning more than 200 bp or presence of two adjacent Coding DNA Sequences (CDS) on each of the complementary DNA strands (this prediction method fits well with experimentally identified operons, when data are available, see Methods). Each MAGP was examined for its distribution relative to operons in the 169 bacteria. Based on the proportion of bacteria in which the two genes belonged to the same operon, coupled with the distance between them in bacteria where they were not in the same operon (see Methods), we tested if a MAGP is maintained by operons. With this estimation, 563 (53%) MAGP were clustered as (part of) operons; removing ribosomal protein genes from the set (they display considerable persistent clustering), we concluded that operons maintained together 268 (48%) of the 558 MAGP (Additional file 6). Thus, co-transcription is one of the forces contributing to the stabilization of the clusters of persistent genes but is not enough to account for the entire phenomenon. It must also be stressed that this contribution correlates with clustering, but that we have no way at this point to know whether it causes clustering.

### Highly conserved protein interaction sets are weakly associated with gene co-localization

A modest proportion of interacting proteins genes in *E. coli *are co-localized in the chromosome [40]. We tested if the physical interaction between proteins could account for MAGP. We restricted our analysis to the set of 197 persistent genes for which all possible interactions were explored by Butland et al: they made up 1164 interactions. In our analysis, 742 MAGP are found for genes in this same set. However, only 127, i.e. 10.9%, of the 1164 interacting pairs are also MAGP, and conversely only 17.1% MAGP are interacting pairs in *E. coli*. A detailed Venn diagram showing the overlapping relationships between all the sets is in Additional file 7. The low overlap between the interacting pairs and MAGP strongly argues against a major stabilizing role for the known protein-protein interactions on the clustering of persistent genes.

When we removed the 44 ribosomal interacting pairs we were left with 83 gene pairs that are MAGP and code for physically interacting proteins, 22 of which were coded in the same operon. As a consequence, after the removal of the overlap (27%) with operon structure, the effect of protein physical interactions contributes to only 19% of MAGP. In summary, gene co-transcription and known protein physical interaction together could explain two thirds of the non-ribosomal MAGP.

### Functional coupling among genes in persistent gene clusters

Co-transcription and protein-protein interactions correspond to direct physical interactions. As other functional couplings may also have a role on MAGP stabilization, we assessed the influence of known functional association in the creation of MAGP. We first classified the genes forming MAGP into functional categories, based on our previous work [41], with some modifications (Additional file 8). We then examined each MAGP to see if the two genes were functionally coupled (see details in Additional file 6). This showed that 618 (58%) MAGP were composed of genes belonging to the same functional category, among which 391 were MAGP maintained by operons. In a similar way, after removing the 506 ribosomal MAGP, only 112 of the 558 non-ribosomal MAGP belonged to the same functional category. Among those, 96 (85%) were accounted for by operons, and another 6 MAGP by physical protein interaction pairs. In summary, functional coupling that would not be already taken into account either in the operon structure or in protein-protein interactions, could explain very few MAGP (~2%). When integrating co-transcription, protein physical interaction and gene functional coupling together, 69% of the non-ribosomal MAGP could be explained. We need to stress however that the concept of functional coupling here is restricted to function definitions as defined in extant ontologies, leaving the possibility of unsuspected novel functional interactions

### Simulation of selective stabilization in gene clustering

In the simulation presented above, we allowed non-persistent genes to freely insert into persistent gene clusters, ignoring selective advantages provided by persistent gene clusters during evolution. Clusters in our simulation are not stable in time: they form and then are disrupted, to form again later in a different configuration. As a control, we examined whether the insertion process might be the cause of gene clusters disruption. Briefly, we did not allow genes to insert into a position where the two nearest persistent genes were close enough (with at most two non-persistent genes in between). Once the insertion was restricted, a new position would be examined, until an unlimited insertion position was found. The other steps in the simulation were kept unchanged. Not unexpectedly, insertion indeed is the cause of the instability of persistent gene clusters in the simulations (compare Figure 5.c with Figure 5.ab). This simulation was repeated for 10 times with similar results, suggesting that the contribution of selective stabilization is indeed essential to account for the observed clustering patterns (Additional file 5.b). A variety of selective advantages of persistent gene clusters could account for the "prevention of insertion" (observed from progenies) into them. Selective stabilization is operating at most levels of integration of biological processes [42]. It is therefore natural to assume that once persistent genes clusters are created, there will be a variety of selective advantages, i.e. stabilization forces, that might concur to preserve such clusters. Naturally, more biological realistic models should now be developed, where insertion is not completely prevented and where non-persistent genes deletion could have a distribution of fitness effects. For the moment, we lack the appropriate quantitative data to quantify such a model.

## Discussion

The tendency of persistent genes to cluster correlates with several biological or biochemical processes, notably co-transcription and protein-protein interactions. While this has been noticed in previous works [11,23-30], no mechanism specifically leading to persistent gene clustering has yet been proposed. A previous analysis of the extent of clustering into operons of essential and rare genes shows that, in *E. coli*, essential genes cluster more frequently than rare genes, leading the authors to question the selfish operon model [17]. This highlights the importance of considering different categories of genes when studying chromosome organisation: unless having obvious reasons to do otherwise, specific mechanisms resulting in clustering could be proposed for each category of genes, taking into account their tendency to be distributed in a large number or only in a few organisms. The parallel variation between gene persistence and their clustering tendency suggests that the persistent character of a gene allows a good classification of genes in the context of the study of chromosome organization. In fact, it is a better character than simply using laboratory essentiality, because these genes share many characteristics with other non-essential but persistent genes.

In an endeavour to understand clustering we constructed a model where batches of contiguous genes could be inserted and deleted randomly into a bacterial genome, while keeping constant its overall length. This process has a differential outcome, whether genes are inserted or deleted at loci involving persistent genes, or genes generally dispensable for the cell multiplication. During the initial generations in our simulation, persistent genes, initially chosen to be uniformly distributed (this displays the lowest possible level of clustering) remained approximately uniformly distributed in the chromosomes. At this step, gene insertion essentially boosted the creation of gene clustering, and this is the reason why we could see a steady growth of the number of chromosomes with their persistent genes clustered at the beginning of simulations (Figure 5.abc). In parallel with the accumulation of clusters, the probability that they would be destroyed by gene insertion increased. This accounts for the decrease of clustering observed at a certain point, when it reached a peak (Figure 5.ab). A straightforward control illustrated that once the insertions were not allowed to break persistent gene clusters, the clustering quickly became stable (Figure 5.c). Figure 5.ab illustrates the mutual opposition between gene deletion (creation) and insertion (destruction) upon gene clustering. We proposed using this simulation that random gene deletion could drive persistent genes clustering together in bacterial genomes. Once persistent gene clusters were created, selective stabilization caused by specific processes would ensure that clusters created by the purely random processes of genome remodeling acquire significant perennity. It should however, be noted that the stabilizing processes we explored are not enough to explain the extent of clustering observed in all genomes.

Among the stabilization forces, the advantage of co-transcription is the most obvious one as it can easily be discovered during evolution by the existing transcription machinery when genes are in close proximity. Our analysis suggested that this process accounts for 48% of overlap between predicted operons and mutually attracted gene pairs (MAGP, removing the bias due to ribosomal proteins). Protein interaction and functional coupling could also lead to clustering, but we found little evidence of an important contribution of these processes in our analysis.

It must also be stressed that protein interaction data contains substantial noise and is incomplete, which may have resulted in the overlook of significant interactions [43]. The question to what extent protein interactions correlate with gene clustering remains therefore open. A limitation of the analysis of functional coupling between genes in persistent gene clusters is that functional classifications are generally of fairly coarse granularity, and, in any event, very inhomogeneous. For example, the gene *secY*, a subunit of the secretome, is assigned to a functional class of protein transport and secretion belonging to the super class of cell compartmentalization. However, in the persistent genes clusters, it formed MAGP together with many ribosomal subunits, belonging to the information transfer class. Experiments indeed proved that *secY *functions closely coupled with ribosome [44]. This illustrates how unknown relationships existing in the genes forming clusters could still reveal unexpected functional coupling. Gene functional coupling is a fairly vague concept, and in the absence of explicit data about various types of functional interactions we cannot therefore exclude that unexpected types of interactions, not identified in functional ontologies, will be discovered that account for most of the stabilization of gene clusters. In any event, even if comprehensive and exact physical/functional interactions were established, one would still have to explore whether the interaction is a cause or a consequence of stabilization of gene clustering.

The degree of clustering of persistent genes varies significantly among genomes (Additional file 1). The reasons for this may be multiple. Firstly, some genomes are more stable than others because they witness an intense gene flux, and one would expect less stable genomes to show lower degree of clustering. Indeed, cyanobacteria genomes, which are unstable [4], show the lowest clustering tendency. Secondly, if gene clustering opens a window of opportunity for genes to become associated, the degree of clustering may reflect the adaptive events occurring in the species history. In both scenarios, understanding the clustering of persistent genes in bacterial chromosomes will allow a better understanding of genome evolution.

## Conclusion

Gene clustering in bacterial genomes is observed in two different categories of genes, persistent genes and rare genes, and the mechanisms leading to their clustering are not identical. Attempts to explain the whole clustering based on a single model are prone to bring forward one-sided views missing important constraints. To account for the clustering of persistent genes, we showed that the strong selective pressure acting on the function of persistent genes, in a permanent state of flux of genes in bacterial genomes, maintaining their size fairly constant, is sufficient to drive genes clustering. A further selective stabilization process might contribute to maintaining the clustering. We emphasized the importance to distinguish causes from simple correlations when discussing the relationships between biological phenomena where the order of causality is not known. The mechanism we proposed, allowing first to create, and then to select for clustered genes, is more likely to reflect the true evolutionary processes, without asking for any external cause, such as driving forces caused by interactions between objects that had no reasons to interact previously.

## Methods

### Bacterial genomes

Bacterial genome sequences and annotation were taken from the EBI entry point of the International Nucleotide Sequence Database Collaboration [45] on Jan. 1st 2007. To avoid bias introduced by a limited genome size when exploring the clustering of persistent genes, we excluded from the study genomes with less than 2000 genes (105 genomes). 30 bacteria with multiple chromosomes were removed as well. Proper gene identification being fundamental for this study, we also put aside 21 bacterial genomes without proper 16S rDNA annotations. This resulted in a set of 227 bacteria from 169 species. To reduce the bias of some species with many sequenced strains, we only used one strain from each species (see Additional file 1).

### Assignation of orthology and definition of persistence

Orthology between genes was identified by Bi-directional Best Hits with >=40% similarity in amino acid sequences and <=20% length difference in their protein sequences. The persistence index was calculated as the ratio of the number of orthologs relative to the total number of bacteria scanned [16]. When examining gene persistence, we used only one strain from each species (see Additional file 1).

### Groups of homologs and mutually attracted genes

From each bacterium, we selected the genes with PI >=65% and grouped all the putative orthologs together using the COG method [46]. We took into account the possibility of gene duplication (we defined duplications as genes from the same bacterium with amino acid sequence similarity >=80% and protein length difference <=20%, and in grouping homologs, a pair of duplicated genes was treated as same as a pair of orthologs). This procedure led to the identification of 580 groups of homologs. Considering gene duplications led to the same groups, as expected since persistent genes rarely duplicate [14]. Some groups comprised genes present in only one or two bacteria, while others had members from all of the 169 bacteria. We picked up the groups of homologs existing in a quorum of the bacteria investigated (>=110, i.e. more than 65%), and this procedure led to a set of 384 groups, each represented by a persistent gene common in the quorum.

The distance between two genes in one chromosome was denoted by dij=NijN/2−1
 MathType@MTEF@5@5@+=feaagaart1ev2aaatCvAUfKttLearuWrP9MDH5MBPbIqV92AaeXatLxBI9gBaebbnrfifHhDYfgasaacPC6xNi=xH8viVGI8Gi=hEeeu0xXdbba9frFj0xb9qqpG0dXdb9aspeI8k8fiI+fsY=rqGqVepae9pg0db9vqaiVgFr0xfr=xfr=xc9adbaqaaeGacaGaaiaabeqaaeqabiWaaaGcbaGaemizaq2aaSbaaSqaaiabdMgaPjabdQgaQbqabaGccqGH9aqpjuaGdaWcaaqaaiabd6eaonaaBaaabaGaemyAaKMaemOAaOgabeaaaeaacqWGobGtcqGGVaWlcqaIYaGmcqGHsislcqaIXaqmaaaaaa@3A8E@, where *N*_*ij *_is the number of intercalated genes between gene *i *and gene *j*, and *N *is the total number of genes of that chromosome. In different bacteria, this distance varied widely. A pair of genes retaining low *d*_*ij *_values in most bacteria signifies that these two are systematically located together. We need to find a measure to explore whether there are such pair-wise associated genes that are constantly close to each other in most bacteria. Since the bacterial genomes available are not equidistantly distributed in the phylogenetic tree, to tone down the bias due to phylogeny, we put aside the smallest and largest 10% *d*_*ij *_and calculated a mean of the remaining *d*_*ij *_acquired from all chromosomes to represent their average distance. In the cases where there are gene duplications, the distance of all combinations of the two genes were considered. When the average distance between a pair of genes was consistently small, it behaved as if the two genes had a mutual attraction. As Figure 6 illustrates, we proposed to use the value of 1 minus this average distance to measure the strength of such attractions. We named this intuitive measure *MA*_*IJ *_(Mutual Attractivity between gene *I *and *J*).

### Kuiper's test

The Kuiper's test assesses whether a distribution is uniform or not. It is adapted from the Kolmogorov-Smirnov test (K-S test). In K-S test, the hypothesis is made that the objects are uniformly distributed among a group of sequential units. The K-S test involves computing a variable called *D*-max which is the largest difference between the observed and expected cumulative frequencies measured for each unit. When the *D*-max is large enough, we can reject the hypothesis that the observed distribution is uniform [47]. This test is not appropriate to examine clusters at the two ends of a linear string, nor suitable to detect clusters distributed in a circle. Kuiper's test is meant to overcome these difficulties, using the sum of *D*^+^-max and *D*^-^-max, referring to the observation's largest deviation above and below the expected cumulative frequencies, respectively, as the statistics [48].

### Operons

We constructed putative operons in the same way as we did previously [16], taking into account the presence of rho-independent transcription terminators [49], the CDS direction and restricting the intergenic distance to less than 200 bp [50]. In the case of *E. coli*, for which the transcripts' data set is the most complete [51], 96% intergenic regions inside their operons are shorter than 200 bp. While this method is not perfect, it consistently produces groups of genes (over 95%) that are experimentally found to be co-transcribed [51].

### Mutually Attracted Gene Pairs and operons

A Mutually Attracted Gene Pair (MAGP, see Results) could be composed of two genes in some highly conserved operon, or be a pair of genes conservatively close together irrespective of operon structures. To find out those MAGP not maintained by operon structures, we examined the genes from each MAGP in all the bacterial chromosomes. If in more than 50% of the bacteria, the two genes from a MAGP were coded in the same operon, we regarded this MAGP as maintained by the operon. If this was not the case, we further measured the number of genes between these two genes in chromosomes where they were not part of the same operon, and calculated a new *MA *for these two genes. When putting aside the operon effect, if this new *MA *showed that the two genes still had a very strong attraction (3 sigma larger than the mean of *MA *drawn from pairs of genes randomly distributed; the mean and standard deviation were from the large class following a normal distribution, retrieved from Figure 7, see Results), we then concluded that this MAGP was not due to operons.

### Multiple alignments of gene contexts in bacterial clades and batches of contiguous genes deletion and insertion

We constructed closely related bacterial clades by picking up those species with more than 3 strains sequenced, putting aside the strains which were almost identical (16S rDNA are 100% the same, or more than 95% of genes are identical), for which we just retained one instance in each clade. For the clades counting many sequenced strains, like *E. coli *and *Staphylococcus aureus*, we limited our analysis to a maximum of 5 genomes. The resulting closely related bacteria clades were used to compare the genome contexts to detect batches of contiguous genes deletion and insertion. To define batches of contiguous genes indels we used the intuitive approach illustrated in figure 3. Where there was a gap (with the minimal length of 2 genes) in only one chromosome in the multiple alignments (Figure 3), we defined it as an indel. An in-depth identification of batches of contiguous genes indels to tell an insertion from a deletion would require a case-by-case analysis, since evolutionary time measured by some other conserved genes might not fit with such genes' influx/efflux in/from the chromosome. This is beyond the focus of this work. We used "batches of contiguous genes indel" as the generic term representing both events (insertion or deletion).

### Algorithm for indel-mediated evolution of the bacterial chromosome

At the initial state, a set of 5000 artificial circular chromosomes each containing 4000 genes was constructed, among which 400 uniformly distributed genes were picked up and labeled as persistent genes. We simulated the gene distribution evolution process with the following steps: at each generation, one random batch of contiguous genes deletion was performed in each chromosome. We assumed that the gene deletion and insertion of batches of contiguous genes length was 3 genes (we tried lengths of 4, 5 and 7 genes as well and this did not change the conclusion of the simulation; data not shown). If this deleted a persistent gene, the corresponding chromosome was not passed into the next generation; we then randomly picked up a position in each surviving progeny and inserted there a batch of contiguous non-persistent genes. The second generation was composed of the surviving progenies. To keep the cell population constant as 5000 bacteria (the model assumes a steady state) through generations, the inadequate amount in second generation was restored by picking up genes randomly from the surviving bacteria. We repeated this evolution process, generation after generation.

### Expectation Maximization and software used

The Expectation-Maximization algorithm to isolate components from the *MA *distribution was carried out by the EM program from Mclust R package [52]. At the initial step, we used *MA *= 0.8 as the boundary value to separate the *MA *into two distributions. An iteration process between expectation and maximization was then carried out through the EM program. For the expectation step, each *MA *was assigned a weight (possibility to belong to these two distributions), and then based on these weights, a maximum likelihood calculation updated the parameters of the two distributions. The process was repeated until parameters converged. Thus we obtained the clear boundary to separate the two distributions.

## List of abbreviations

PI, Persistence Index

MA, Mutual Attractivity

MAGP, Mutually Attracted Gene Pairs

## Authors' contributions

All authors contributed to the writing of the manuscript. GF performed the study and introduced the concept of mutual attraction, ER validated the statistical approaches and placed the study in the perspective of evolution of interactions, and AD proposed the study and the idea of purely passive evolution to gene clustering followed by selective stabilization.

## Supplementary Material

Additional file 1 Bacterial genomes used in this study, persistent genes and operons' distributions in bacterial chromosomes. Click here for file

Additional file 2 Gene clustering in bacterial chromosomes Click here for file

Additional file 3 Distribution of groups of genes according to their persistence index Click here for file

Additional file 4 Length of batches of contiguous genes indels Click here for file

Additional file 5 a: Simulation without considering stabilization forces. b: Simulation with stabilization forces Click here for file

Additional file 6 MAGP and its composition Click here for file

Additional file 7 Venn diagram showing the intersections between the datasets of protein interactions and MAGP Click here for file

Additional file 8 Functional annotation of the genes involved in MAGP Click here for file
