# Supplementary material for: Persistence drives gene clustering in bacterial genomes
Source: BMC Genomics. 2008 Jan 7;9:4. doi: 10.1186/1471-2164-9-4 (PMC2234087; doi:10.1186/1471-2164-9-4)
Supplement: Additional file 2 — Gene clustering in bacterial chromosomes [file 1471-2164-9-4-S2.pdf]

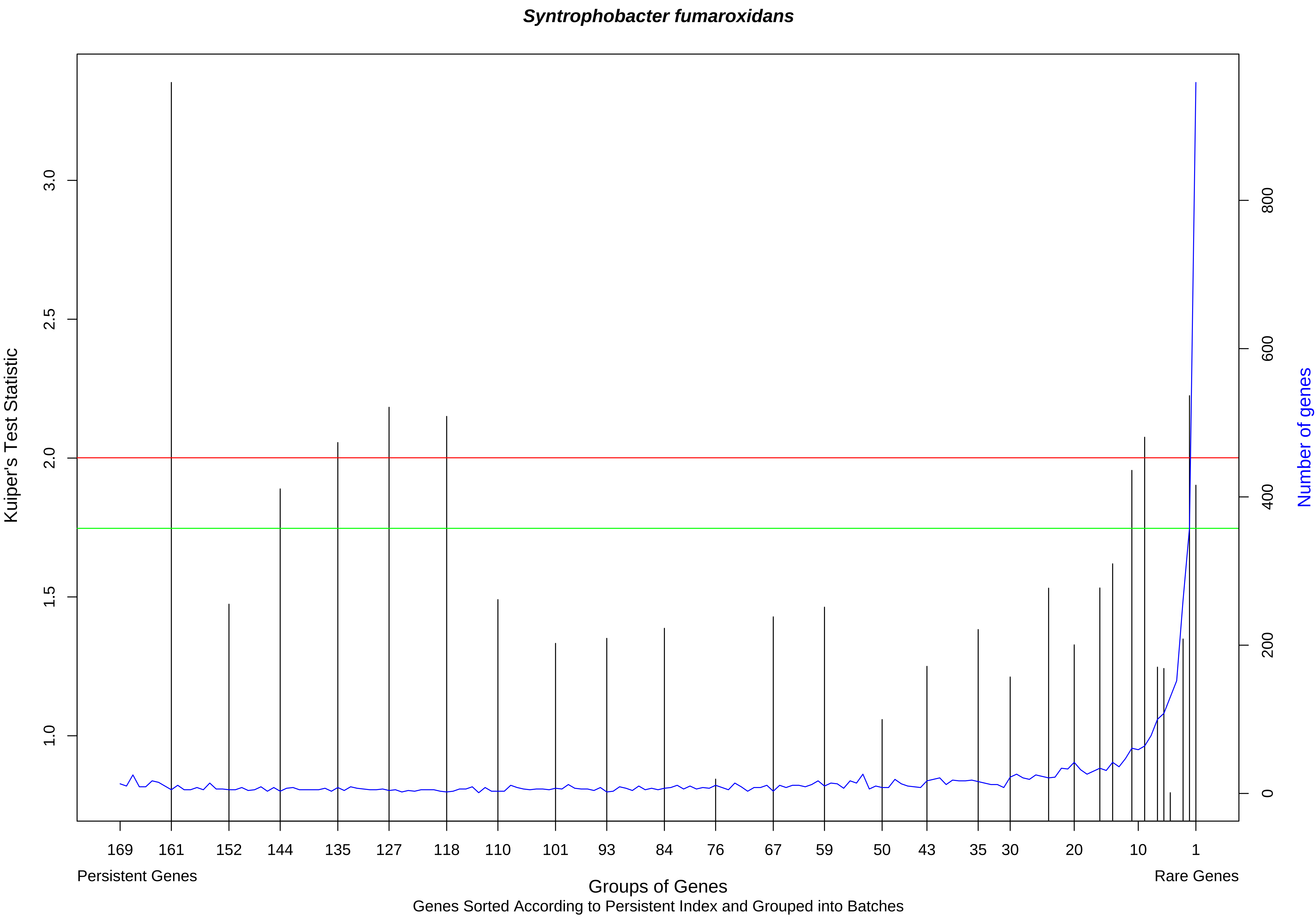

Syntrophobacter fumaroxidans
Kuiper's Test Statistic
Number of genes
1.0
1.5
2.0
2.5
3.0
0
200
400
600
800
169
161
152
144
135
127
118
110
101
93
84
76
67
59
50
43
35
30
20
10
1
Persistent Genes
Rare Genes
Groups of Genes
Genes Sorted According to Persistent Index and Grouped into Batches

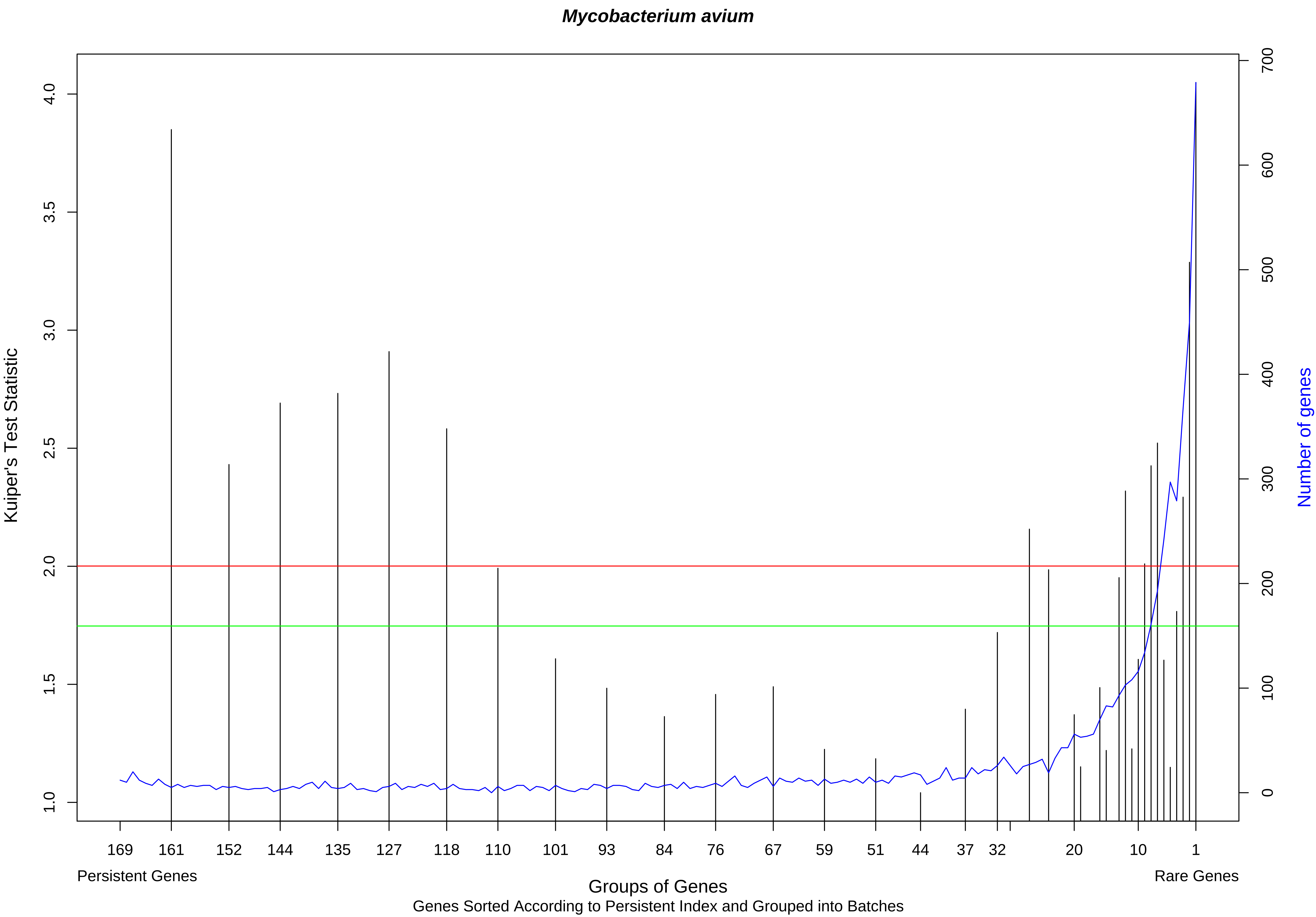
Mycobacterium avium
Kuiper's Test Statistic
Number of genes
1.0
1.5
2.0
2.5
3.0
3.5
4.0
0
100
200
300
400
500
600
700
169
161
152
144
135
127
118
110
101
93
84
76
67
59
51
44
37
32
20
10
1
Persistent Genes
Rare Genes
Groups of Genes
Genes Sorted According to Persistent Index and Grouped into Batches

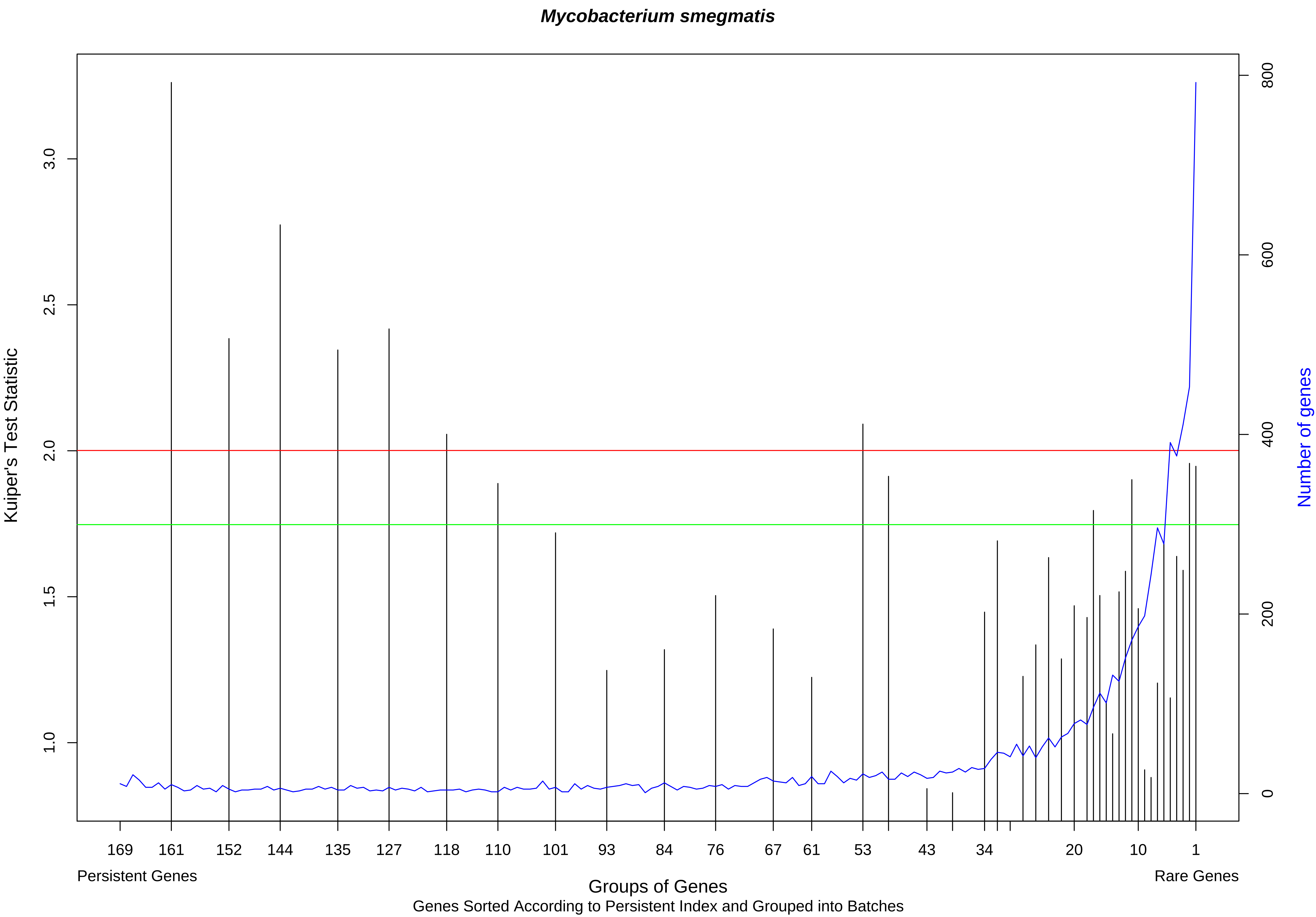

Mycobacterium smegmatis
Kuiper's Test Statistic
Number of genes
1.0
1.5
2.0
2.5
3.0
0
200
400
600
800
169
161
152
144
135
127
118
110
101
93
84
76
67
61
53
43
34
20
10
1
Persistent Genes
Rare Genes
Groups of Genes
Genes Sorted According to Persistent Index and Grouped into Batches

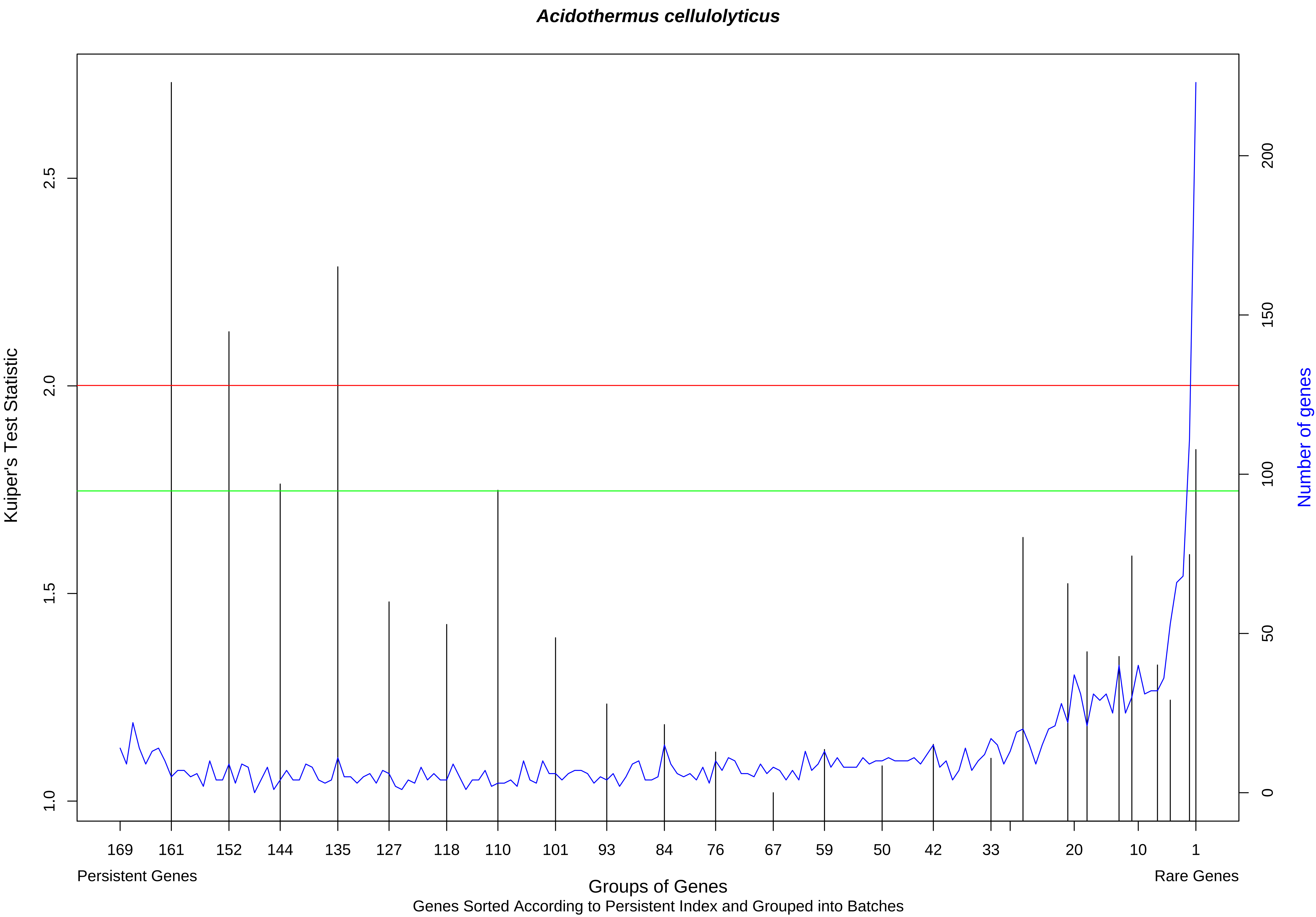
Acidothermus cellulolyticus
Kuiper's Test Statistic
1.0
1.5
2.0
2.5
Number of genes
0
50
100
150
200
169
161
152
144
135
127
118
110
101
93
84
76
67
59
50
42
33
20
10
1
Persistent Genes
Rare Genes
Groups of Genes
Genes Sorted According to Persistent Index and Grouped into Batches

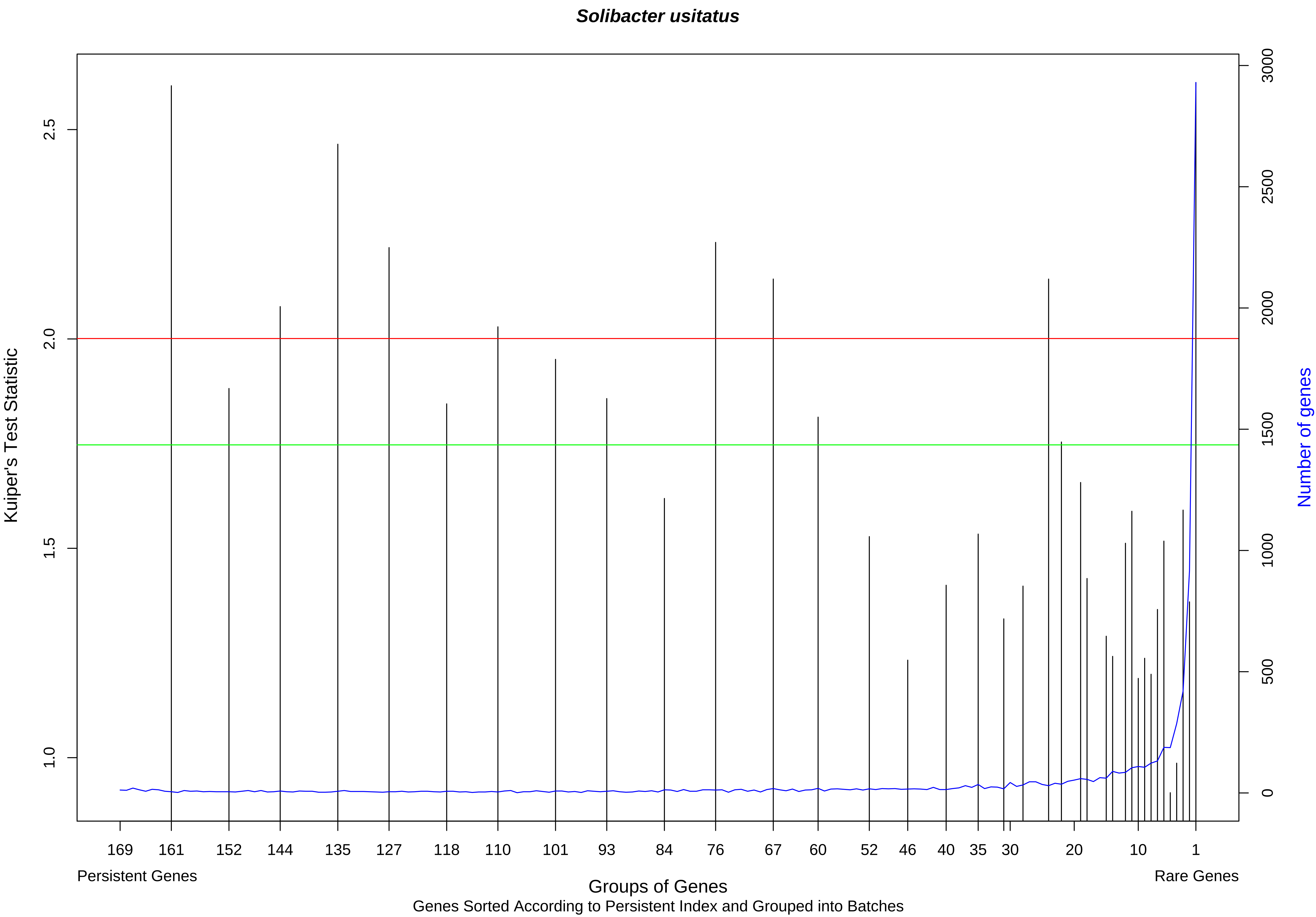

Solibacter usitatus
Kuiper's Test Statistic
Number of genes
2.5
2.0
1.5
1.0
3000
2500
2000
1500
1000
500
0
169
161
152
144
135
127
118
110
101
93
84
76
67
60
52
46
40
35
30
20
10
1
Persistent Genes
Rare Genes
Groups of Genes
Genes Sorted According to Persistent Index and Grouped into Batches

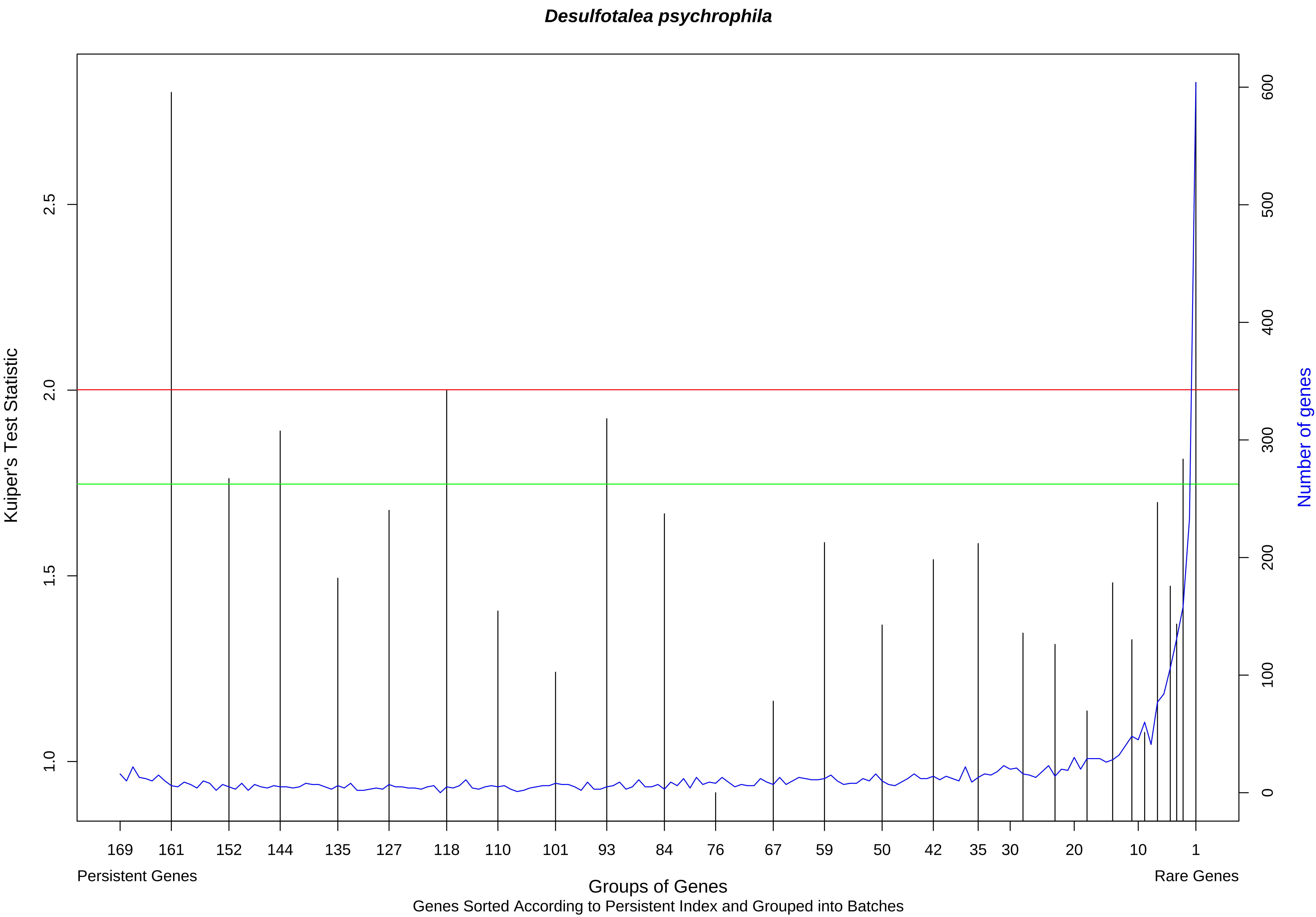

Desulfotalea psychrophila
Kuiper's Test Statistic
1.0
1.5
2.0
2.5
Number of genes
0
100
200
300
400
500
600
169
161
152
144
135
127
118
110
101
93
84
76
67
59
50
42
35
30
20
10
1
Persistent Genes
Rare Genes
Groups of Genes
Genes Sorted According to Persistent Index and Grouped into Batches

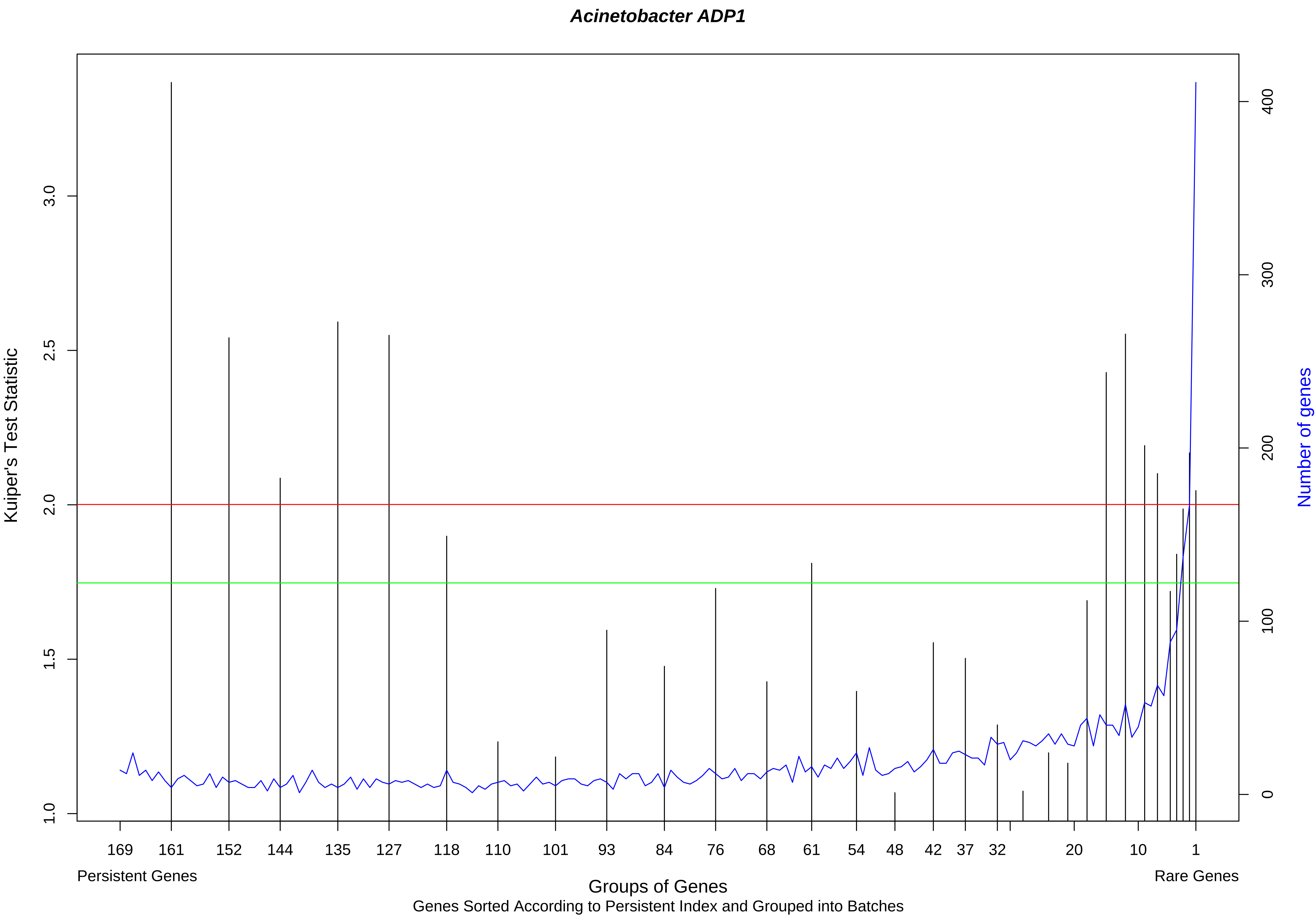
Acinetobacter ADP1
Kuiper's Test Statistic
Number of genes
1.0
1.5
2.0
2.5
3.0
0
100
200
300
400
169
161
152
144
135
127
118
110
101
93
84
76
68
61
54
48
42
37
32
20
10
1
Persistent Genes
Rare Genes
Groups of Genes
Genes Sorted According to Persistent Index and Grouped into Batches

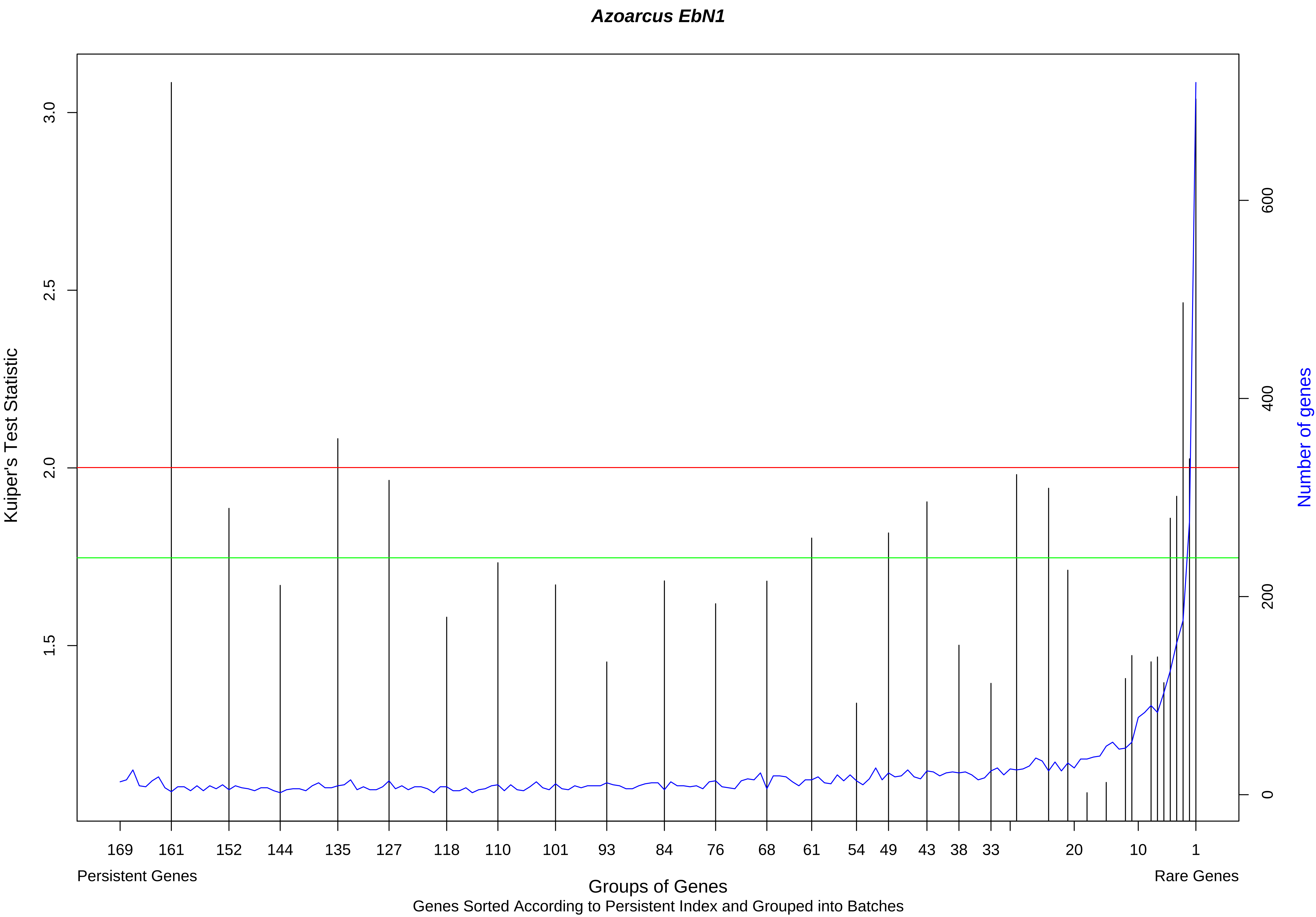

Azoarcus EbN1
Kuiper's Test Statistic
1.5
2.0
2.5
3.0
Number of genes
0
200
400
600
169
161
152
144
135
127
118
110
101
93
84
76
68
61
54
49
43
38
33
20
10
1
Persistent Genes
Rare Genes
Groups of Genes
Genes Sorted According to Persistent Index and Grouped into Batches

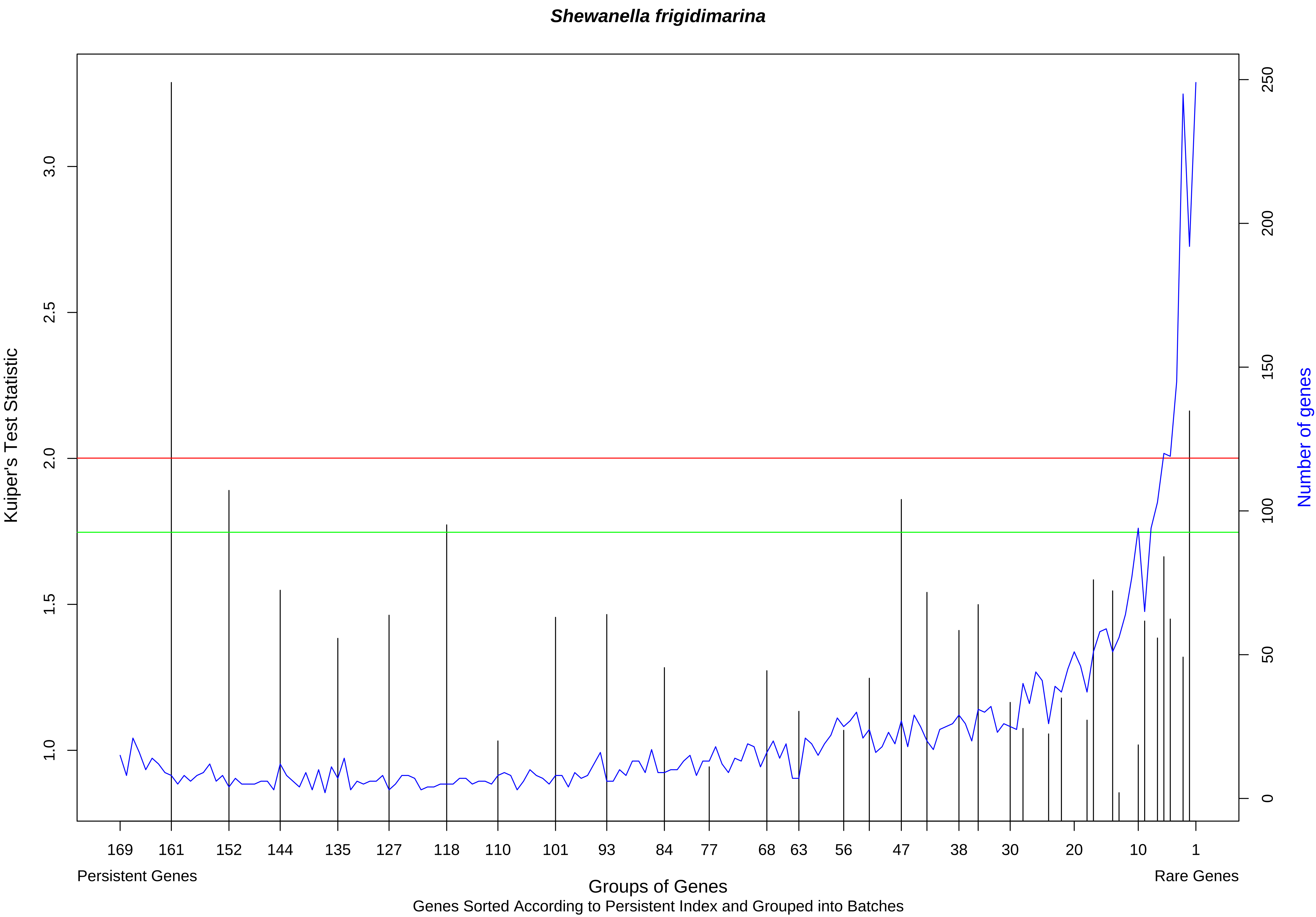
Shewanella frigidimarina
Kuiper's Test Statistic
Number of genes
3.0
2.5
2.0
1.5
1.0
250
200
150
100
50
0
169
161
152
144
135
127
118
110
101
93
84
77
68
63
56
47
38
30
20
10
1
Persistent Genes
Rare Genes
Groups of Genes
Genes Sorted According to Persistent Index and Grouped into Batches

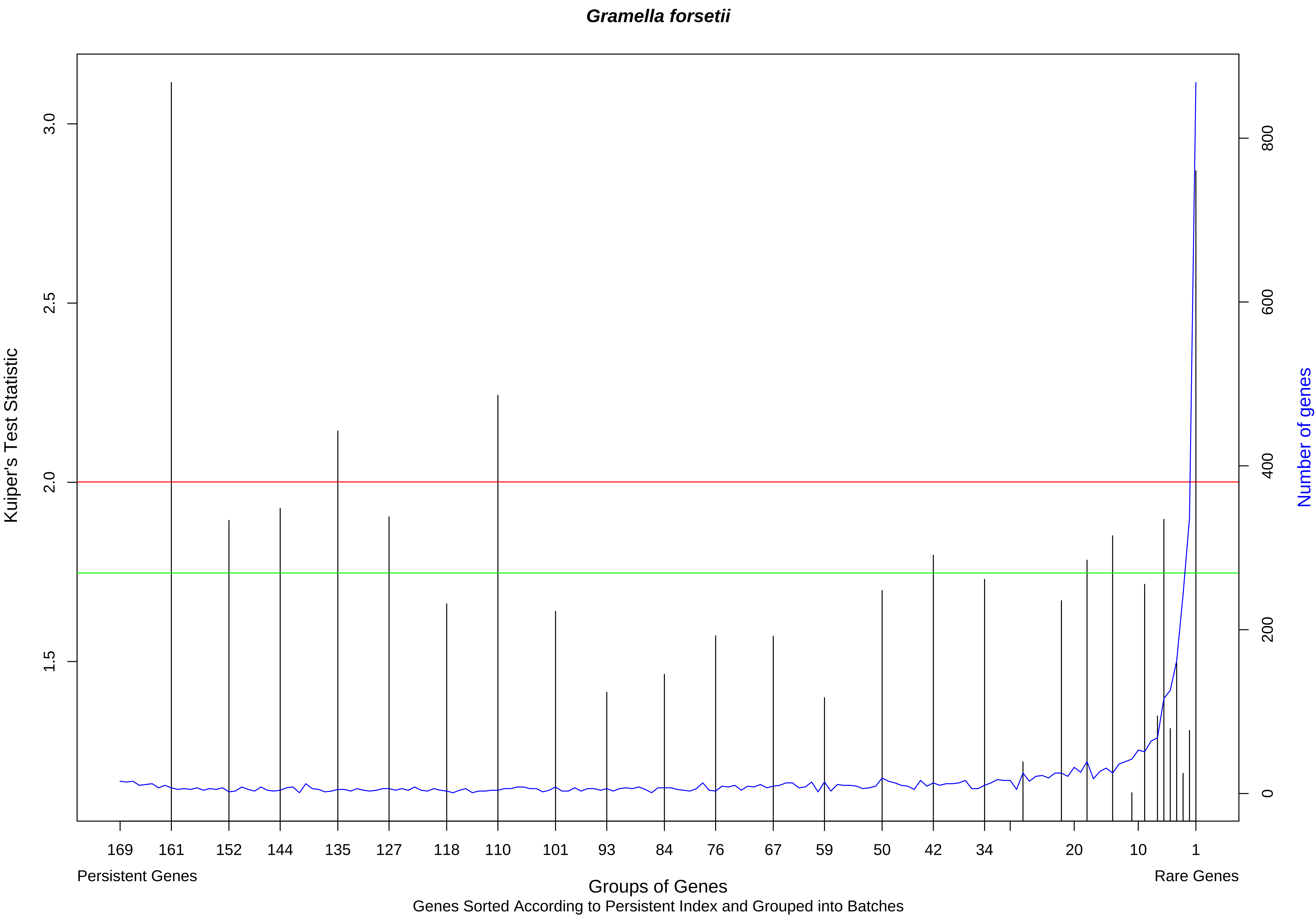

Gramella forsetii
Kuiper's Test Statistic
3.0
2.5
2.0
1.5
Number of genes
800
600
400
200
0
169
161
152
144
135
127
118
110
101
93
84
76
67
59
50
42
34
20
10
1
Persistent Genes
Rare Genes
Groups of Genes
Genes Sorted According to Persistent Index and Grouped into Batches

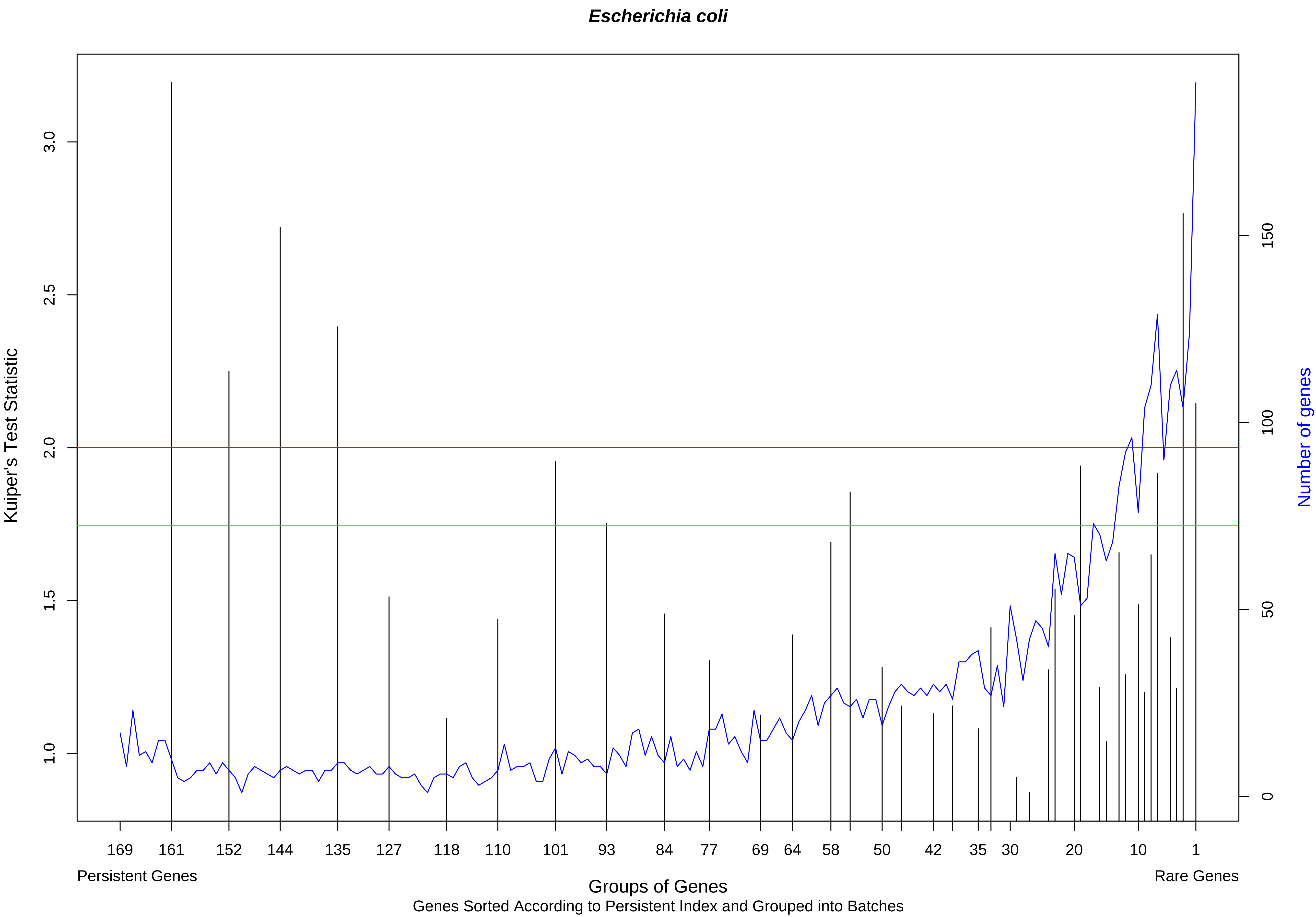

Escherichia coli
Kuiper's Test Statistic
3.0
2.5
2.0
1.5
1.0
Number of genes
150
100
50
0
169
161
152
144
135
127
118
110
101
93
84
77
69
64
58
50
42
35
30
20
10
1
Persistent Genes
Rare Genes
Groups of Genes
Genes Sorted According to Persistent Index and Grouped into Batches

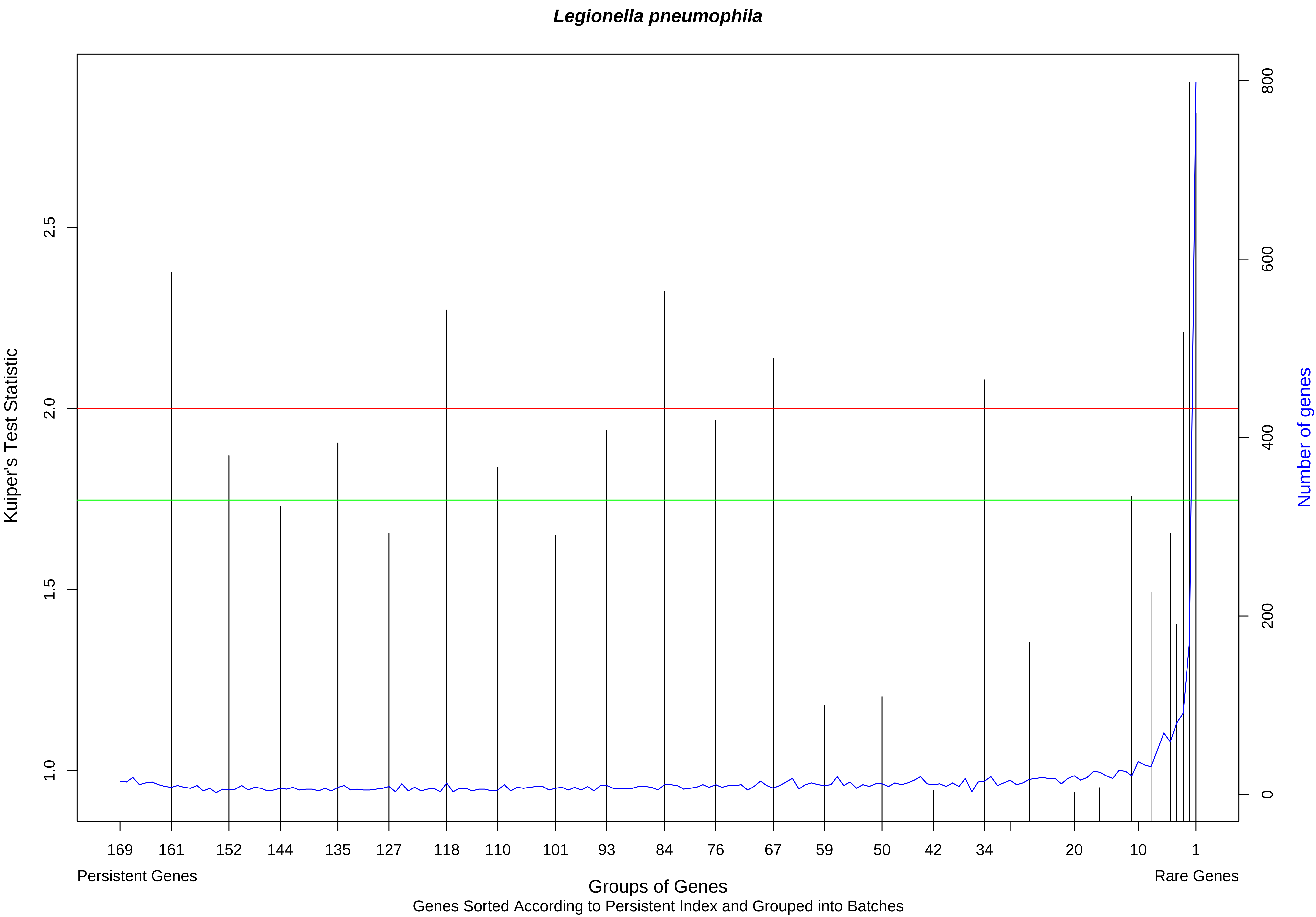

Legionella pneumophila
Kuiper's Test Statistic
1.0
1.5
2.0
2.5
Number of genes
0
200
400
600
800
169
161
152
144
135
127
118
110
101
93
84
76
67
59
50
42
34
20
10
1
Persistent Genes
Rare Genes
Groups of Genes
Genes Sorted According to Persistent Index and Grouped into Batches

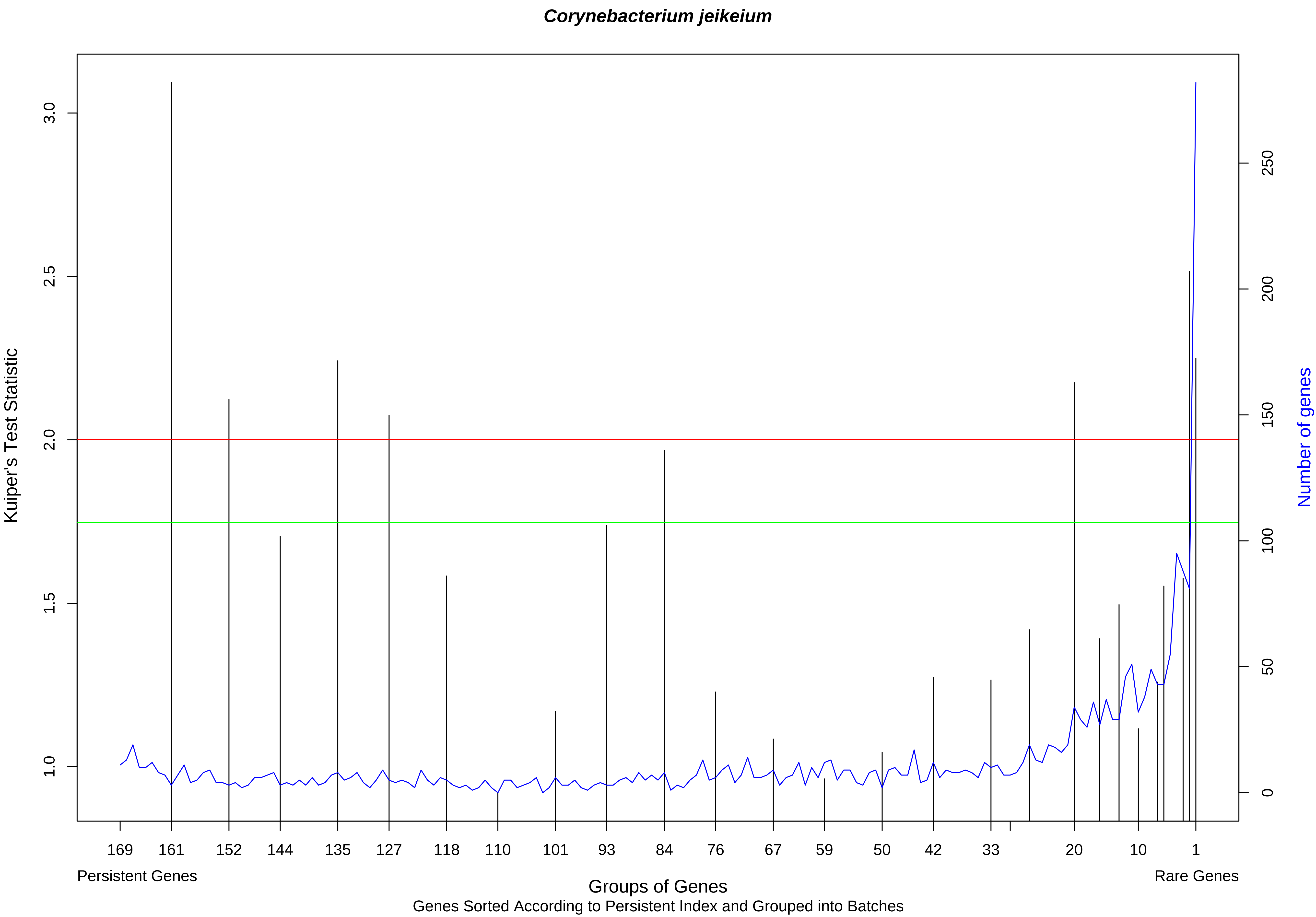

Corynebacterium jeikeium
Kuiper's Test Statistic
Number of genes
3.0
2.5
2.0
1.5
1.0
250
200
150
100
50
0
169 161 152 144 135 127 118 110 101 93 84 76 67 59 50 42 33 20 10 1
Persistent Genes
Rare Genes
Groups of Genes
Genes Sorted According to Persistent Index and Grouped into Batches

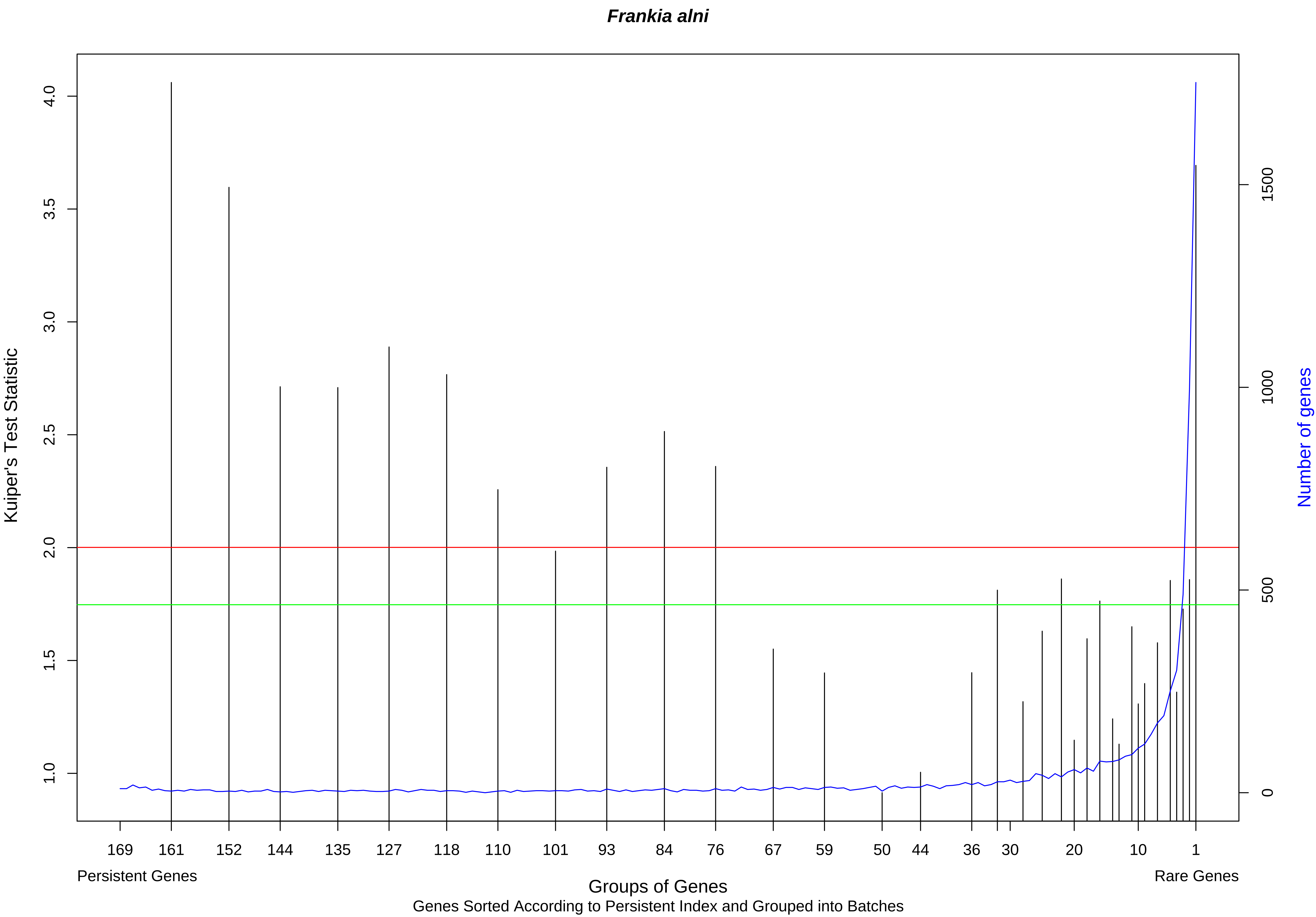

Frankia alni
Kuiper's Test Statistic
Number of genes
4.0
3.5
3.0
2.5
2.0
1.5
1.0
1500
1000
500
0
169
161
152
144
135
127
118
110
101
93
84
76
67
59
50
44
36
30
20
10
1
Persistent Genes
Rare Genes
Groups of Genes
Genes Sorted According to Persistent Index and Grouped into Batches

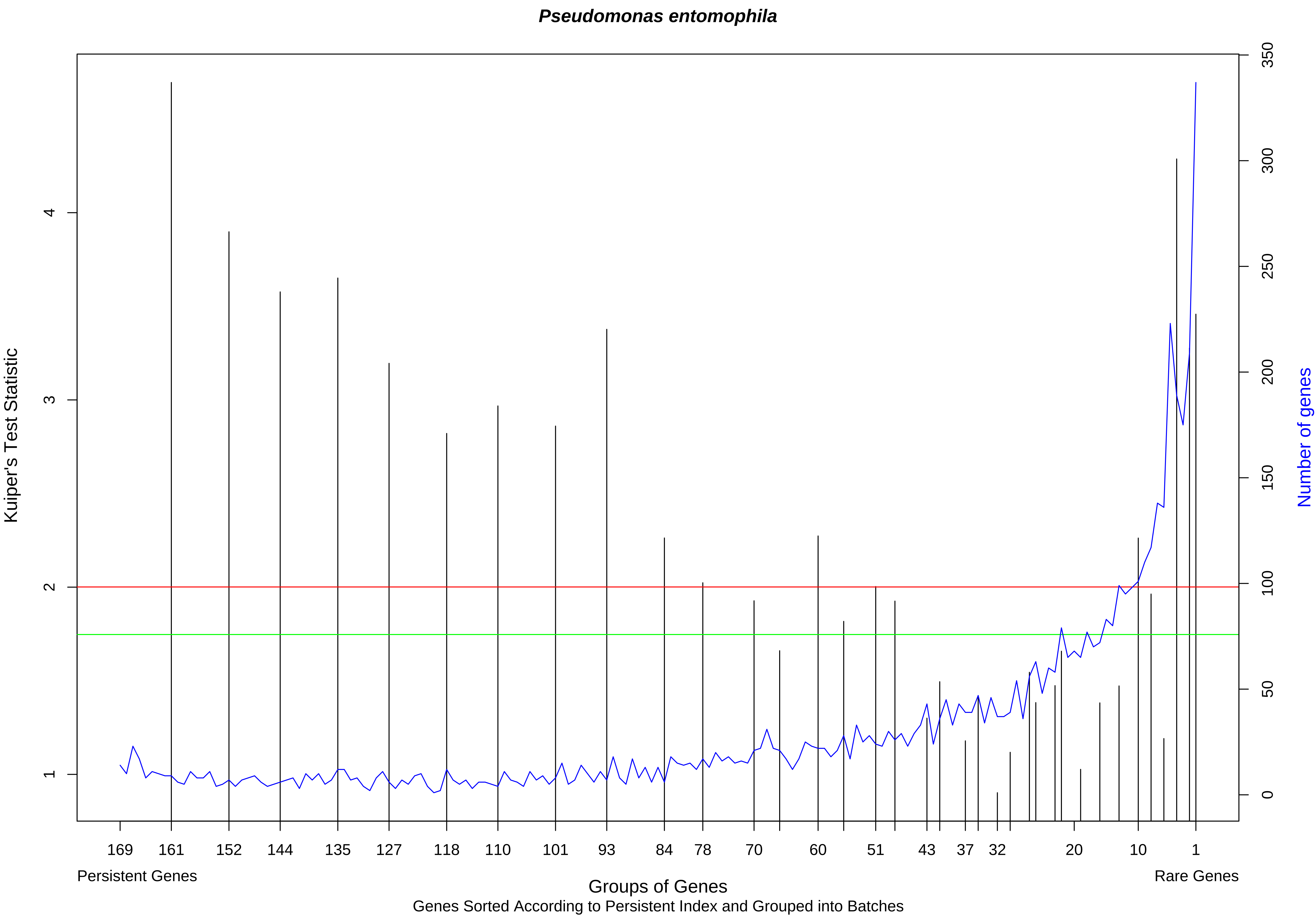

Pseudomonas entomophila
Kuiper's Test Statistic
Number of genes
1
2
3
4
0
50
100
150
200
250
300
350
169
161
152
144
135
127
118
110
101
93
84
78
70
60
51
43
37
32
20
10
1
Persistent Genes
Rare Genes
Groups of Genes
Genes Sorted According to Persistent Index and Grouped into Batches

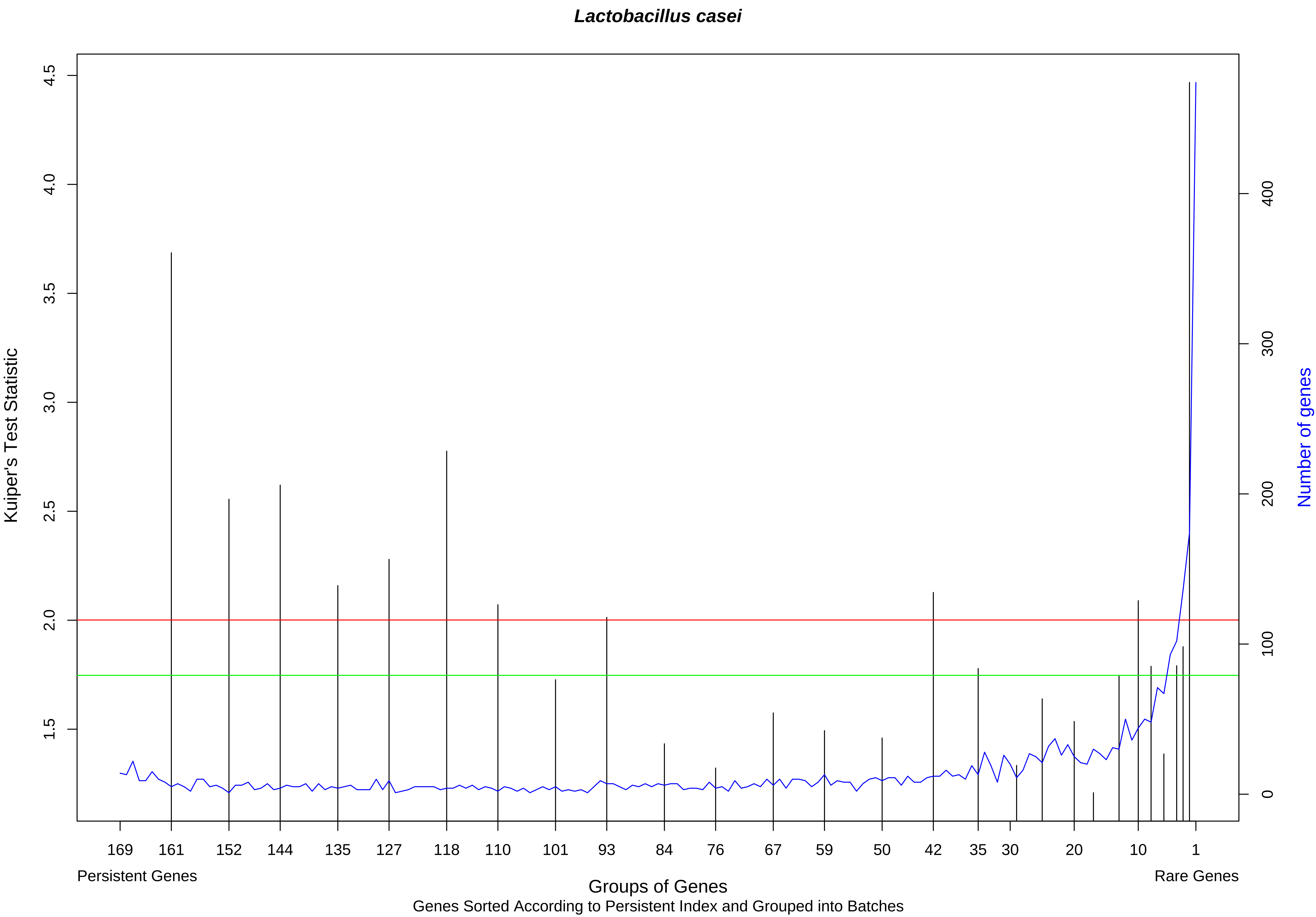

Lactobacillus casei
Kuiper's Test Statistic
Number of genes
1.5
2.0
2.5
3.0
3.5
4.0
4.5
0
100
200
300
400
169
161
152
144
135
127
118
110
101
93
84
76
67
59
50
42
35
30
20
10
1
Persistent Genes
Rare Genes
Groups of Genes
Genes Sorted According to Persistent Index and Grouped into Batches

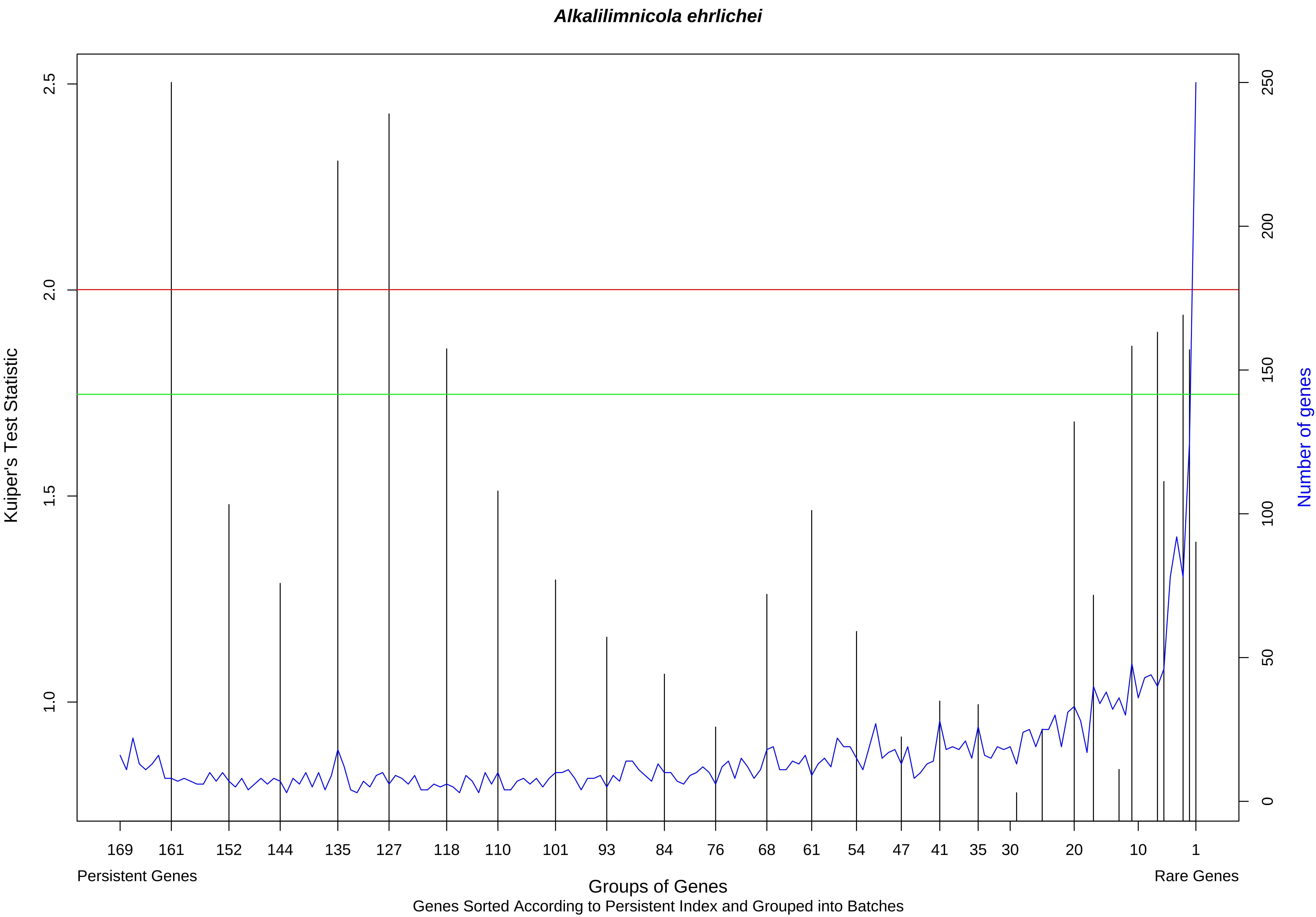
Alkalilimnicola ehrlichei
Kuiper's Test Statistic
2.5
2.0
1.5
1.0
Number of genes
250
200
150
100
50
0
169
161
152
144
135
127
118
110
101
93
84
76
68
61
54
47
41
35
30
20
10
1
Persistent Genes
Rare Genes
Groups of Genes
Genes Sorted According to Persistent Index and Grouped into Batches

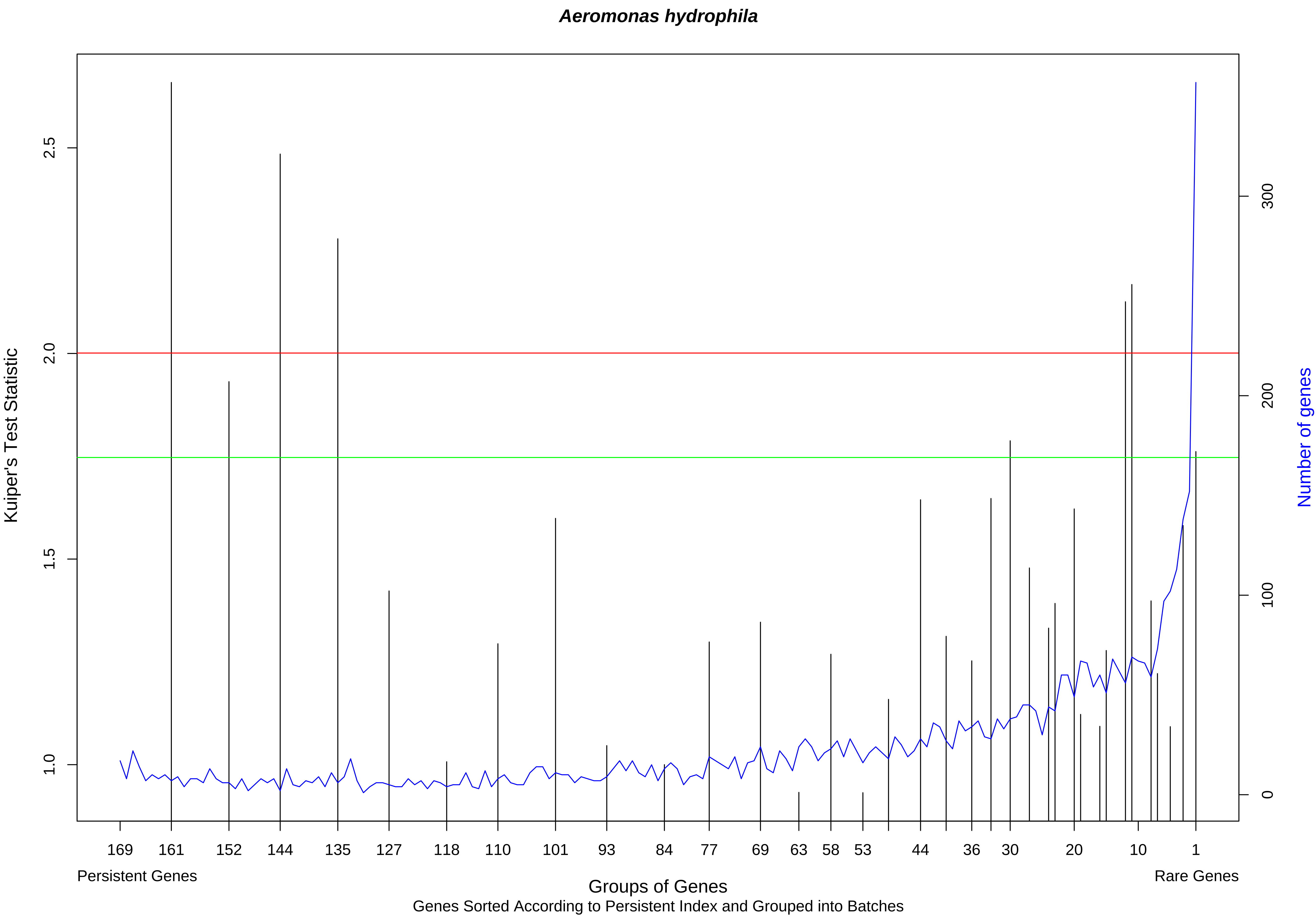
Aeromonas hydrophila
Kuiper's Test Statistic
2.5
2.0
1.5
1.0
Number of genes
300
200
100
0
169
161
152
144
135
127
118
110
101
93
84
77
69
63
58
53
44
36
30
20
10
1
Persistent Genes
Rare Genes
Groups of Genes
Genes Sorted According to Persistent Index and Grouped into Batches

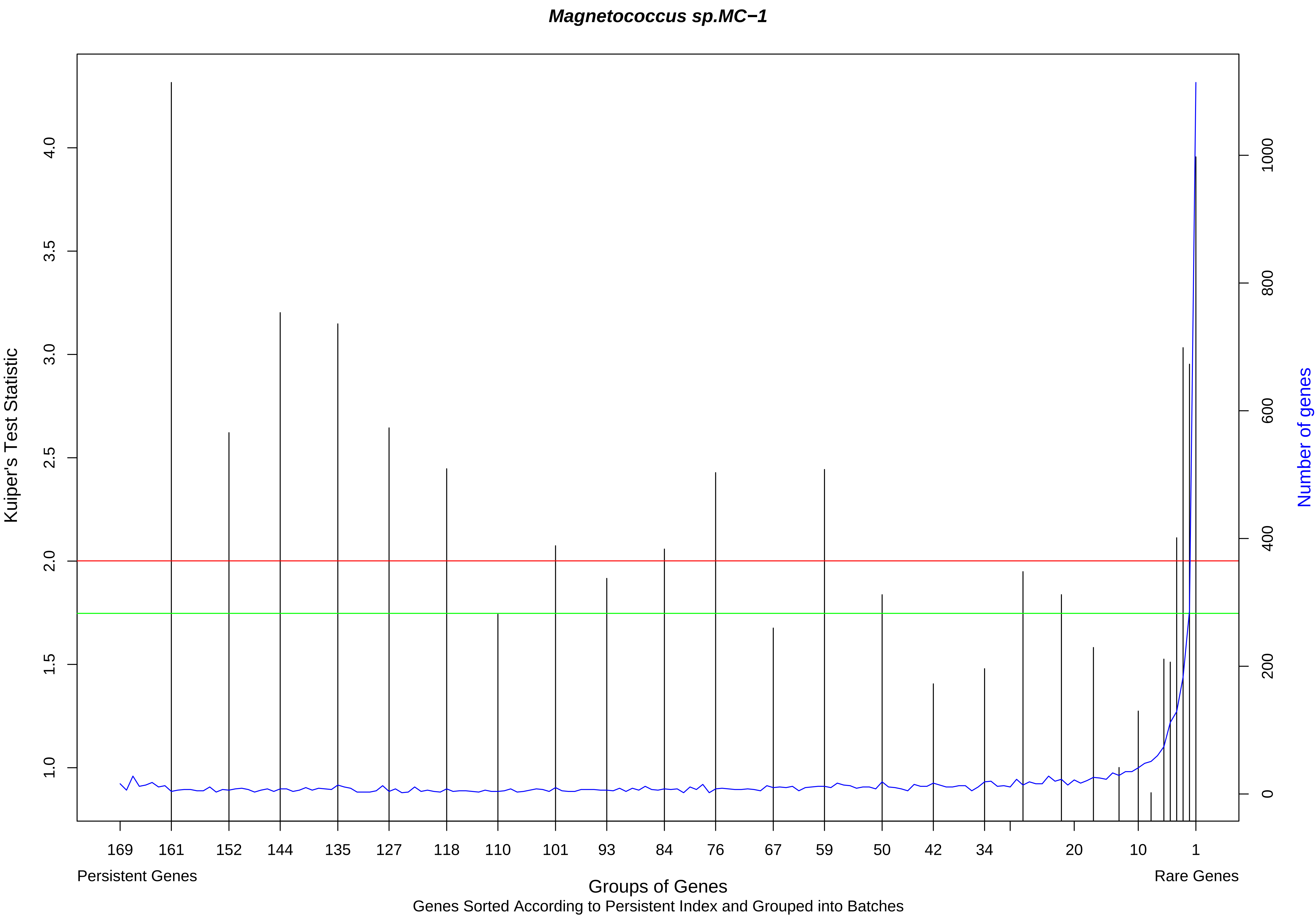
Magnetococcus sp.MC−1
Kuiper's Test Statistic
1.0
1.5
2.0
2.5
3.0
3.5
4.0
Number of genes
0
200
400
600
800
1000
169
161
152
144
135
127
118
110
101
93
84
76
67
59
50
42
34
20
10
1
Persistent Genes
Rare Genes
Groups of Genes
Genes Sorted According to Persistent Index and Grouped into Batches

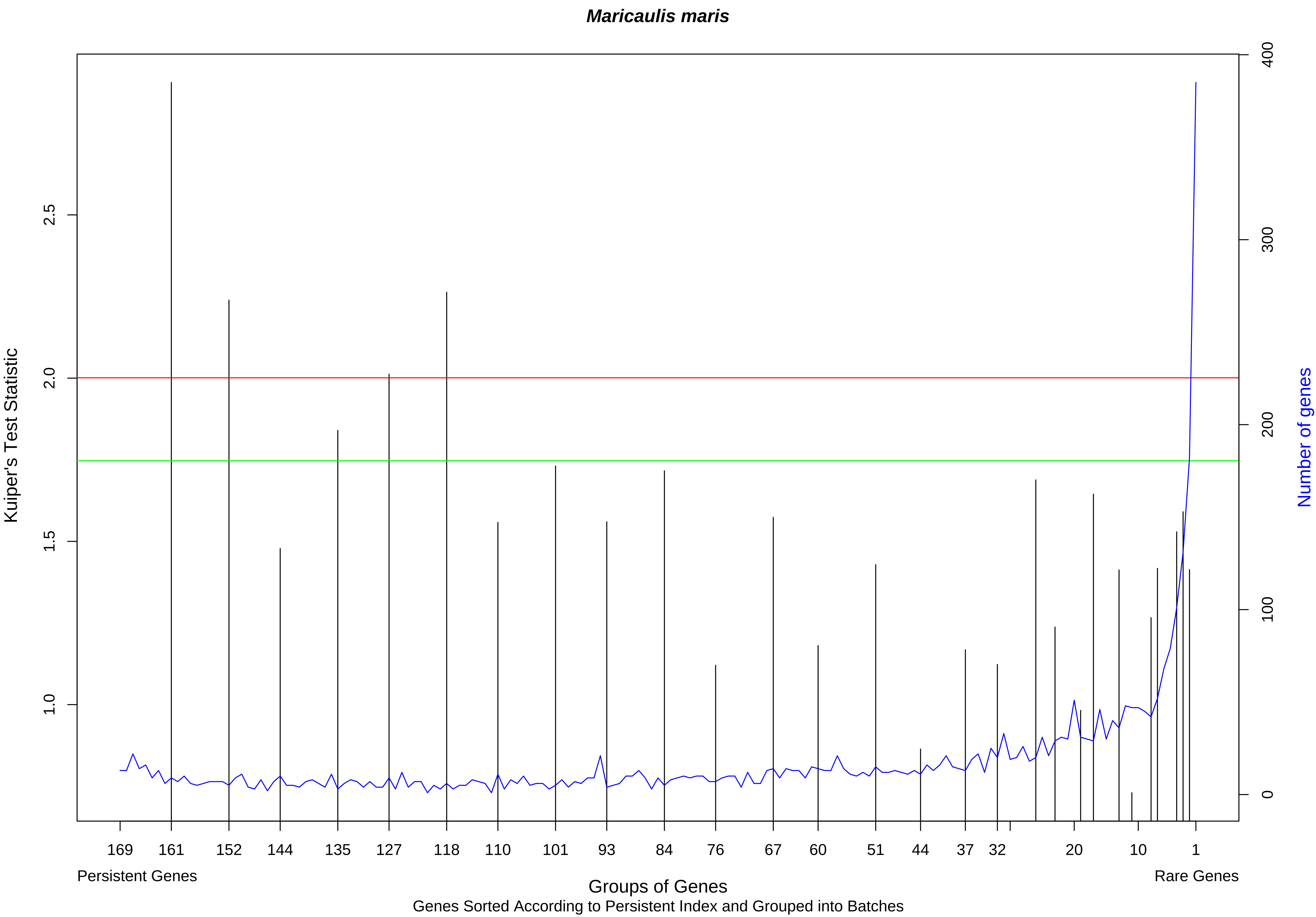
Maricaulis maris
Kuiper's Test Statistic
1.0
1.5
2.0
2.5
Number of genes
0
100
200
300
400
169
161
152
144
135
127
118
110
101
93
84
76
67
60
51
44
37
32
20
10
1
Persistent Genes
Rare Genes
Groups of Genes
Genes Sorted According to Persistent Index and Grouped into Batches

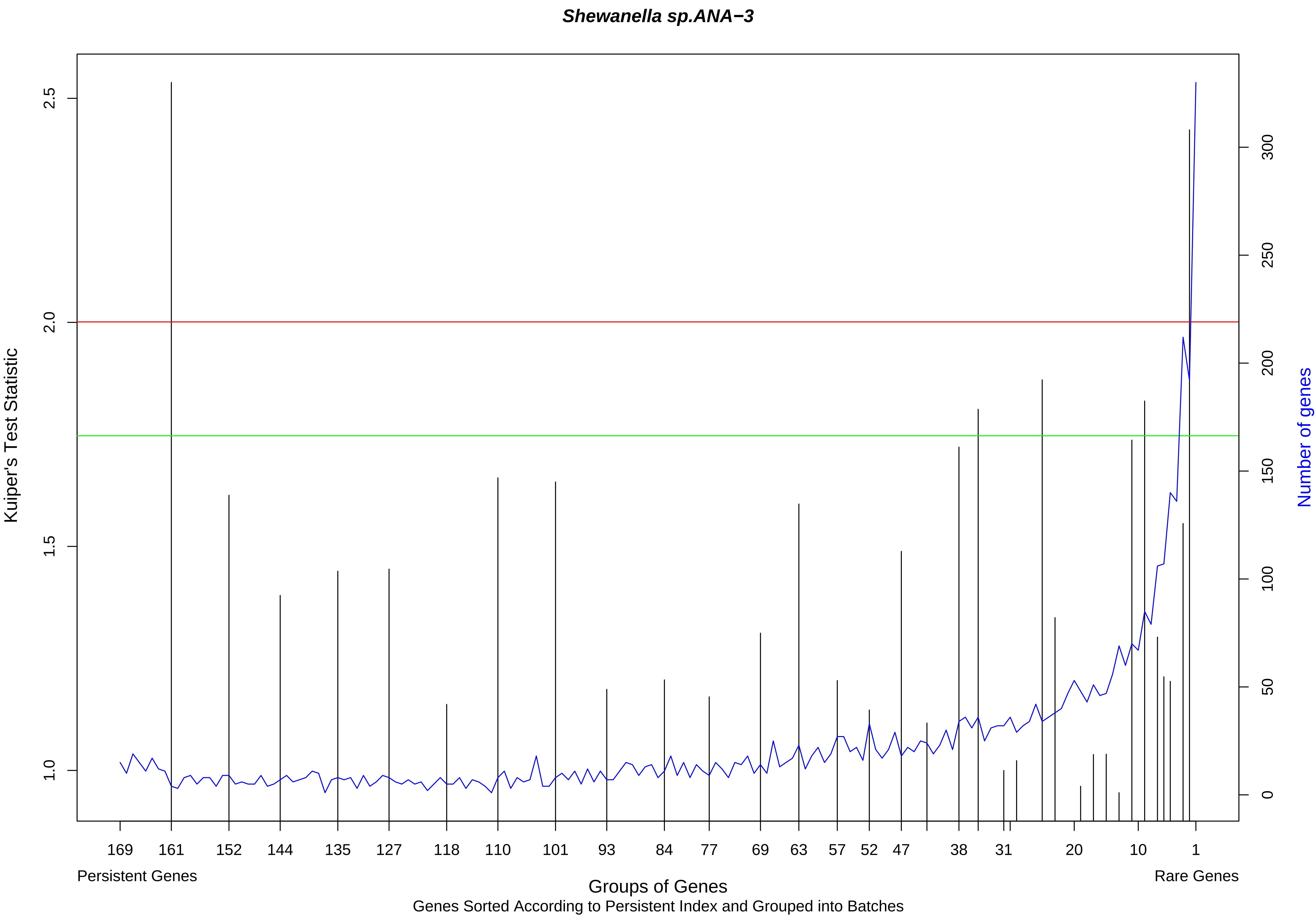
Shewanella sp.ANA−3
Kuiper's Test Statistic
Number of genes
2.5
2.0
1.5
1.0
300
250
200
150
100
50
0
169
161
152
144
135
127
118
110
101
93
84
77
69
63
57
52
47
38
31
20
10
1
Persistent Genes
Rare Genes
Groups of Genes
Genes Sorted According to Persistent Index and Grouped into Batches

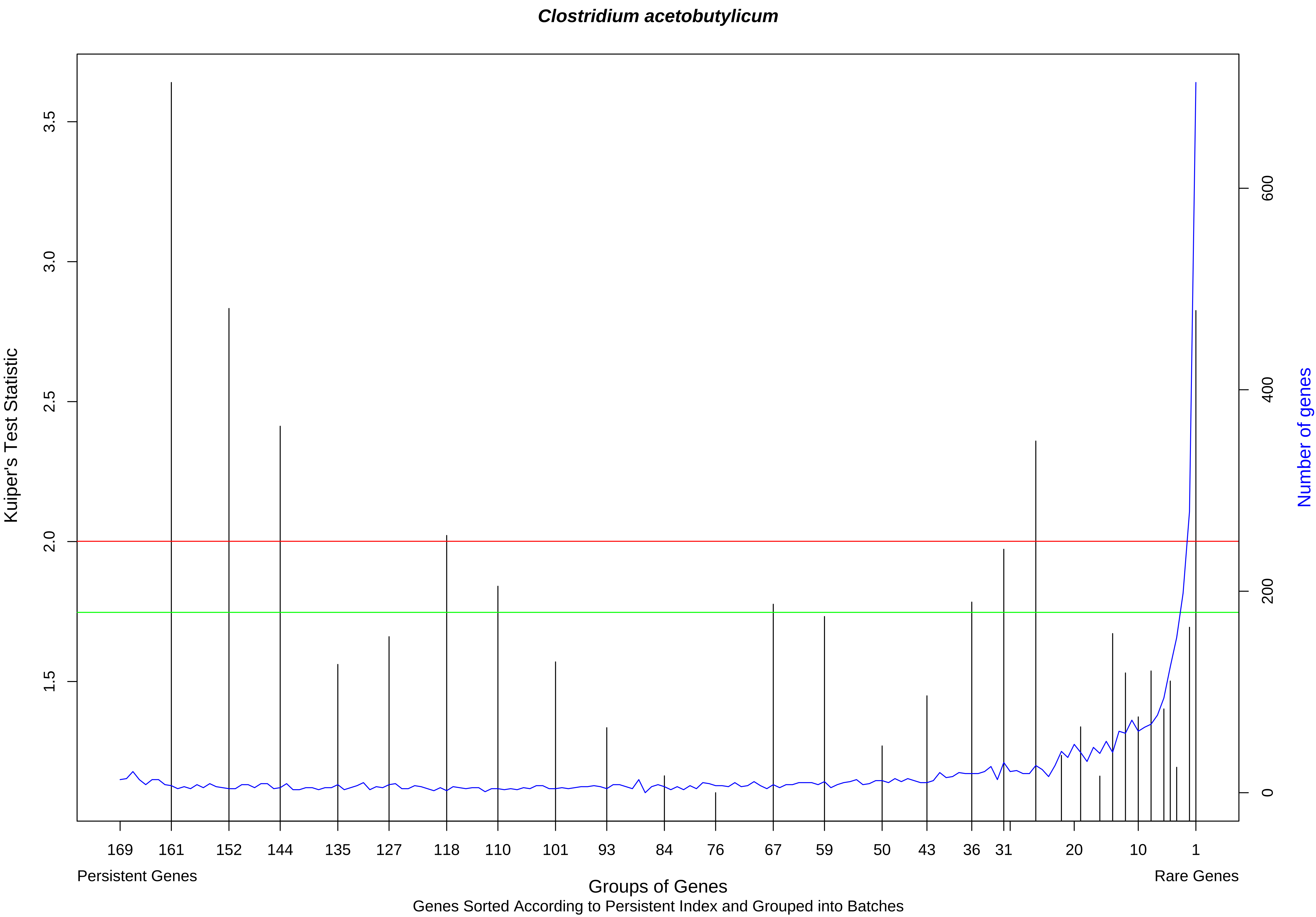

Clostridium acetobutylicum
Kuiper's Test Statistic
3.5
3.0
2.5
2.0
1.5
Number of genes
600
400
200
0
169
161
152
144
135
127
118
110
101
93
84
76
67
59
50
43
36
31
20
10
1
Persistent Genes
Rare Genes
Groups of Genes
Genes Sorted According to Persistent Index and Grouped into Batches

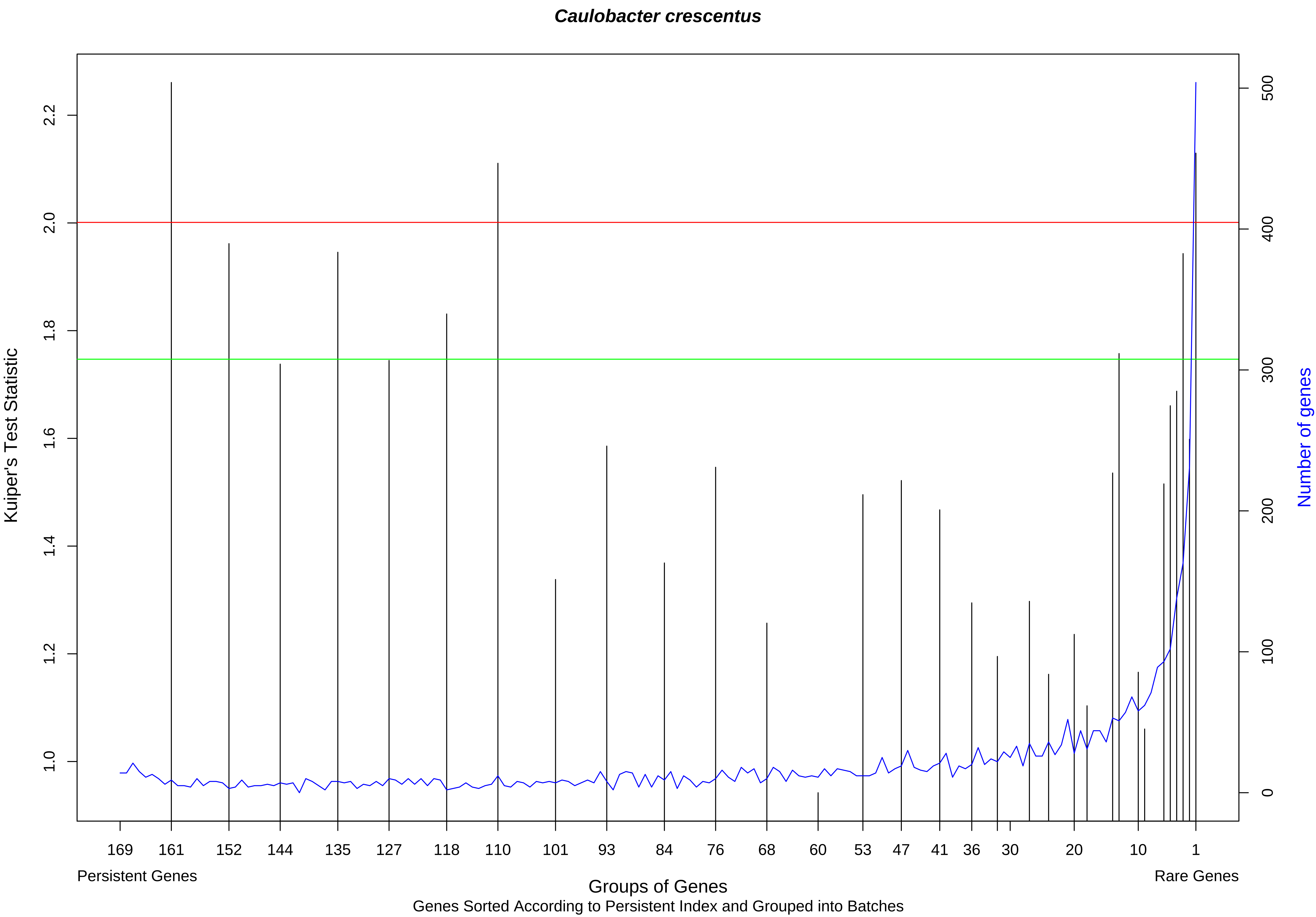
Caulobacter crescentus
Kuiper's Test Statistic
1.0
1.2
1.4
1.6
1.8
2.0
2.2
Number of genes
0
100
200
300
400
500
169 161 152 144 135 127 118 110 101 93 84 76 68 60 53 47 41 36 30 20 10 1
Persistent Genes
Rare Genes
Groups of Genes
Genes Sorted According to Persistent Index and Grouped into Batches

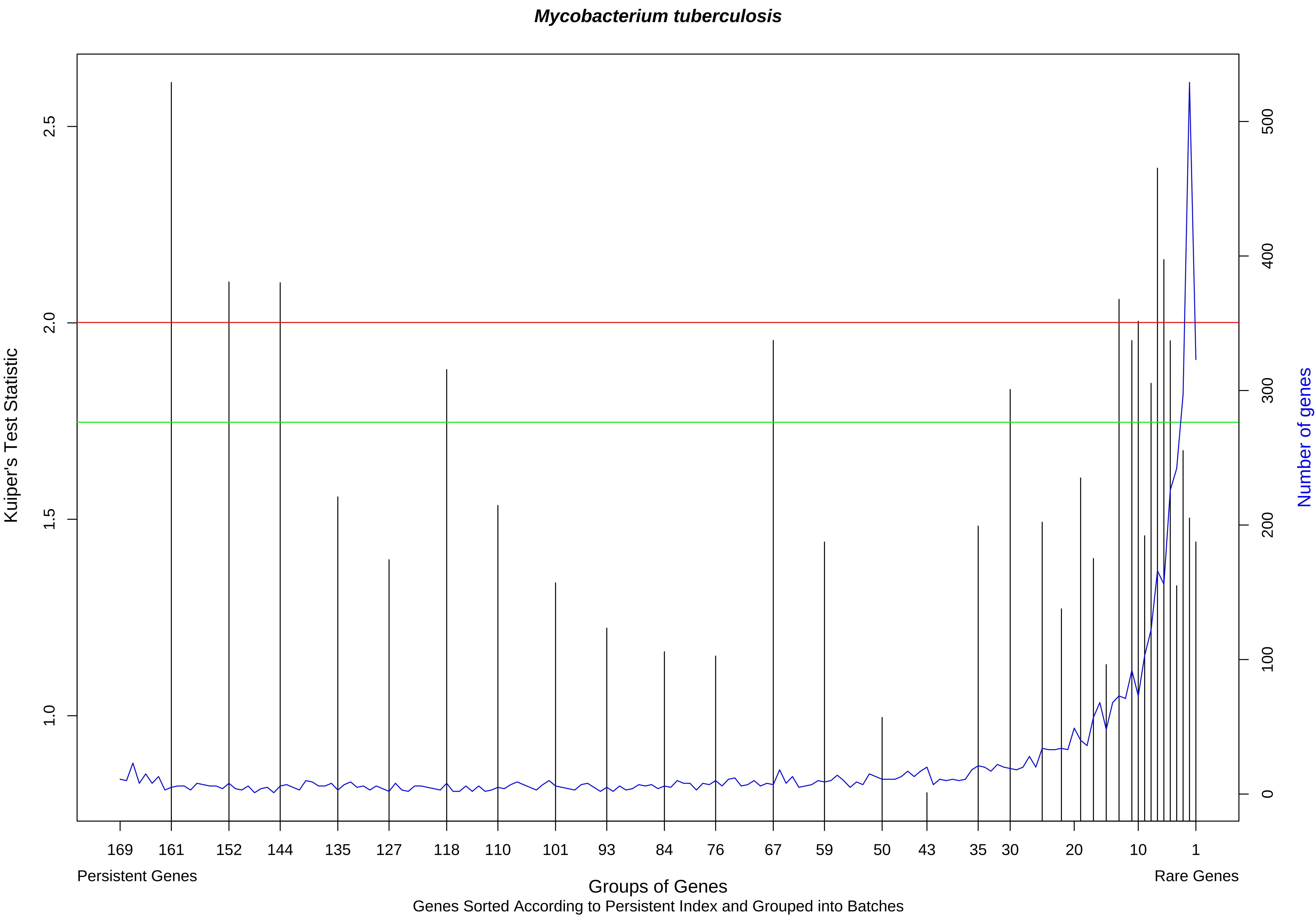

Mycobacterium tuberculosis
Kuiper's Test Statistic
Number of genes
1.0
1.5
2.0
2.5
0
100
200
300
400
500
169
161
152
144
135
127
118
110
101
93
84
76
67
59
50
43
35
30
20
10
1
Persistent Genes
Rare Genes
Groups of Genes
Genes Sorted According to Persistent Index and Grouped into Batches

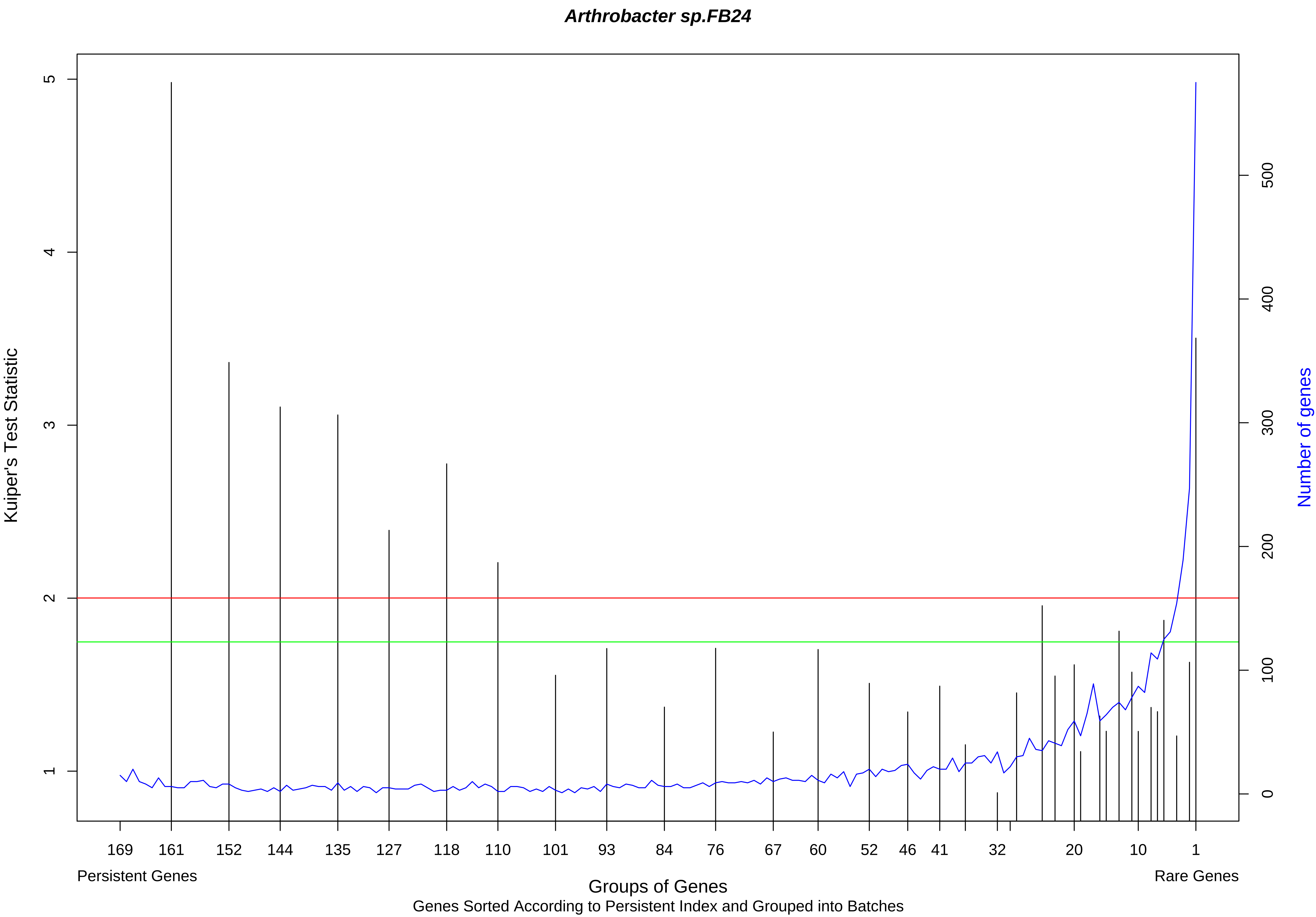
Arthrobacter sp.FB24
Kuiper's Test Statistic
Number of genes
5
4
3
2
1
500
400
300
200
100
0
169
161
152
144
135
127
118
110
101
93
84
76
67
60
52
46
41
32
20
10
1
Persistent Genes
Rare Genes
Groups of Genes
Genes Sorted According to Persistent Index and Grouped into Batches

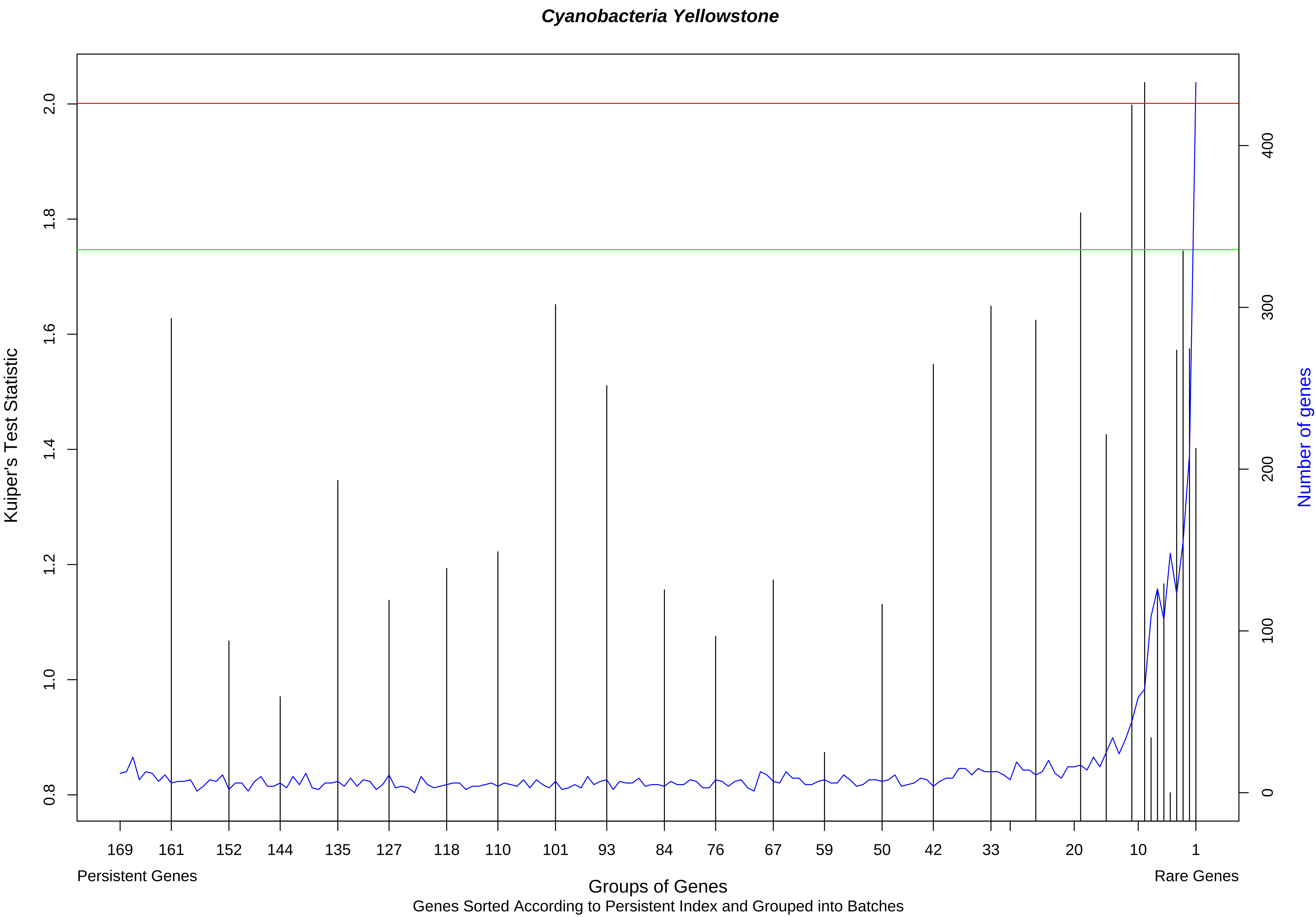

Cyanobacteria Yellowstone
Kuiper's Test Statistic
0.8
1.0
1.2
1.4
1.6
1.8
2.0
Number of genes
0
100
200
300
400
169
161
152
144
135
127
118
110
101
93
84
76
67
59
50
42
33
20
10
1
Persistent Genes
Rare Genes
Groups of Genes
Genes Sorted According to Persistent Index and Grouped into Batches

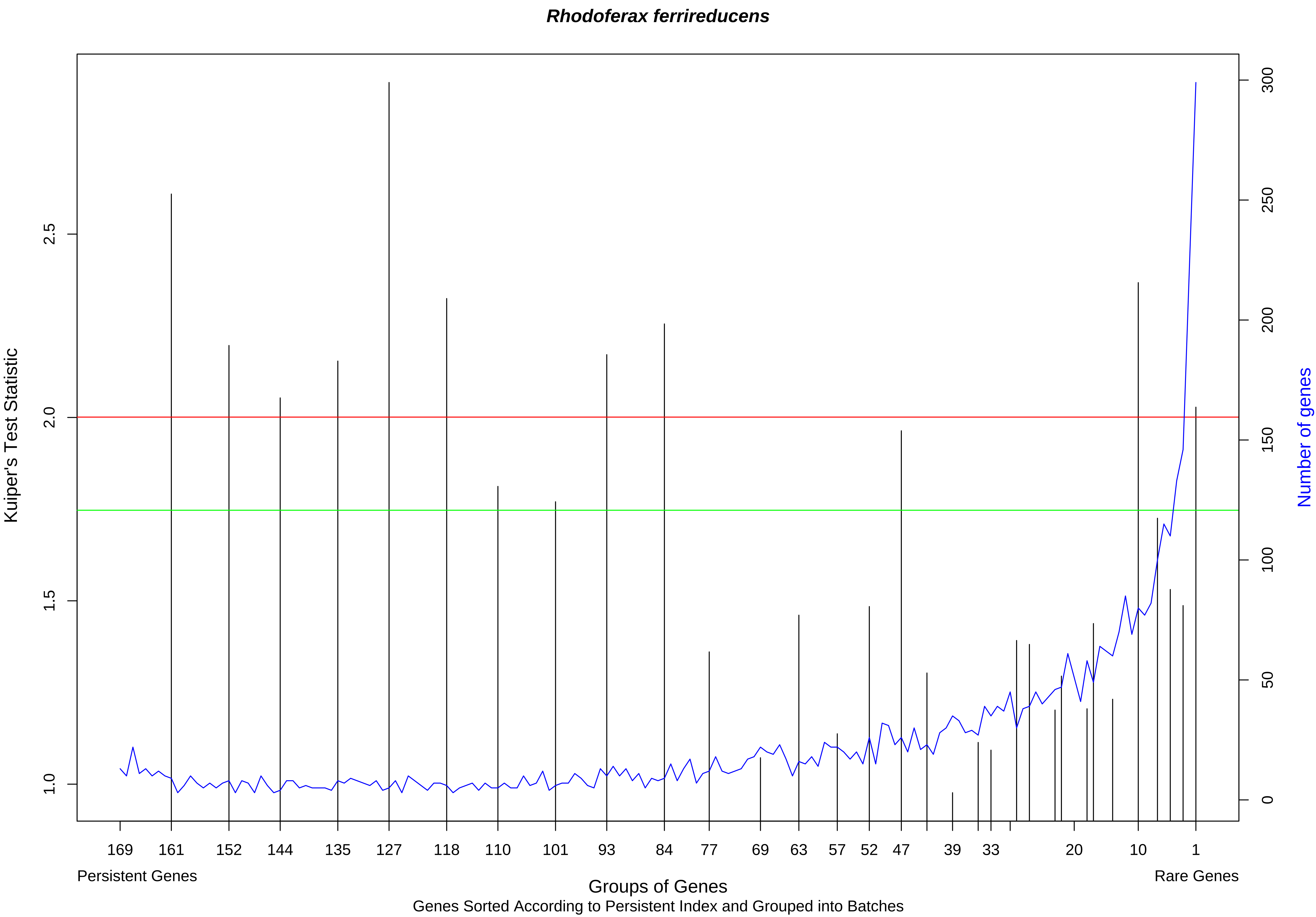

Rhodoferax ferrireducens
Kuiper's Test Statistic
Number of genes
1.0
1.5
2.0
2.5
0
50
100
150
200
250
300
169
161
152
144
135
127
118
110
101
93
84
77
69
63
57
52
47
39
33
20
10
1
Persistent Genes
Rare Genes
Groups of Genes
Genes Sorted According to Persistent Index and Grouped into Batches

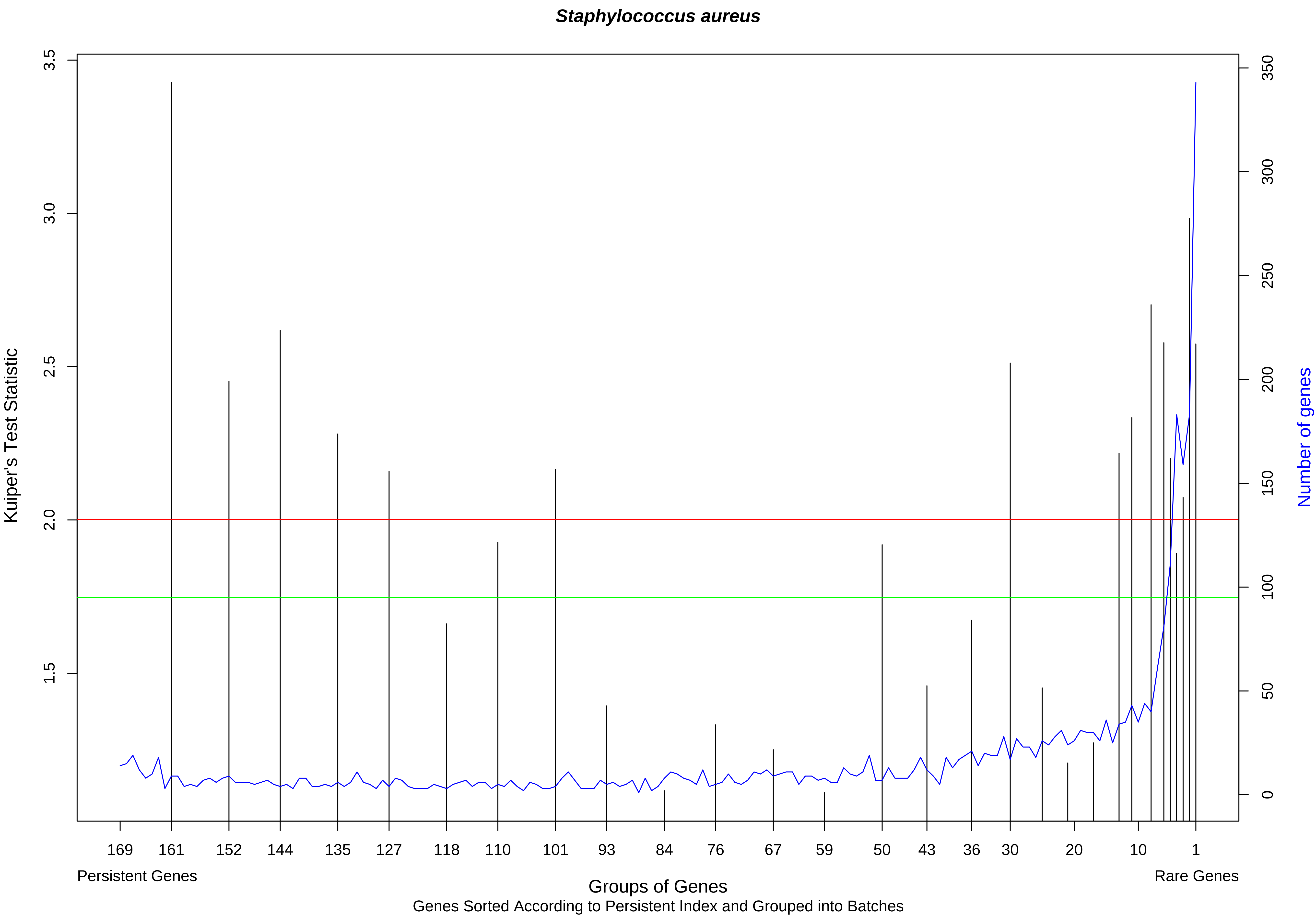

Staphylococcus aureus
Kuiper's Test Statistic
3.5
3.0
2.5
2.0
1.5
Number of genes
350
300
250
200
150
100
50
0
169
161
152
144
135
127
118
110
101
93
84
76
67
59
50
43
36
30
20
10
1
Persistent Genes
Rare Genes
Groups of Genes
Genes Sorted According to Persistent Index and Grouped into Batches

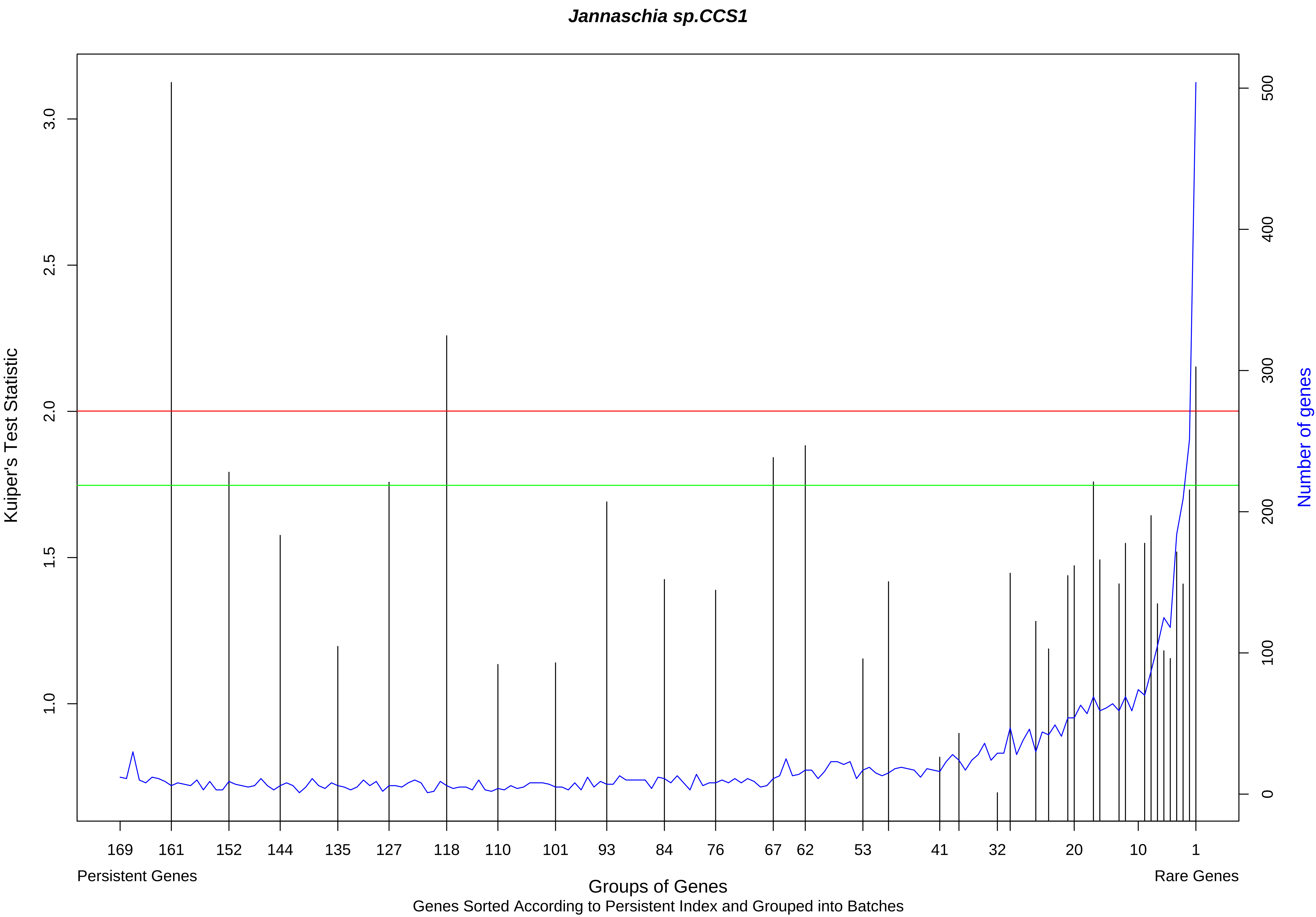

Jannaschia sp.CCS1
Kuiper's Test Statistic
Number of genes
3.0
2.5
2.0
1.5
1.0
500
400
300
200
100
0
169
161
152
144
135
127
118
110
101
93
84
76
67
62
53
41
32
20
10
1
Persistent Genes
Rare Genes
Groups of Genes
Genes Sorted According to Persistent Index and Grouped into Batches

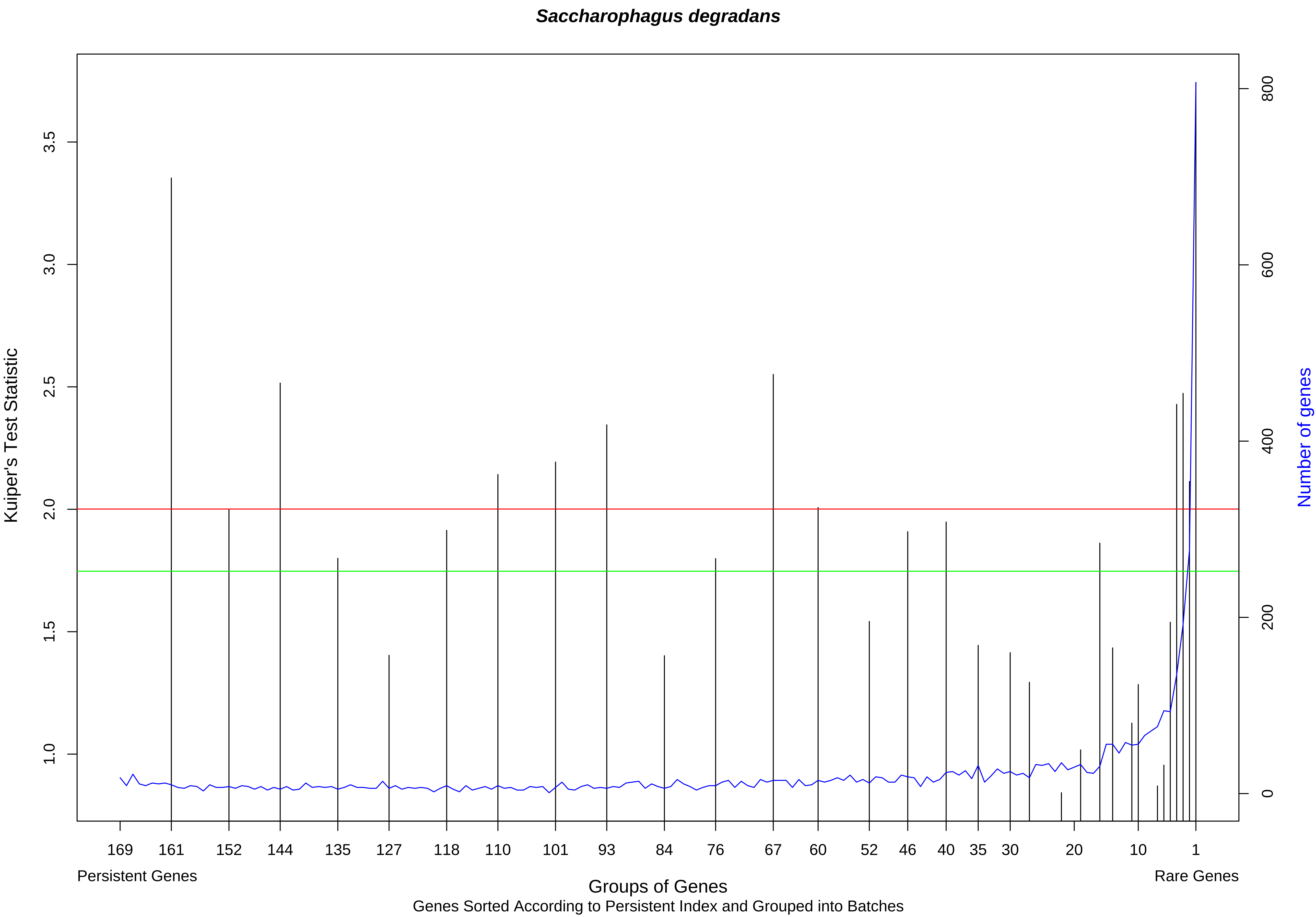

Saccharophagus degradans
Kuiper's Test Statistic
3.5
3.0
2.5
2.0
1.5
1.0
Number of genes
800
600
400
200
0
169
161
152
144
135
127
118
110
101
93
84
76
67
60
52
46
40
35
30
20
10
1
Persistent Genes
Rare Genes
Groups of Genes
Genes Sorted According to Persistent Index and Grouped into Batches

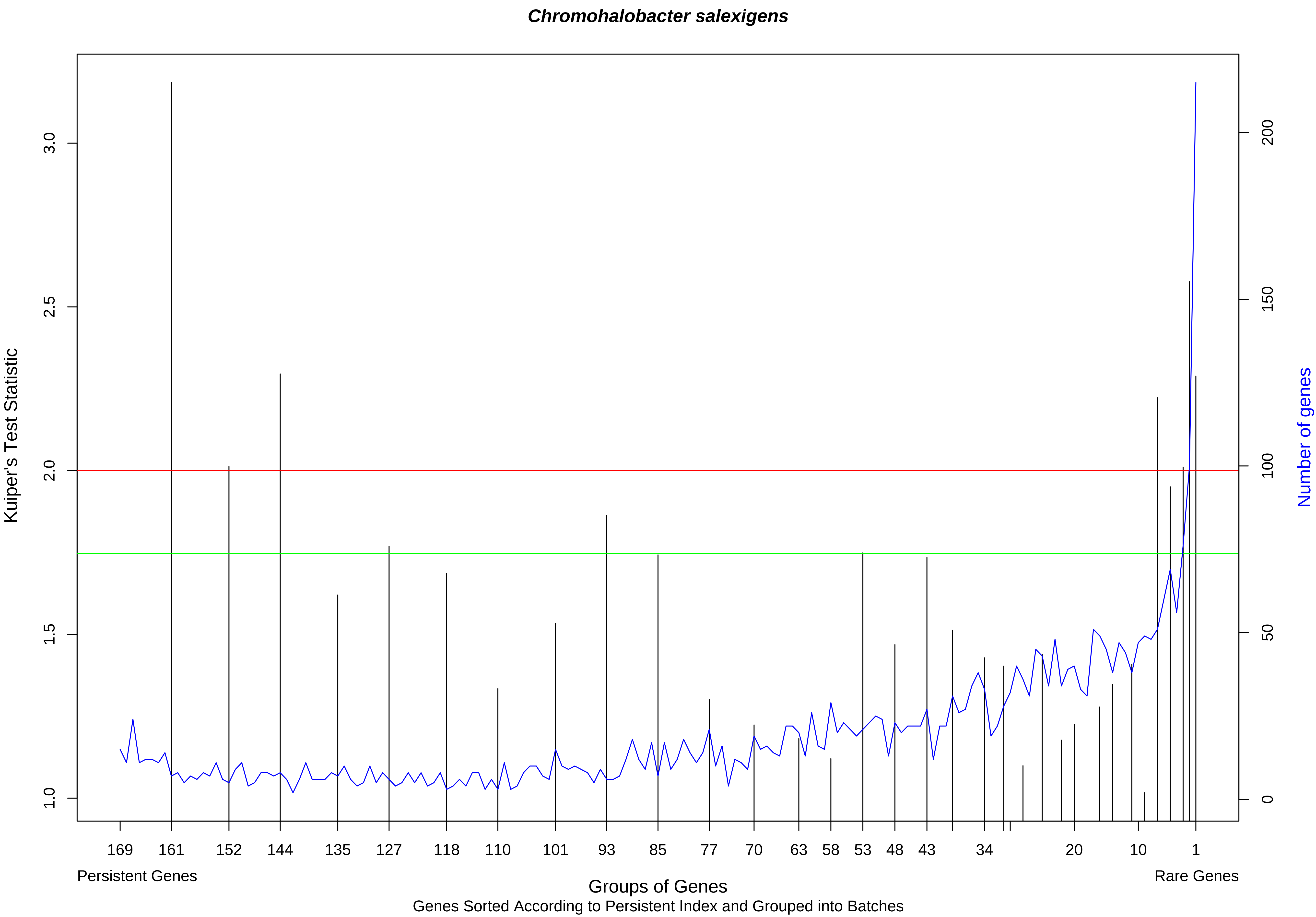

Chromohalobacter salexigens
Kuiper's Test Statistic
Number of genes
1.0
1.5
2.0
2.5
3.0
0
50
100
150
200
169
161
152
144
135
127
118
110
101
93
85
77
70
63
58
53
48
43
34
20
10
1
Persistent Genes
Rare Genes
Groups of Genes
Genes Sorted According to Persistent Index and Grouped into Batches

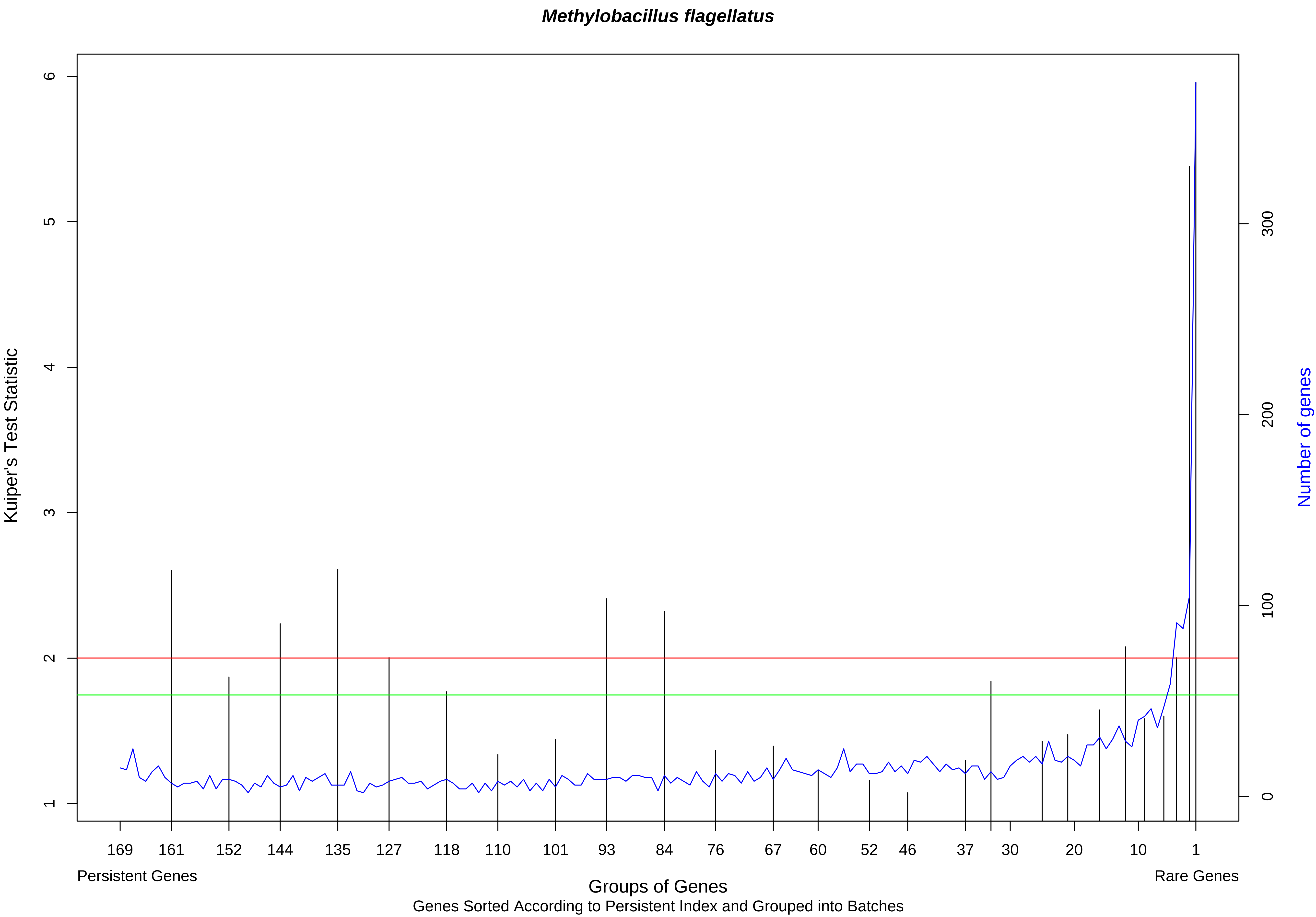

Methylobacillus flagellatus
Kuiper's Test Statistic
1
2
3
4
5
6
Number of genes
0
100
200
300
169
161
152
144
135
127
118
110
101
93
84
76
67
60
52
46
37
30
20
10
1
Persistent Genes
Rare Genes
Groups of Genes
Genes Sorted According to Persistent Index and Grouped into Batches

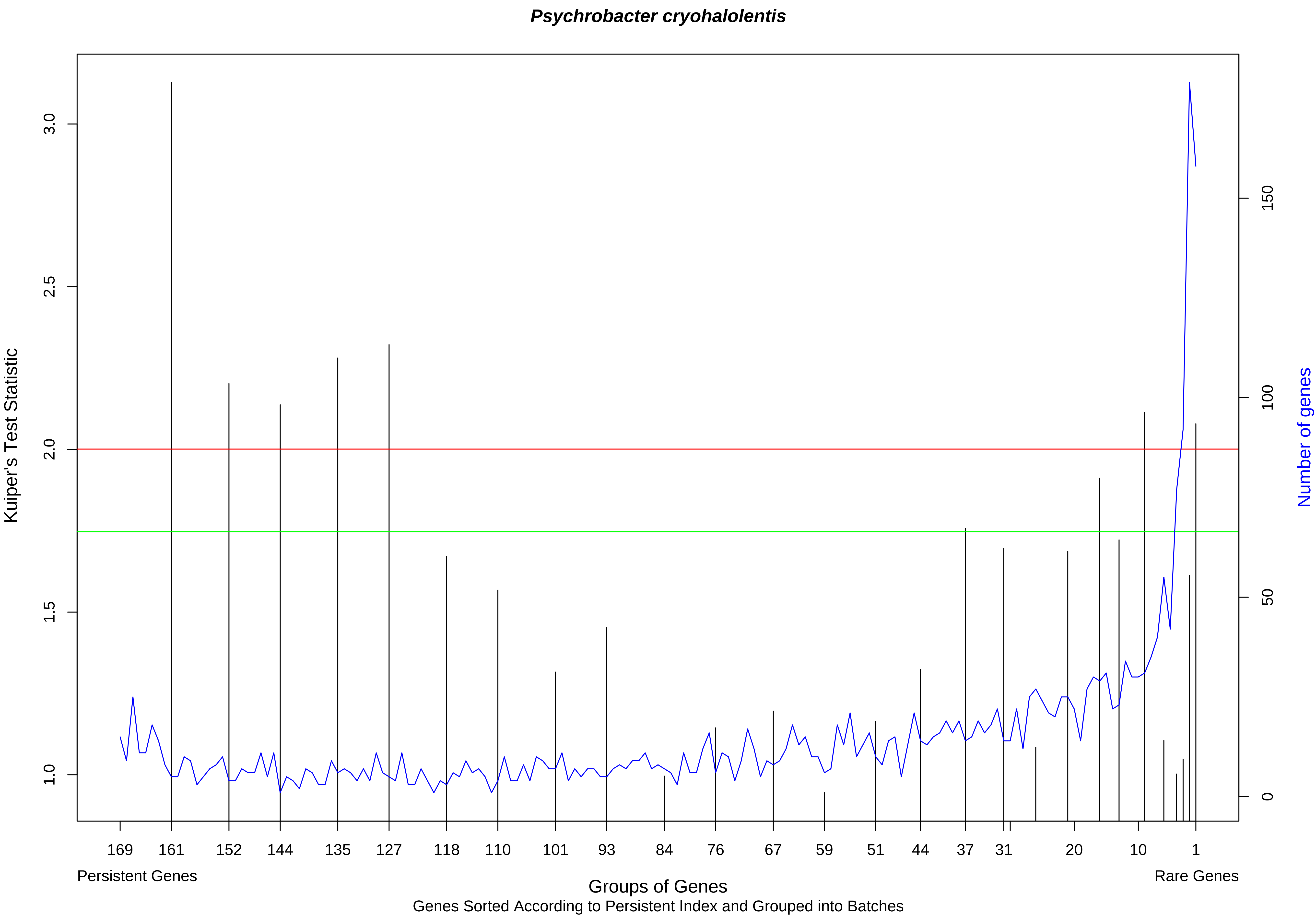

Psychrobacter cryohalolentis
Kuiper's Test Statistic
3.0
2.5
2.0
1.5
1.0
Number of genes
150
100
50
0
169
161
152
144
135
127
118
110
101
93
84
76
67
59
51
44
37
31
20
10
1
Persistent Genes
Rare Genes
Groups of Genes
Genes Sorted According to Persistent Index and Grouped into Batches

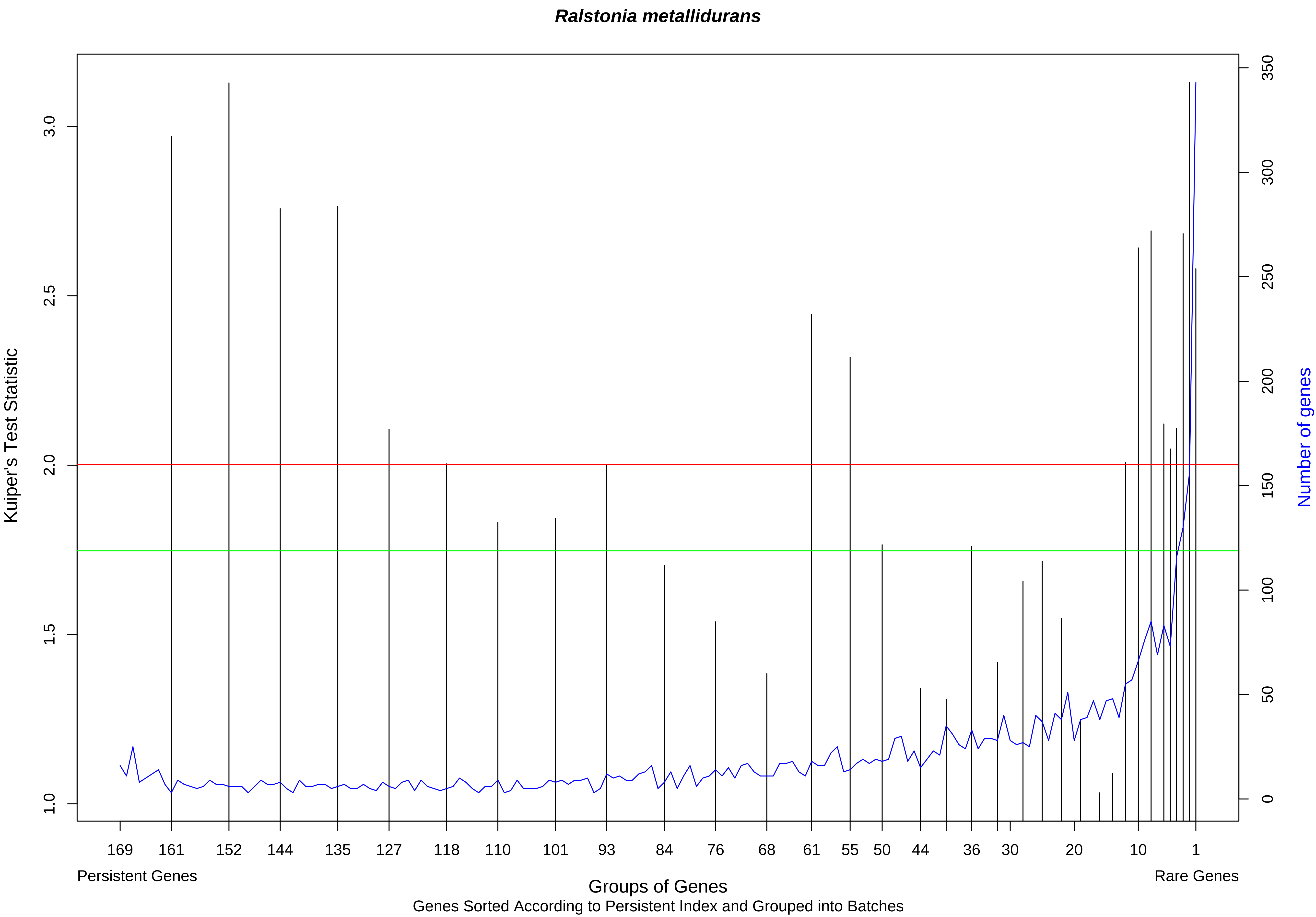

Ralstonia metallidurans
Kuiper's Test Statistic
Number of genes
1.0
1.5
2.0
2.5
3.0
0
50
100
150
200
250
300
350
169
161
152
144
135
127
118
110
101
93
84
76
68
61
55
50
44
36
30
20
10
1
Persistent Genes
Rare Genes
Groups of Genes
Genes Sorted According to Persistent Index and Grouped into Batches

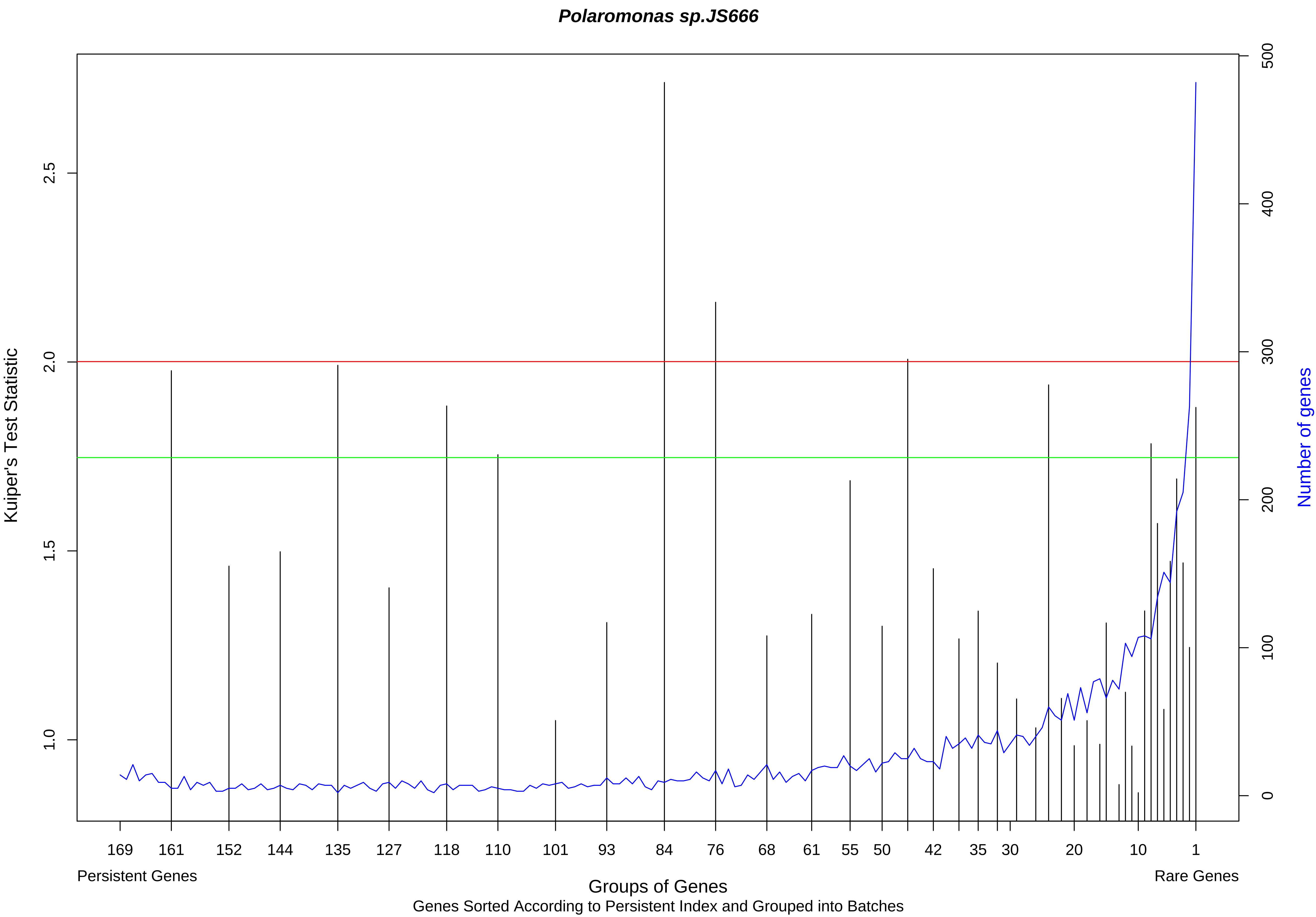

Polaromonas sp.JS666
Kuiper's Test Statistic
1.0
1.5
2.0
2.5
Number of genes
0
100
200
300
400
500
169
161
152
144
135
127
118
110
101
93
84
76
68
61
55
50
42
35
30
20
10
1
Persistent Genes
Rare Genes
Groups of Genes
Genes Sorted According to Persistent Index and Grouped into Batches

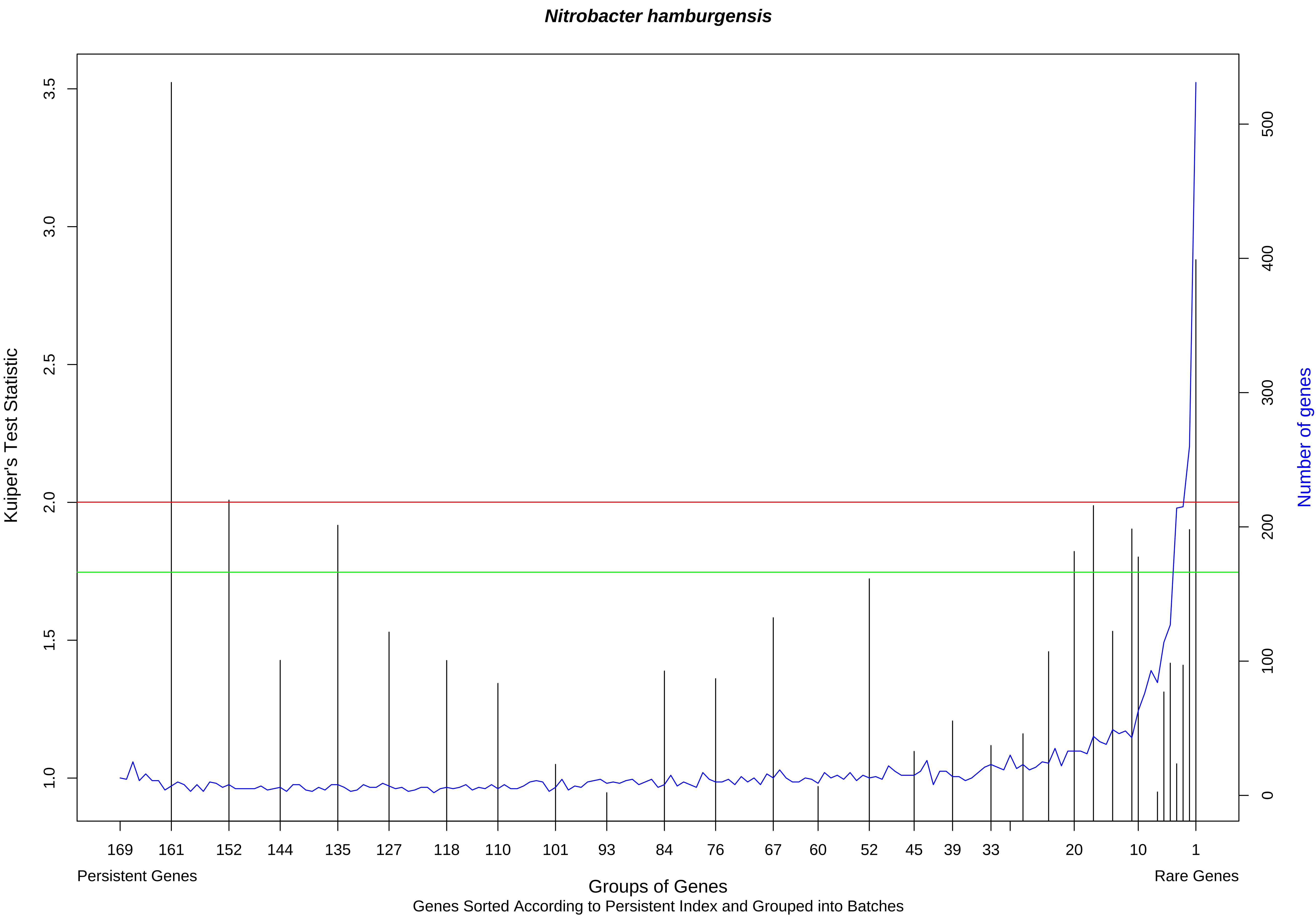

Nitrobacter hamburgensis
Kuiper's Test Statistic
Number of genes
1.0
1.5
2.0
2.5
3.0
3.5
0
100
200
300
400
500
169 161 152 144 135 127 118 110 101 93 84 76 67 60 52 45 39 33 20 10 1
Persistent Genes
Rare Genes
Groups of Genes
Genes Sorted According to Persistent Index and Grouped into Batches

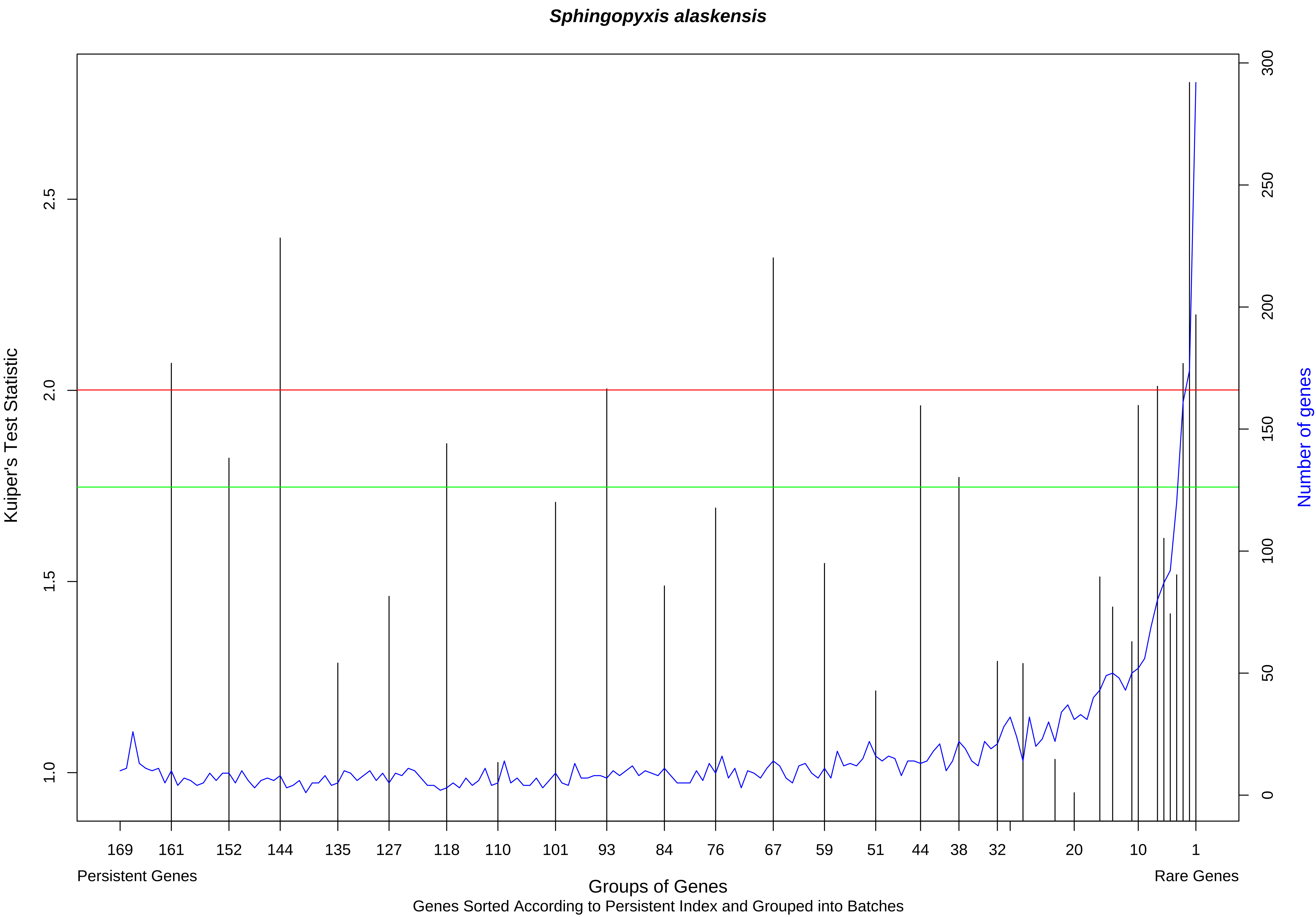

Sphingopyxis alaskensis
Kuiper's Test Statistic
1.0
1.5
2.0
2.5
Number of genes
0
50
100
150
200
250
300
169
161
152
144
135
127
118
110
101
93
84
76
67
59
51
44
38
32
20
10
1
Persistent Genes
Rare Genes
Groups of Genes
Genes Sorted According to Persistent Index and Grouped into Batches

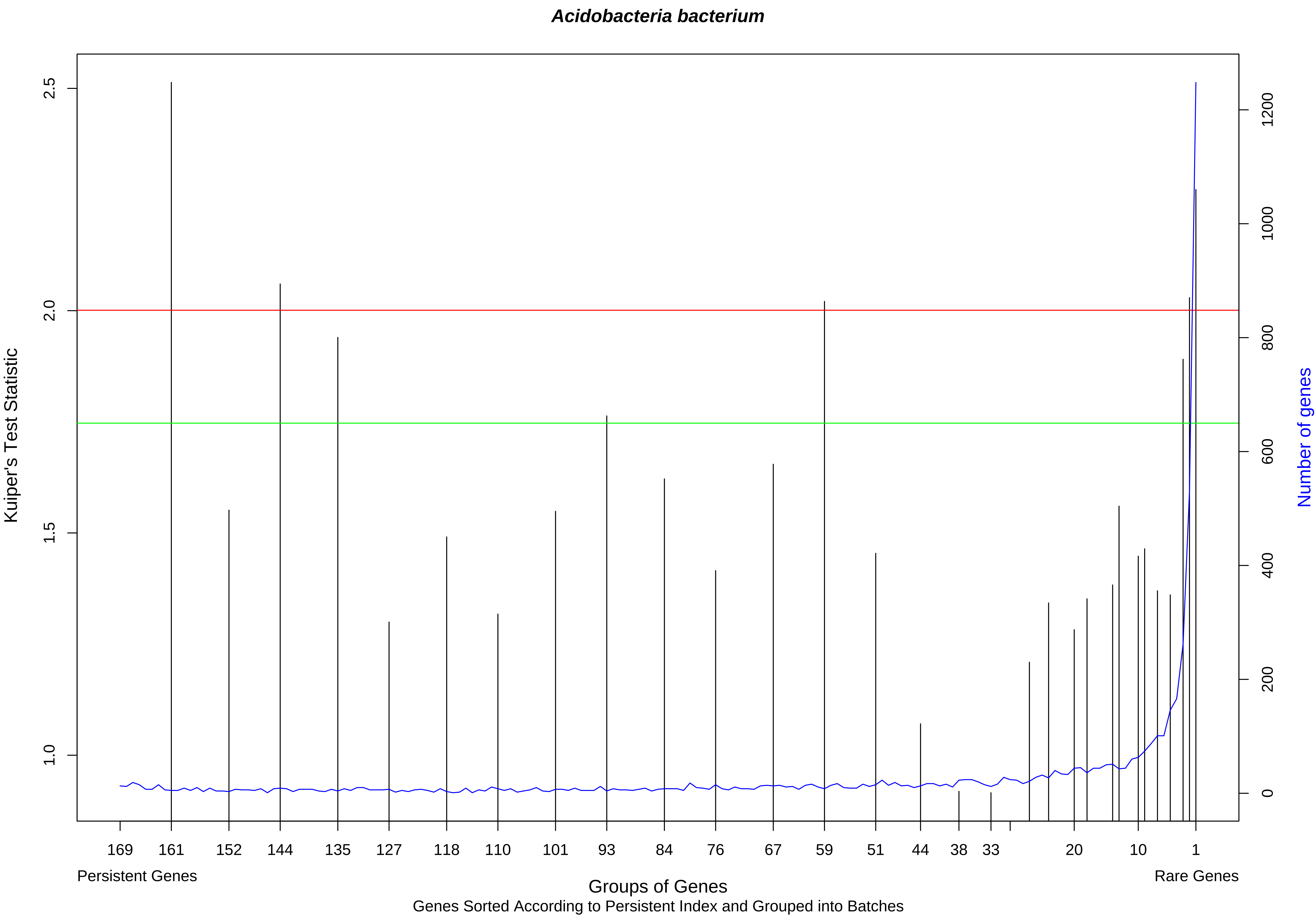
Acidobacteria bacterium
Kuiper's Test Statistic
Number of genes
2.5
2.0
1.5
1.0
1200
1000
800
600
400
200
0
169
161
152
144
135
127
118
110
101
93
84
76
67
59
51
44
38
33
20
10
1
Persistent Genes
Rare Genes
Groups of Genes
Genes Sorted According to Persistent Index and Grouped into Batches

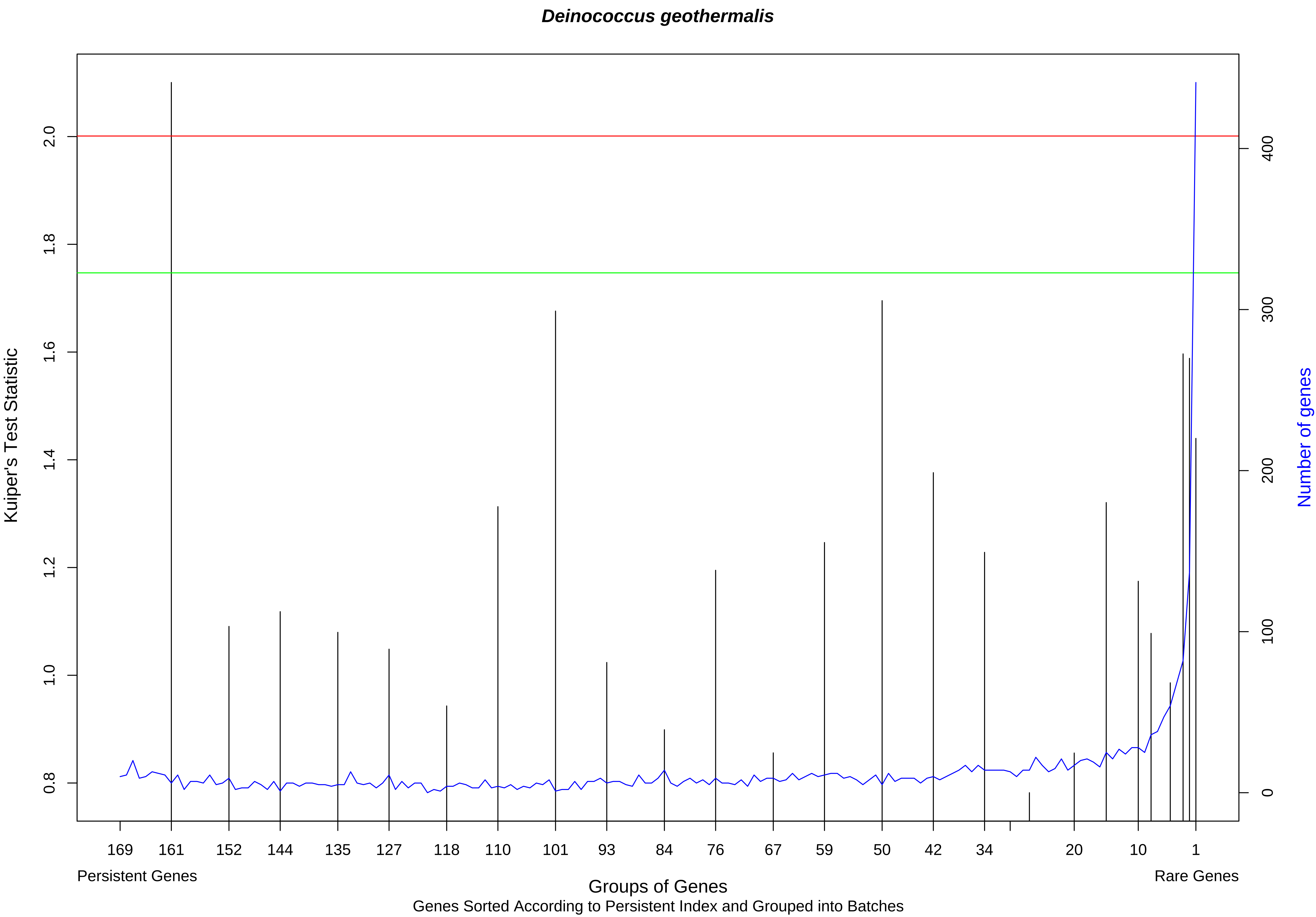
Deinococcus geothermalis
Kuiper's Test Statistic
Number of genes
0.8
1.0
1.2
1.4
1.6
1.8
2.0
0
100
200
300
400
169
161
152
144
135
127
118
110
101
93
84
76
67
59
50
42
34
20
10
1
Persistent Genes
Rare Genes
Groups of Genes
Genes Sorted According to Persistent Index and Grouped into Batches

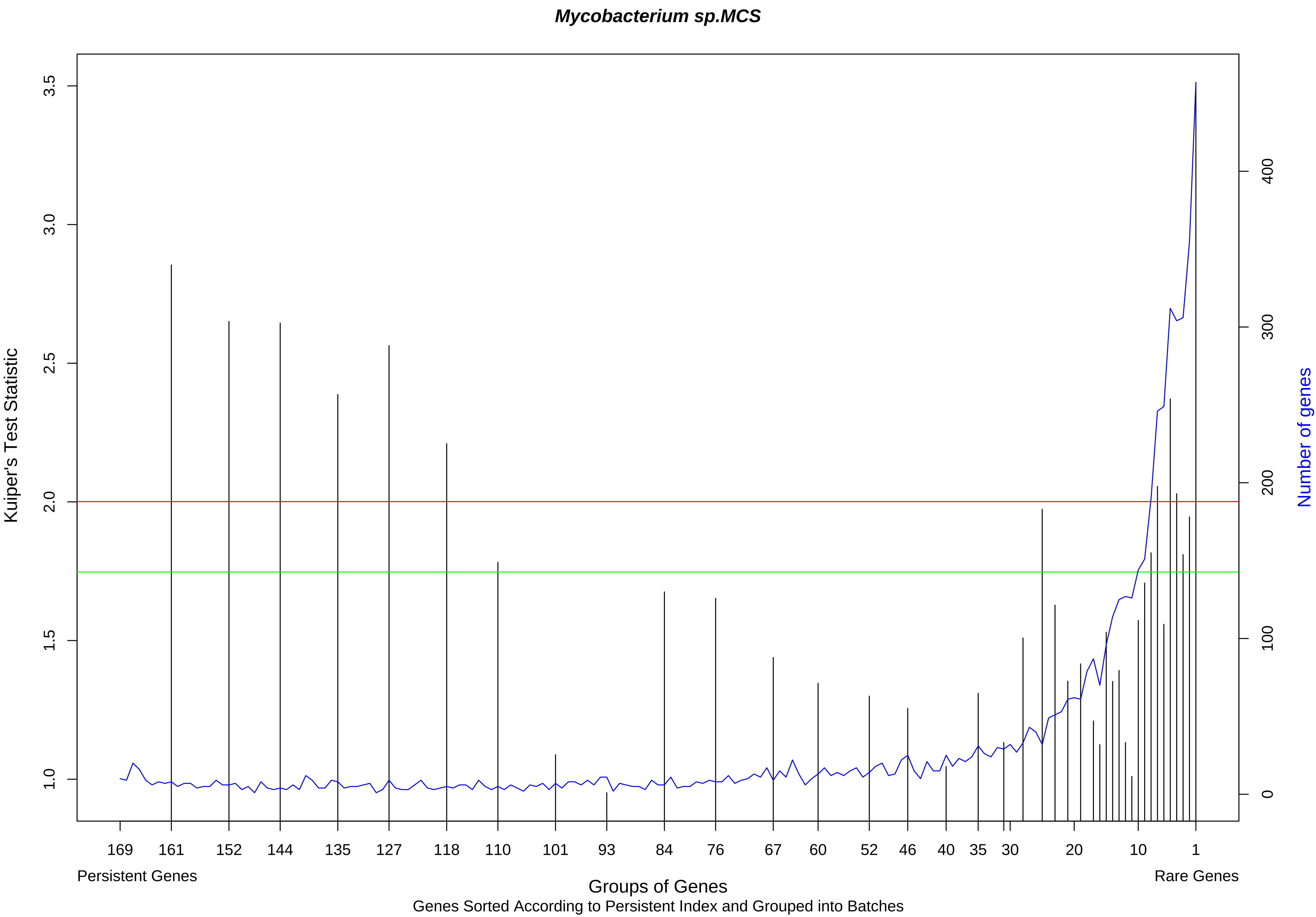

Mycobacterium sp.MCS
Kuiper's Test Statistic
Number of genes
3.5
3.0
2.5
2.0
1.5
1.0
400
300
200
100
0
169
161
152
144
135
127
118
110
101
93
84
76
67
60
52
46
40
35
30
20
10
1
Persistent Genes
Rare Genes
Groups of Genes
Genes Sorted According to Persistent Index and Grouped into Batches

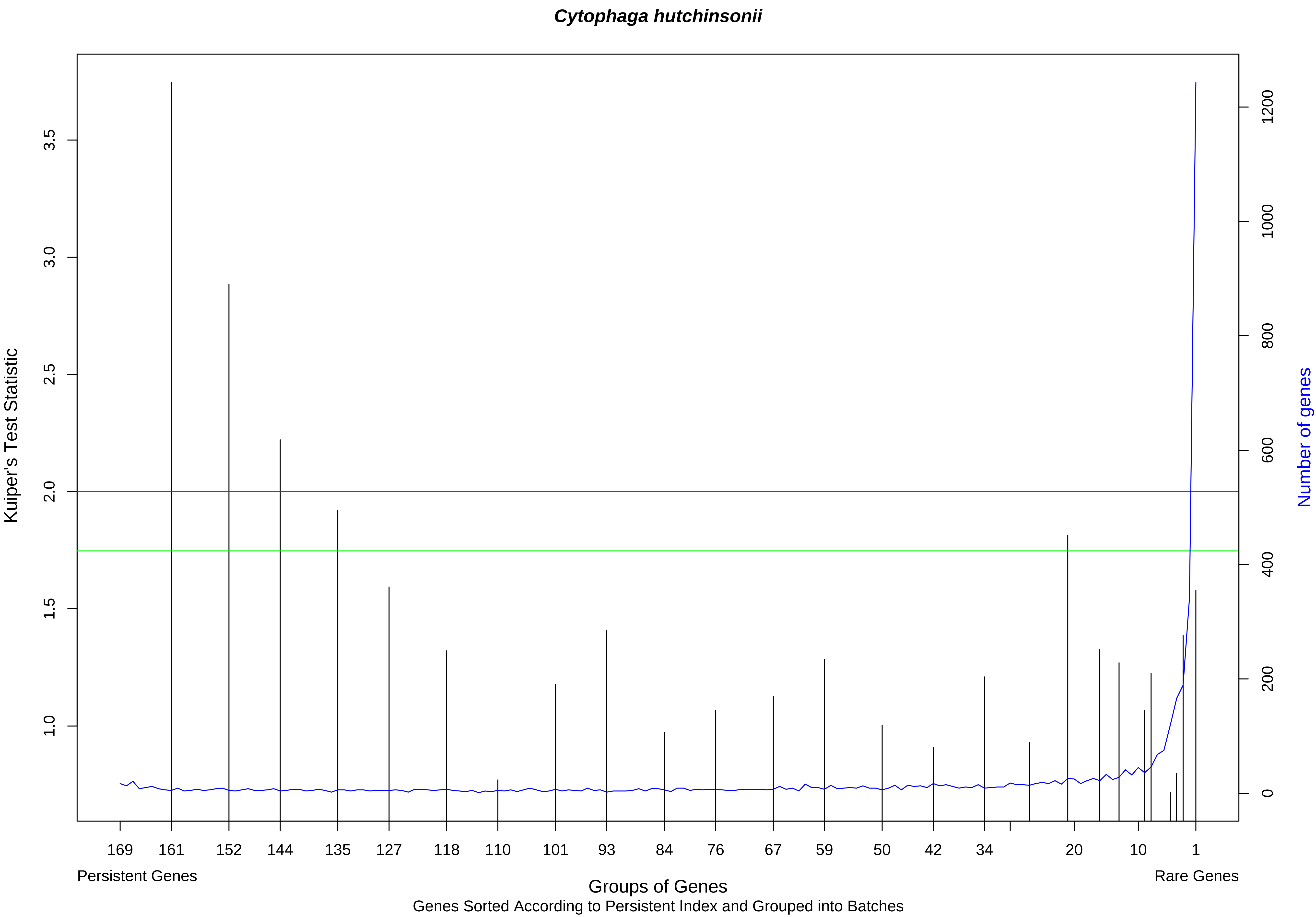

Cytophaga hutchinsonii
Kuiper's Test Statistic
Number of genes
1.0
1.5
2.0
2.5
3.0
3.5
0
200
400
600
800
1000
1200
169
161
152
144
135
127
118
110
101
93
84
76
67
59
50
42
34
20
10
1
Persistent Genes
Rare Genes
Groups of Genes
Genes Sorted According to Persistent Index and Grouped into Batches

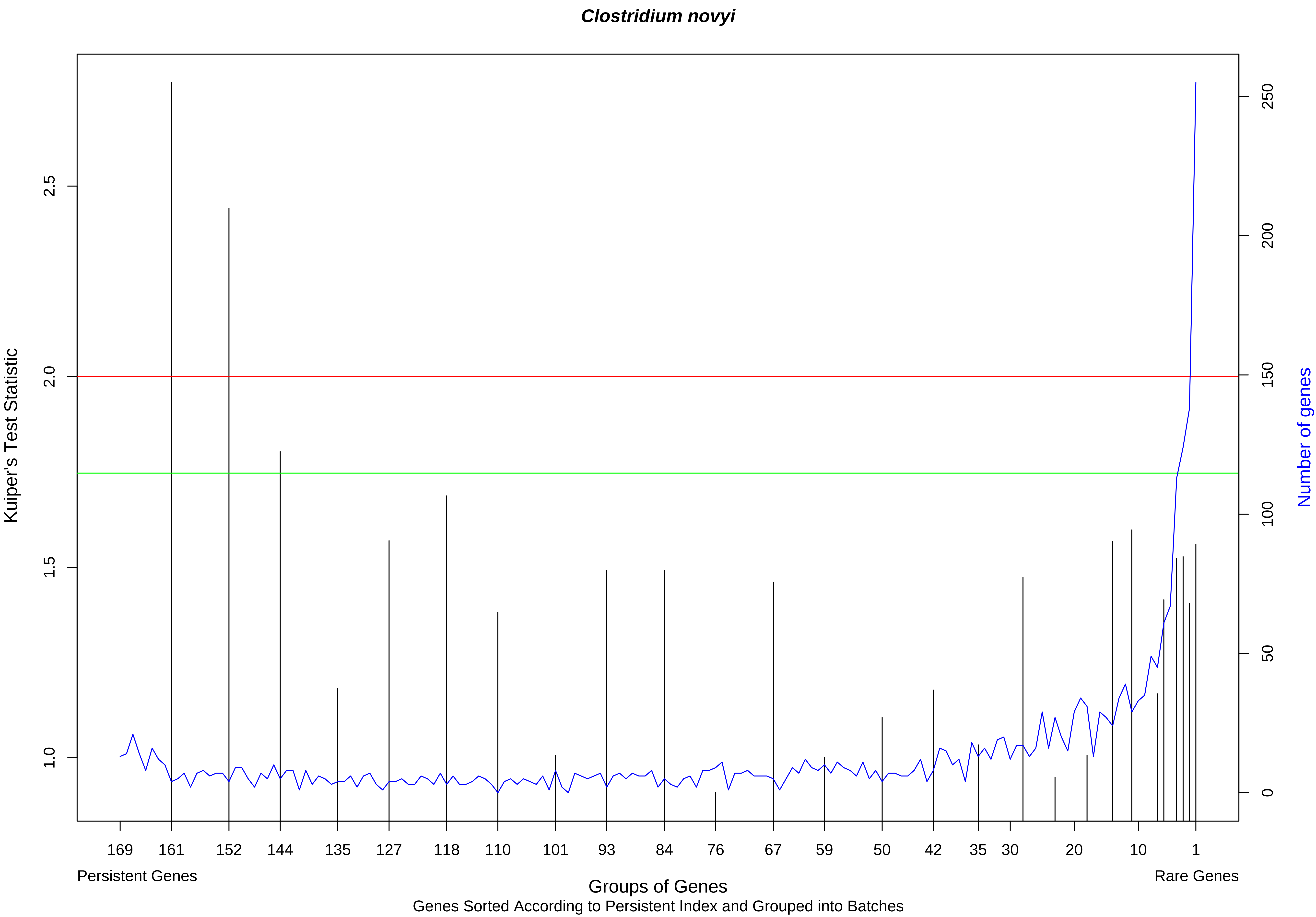

Clostridium novyi
Kuiper's Test Statistic
1.0
1.5
2.0
2.5
Number of genes
0
50
100
150
200
250
169
161
152
144
135
127
118
110
101
93
84
76
67
59
50
42
35
30
20
10
1
Persistent Genes
Rare Genes
Groups of Genes
Genes Sorted According to Persistent Index and Grouped into Batches

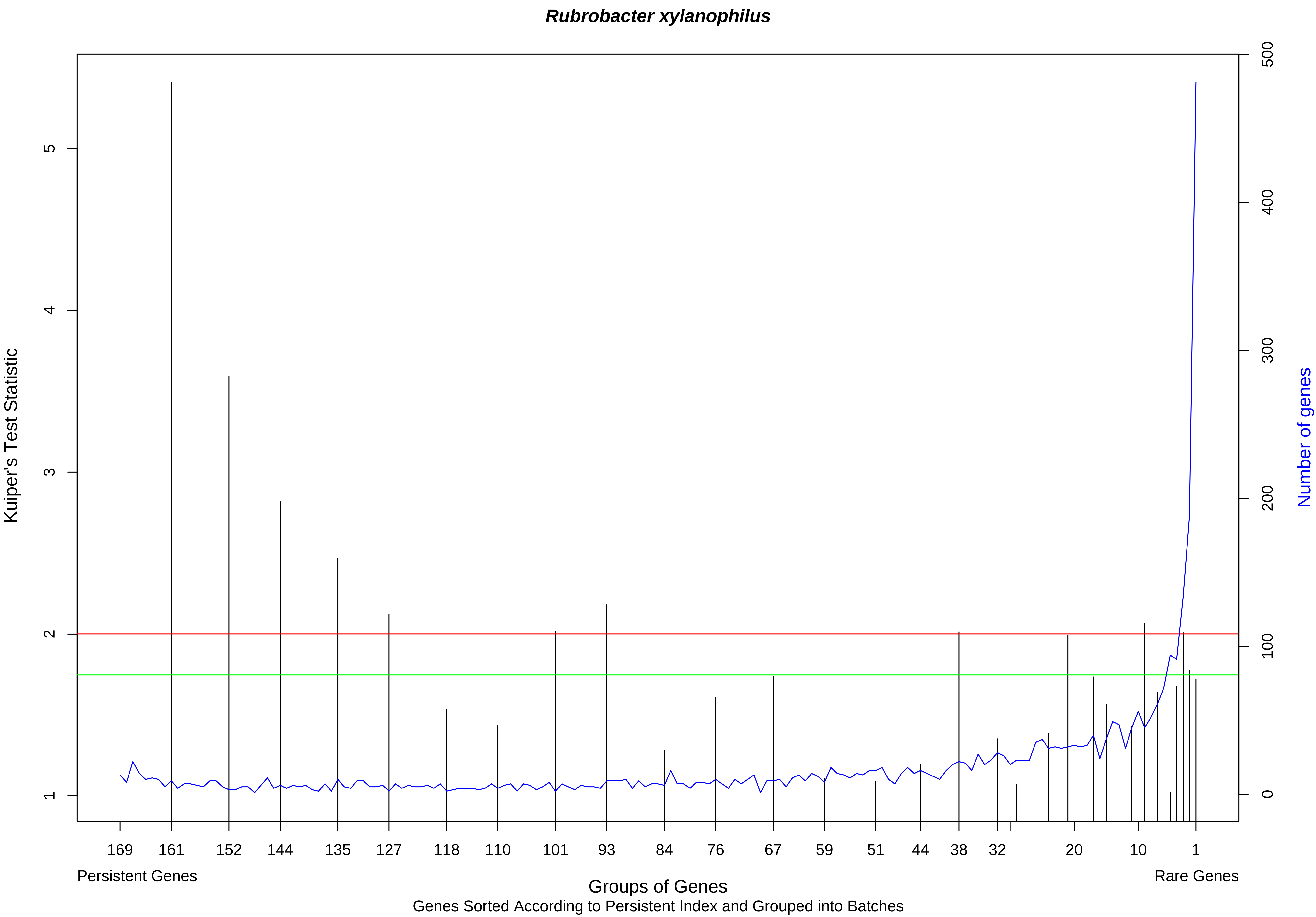

Rubrobacter xylanophilus
Kuiper's Test Statistic
1
2
3
4
5
Number of genes
0
100
200
300
400
500
169
161
152
144
135
127
118
110
101
93
84
76
67
59
51
44
38
32
20
10
1
Persistent Genes
Rare Genes
Groups of Genes
Genes Sorted According to Persistent Index and Grouped into Batches

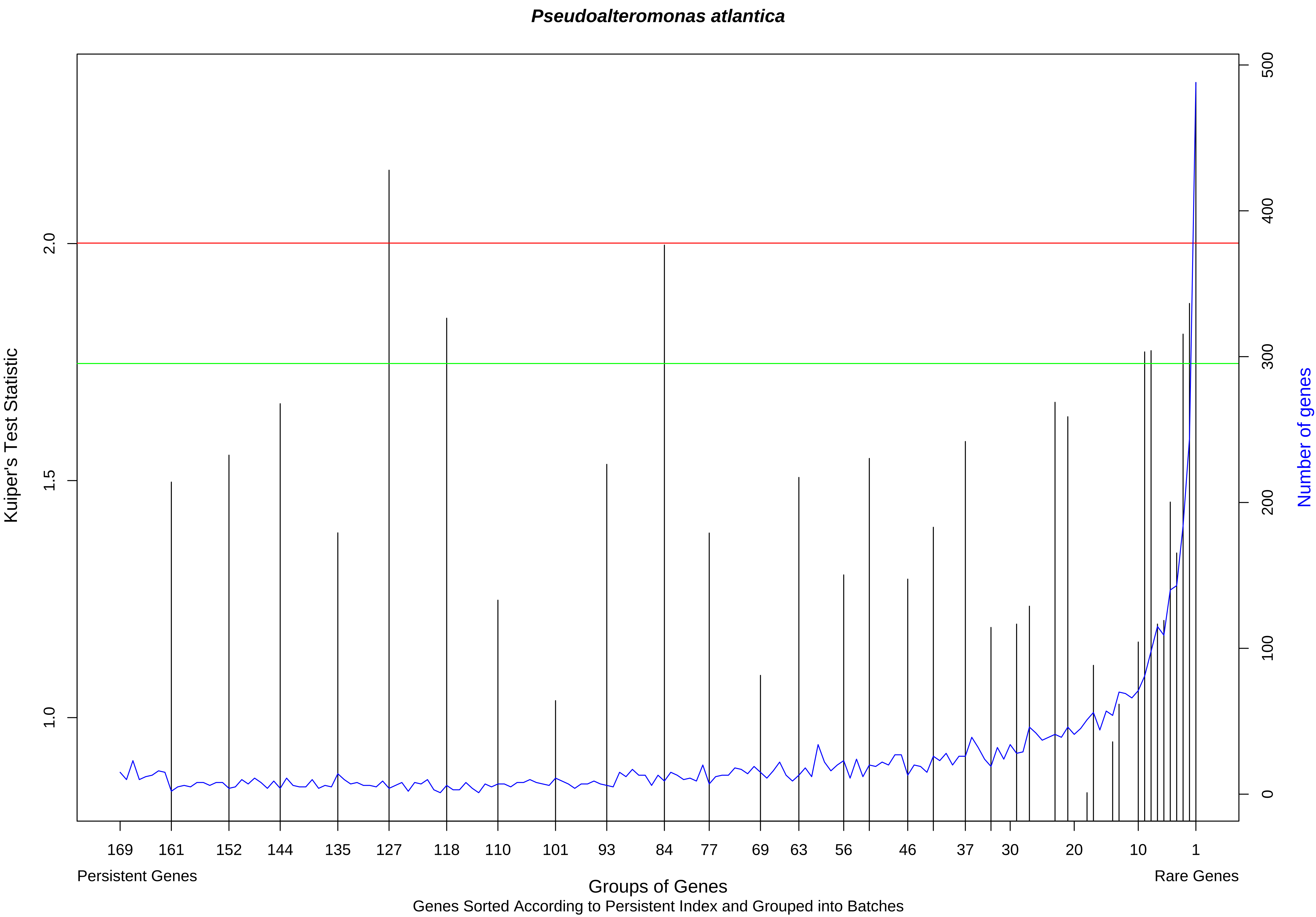

Pseudoalteromonas atlantica
Kuiper's Test Statistic
Number of genes
2.0
1.5
1.0
500
400
300
200
100
0
169
161
152
144
135
127
118
110
101
93
84
77
69
63
56
46
37
30
20
10
1
Persistent Genes
Rare Genes
Groups of Genes
Genes Sorted According to Persistent Index and Grouped into Batches

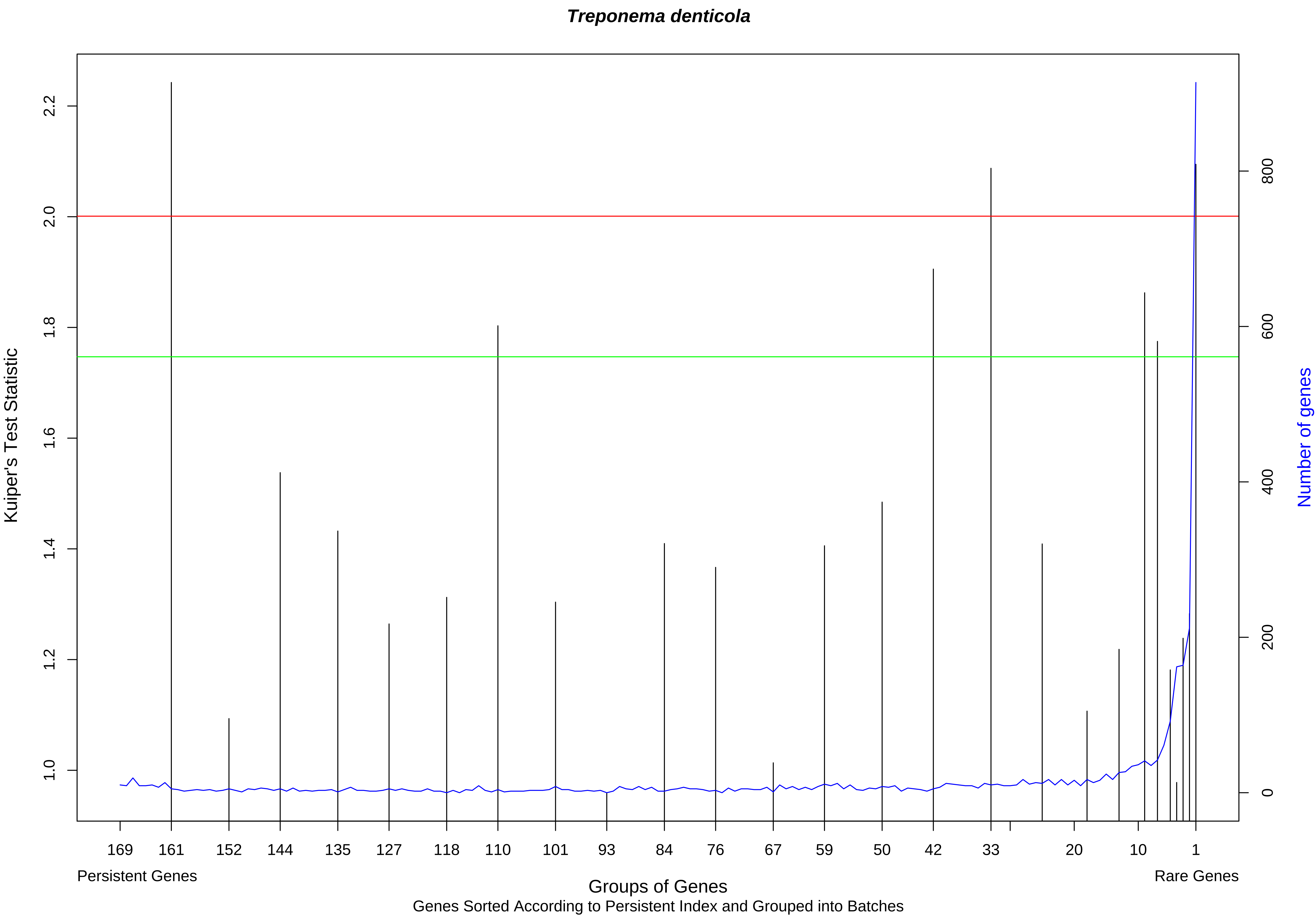

Treponema denticola
Kuiper's Test Statistic
2.2
2.0
1.8
1.6
1.4
1.2
1.0
Number of genes
800
600
400
200
0
169
161
152
144
135
127
118
110
101
93
84
76
67
59
50
42
33
20
10
1
Persistent Genes
Rare Genes
Groups of Genes
Genes Sorted According to Persistent Index and Grouped into Batches

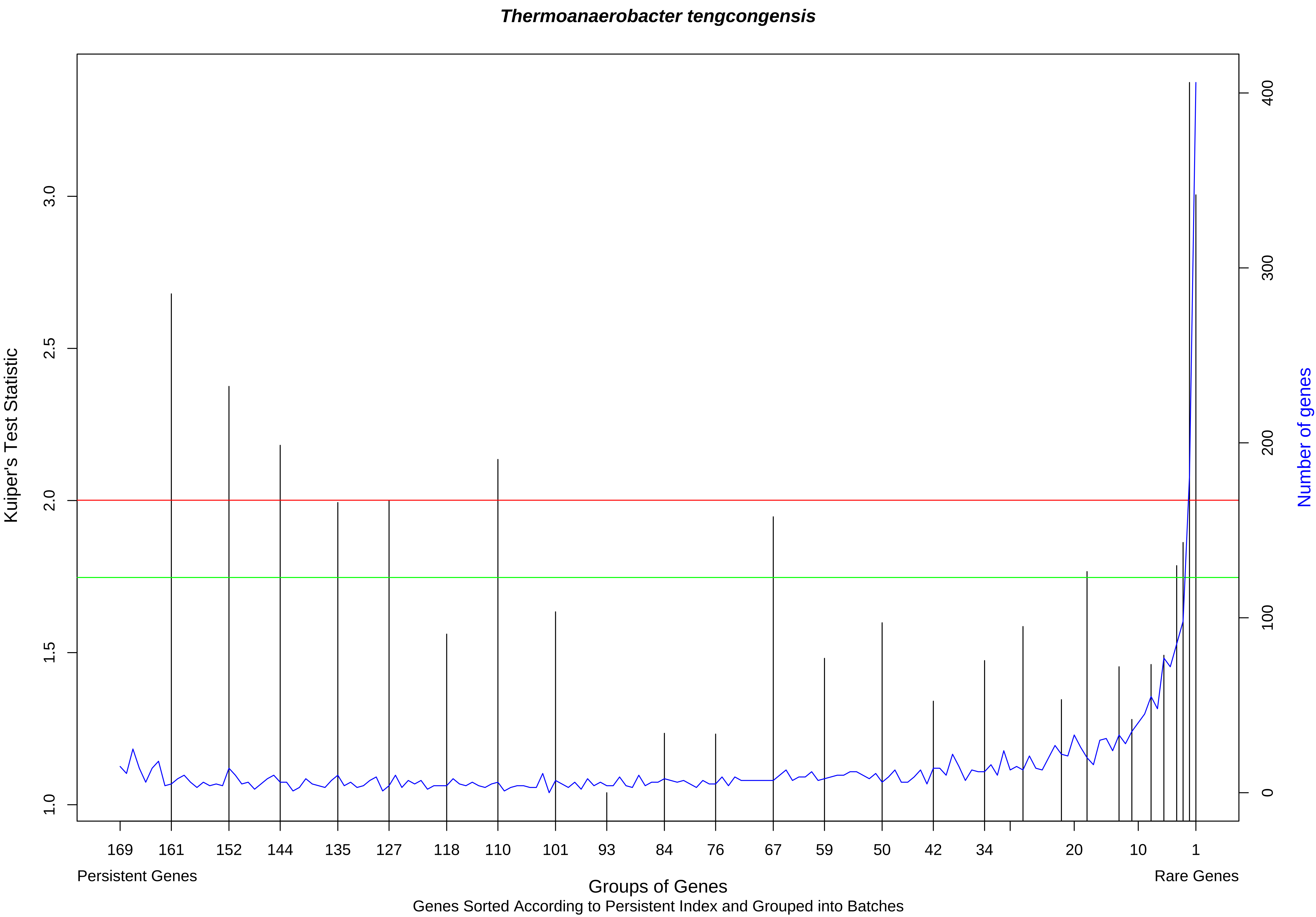

Thermoanaerobacter tengcongensis
Kuiper's Test Statistic
1.0
1.5
2.0
2.5
3.0
Number of genes
0
100
200
300
400
169
161
152
144
135
127
118
110
101
93
84
76
67
59
50
42
34
20
10
1
Persistent Genes
Rare Genes
Groups of Genes
Genes Sorted According to Persistent Index and Grouped into Batches

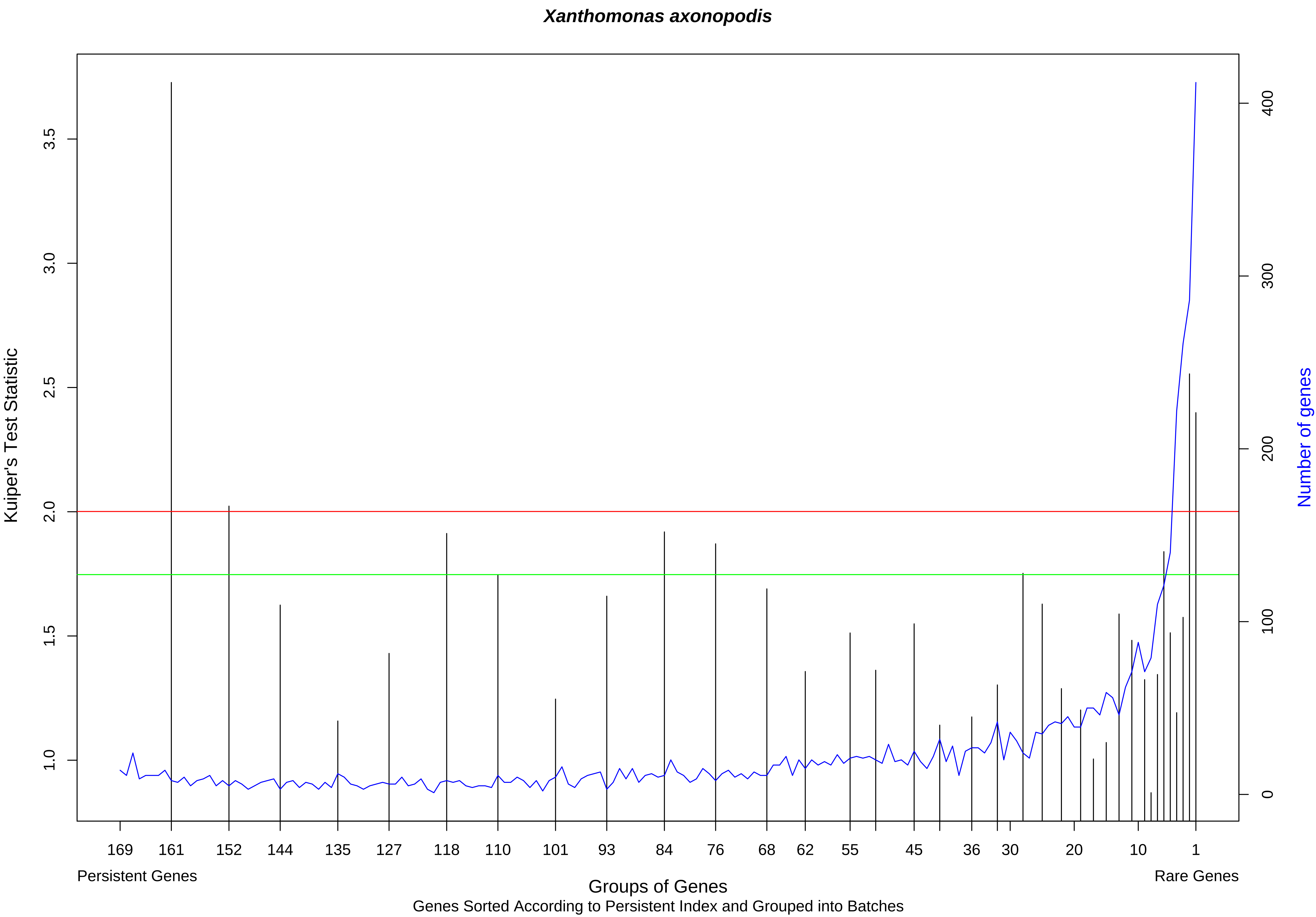

Xanthomonas axonopodis
Kuiper's Test Statistic
1.0
1.5
2.0
2.5
3.0
3.5
Number of genes
0
100
200
300
400
169
161
152
144
135
127
118
110
101
93
84
76
68
62
55
45
36
30
20
10
1
Persistent Genes
Rare Genes
Groups of Genes
Genes Sorted According to Persistent Index and Grouped into Batches

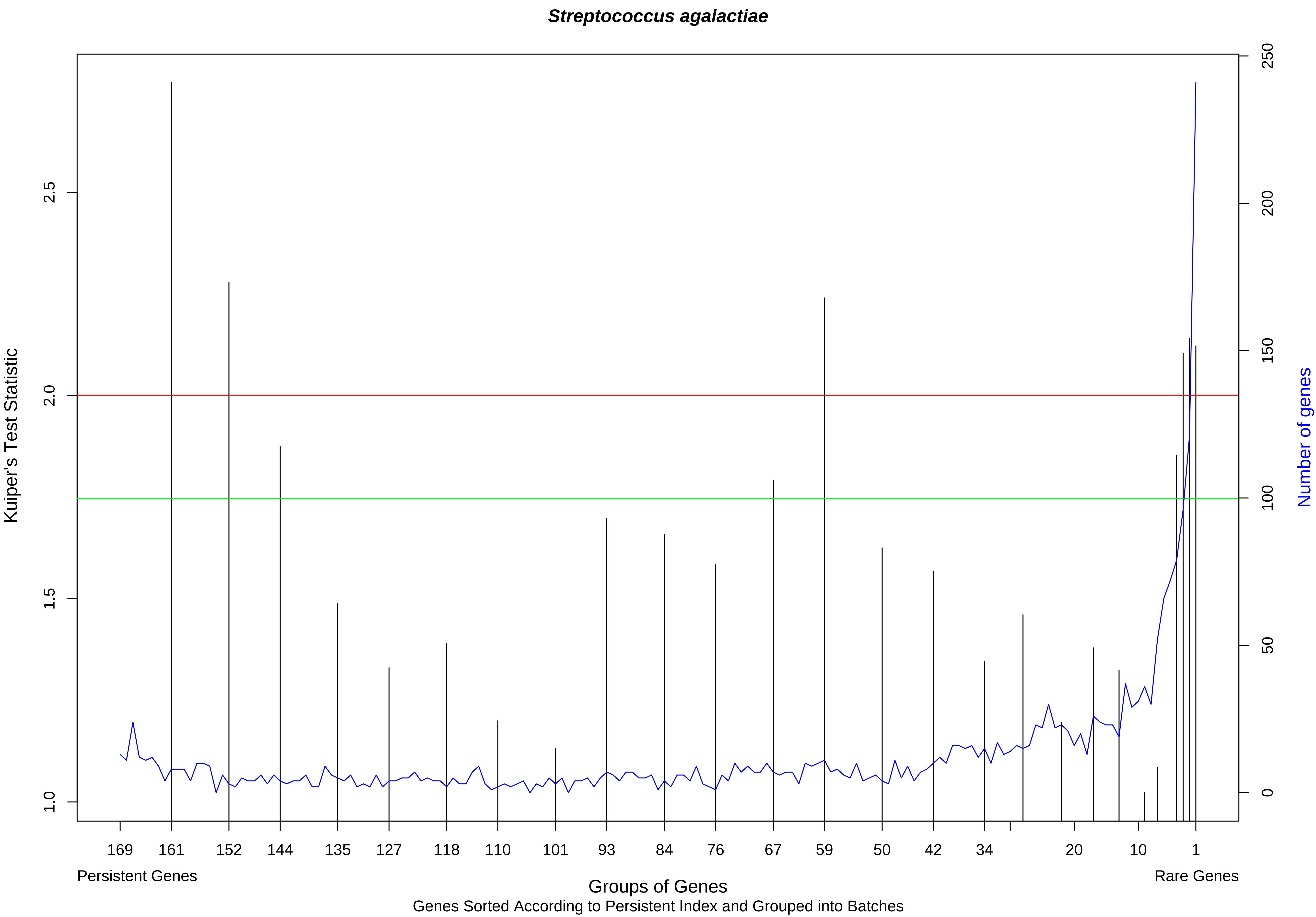

Streptococcus agalactiae
Kuiper's Test Statistic
Number of genes
1.0
1.5
2.0
2.5
0
50
100
150
200
250
169
161
152
144
135
127
118
110
101
93
84
76
67
59
50
42
34
20
10
1
Persistent Genes
Rare Genes
Groups of Genes
Genes Sorted According to Persistent Index and Grouped into Batches

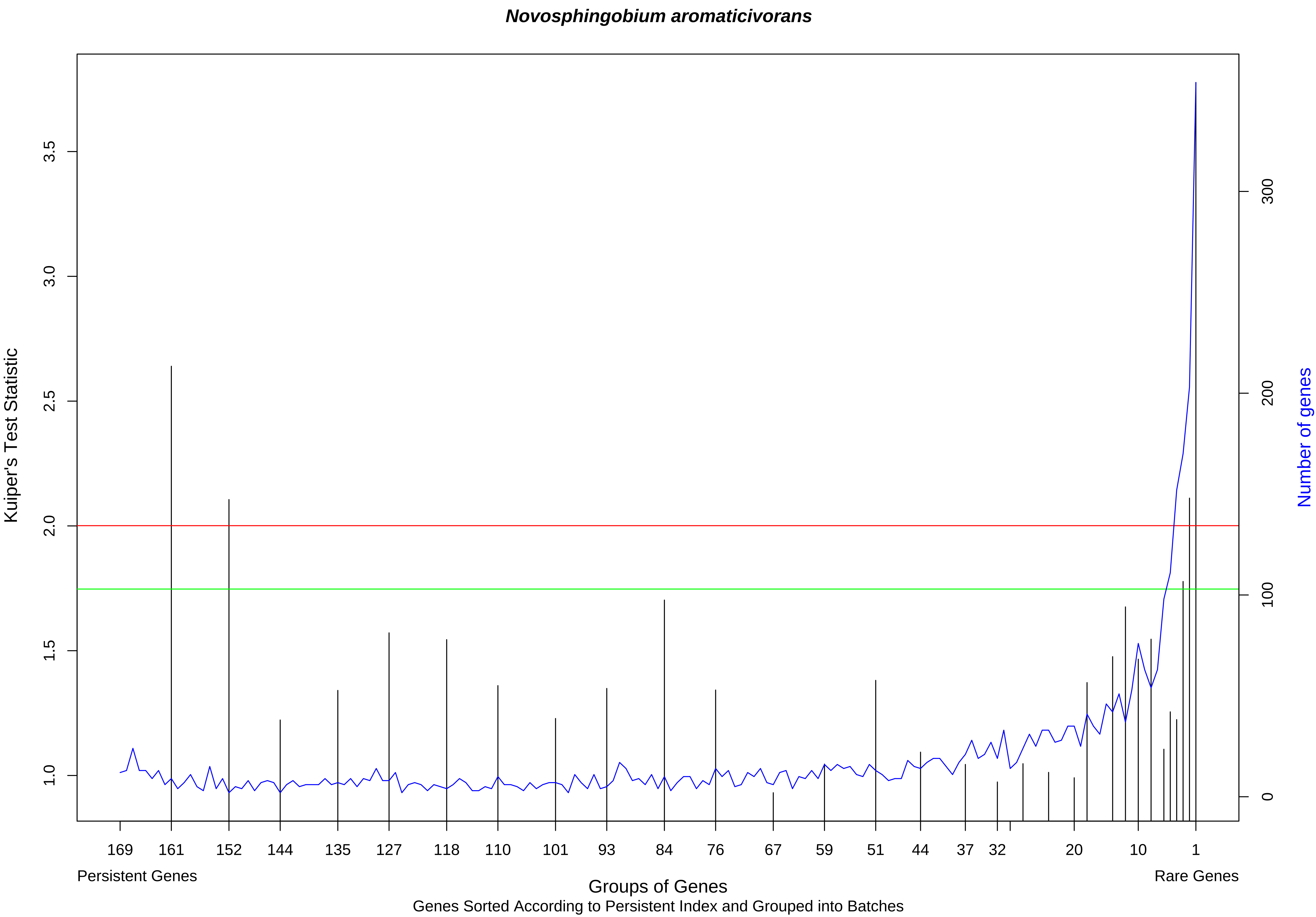

Novosphingobium aromaticivorans
Kuiper's Test Statistic
1.0
1.5
2.0
2.5
3.0
3.5
Number of genes
0
100
200
300
169
161
152
144
135
127
118
110
101
93
84
76
67
59
51
44
37
32
20
10
1
Persistent Genes
Rare Genes
Groups of Genes
Genes Sorted According to Persistent Index and Grouped into Batches

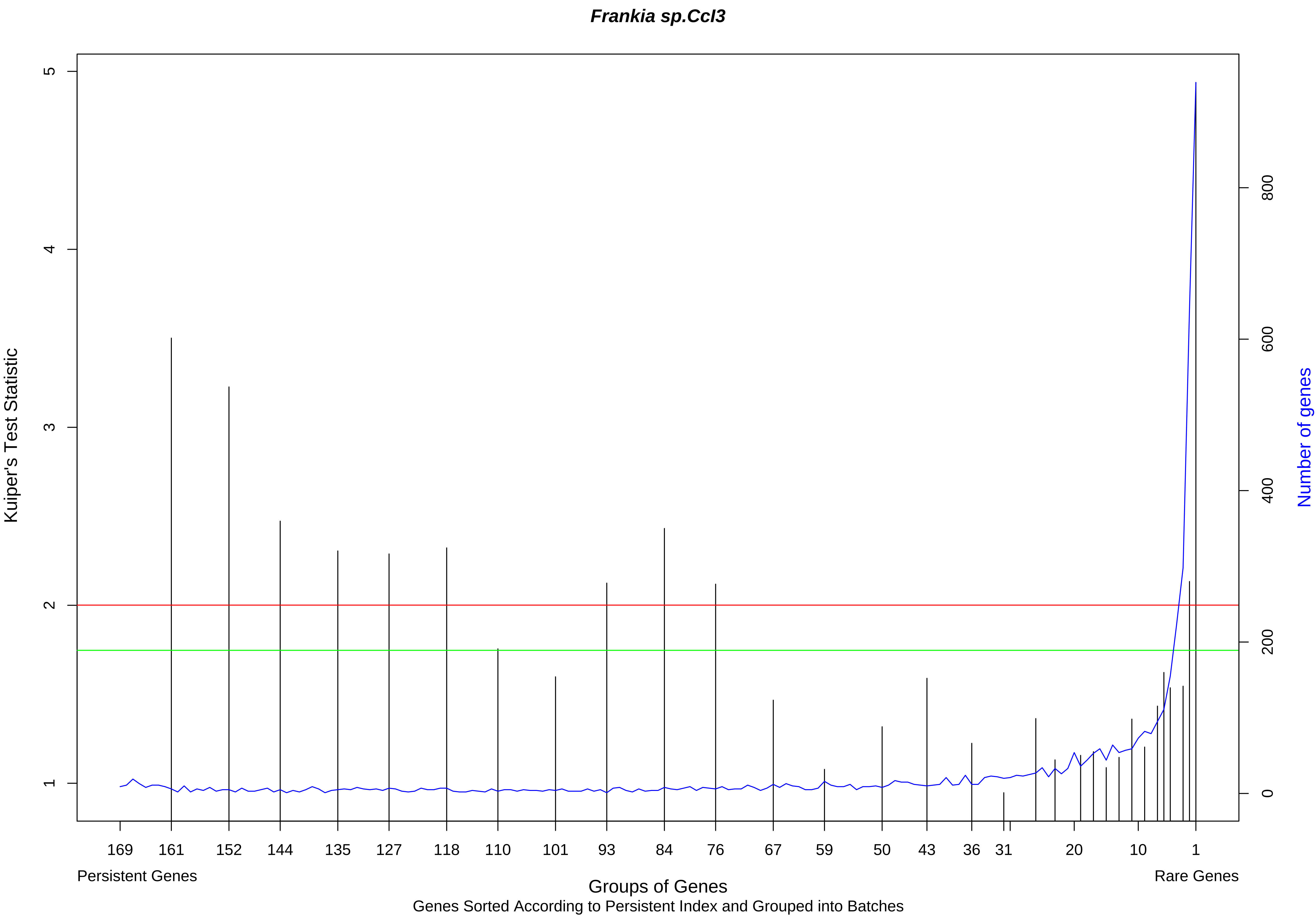

Frankia sp.CcI3
Kuiper's Test Statistic
Number of genes
5
4
3
2
1
800
600
400
200
0
169
161
152
144
135
127
118
110
101
93
84
76
67
59
50
43
36
31
20
10
1
Persistent Genes
Rare Genes
Groups of Genes
Genes Sorted According to Persistent Index and Grouped into Batches

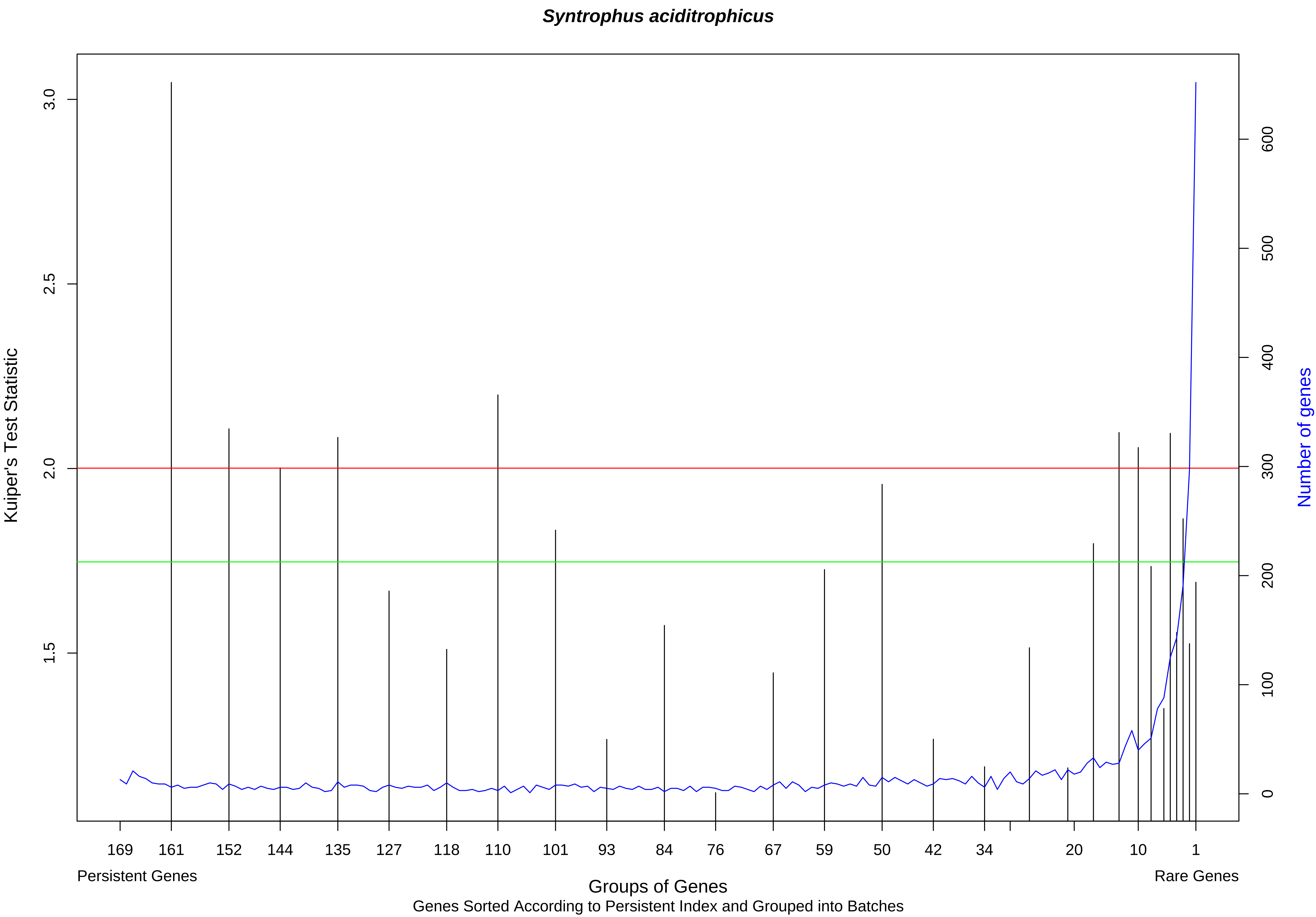

Syntrophus aciditrophicus
Kuiper's Test Statistic
3.0
2.5
2.0
1.5
Number of genes
600
500
400
300
200
100
0
169
161
152
144
135
127
118
110
101
93
84
76
67
59
50
42
34
20
10
1
Persistent Genes
Rare Genes
Groups of Genes
Genes Sorted According to Persistent Index and Grouped into Batches

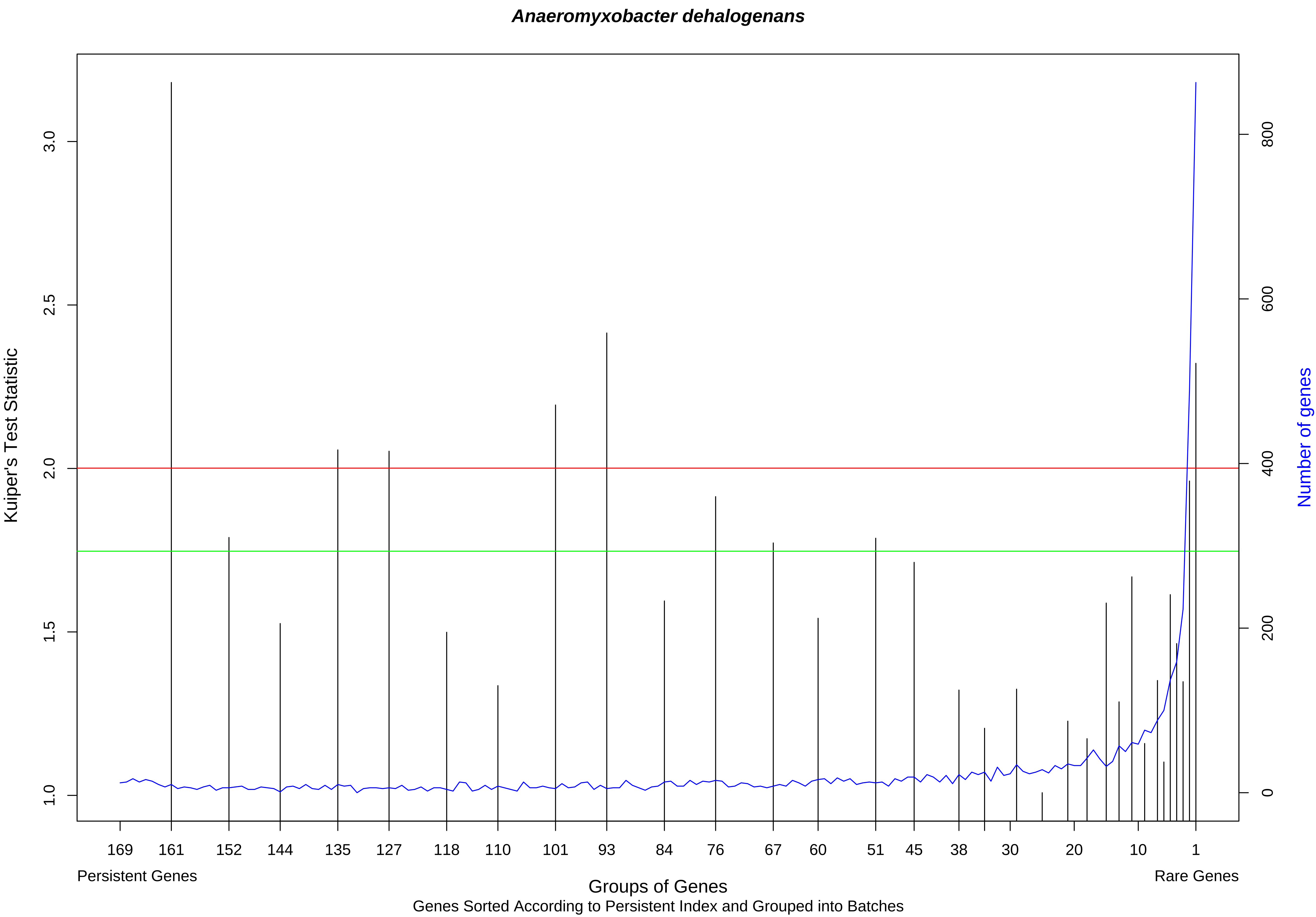

Anaeromyxobacter dehalogenans
Kuiper's Test Statistic
Number of genes
3.0
2.5
2.0
1.5
1.0
800
600
400
200
0
169
161
152
144
135
127
118
110
101
93
84
76
67
60
51
45
38
30
20
10
1
Persistent Genes
Rare Genes
Groups of Genes
Genes Sorted According to Persistent Index and Grouped into Batches

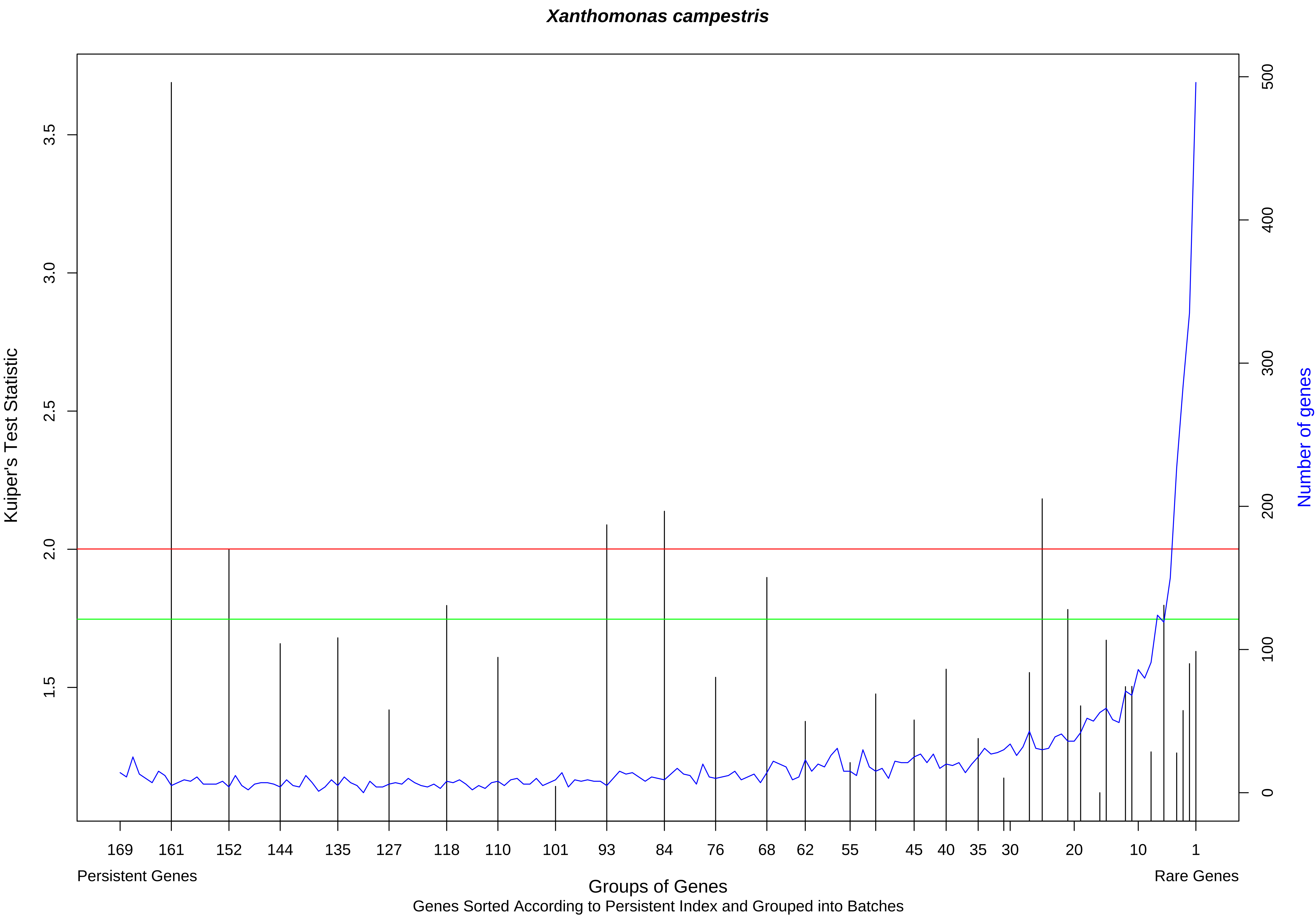

Xanthomonas campestris
Kuiper's Test Statistic
3.5
3.0
2.5
2.0
1.5
Number of genes
500
400
300
200
100
0
169
161
152
144
135
127
118
110
101
93
84
76
68
62
55
45
40
35
30
20
10
1
Persistent Genes
Rare Genes
Groups of Genes
Genes Sorted According to Persistent Index and Grouped into Batches

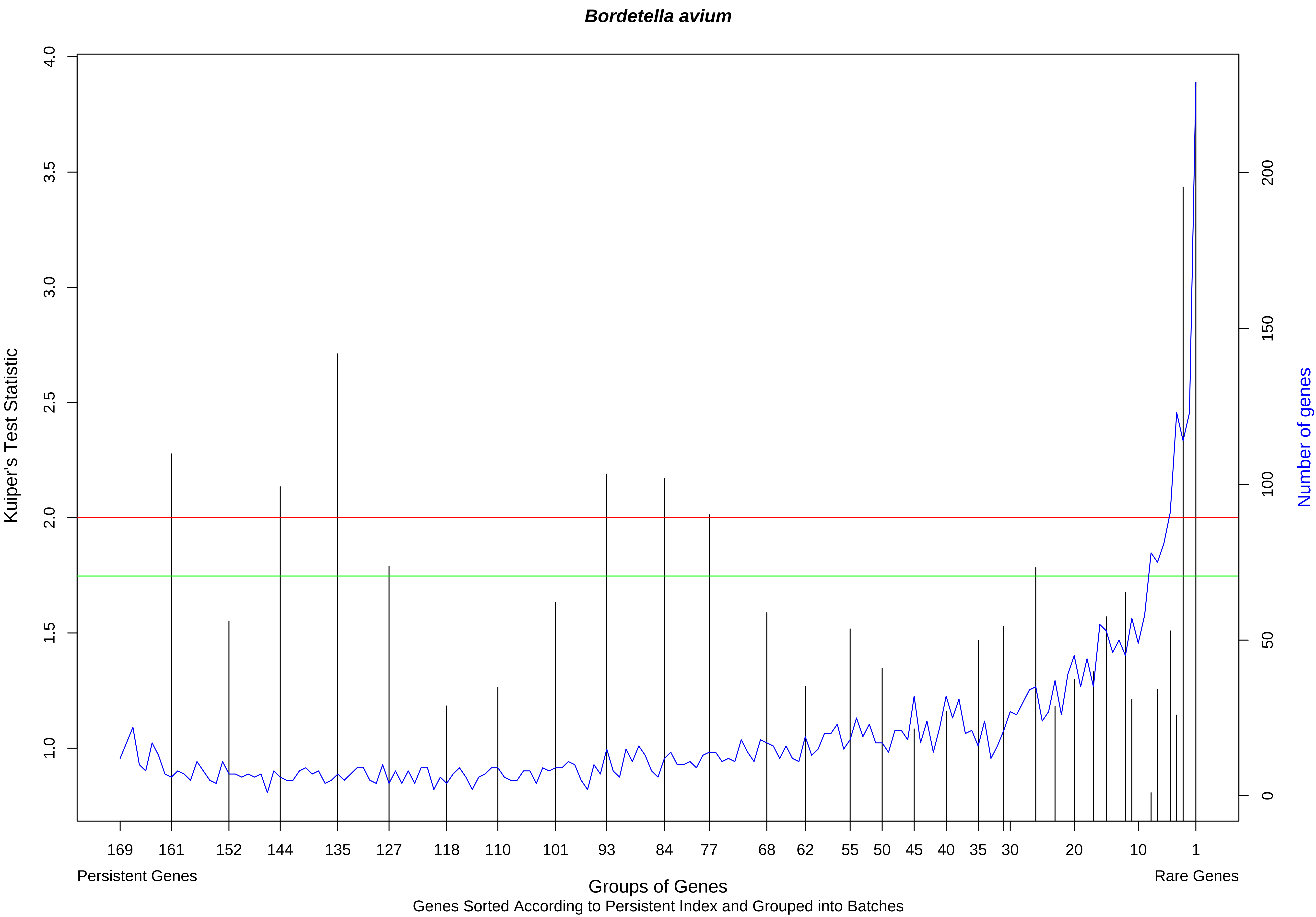
Bordetella avium
Kuiper's Test Statistic
Number of genes
4.0
3.5
3.0
2.5
2.0
1.5
1.0
200
150
100
50
0
169
161
152
144
135
127
118
110
101
93
84
77
68
62
55
50
45
40
35
30
20
10
1
Persistent Genes
Rare Genes
Groups of Genes
Genes Sorted According to Persistent Index and Grouped into Batches

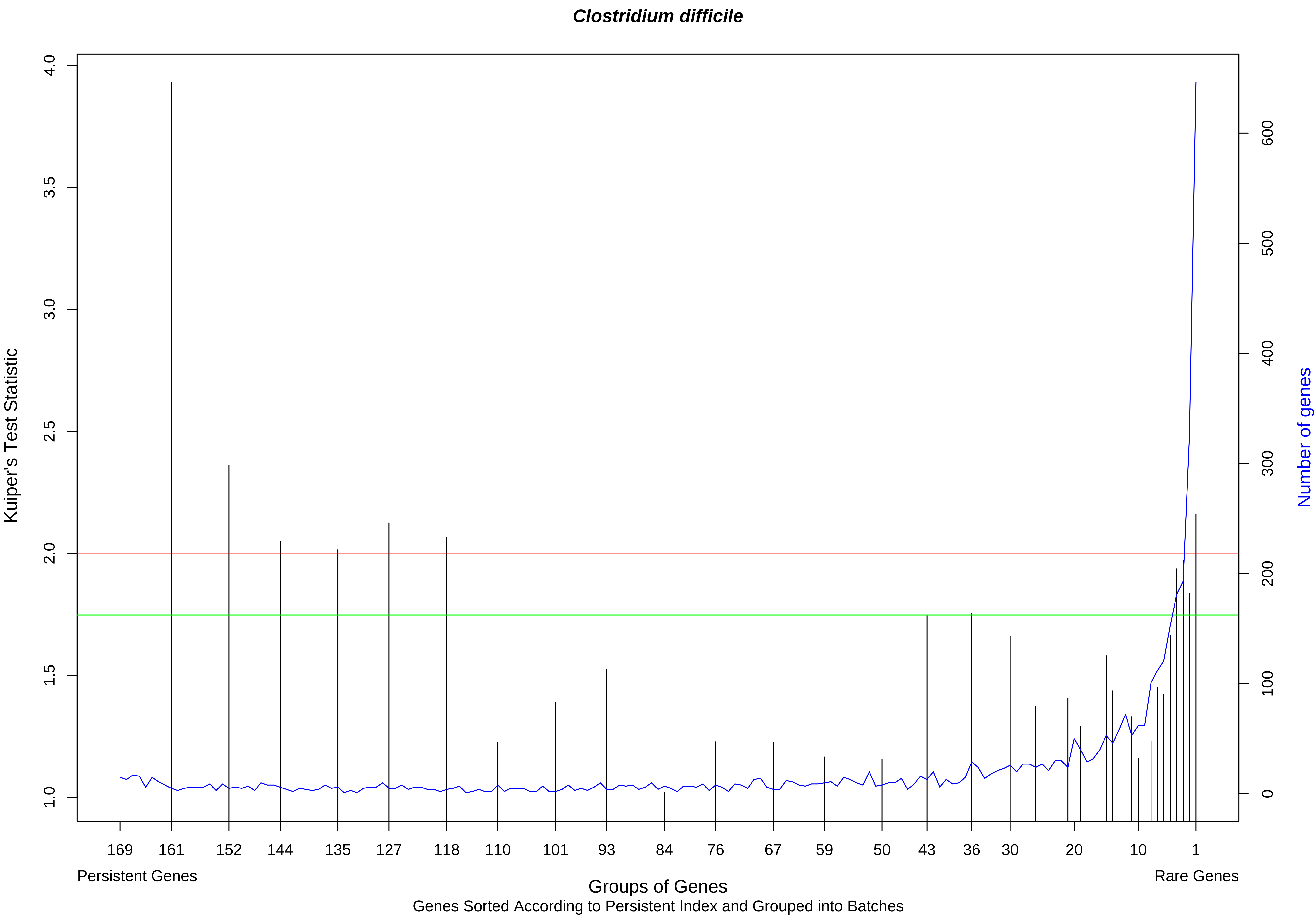

Clostridium difficile
Kuiper's Test Statistic
Number of genes
1.0
1.5
2.0
2.5
3.0
3.5
4.0
0
100
200
300
400
500
600
169
161
152
144
135
127
118
110
101
93
84
76
67
59
50
43
36
30
20
10
1
Persistent Genes
Rare Genes
Groups of Genes
Genes Sorted According to Persistent Index and Grouped into Batches

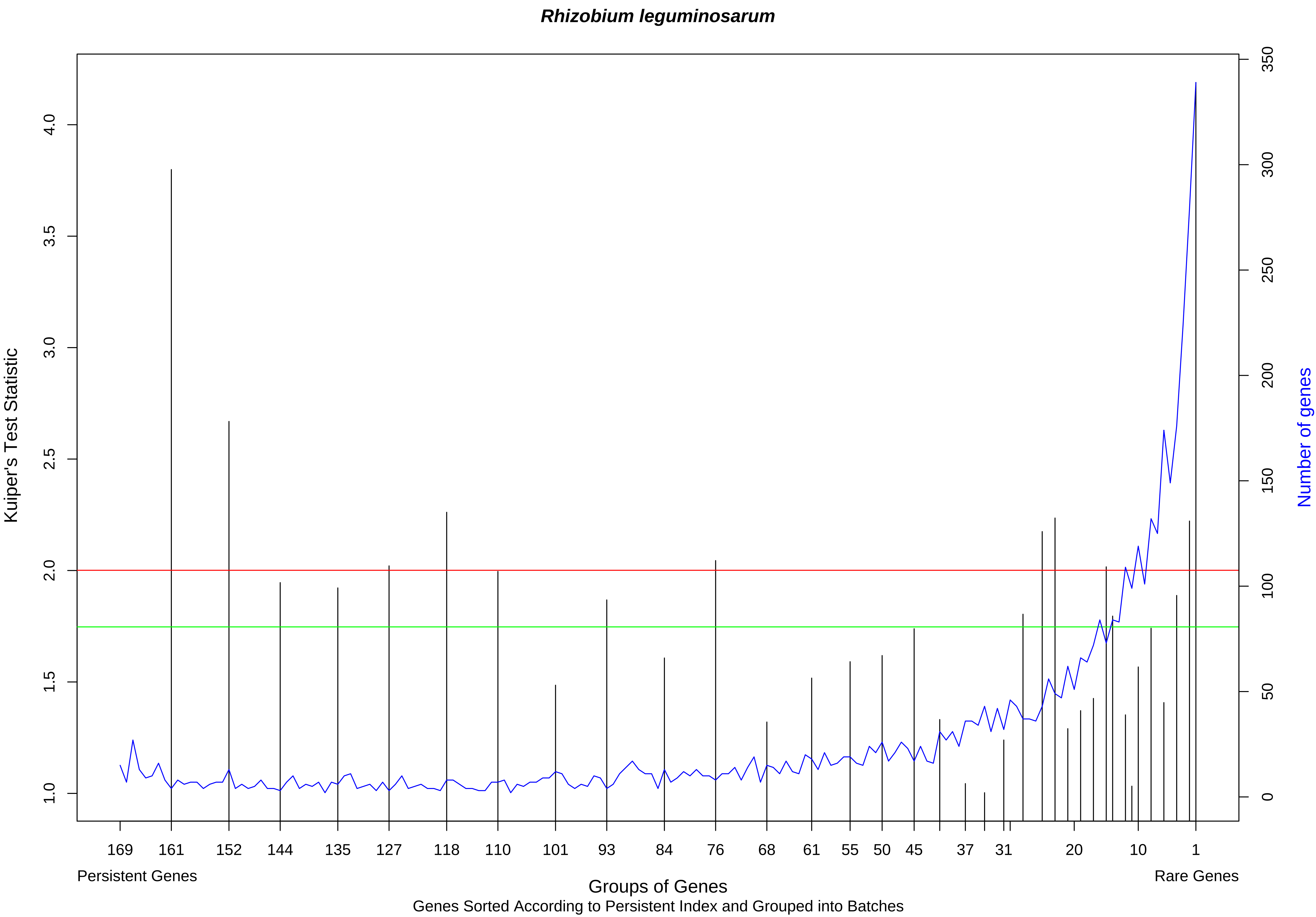
Rhizobium leguminosarum
Kuiper's Test Statistic
Number of genes
Groups of Genes
Genes Sorted According to Persistent Index and Grouped into Batches
Persistent Genes
Rare Genes
169
161
152
144
135
127
118
110
101
93
84
76
68
61
55
50
45
37
31
20
10
1
1.0
1.5
2.0
2.5
3.0
3.5
4.0
0
50
100
150
200
250
300
350

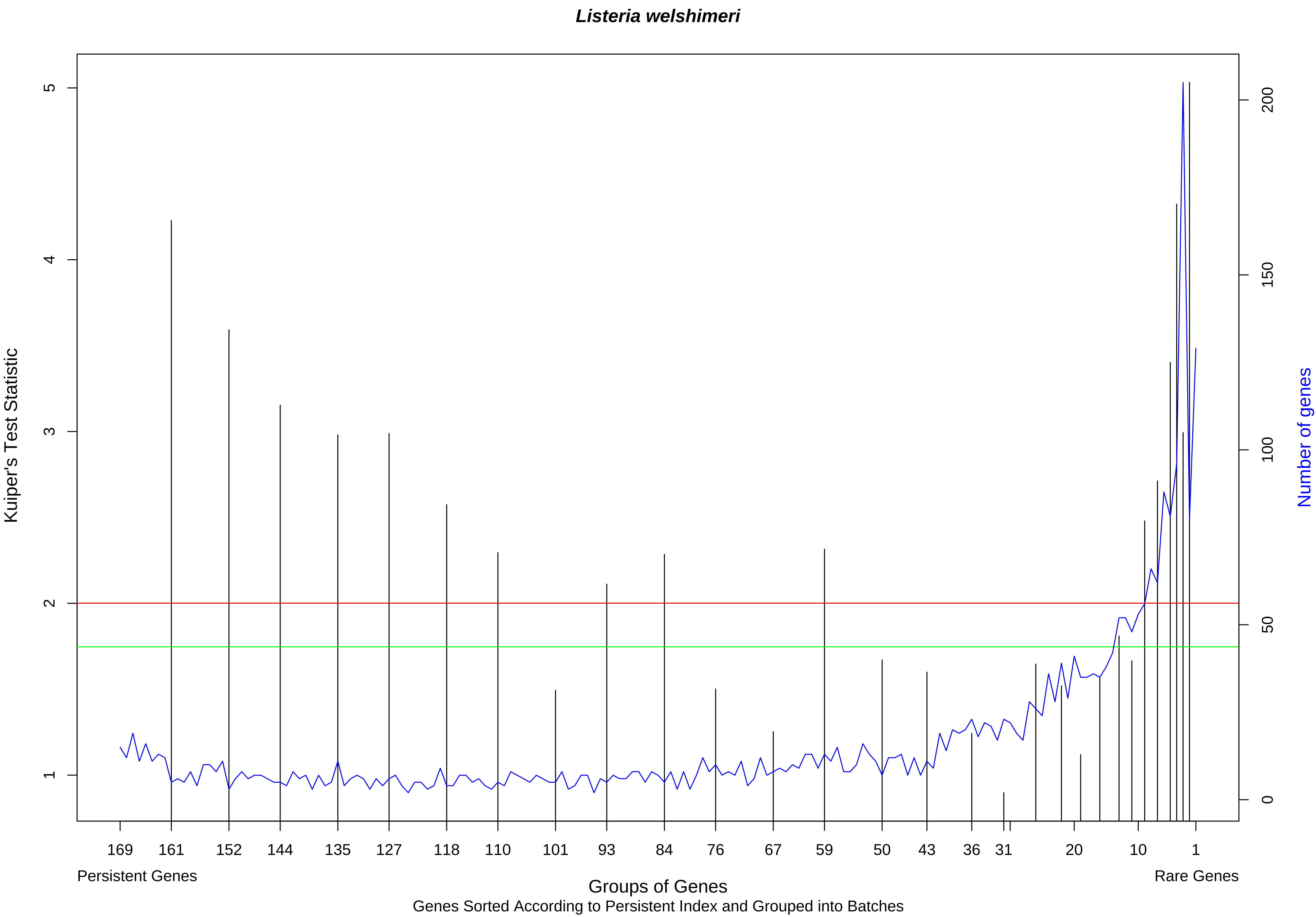

Listeria welshimeri
Kuiper's Test Statistic
Number of genes
5
4
3
2
1
200
150
100
50
0
169
161
152
144
135
127
118
110
101
93
84
76
67
59
50
43
36
31
20
10
1
Persistent Genes
Rare Genes
Groups of Genes
Genes Sorted According to Persistent Index and Grouped into Batches

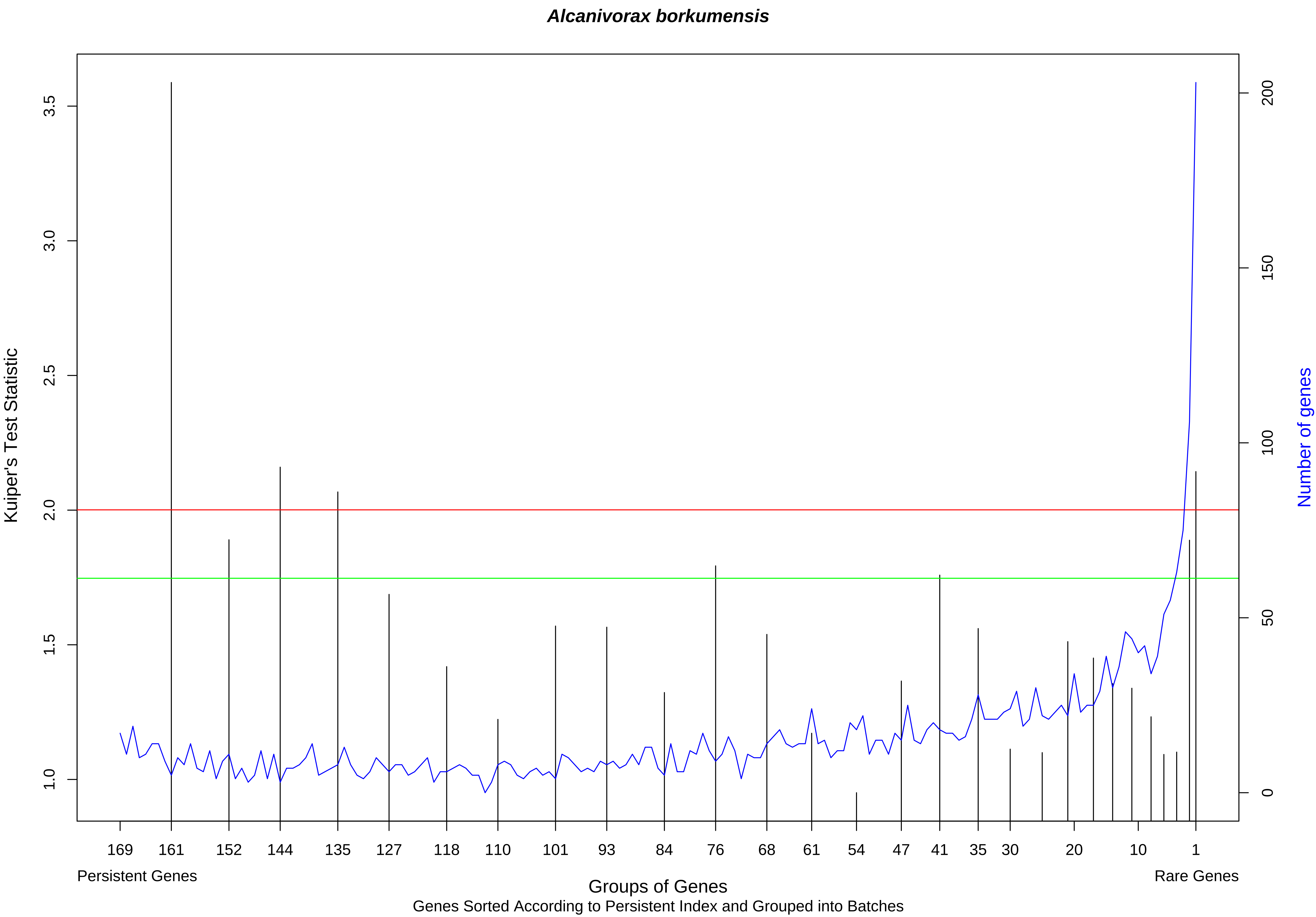
Alcanivorax borkumensis
Kuiper's Test Statistic
1.0
1.5
2.0
2.5
3.0
3.5
Number of genes
0
50
100
150
200
169
161
152
144
135
127
118
110
101
93
84
76
68
61
54
47
41
35
30
20
10
1
Persistent Genes
Rare Genes
Groups of Genes
Genes Sorted According to Persistent Index and Grouped into Batches

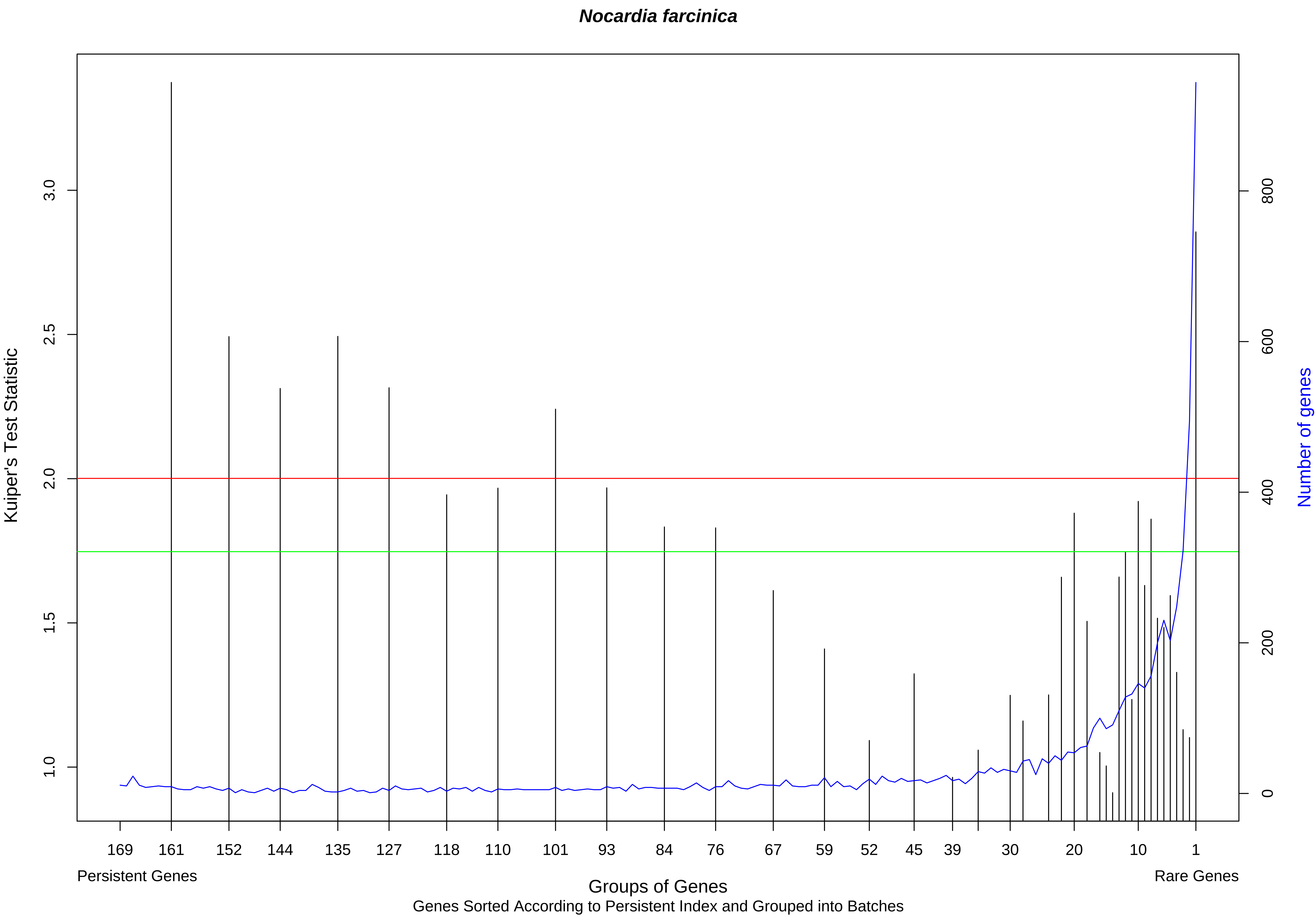
Nocardia farcinica
Kuiper's Test Statistic
1.0
1.5
2.0
2.5
3.0
Number of genes
0
200
400
600
800
169
161
152
144
135
127
118
110
101
93
84
76
67
59
52
45
39
30
20
10
1
Persistent Genes
Rare Genes
Groups of Genes
Genes Sorted According to Persistent Index and Grouped into Batches

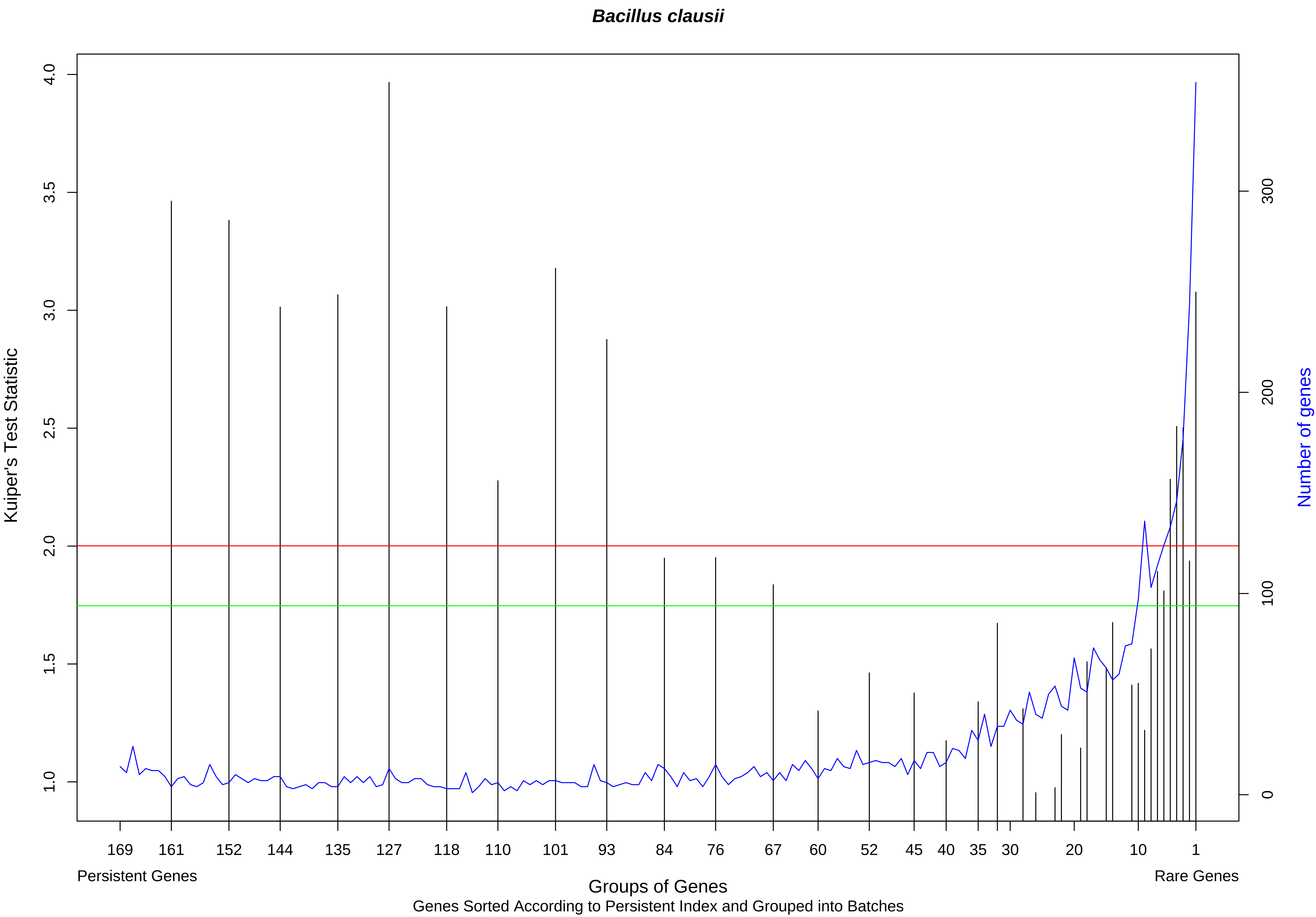

Bacillus clausii
Kuiper's Test Statistic
1.0
1.5
2.0
2.5
3.0
3.5
4.0
Number of genes
0
100
200
300
169
161
152
144
135
127
118
110
101
93
84
76
67
60
52
45
40
35
30
20
10
1
Persistent Genes
Rare Genes
Groups of Genes
Genes Sorted According to Persistent Index and Grouped into Batches

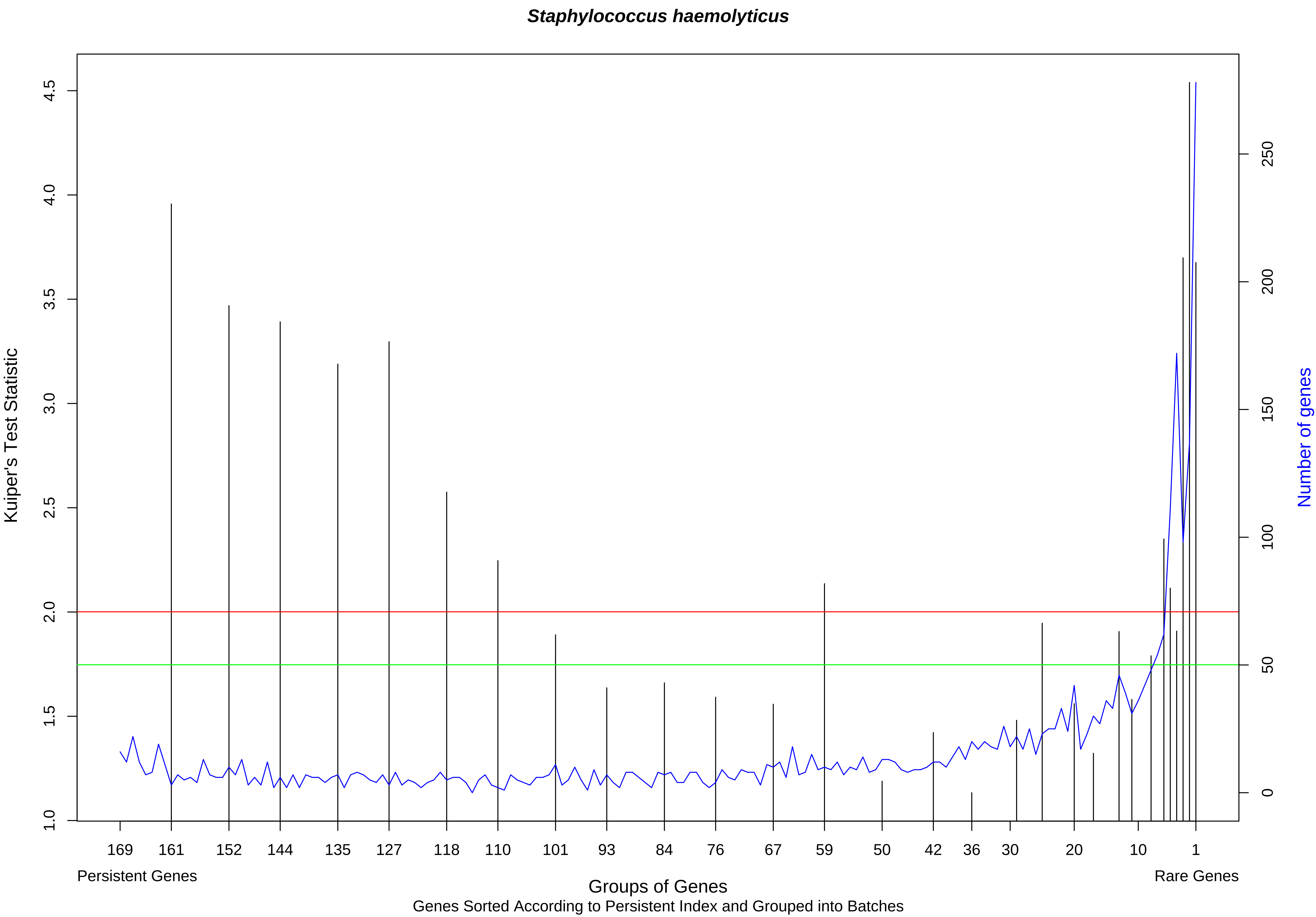

Staphylococcus haemolyticus
Kuiper's Test Statistic
Number of genes
1.0
1.5
2.0
2.5
3.0
3.5
4.0
4.5
0
50
100
150
200
250
169
161
152
144
135
127
118
110
101
93
84
76
67
59
50
42
36
30
20
10
1
Persistent Genes
Rare Genes
Groups of Genes
Genes Sorted According to Persistent Index and Grouped into Batches

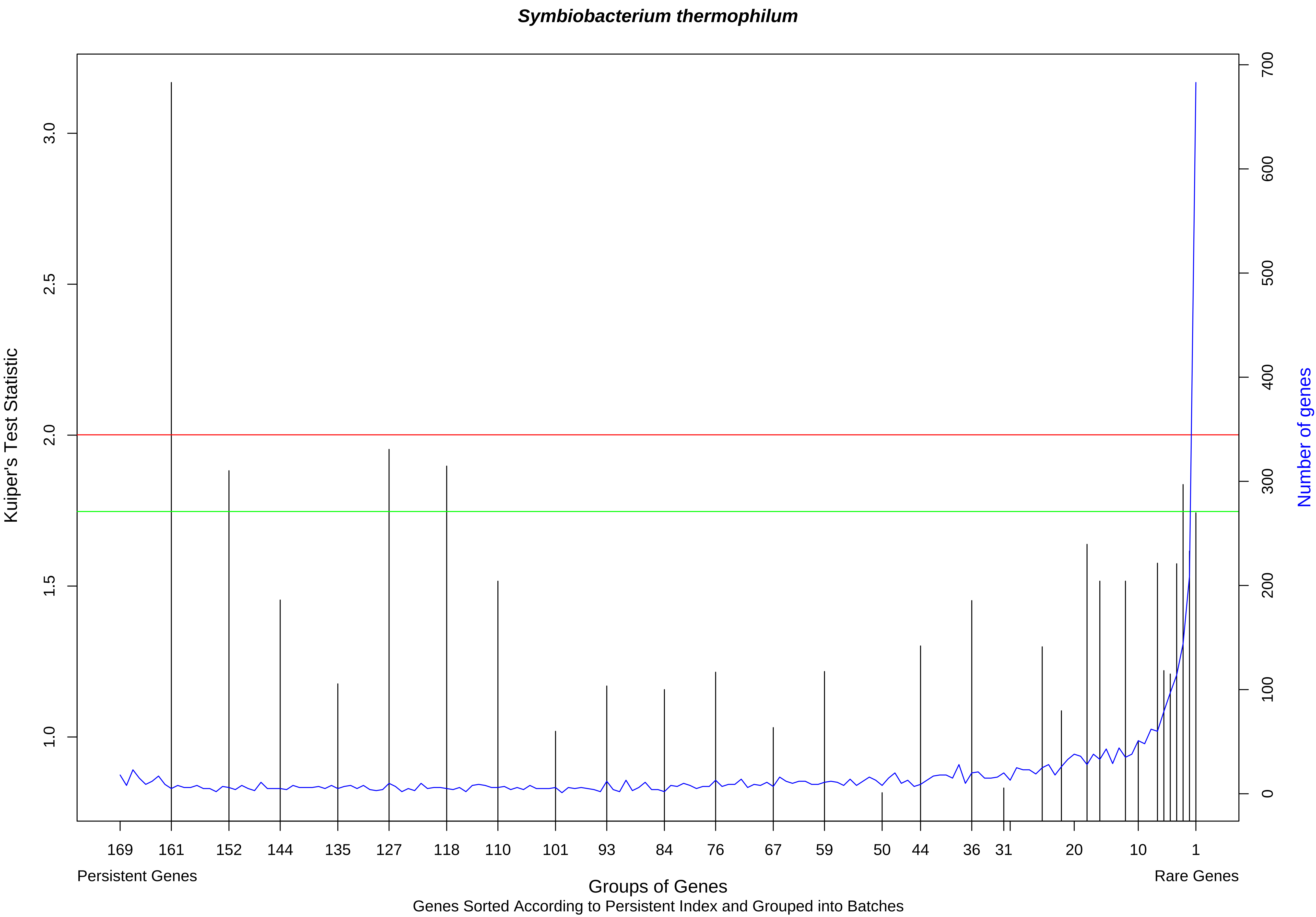
Symbiobacterium thermophilum
Kuiper's Test Statistic
Number of genes
3.0
2.5
2.0
1.5
1.0
700
600
500
400
300
200
100
0
169
161
152
144
135
127
118
110
101
93
84
76
67
59
50
44
36
31
20
10
1
Persistent Genes
Rare Genes
Groups of Genes
Genes Sorted According to Persistent Index and Grouped into Batches

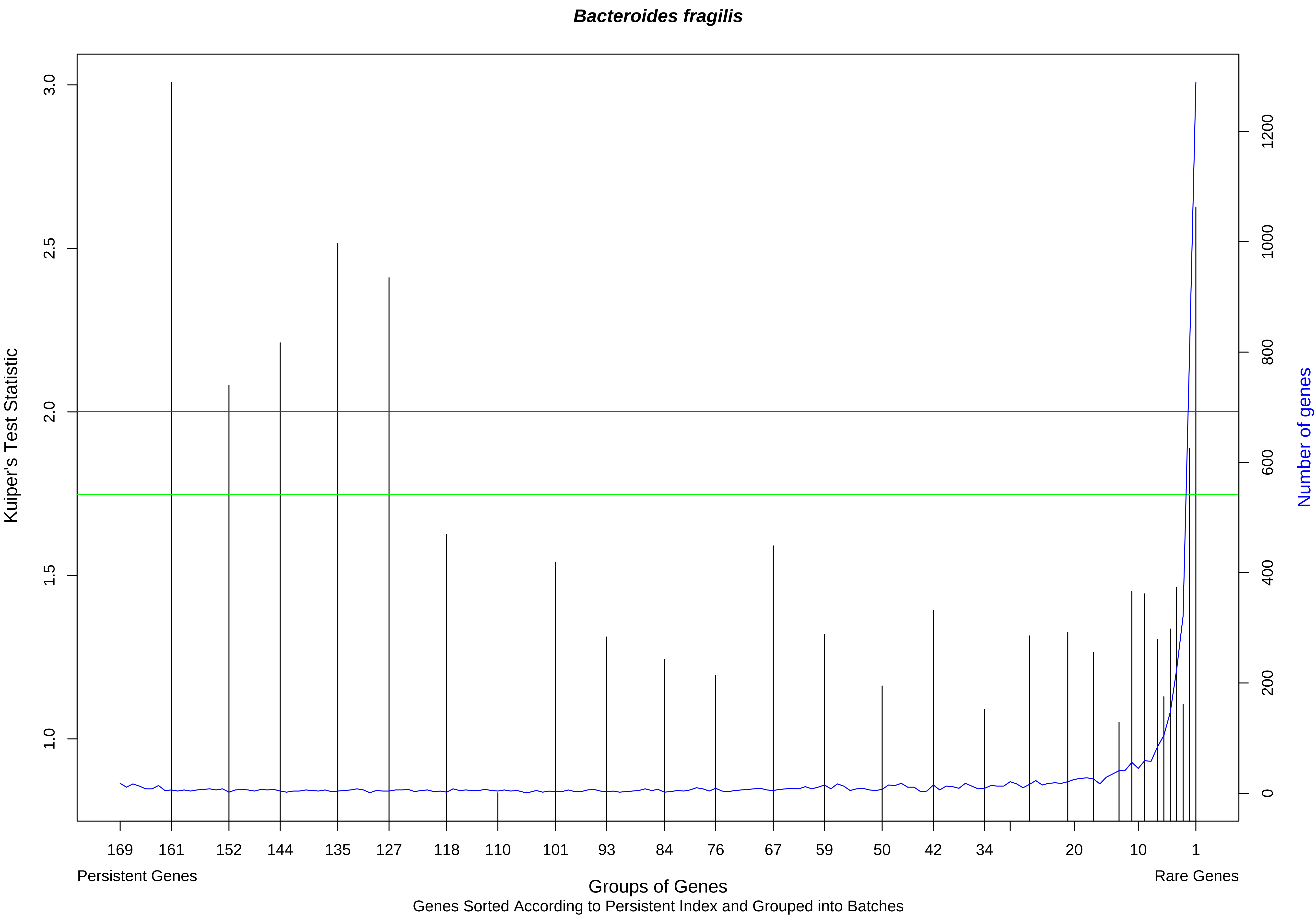
Bacteroides fragilis
Kuiper's Test Statistic
3.0
2.5
2.0
1.5
1.0
Number of genes
1200
1000
800
600
400
200
0
169
161
152
144
135
127
118
110
101
93
84
76
67
59
50
42
34
20
10
1
Persistent Genes
Rare Genes
Groups of Genes
Genes Sorted According to Persistent Index and Grouped into Batches

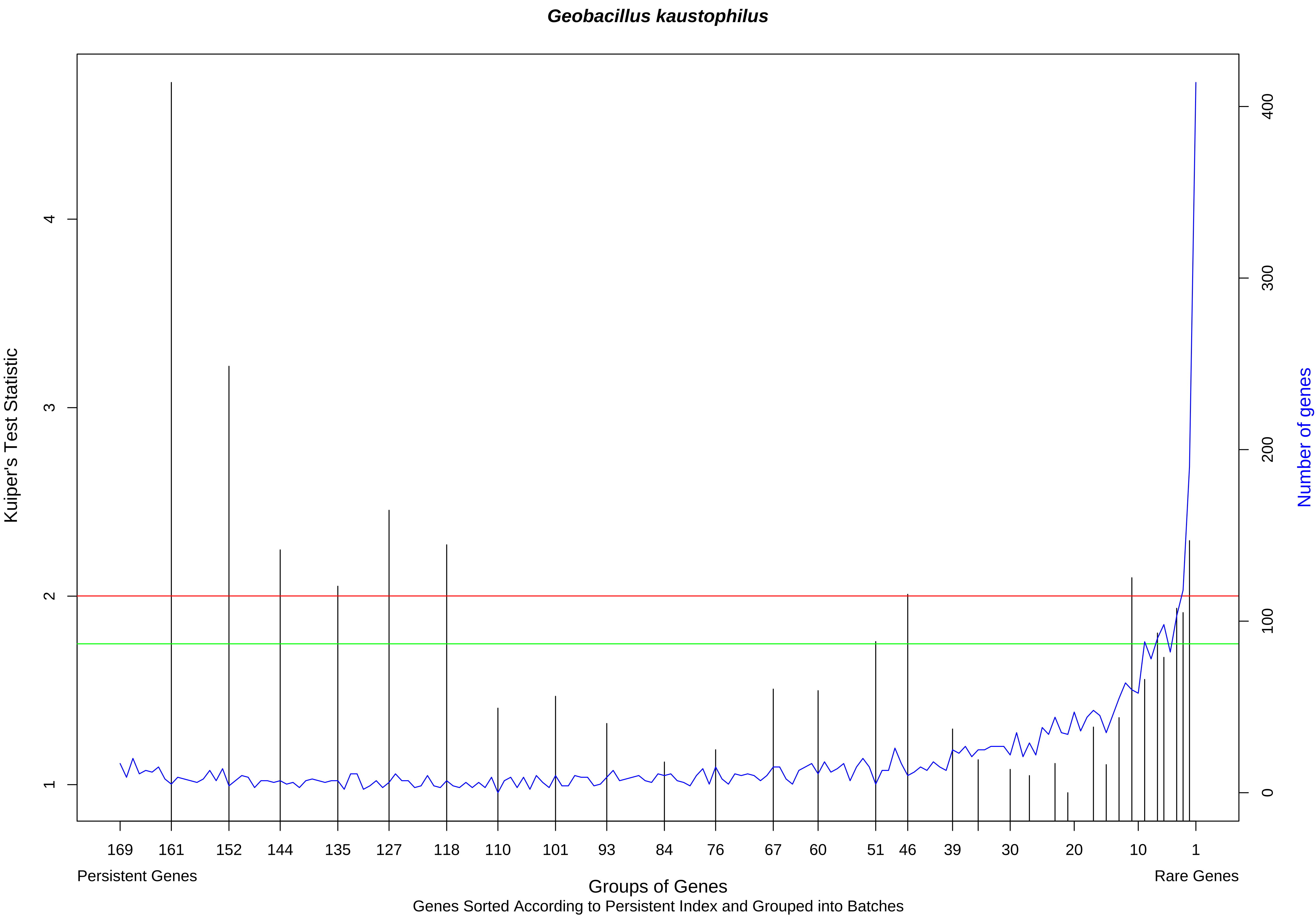

Geobacillus kaustophilus
Kuiper's Test Statistic
1
2
3
4
Number of genes
0
100
200
300
400
169
161
152
144
135
127
118
110
101
93
84
76
67
60
51
46
39
30
20
10
1
Persistent Genes
Rare Genes
Groups of Genes
Genes Sorted According to Persistent Index and Grouped into Batches

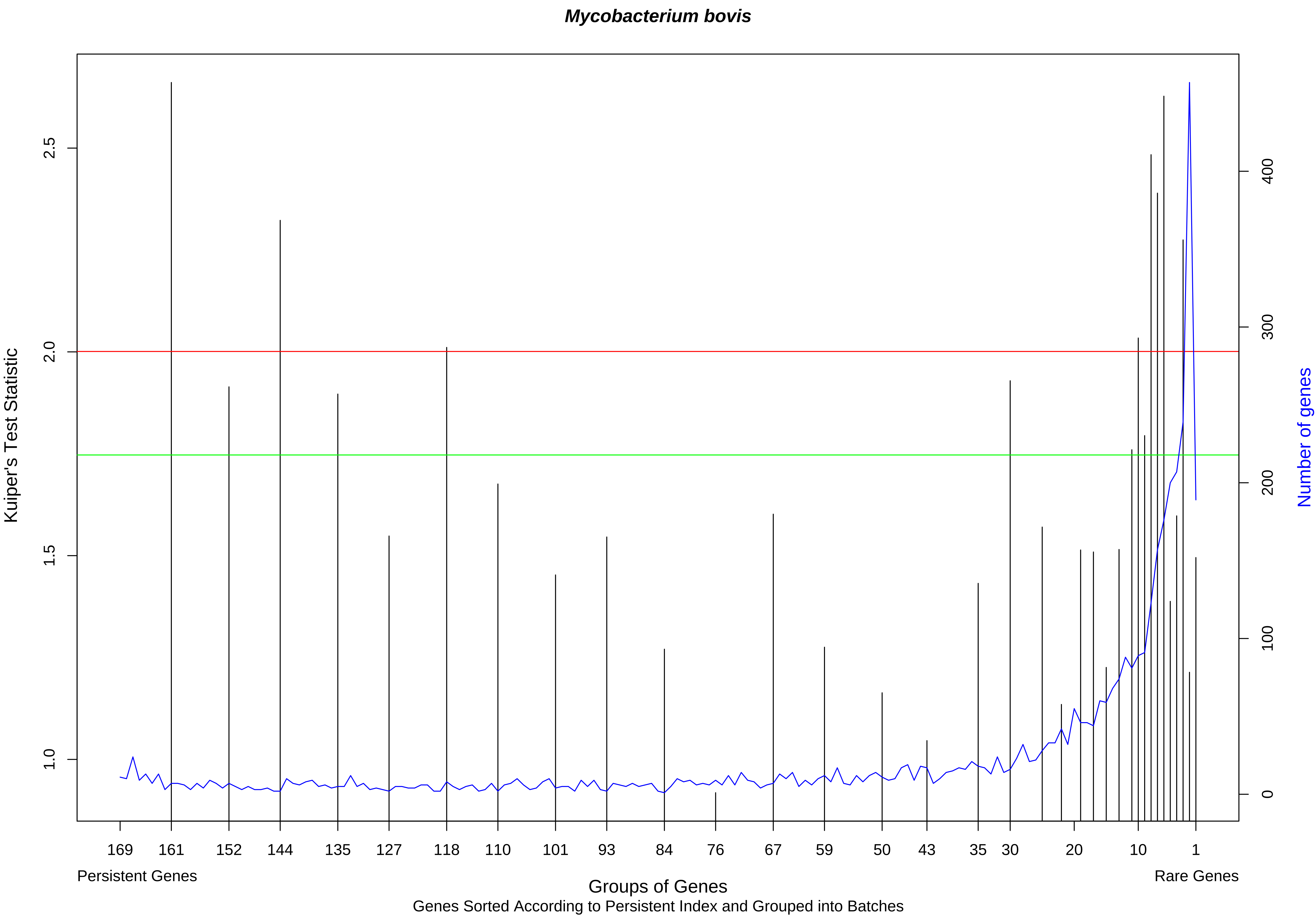

Mycobacterium bovis
Kuiper's Test Statistic
Number of genes
1.0
1.5
2.0
2.5
0
100
200
300
400
169
161
152
144
135
127
118
110
101
93
84
76
67
59
50
43
35
30
20
10
1
Persistent Genes
Rare Genes
Groups of Genes
Genes Sorted According to Persistent Index and Grouped into Batches

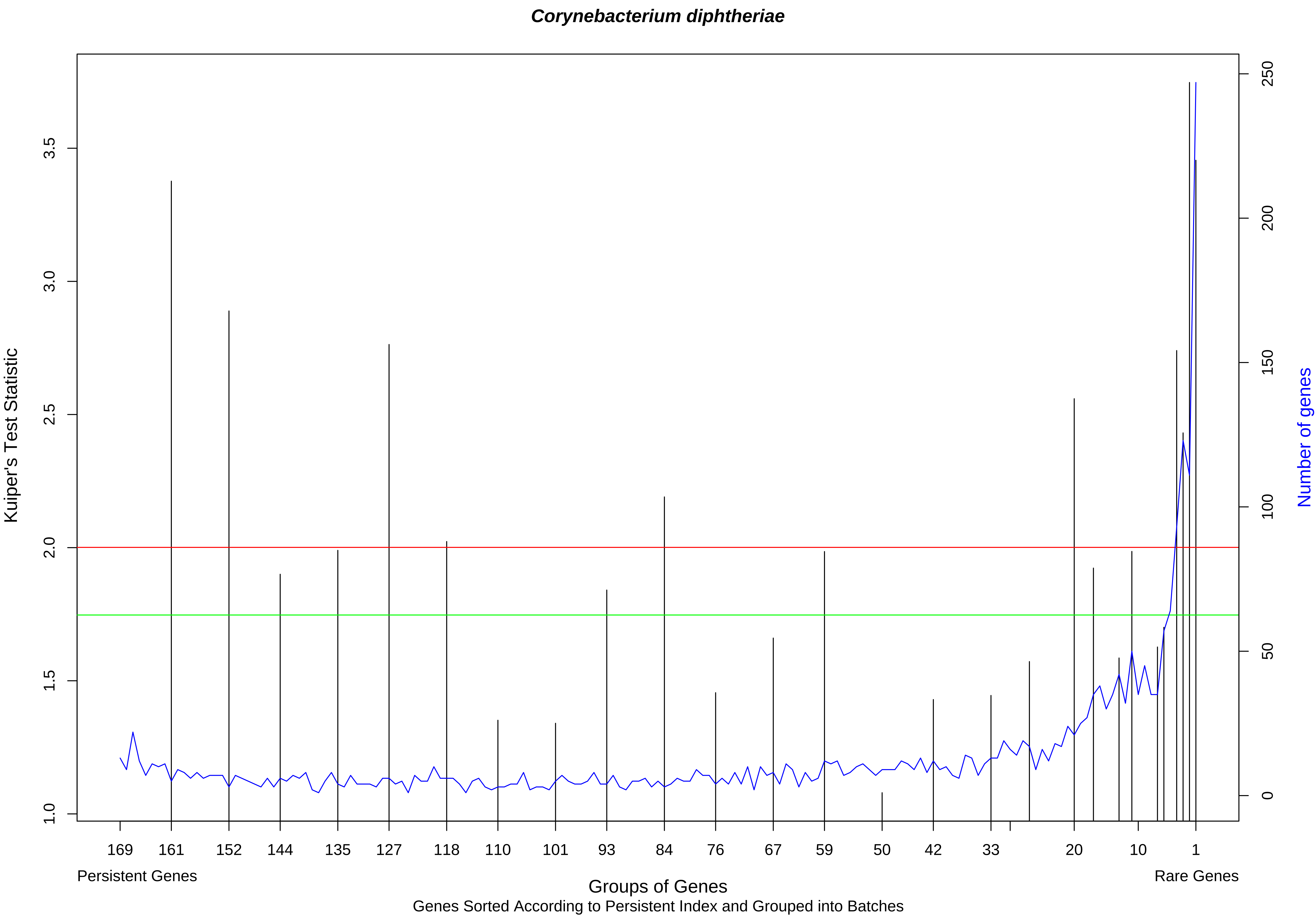

Corynebacterium diphtheriae
Kuiper's Test Statistic
Number of genes
1.0
1.5
2.0
2.5
3.0
3.5
0
50
100
150
200
250
169
161
152
144
135
127
118
110
101
93
84
76
67
59
50
42
33
20
10
1
Persistent Genes
Rare Genes
Groups of Genes
Genes Sorted According to Persistent Index and Grouped into Batches

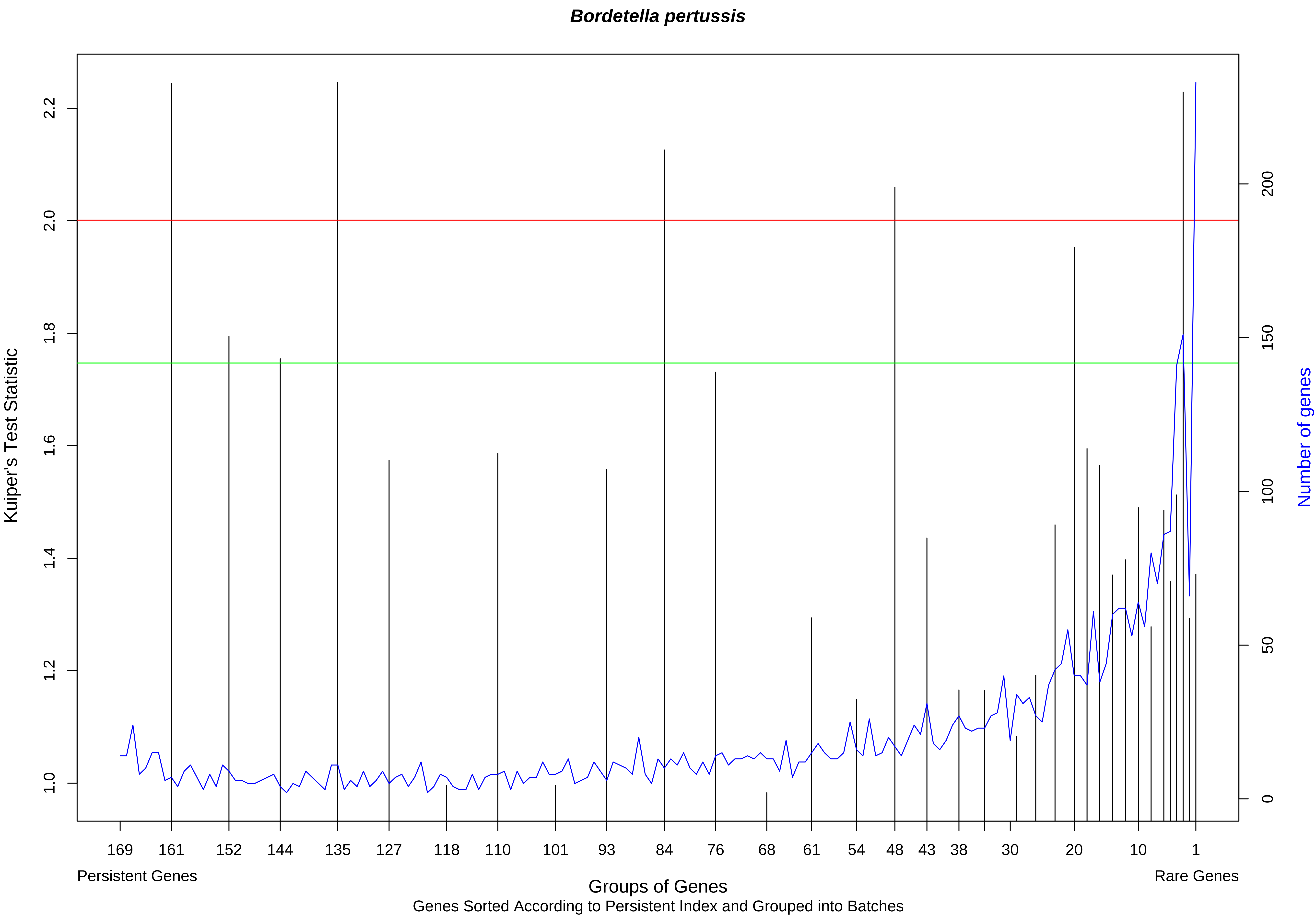

Bordetella pertussis
Kuiper's Test Statistic
1.0
1.2
1.4
1.6
1.8
2.0
2.2
Number of genes
0
50
100
150
200
169
161
152
144
135
127
118
110
101
93
84
76
68
61
54
48
43
38
30
20
10
1
Persistent Genes
Rare Genes
Groups of Genes
Genes Sorted According to Persistent Index and Grouped into Batches

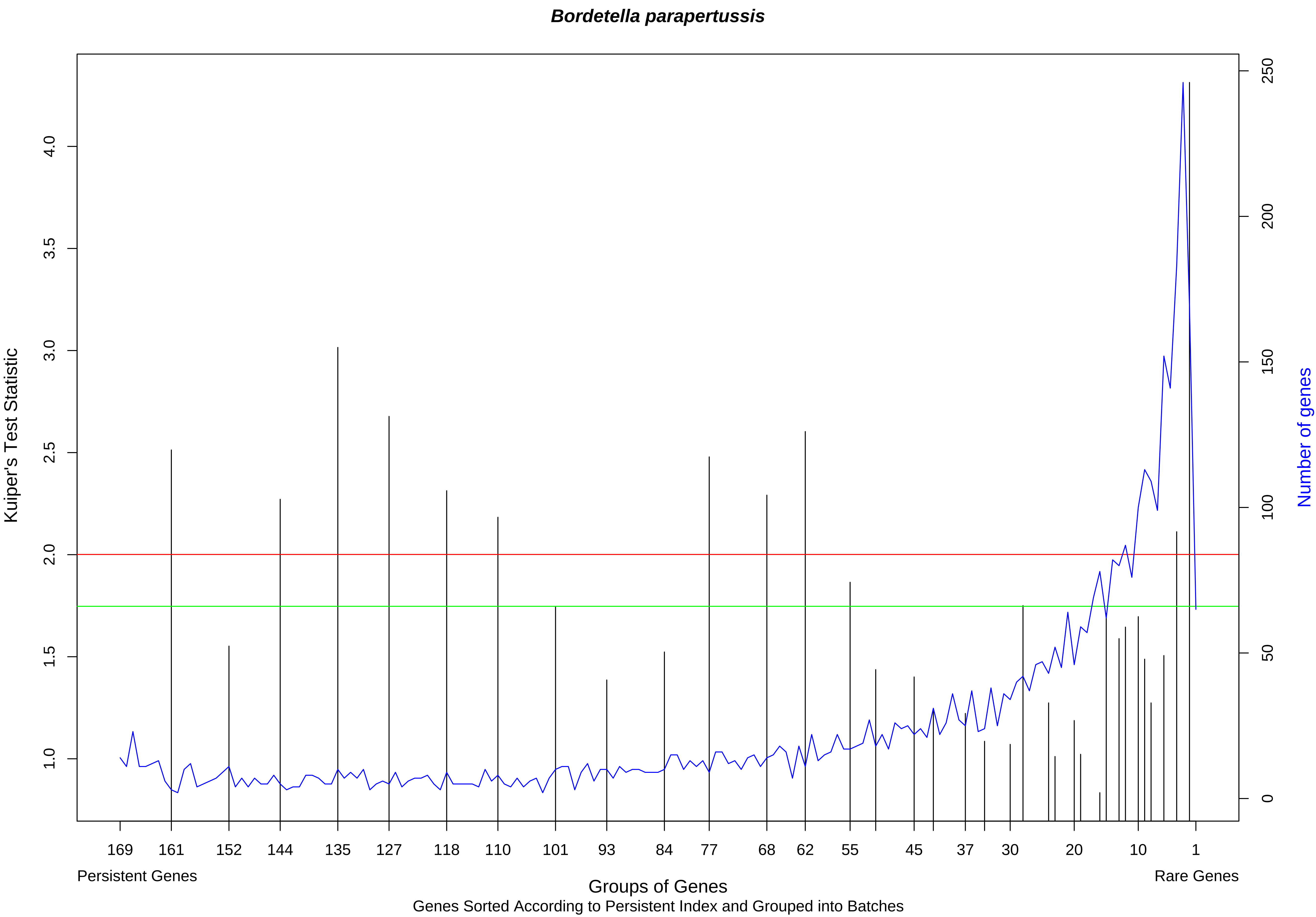
Bordetella parapertussis
Kuiper's Test Statistic
1.0
1.5
2.0
2.5
3.0
3.5
4.0
Number of genes
0
50
100
150
200
250
169
161
152
144
135
127
118
110
101
93
84
77
68
62
55
45
37
30
20
10
1
Persistent Genes
Rare Genes
Groups of Genes
Genes Sorted According to Persistent Index and Grouped into Batches

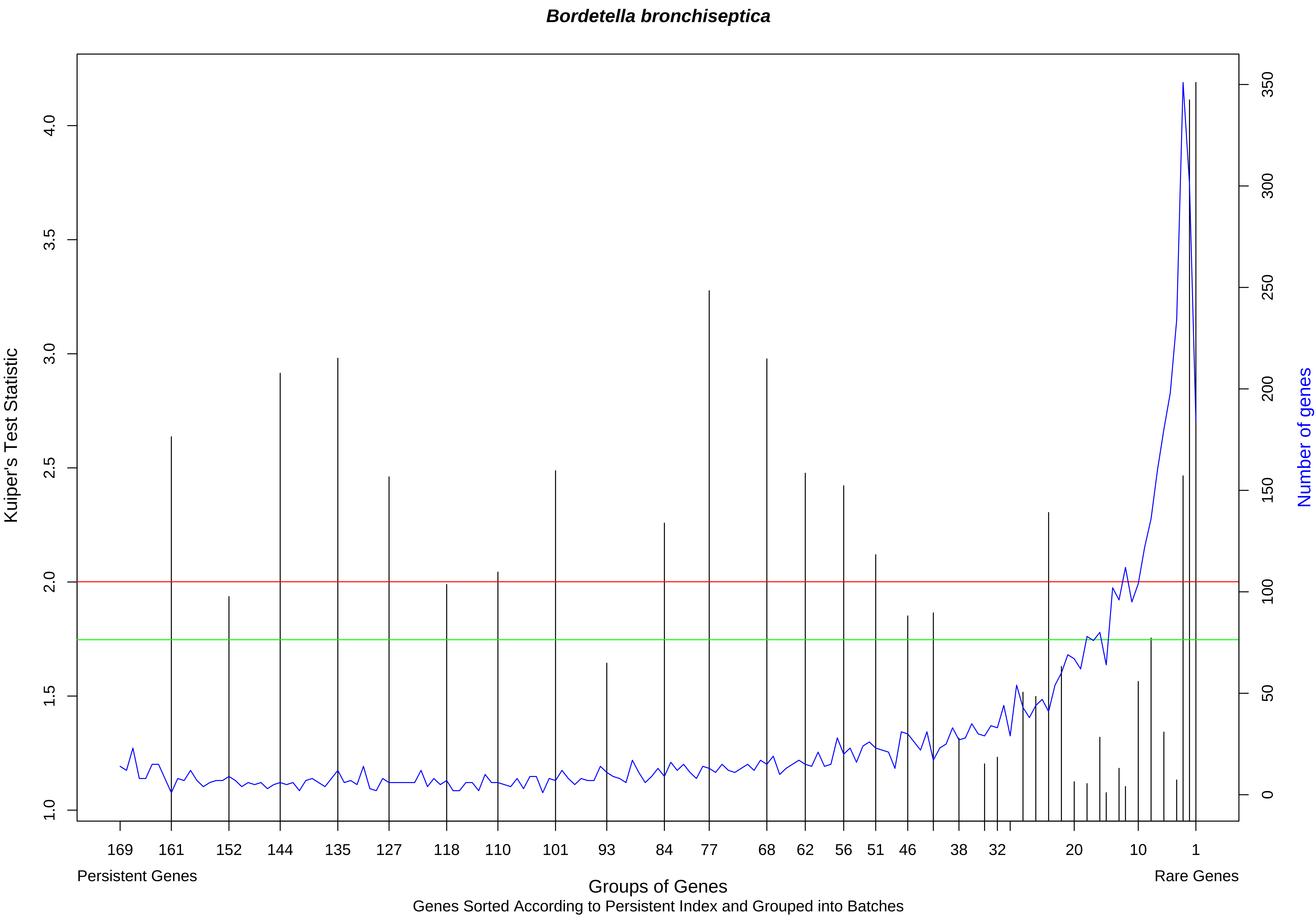
Bordetella bronchiseptica
Kuiper's Test Statistic
Number of genes
1.0
1.5
2.0
2.5
3.0
3.5
4.0
0
50
100
150
200
250
300
350
169
161
152
144
135
127
118
110
101
93
84
77
68
62
56
51
46
38
32
20
10
1
Persistent Genes
Rare Genes
Groups of Genes
Genes Sorted According to Persistent Index and Grouped into Batches

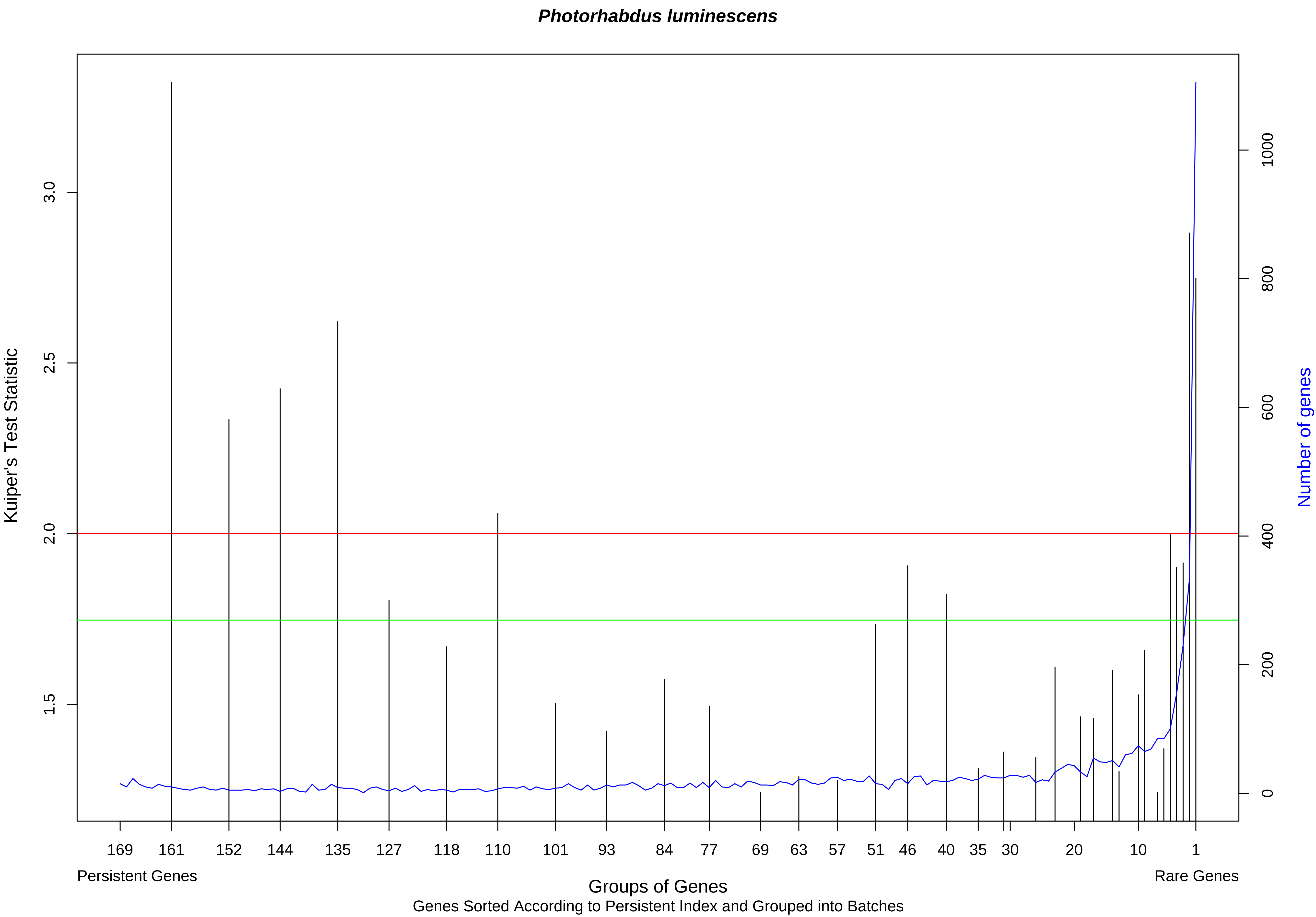
Photorhabdus luminescens
Kuiper's Test Statistic
3.0
2.5
2.0
1.5
Number of genes
1000
800
600
400
200
0
169
161
152
144
135
127
118
110
101
93
84
77
69
63
57
51
46
40
35
30
20
10
1
Persistent Genes
Rare Genes
Groups of Genes
Genes Sorted According to Persistent Index and Grouped into Batches

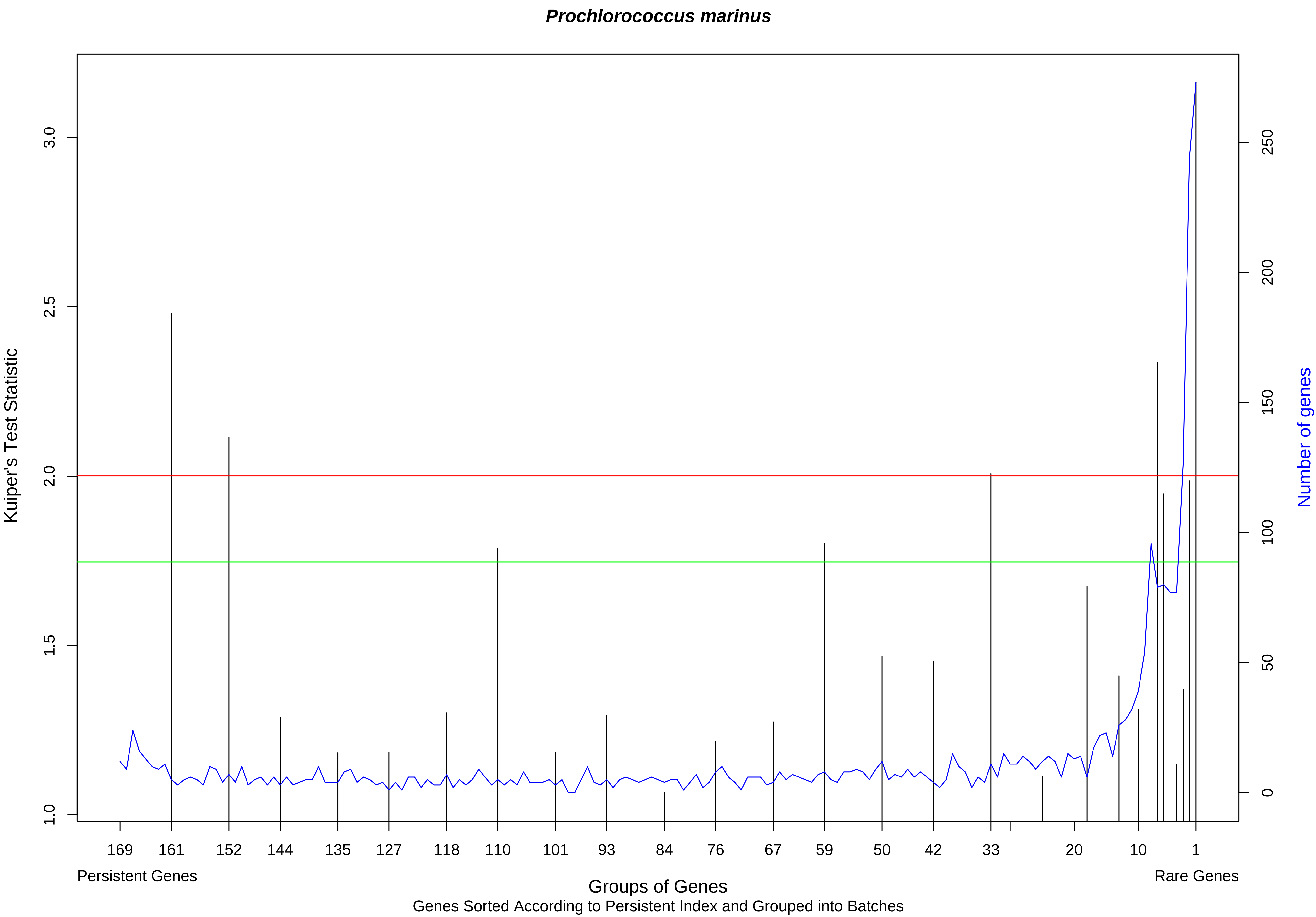

Prochlorococcus marinus
Kuiper's Test Statistic
1.0
1.5
2.0
2.5
3.0
Number of genes
0
50
100
150
200
250
169
161
152
144
135
127
118
110
101
93
84
76
67
59
50
42
33
20
10
1
Persistent Genes
Rare Genes
Groups of Genes
Genes Sorted According to Persistent Index and Grouped into Batches

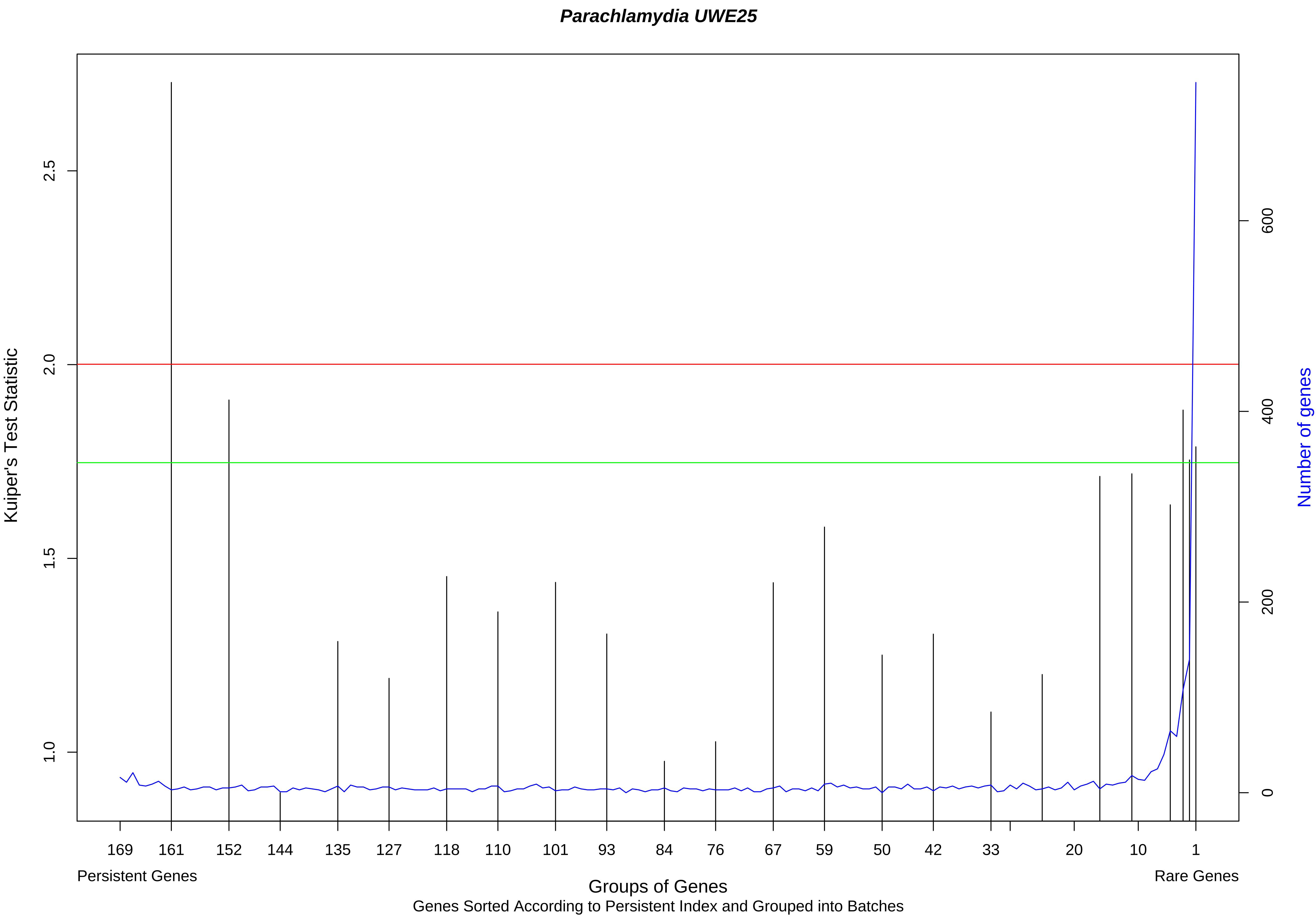
Parachlamydia UWE25
Kuiper's Test Statistic
2.5
2.0
1.5
1.0
Number of genes
600
400
200
0
169
161
152
144
135
127
118
110
101
93
84
76
67
59
50
42
33
20
10
1
Persistent Genes
Rare Genes
Groups of Genes
Genes Sorted According to Persistent Index and Grouped into Batches

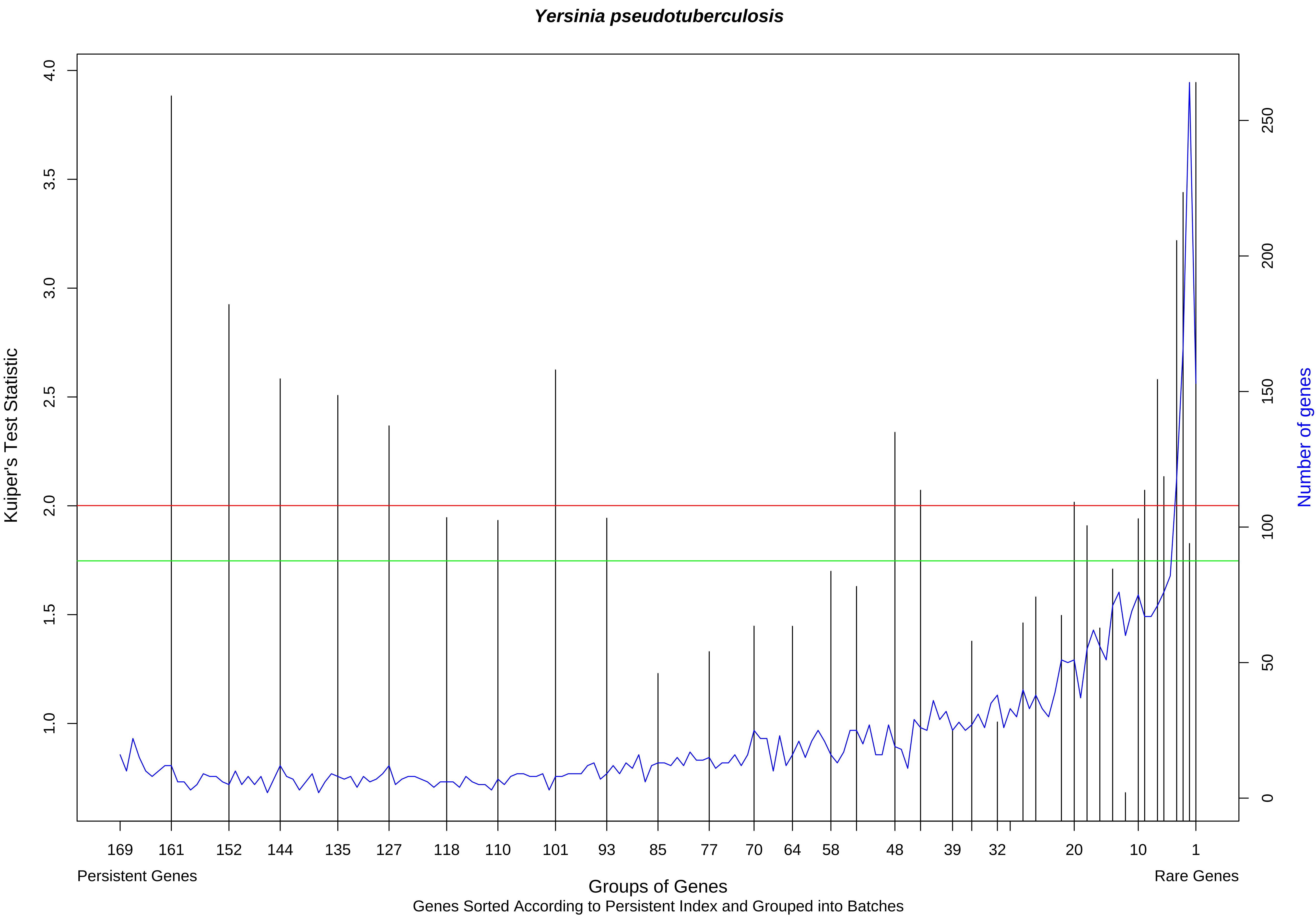

Yersinia pseudotuberculosis
Kuiper's Test Statistic
Number of genes
4.0
3.5
3.0
2.5
2.0
1.5
1.0
250
200
150
100
50
0
169
161
152
144
135
127
118
110
101
93
85
77
70
64
58
48
39
32
20
10
1
Persistent Genes
Rare Genes
Groups of Genes
Genes Sorted According to Persistent Index and Grouped into Batches

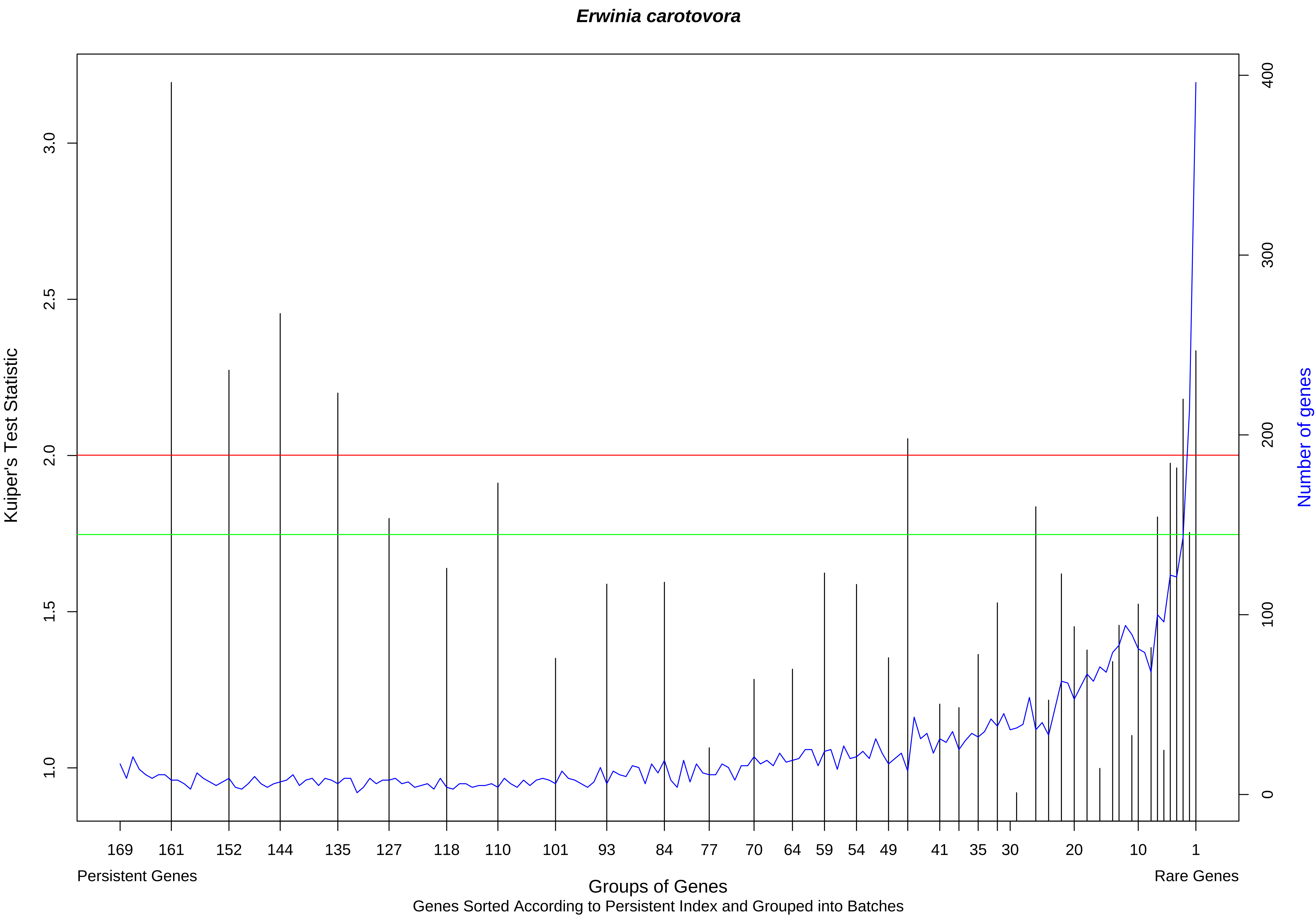
Erwinia carotovora
Kuiper's Test Statistic
Number of genes
1.0
1.5
2.0
2.5
3.0
0
100
200
300
400
169 161 152 144 135 127 118 110 101 93 84 77 70 64 59 54 49 41 35 30 20 10 1
Persistent Genes
Rare Genes
Groups of Genes
Genes Sorted According to Persistent Index and Grouped into Batches

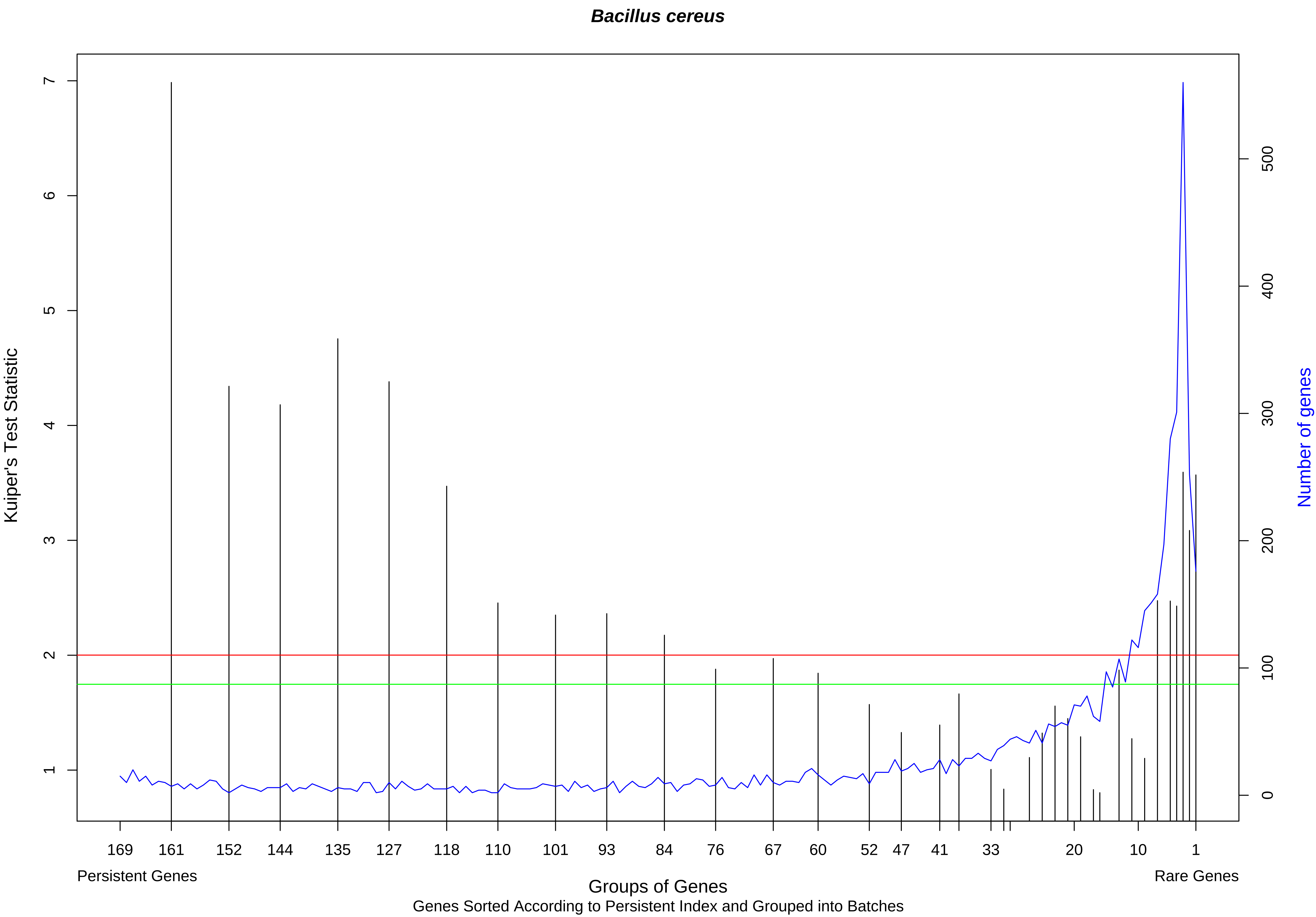

Bacillus cereus
Kuiper's Test Statistic
Number of genes
1
2
3
4
5
6
7
0
100
200
300
400
500
169
161
152
144
135
127
118
110
101
93
84
76
67
60
52
47
41
33
20
10
1
Persistent Genes
Rare Genes
Groups of Genes
Genes Sorted According to Persistent Index and Grouped into Batches

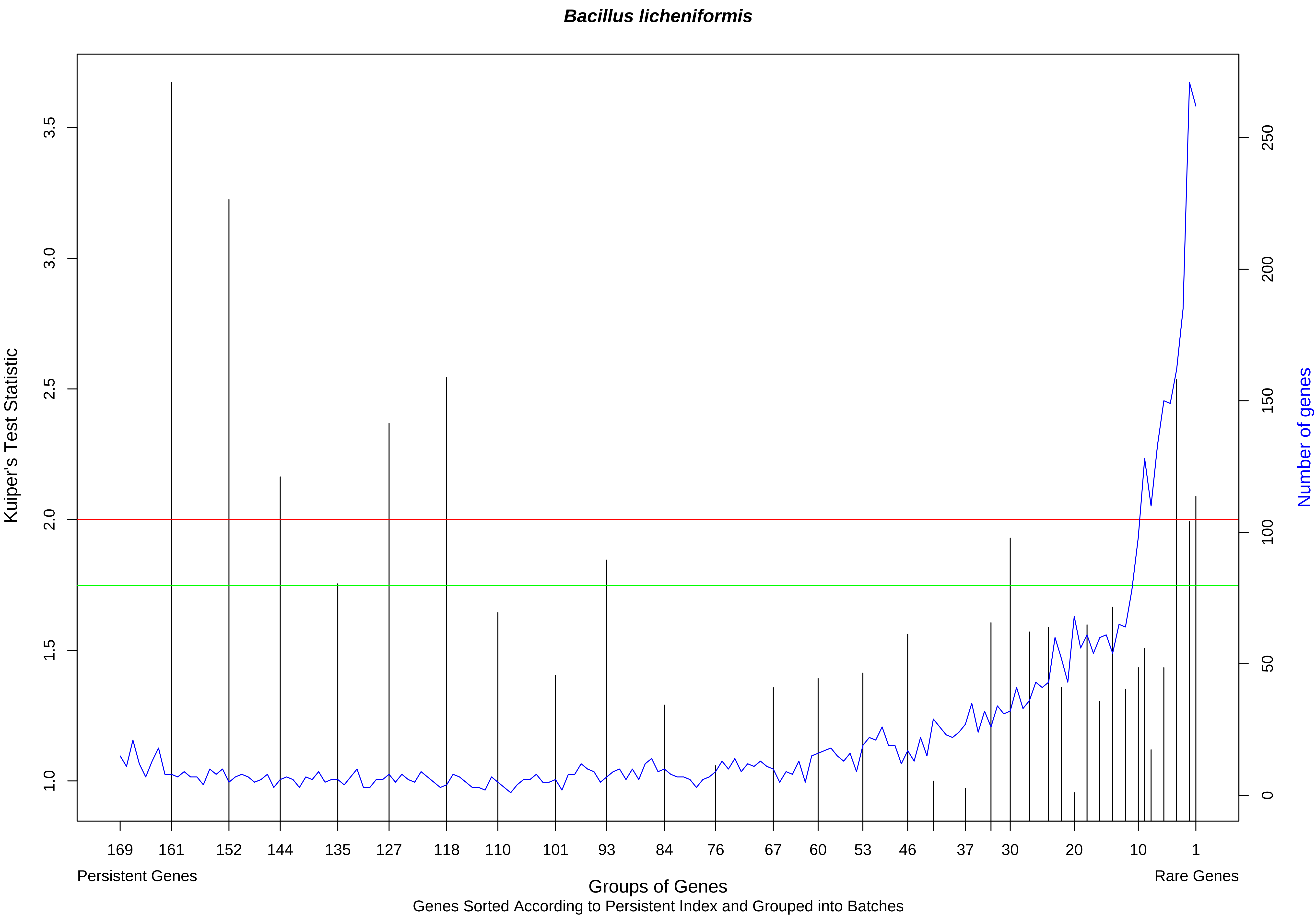
Bacillus licheniformis
Kuiper's Test Statistic
Number of genes
3.5
3.0
2.5
2.0
1.5
1.0
250
200
150
100
50
0
169
161
152
144
135
127
118
110
101
93
84
76
67
60
53
46
37
30
20
10
1
Persistent Genes
Rare Genes
Groups of Genes
Genes Sorted According to Persistent Index and Grouped into Batches

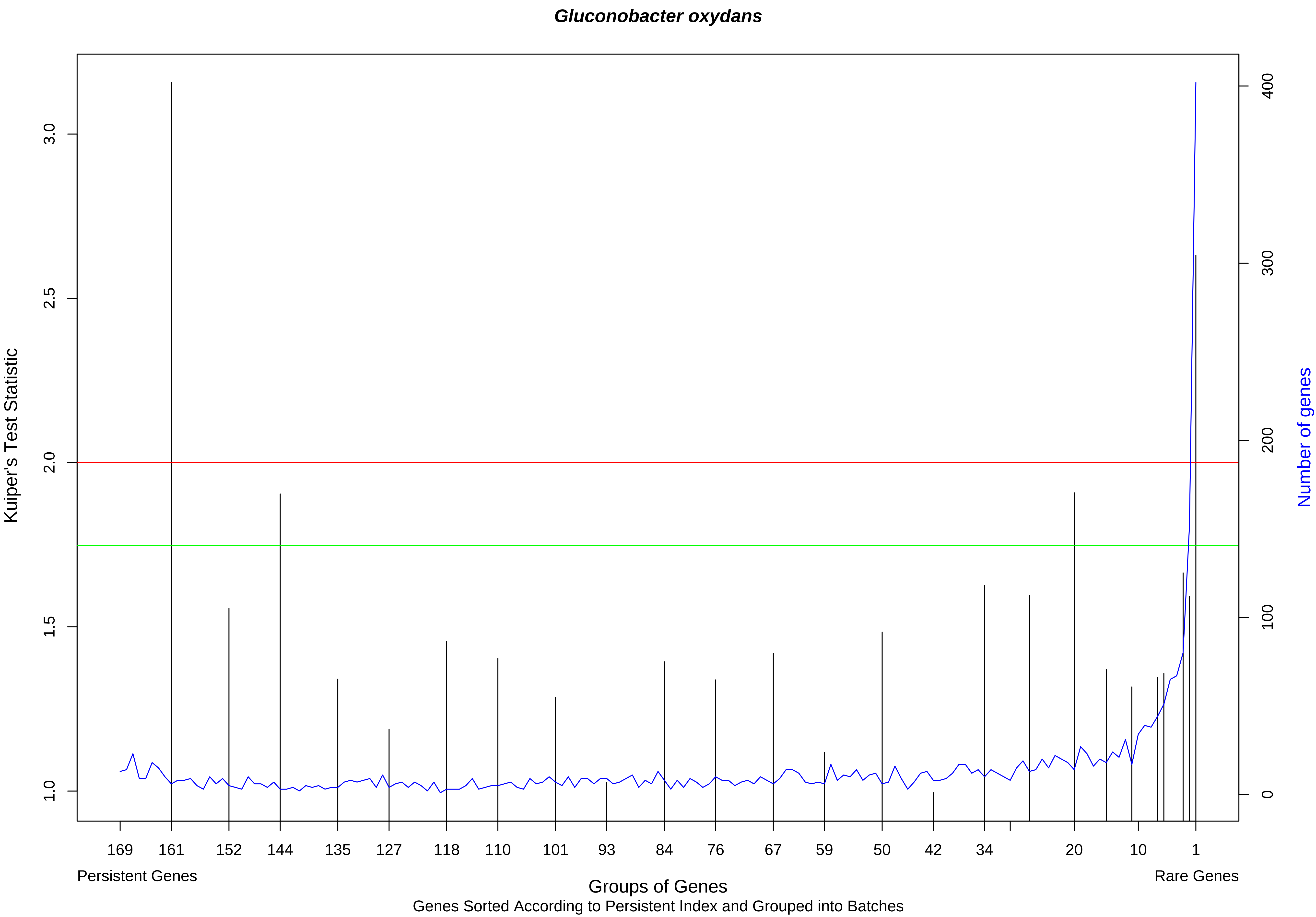
Gluconobacter oxydans
Kuiper's Test Statistic
1.0
1.5
2.0
2.5
3.0
Number of genes
0
100
200
300
400
169
161
152
144
135
127
118
110
101
93
84
76
67
59
50
42
34
20
10
1
Persistent Genes
Rare Genes
Groups of Genes
Genes Sorted According to Persistent Index and Grouped into Batches

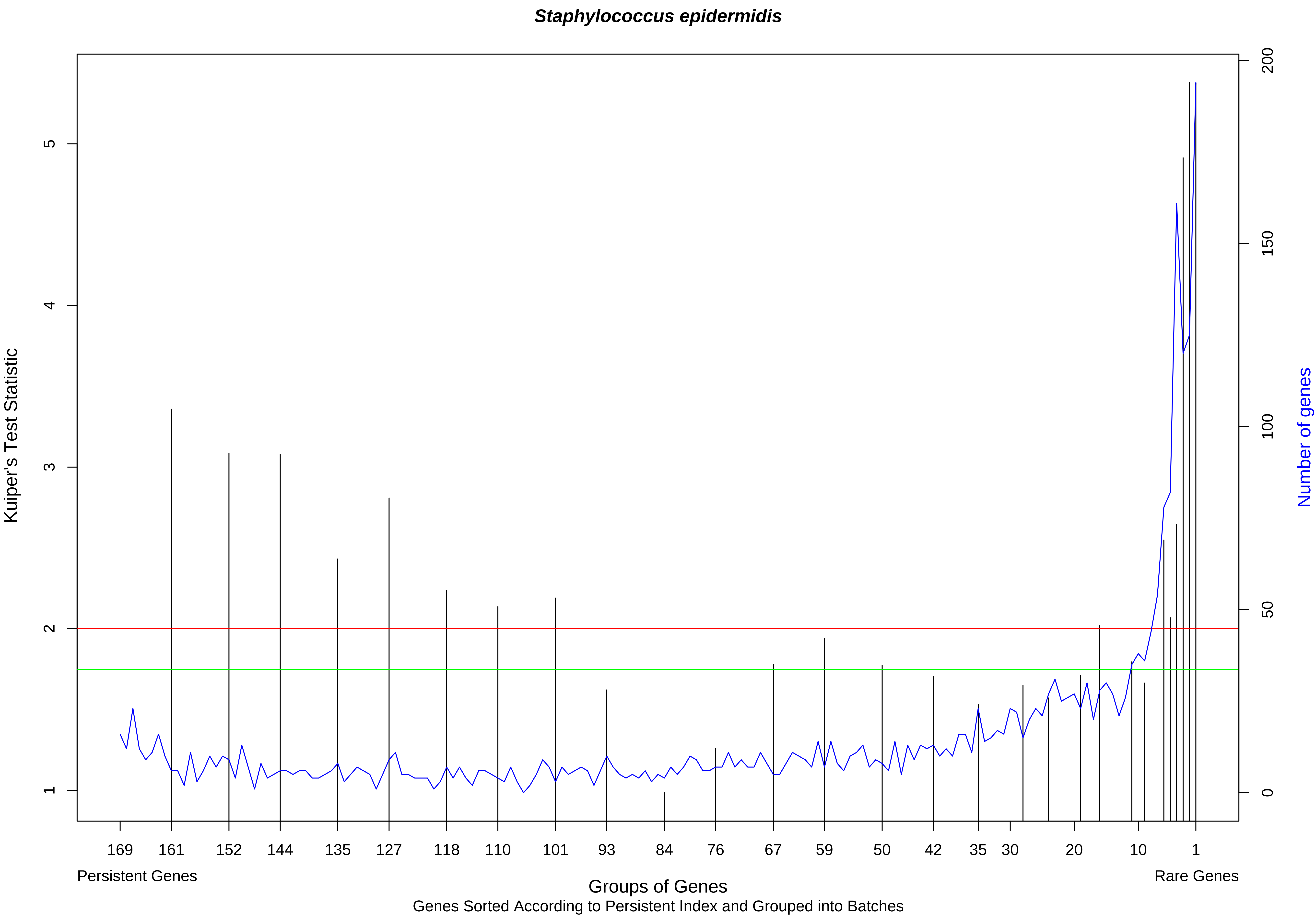

Staphylococcus epidermidis
Kuiper's Test Statistic
1
2
3
4
5
Number of genes
0
50
100
150
200
169
161
152
144
135
127
118
110
101
93
84
76
67
59
50
42
35
30
20
10
1
Persistent Genes
Rare Genes
Groups of Genes
Genes Sorted According to Persistent Index and Grouped into Batches

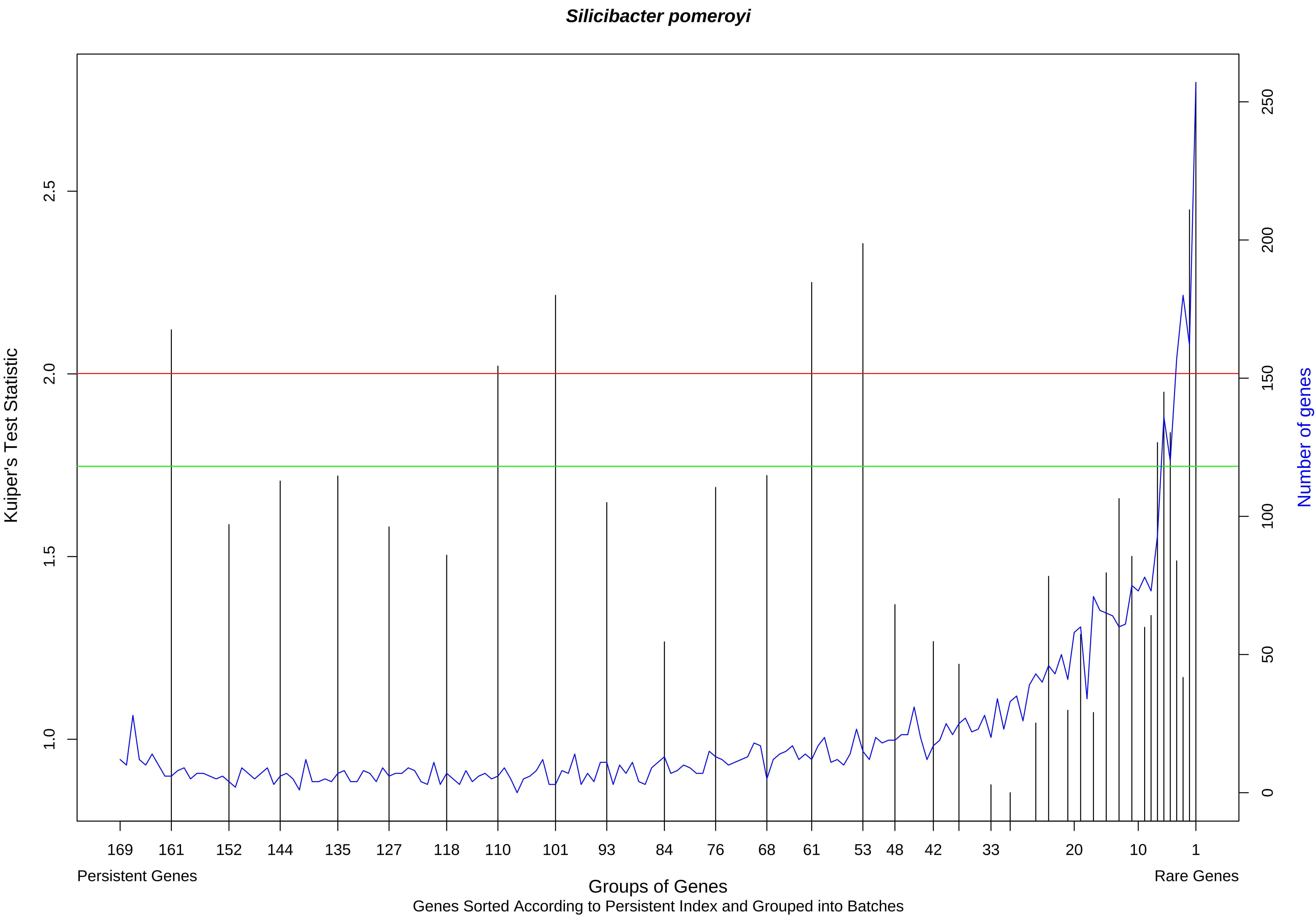

Silicibacter pomeroyi
Kuiper's Test Statistic
Number of genes
1.0
1.5
2.0
2.5
0
50
100
150
200
250
169
161
152
144
135
127
118
110
101
93
84
76
68
61
53
48
42
33
20
10
1
Persistent Genes
Rare Genes
Groups of Genes
Genes Sorted According to Persistent Index and Grouped into Batches

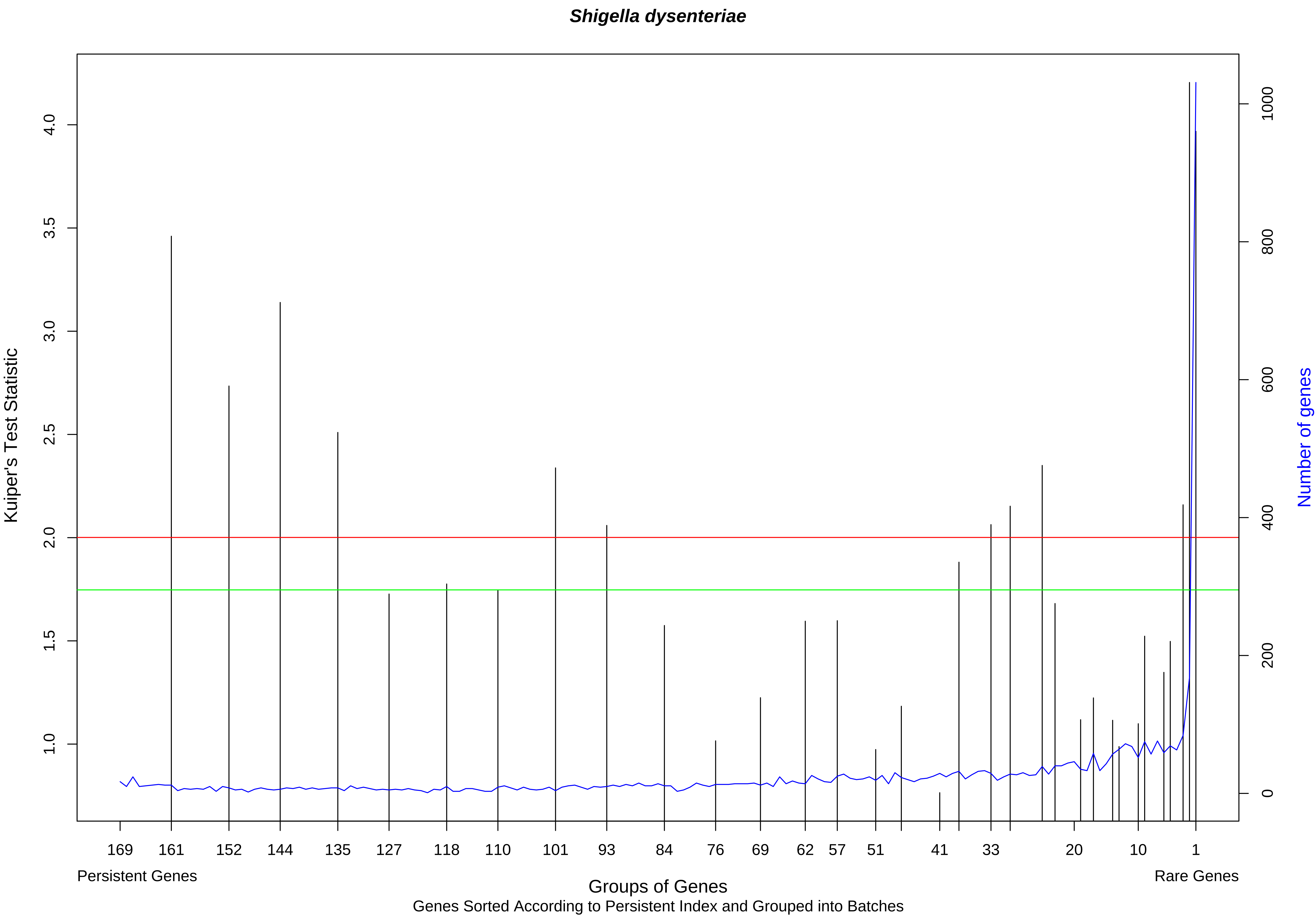

Shigella dysenteriae
Kuiper's Test Statistic
1.0
1.5
2.0
2.5
3.0
3.5
4.0
Number of genes
0
200
400
600
800
1000
169
161
152
144
135
127
118
110
101
93
84
76
69
62
57
51
41
33
20
10
1
Persistent Genes
Rare Genes
Groups of Genes
Genes Sorted According to Persistent Index and Grouped into Batches

Shigella boydii

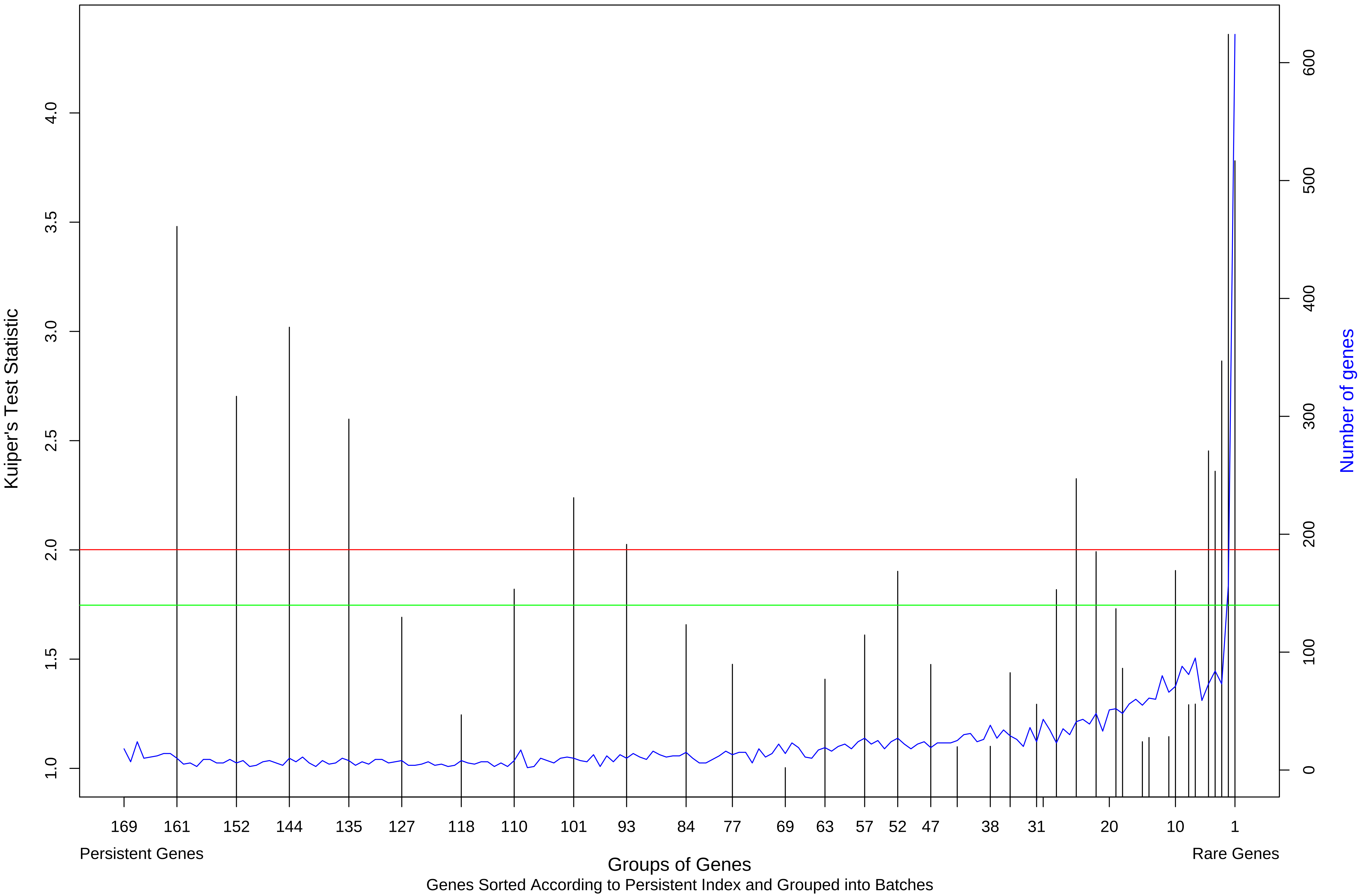

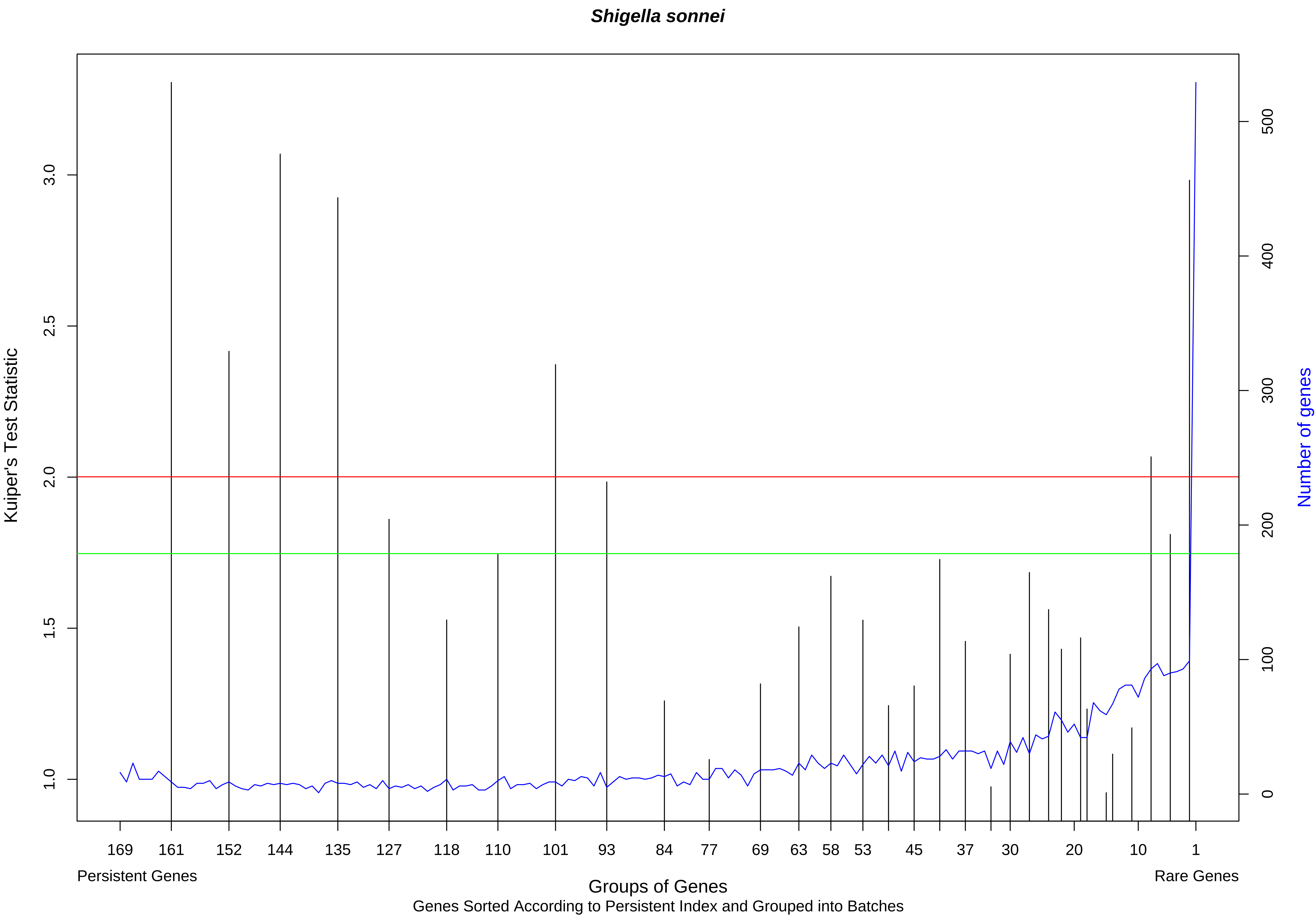

Shigella sonnei
Kuiper's Test Statistic
Number of genes
1.0
1.5
2.0
2.5
3.0
0
100
200
300
400
500
169
161
152
144
135
127
118
110
101
93
84
77
69
63
58
53
45
37
30
20
10
1
Persistent Genes
Rare Genes
Groups of Genes
Genes Sorted According to Persistent Index and Grouped into Batches

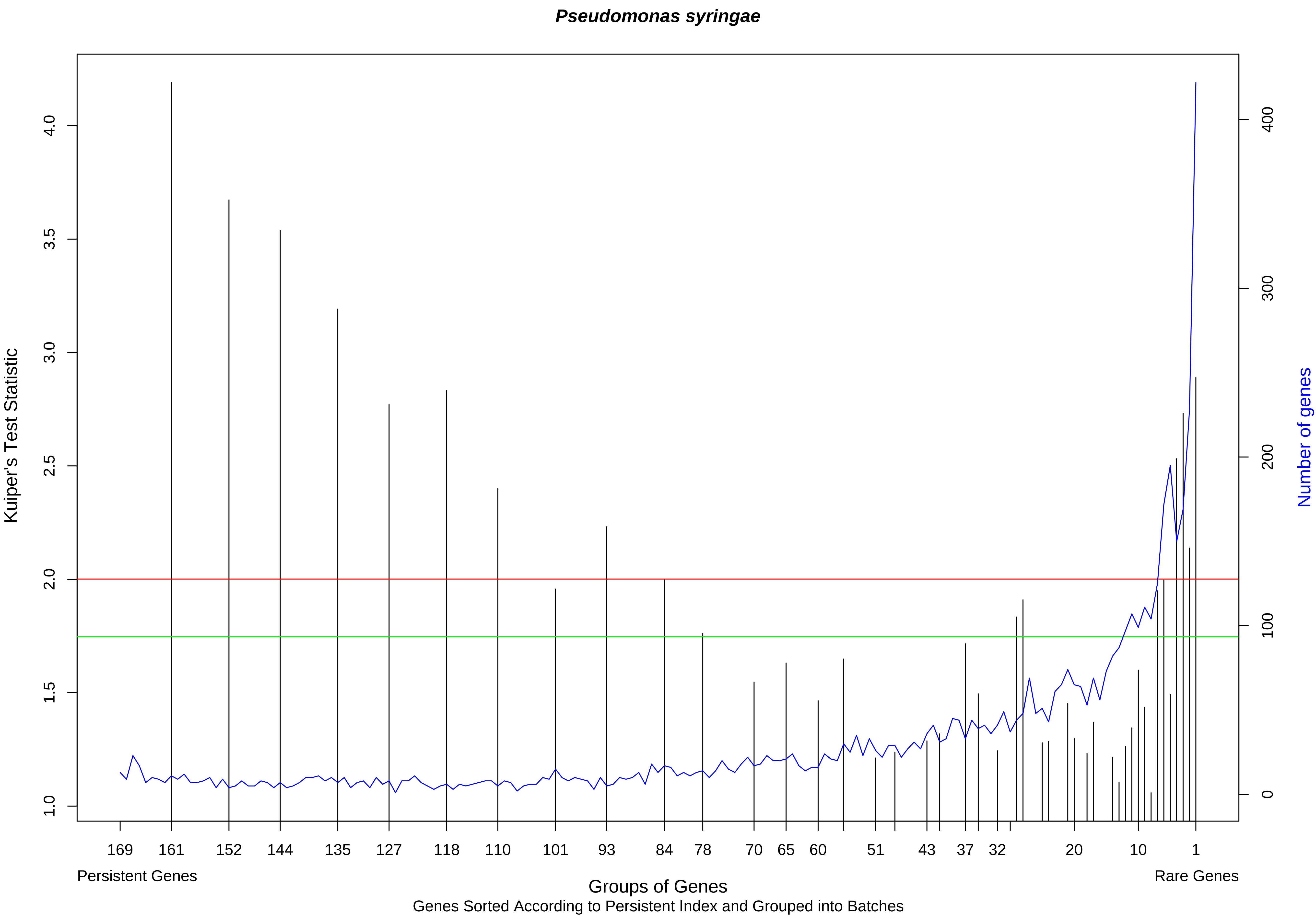

Pseudomonas syringae
Kuiper's Test Statistic
1.0
1.5
2.0
2.5
3.0
3.5
4.0
Number of genes
0
100
200
300
400
169
161
152
144
135
127
118
110
101
93
84
78
70
65
60
51
43
37
32
20
10
1
Persistent Genes
Rare Genes
Groups of Genes
Genes Sorted According to Persistent Index and Grouped into Batches

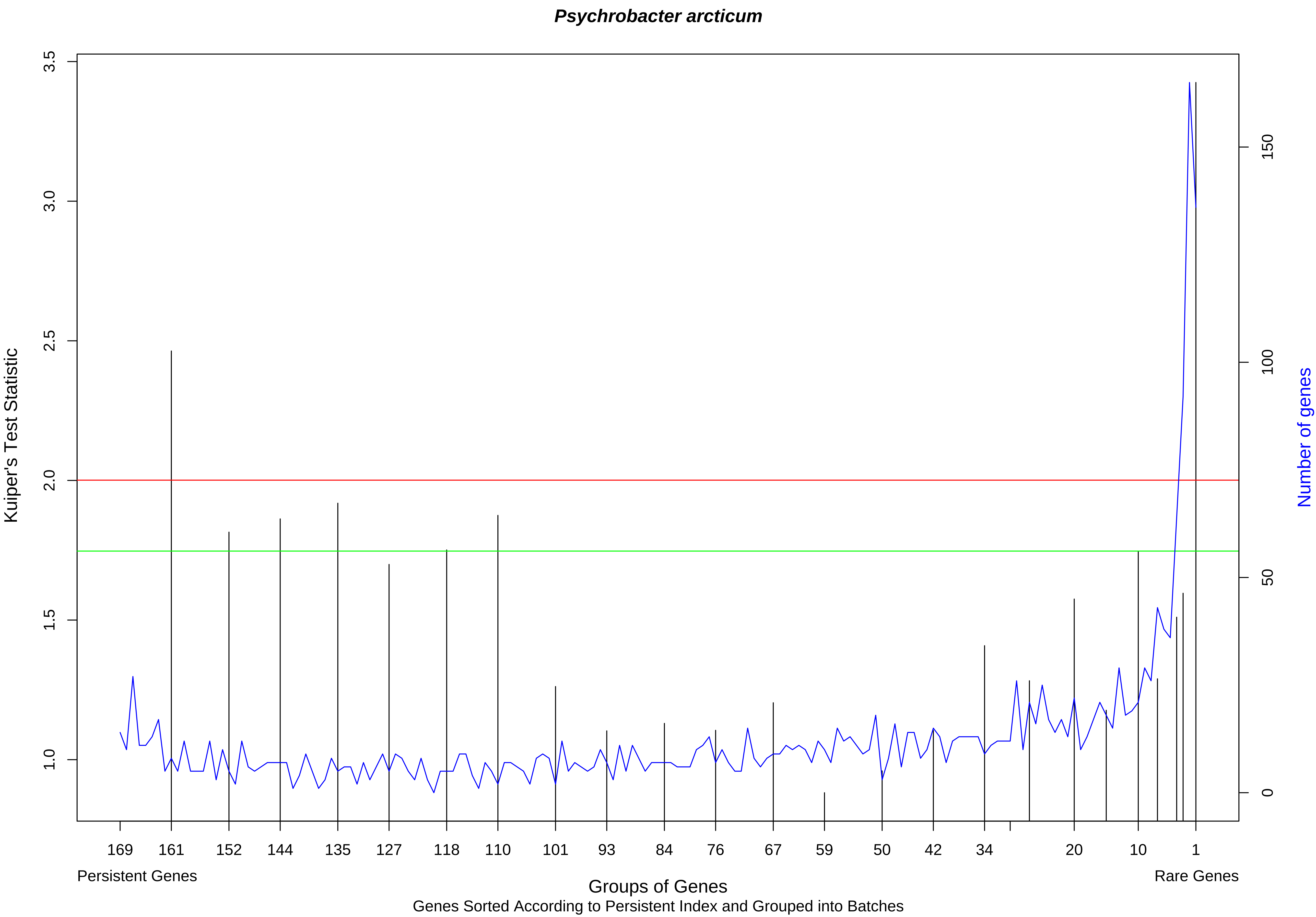

Psychrobacter arcticum
Kuiper's Test Statistic
3.5
3.0
2.5
2.0
1.5
1.0
Number of genes
150
100
50
0
169
161
152
144
135
127
118
110
101
93
84
76
67
59
50
42
34
20
10
1
Persistent Genes
Rare Genes
Groups of Genes
Genes Sorted According to Persistent Index and Grouped into Batches

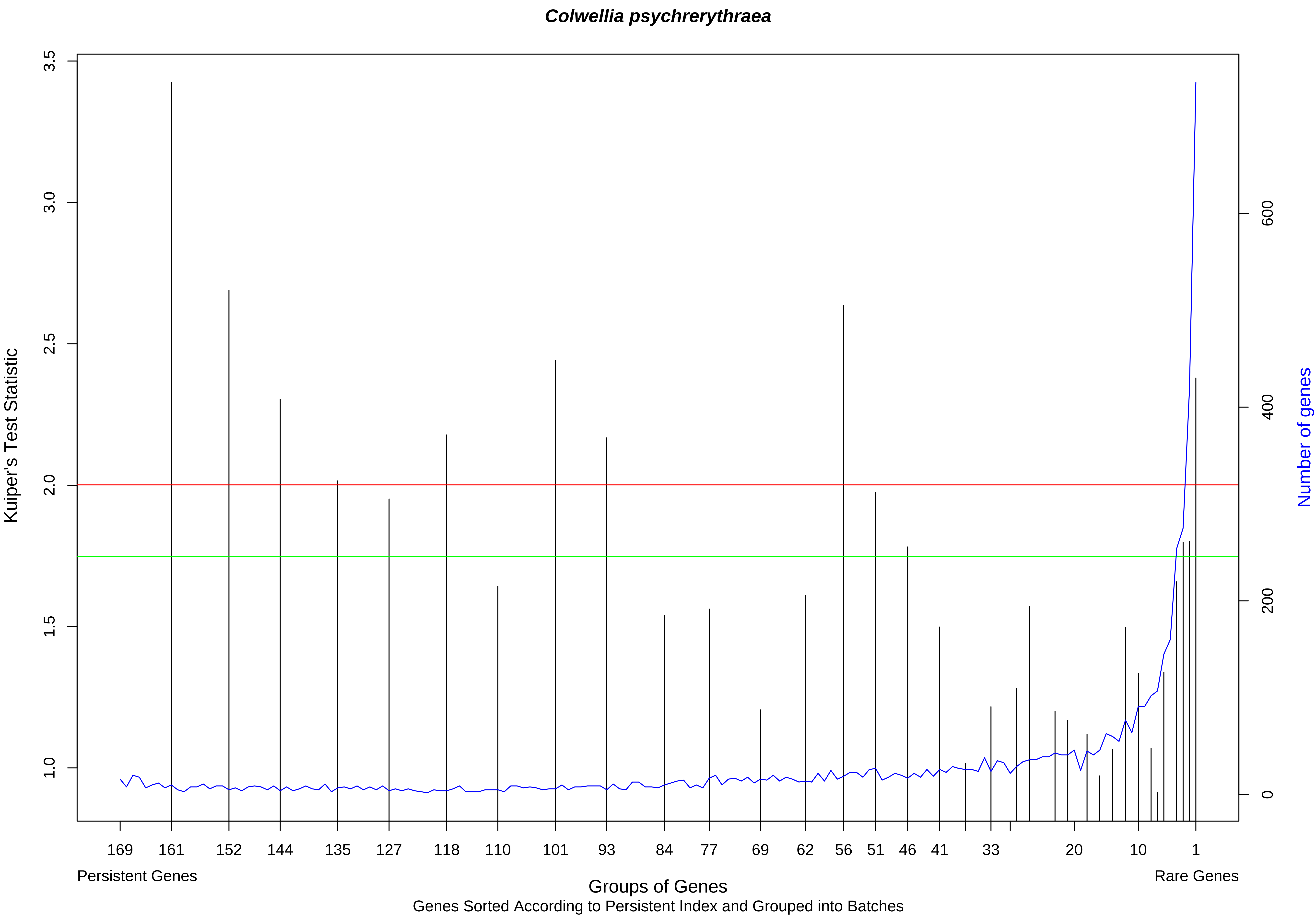

Colwellia psychrerythraea
Kuiper's Test Statistic
3.5
3.0
2.5
2.0
1.5
1.0
Number of genes
600
400
200
0
169
161
152
144
135
127
118
110
101
93
84
77
69
62
56
51
46
41
33
20
10
1
Persistent Genes
Rare Genes
Groups of Genes
Genes Sorted According to Persistent Index and Grouped into Batches

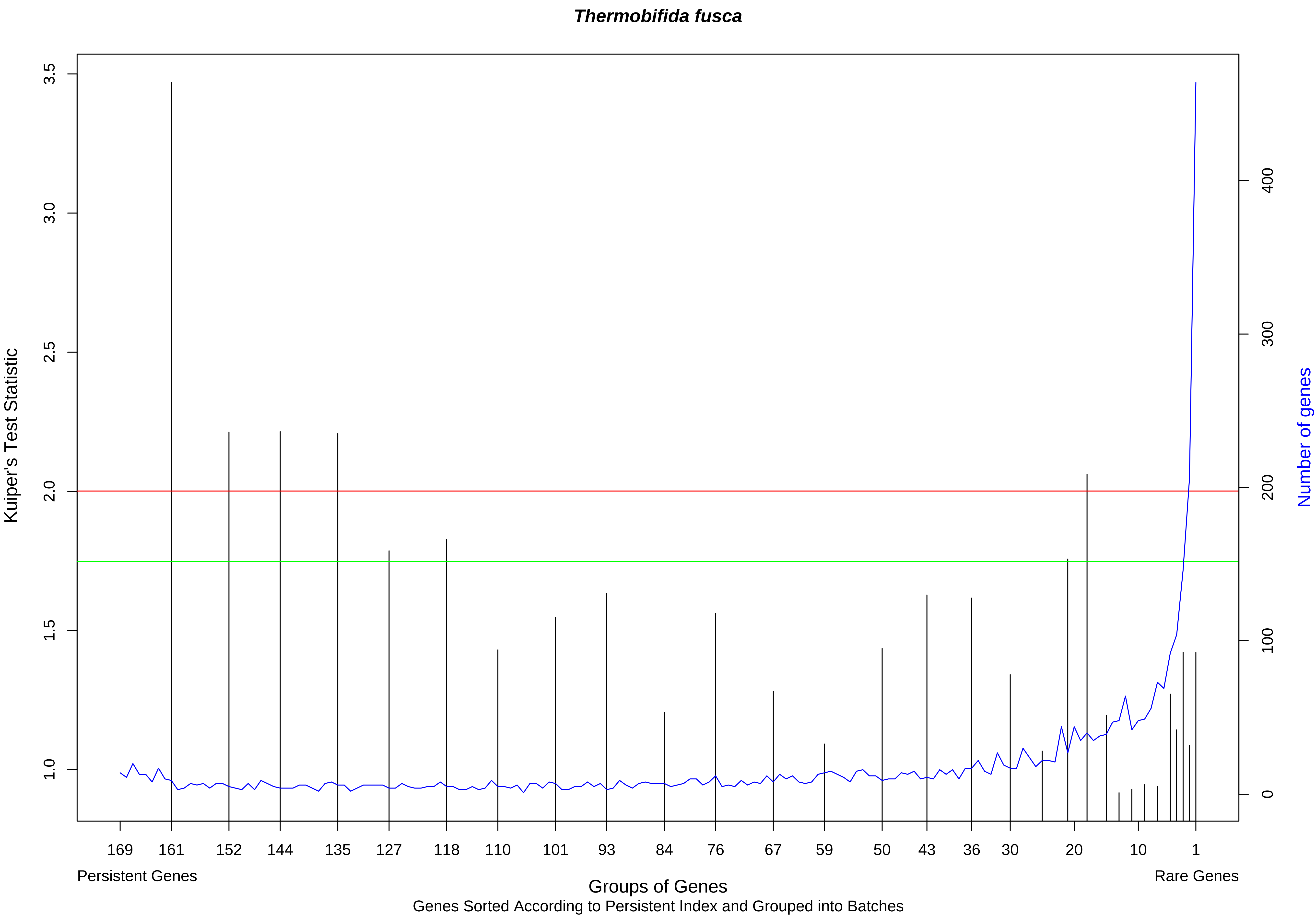

Thermobifida fusca
Kuiper's Test Statistic
3.5
3.0
2.5
2.0
1.5
1.0
Number of genes
400
300
200
100
0
169
161
152
144
135
127
118
110
101
93
84
76
67
59
50
43
36
30
20
10
1
Persistent Genes
Rare Genes
Groups of Genes
Genes Sorted According to Persistent Index and Grouped into Batches

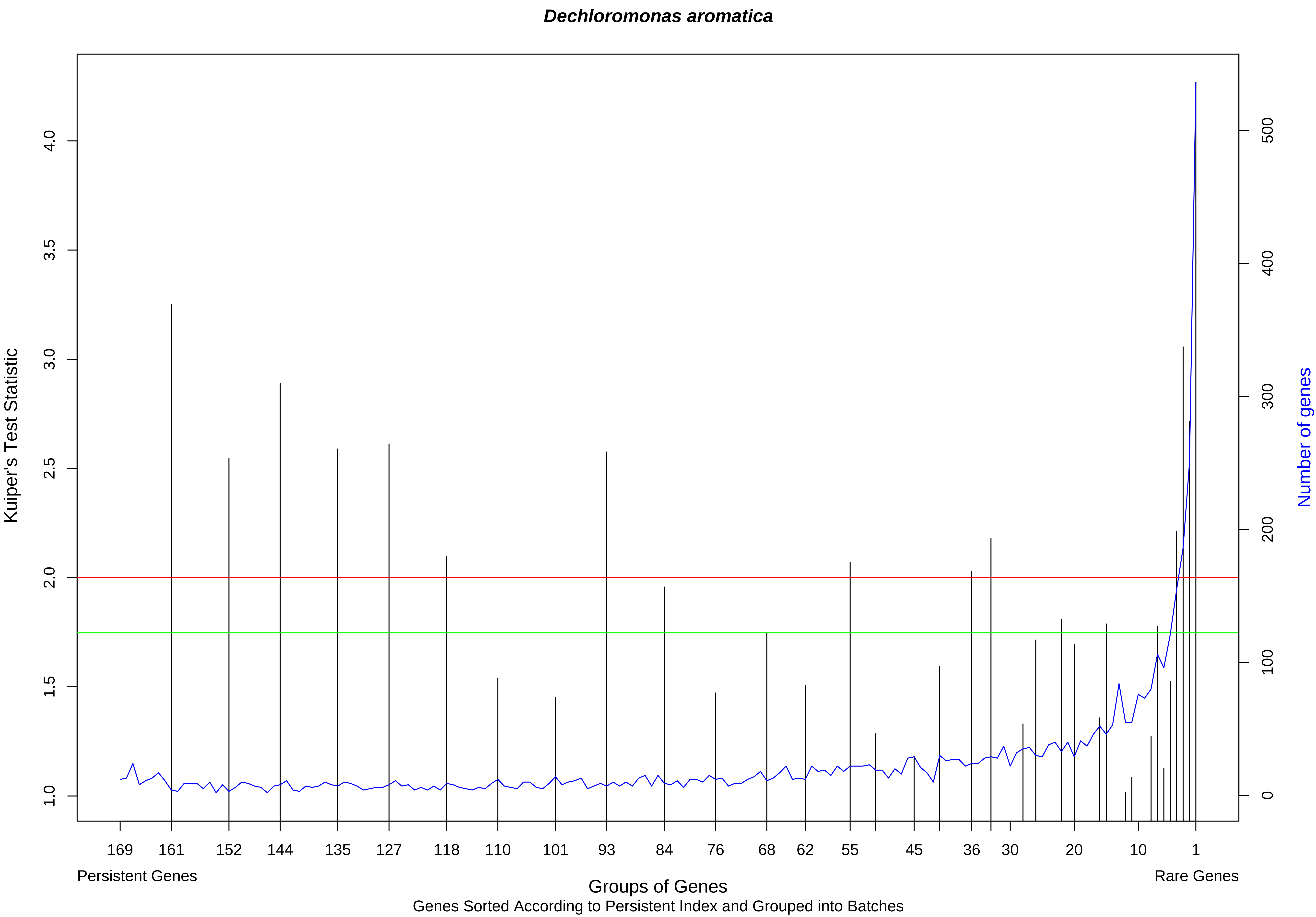

Dechloromonas aromatica
Kuiper's Test Statistic
1.0
1.5
2.0
2.5
3.0
3.5
4.0
Number of genes
0
100
200
300
400
500
169
161
152
144
135
127
118
110
101
93
84
76
68
62
55
45
36
30
20
10
1
Persistent Genes
Rare Genes
Groups of Genes
Genes Sorted According to Persistent Index and Grouped into Batches

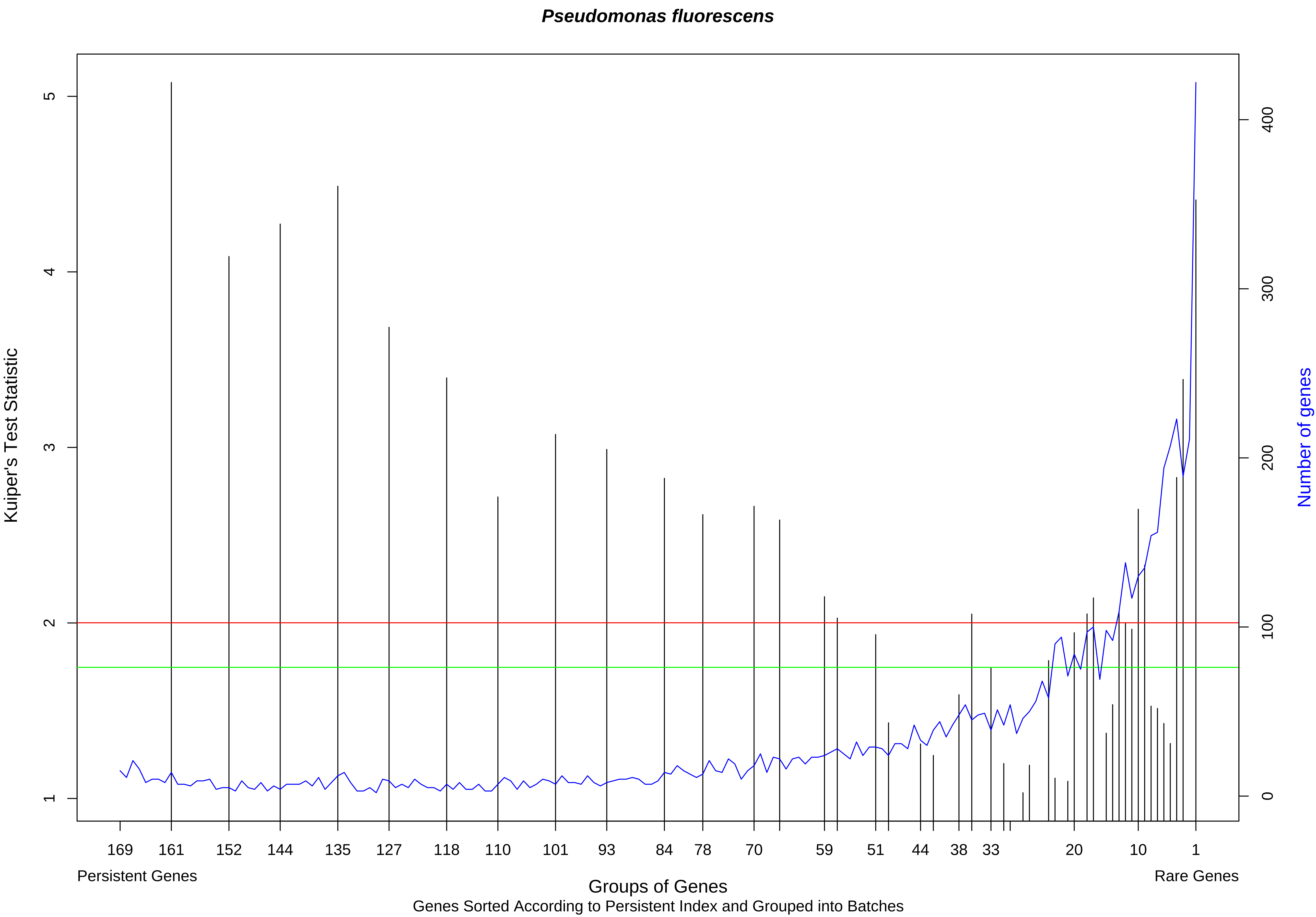

Pseudomonas fluorescens
Kuiper's Test Statistic
Number of genes
1
2
3
4
5
0
100
200
300
400
169
161
152
144
135
127
118
110
101
93
84
78
70
59
51
44
38
33
20
10
1
Persistent Genes
Rare Genes
Groups of Genes
Genes Sorted According to Persistent Index and Grouped into Batches

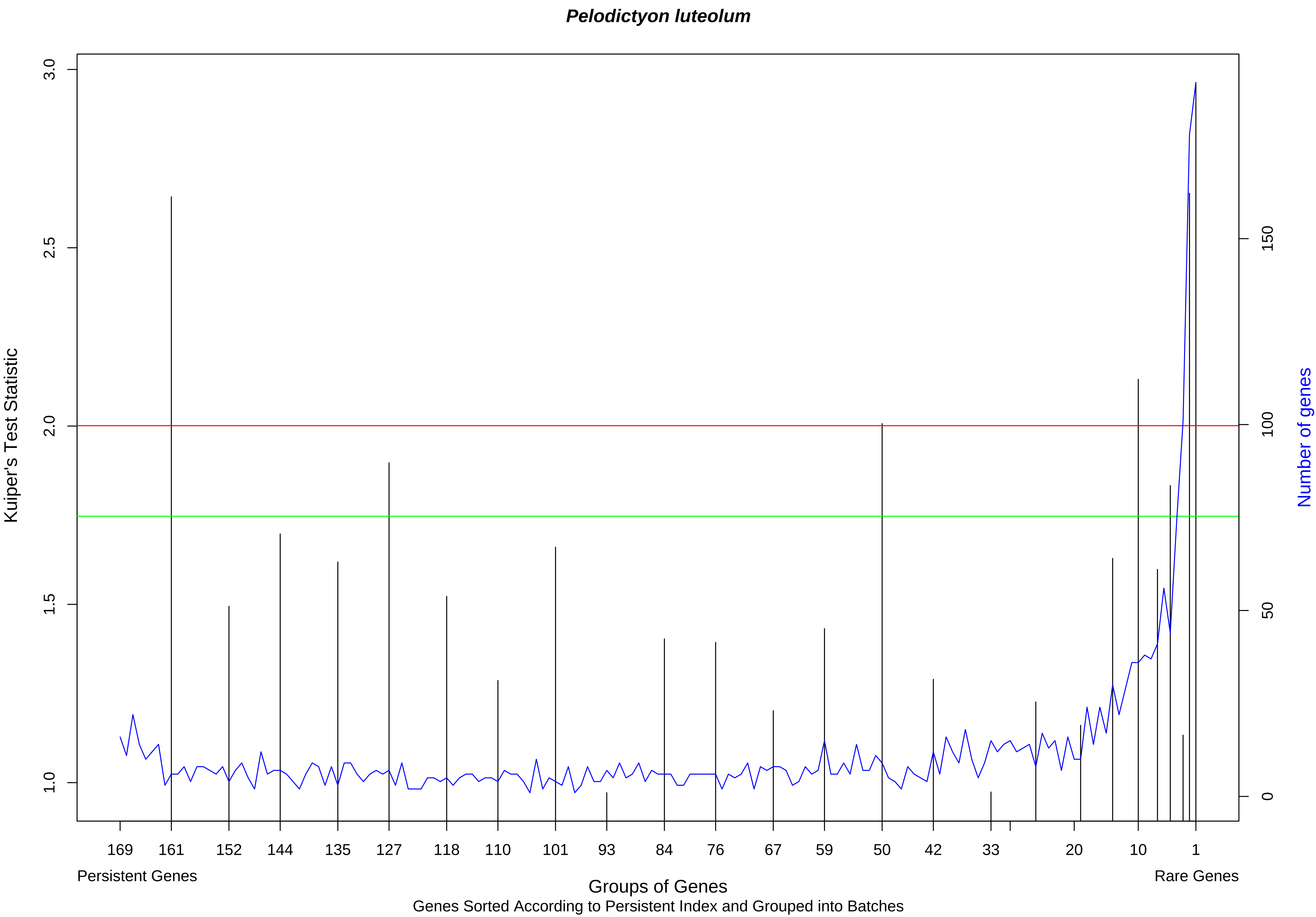

Pelodictyon luteolum
Kuiper's Test Statistic
Number of genes
3.0
2.5
2.0
1.5
1.0
150
100
50
0
169
161
152
144
135
127
118
110
101
93
84
76
67
59
50
42
33
20
10
1
Persistent Genes
Rare Genes
Groups of Genes
Genes Sorted According to Persistent Index and Grouped into Batches

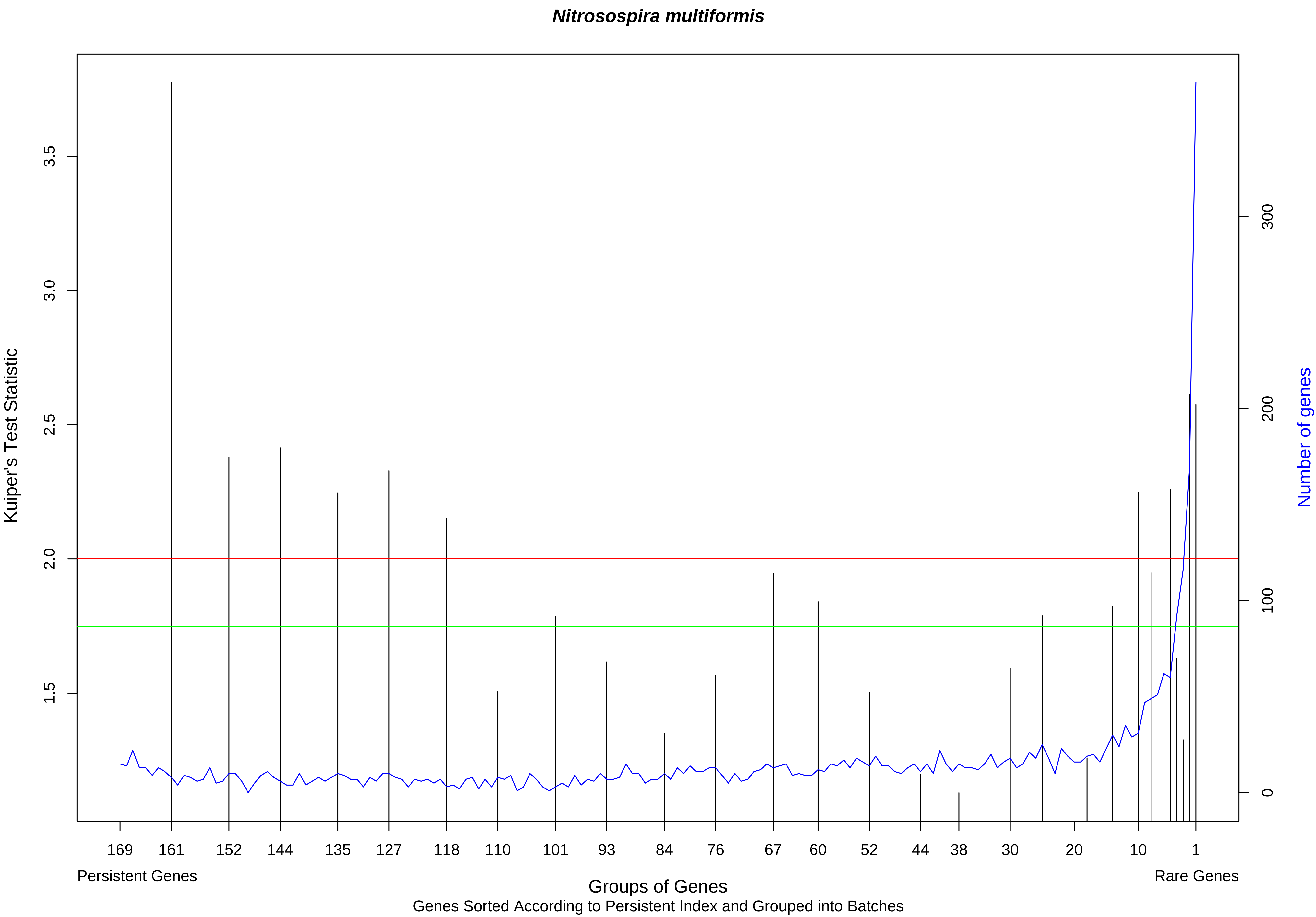

Nitrosospira multiformis
Kuiper's Test Statistic
3.5
3.0
2.5
2.0
1.5
Number of genes
300
200
100
0
169
161
152
144
135
127
118
110
101
93
84
76
67
60
52
44
38
30
20
10
1
Persistent Genes
Rare Genes
Groups of Genes
Genes Sorted According to Persistent Index and Grouped into Batches

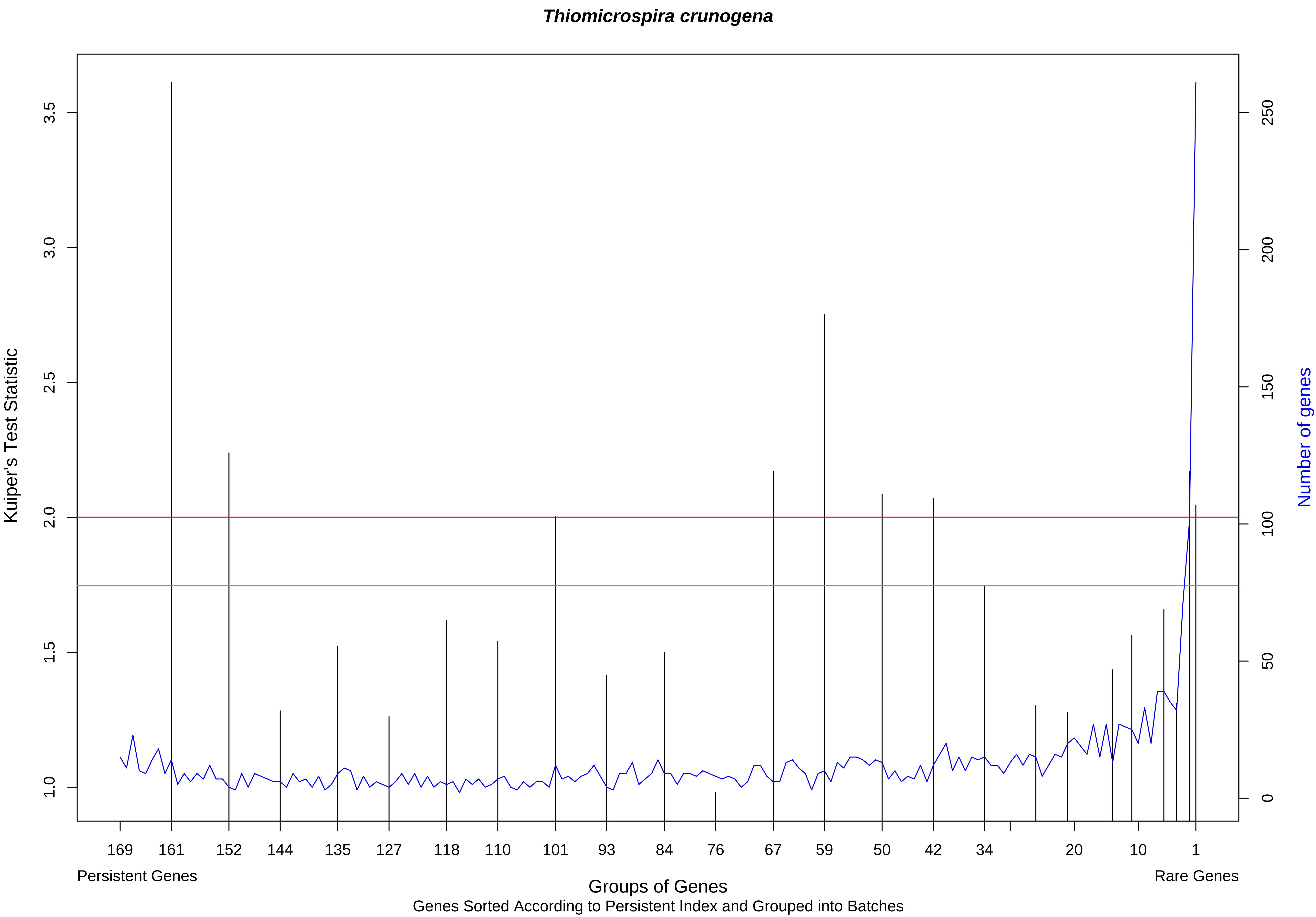

Thiomicrospira crunogena
Kuiper's Test Statistic
3.5
3.0
2.5
2.0
1.5
1.0
Number of genes
250
200
150
100
50
0
169
161
152
144
135
127
118
110
101
93
84
76
67
59
50
42
34
20
10
1
Persistent Genes
Rare Genes
Groups of Genes
Genes Sorted According to Persistent Index and Grouped into Batches

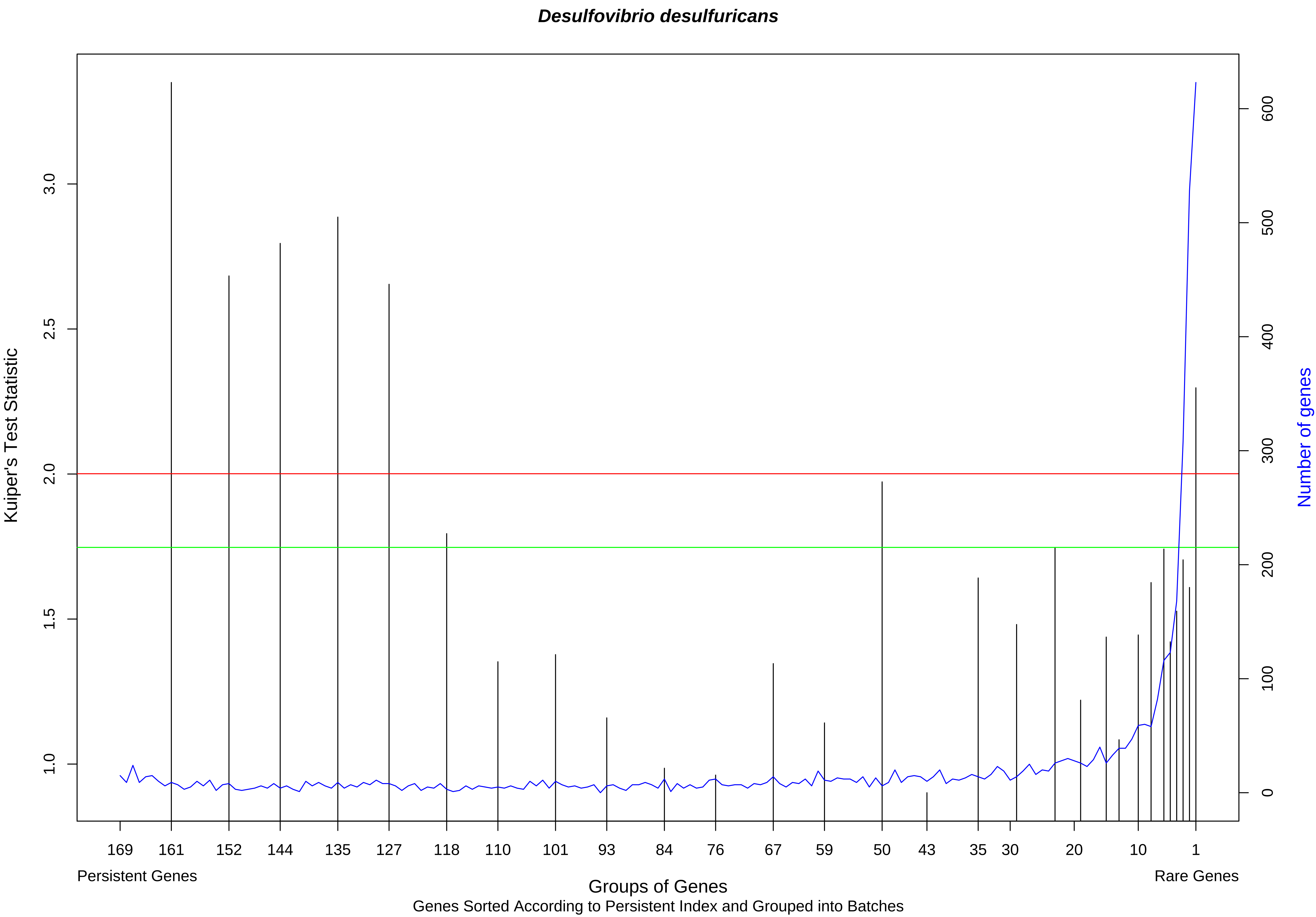

Desulfovibrio desulfuricans
Kuiper's Test Statistic
1.0
1.5
2.0
2.5
3.0
Number of genes
0
100
200
300
400
500
600
169
161
152
144
135
127
118
110
101
93
84
76
67
59
50
43
35
30
20
10
1
Persistent Genes
Rare Genes
Groups of Genes
Genes Sorted According to Persistent Index and Grouped into Batches

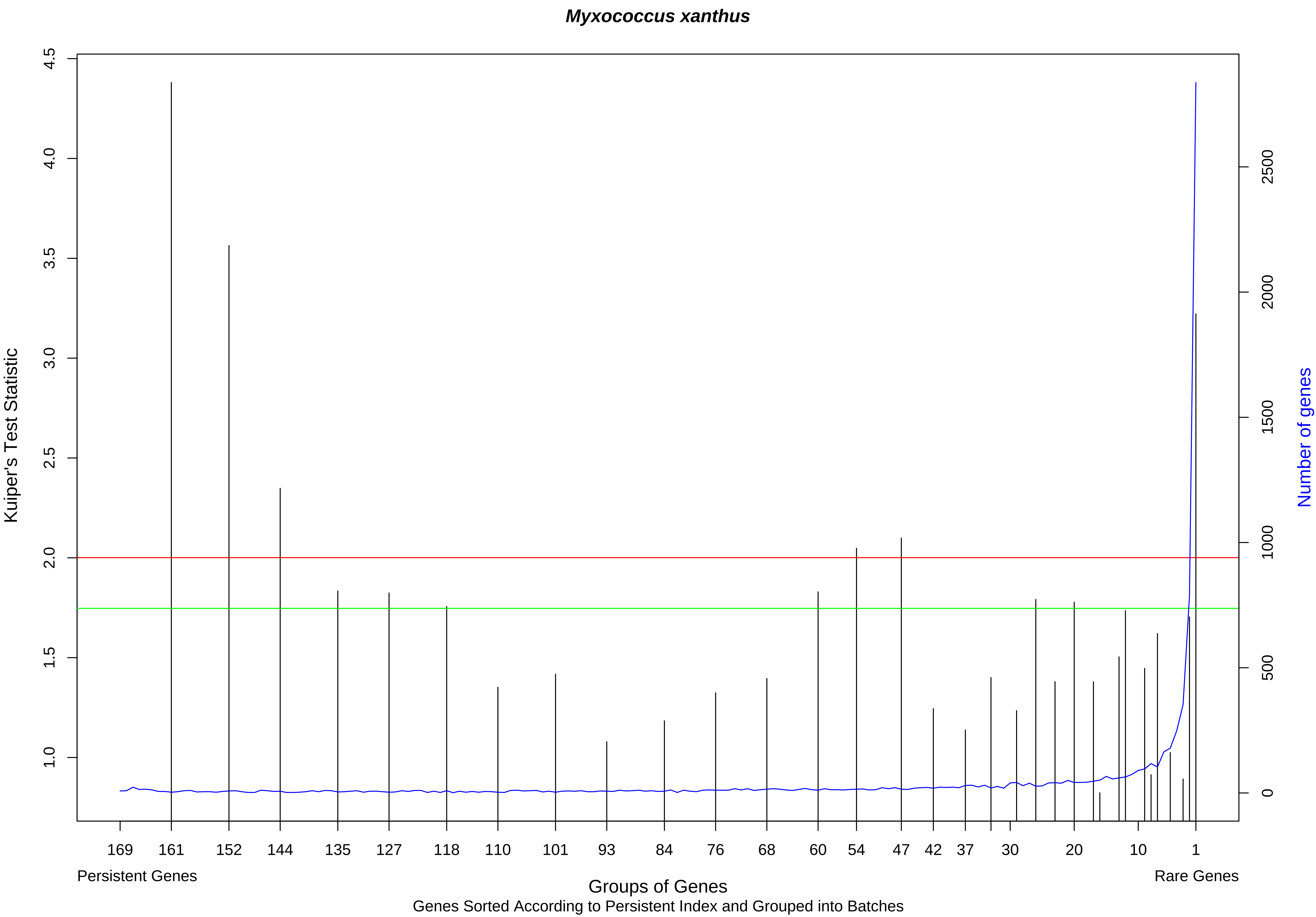
Myxococcus xanthus
Kuiper's Test Statistic
4.5
4.0
3.5
3.0
2.5
2.0
1.5
1.0
Number of genes
2500
2000
1500
1000
500
0
169 161 152 144 135 127 118 110 101 93 84 76 68 60 54 47 42 37 30 20 10 1
Persistent Genes
Rare Genes
Groups of Genes
Genes Sorted According to Persistent Index and Grouped into Batches

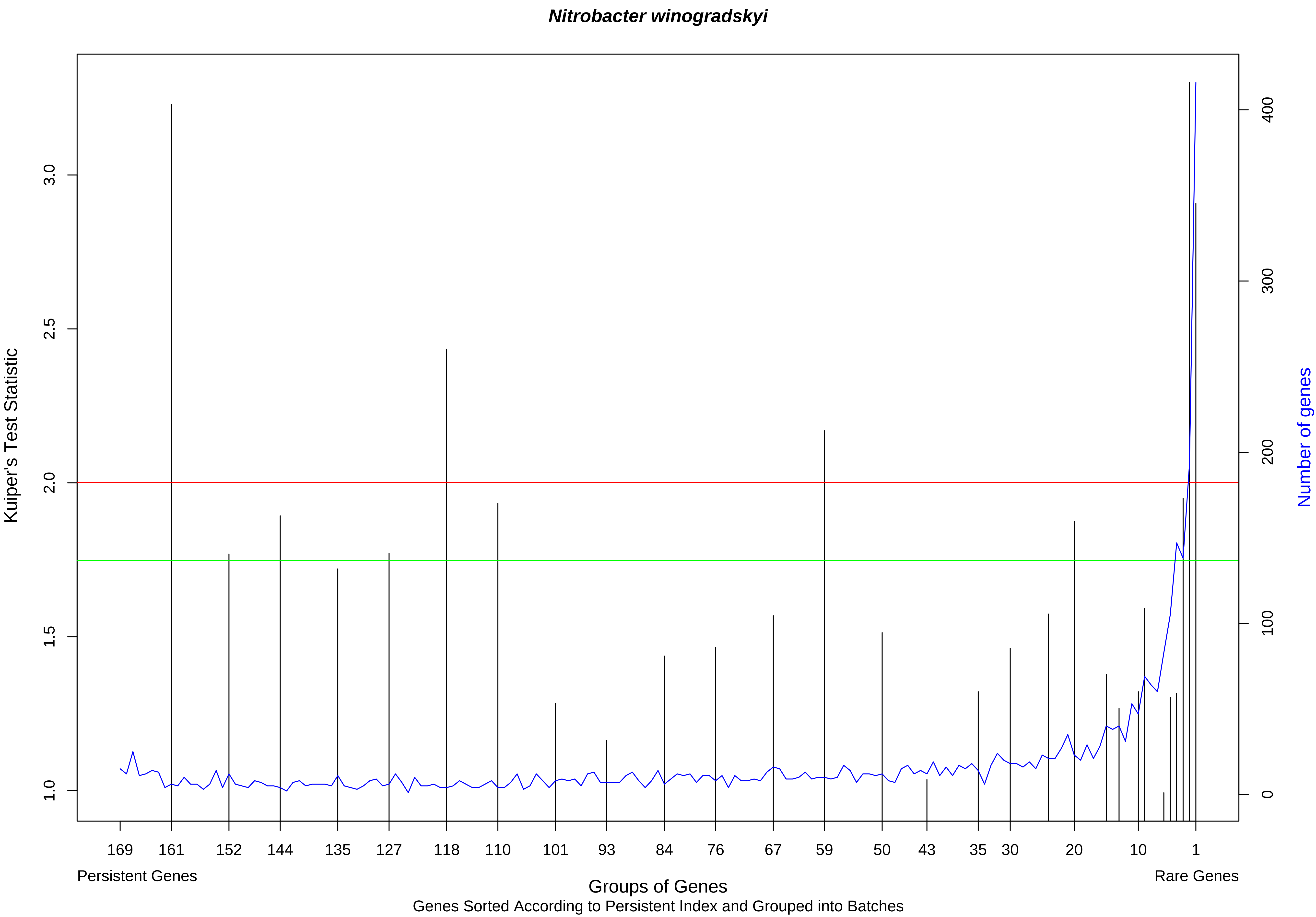

Nitrobacter winogradskyi
Kuiper's Test Statistic
1.0
1.5
2.0
2.5
3.0
Number of genes
0
100
200
300
400
169
161
152
144
135
127
118
110
101
93
84
76
67
59
50
43
35
30
20
10
1
Persistent Genes
Rare Genes
Groups of Genes
Genes Sorted According to Persistent Index and Grouped into Batches

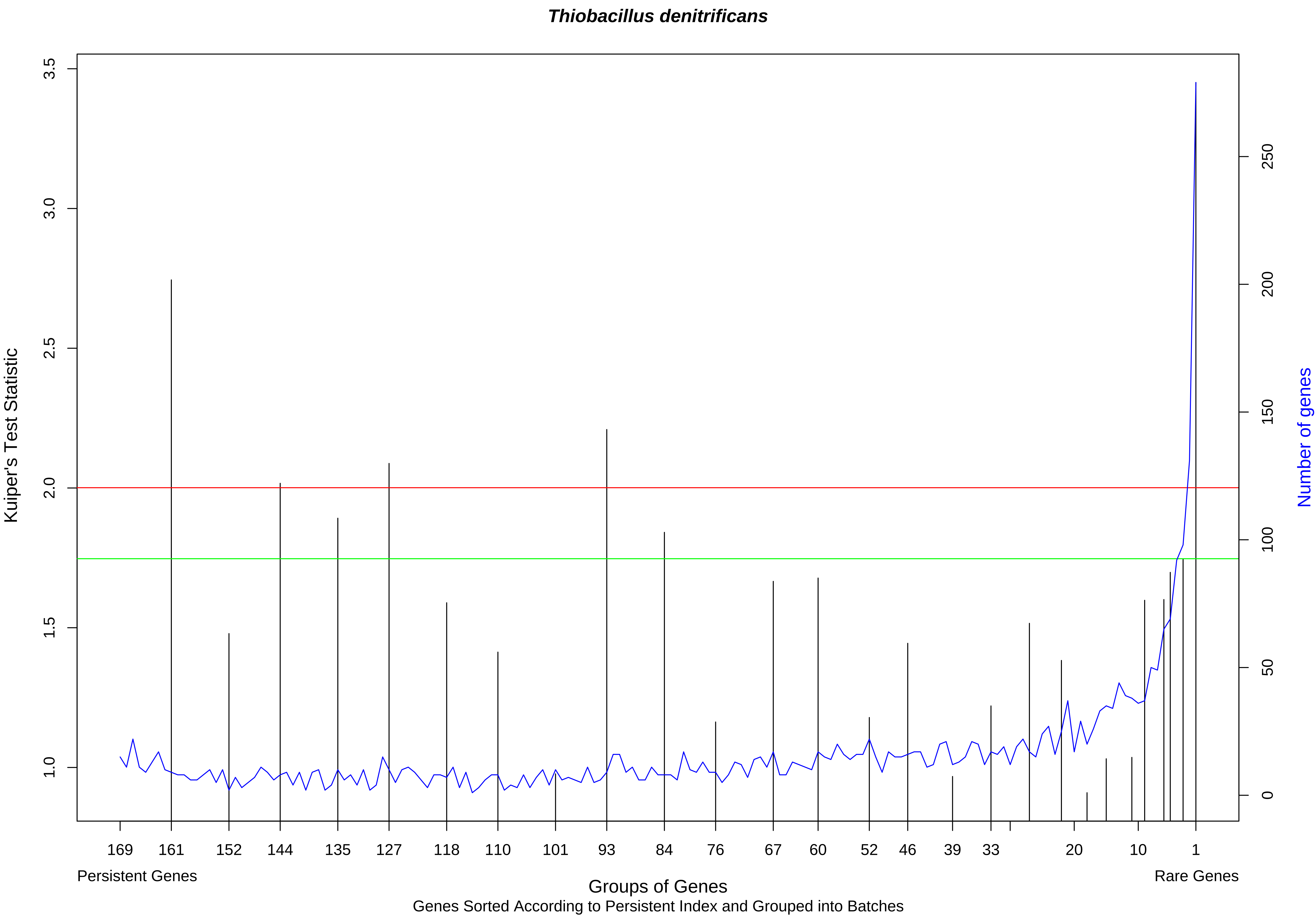
Thiobacillus denitrificans
Kuiper's Test Statistic
Number of genes
3.5
3.0
2.5
2.0
1.5
1.0
250
200
150
100
50
0
169
161
152
144
135
127
118
110
101
93
84
76
67
60
52
46
39
33
20
10
1
Persistent Genes
Rare Genes
Groups of Genes
Genes Sorted According to Persistent Index and Grouped into Batches

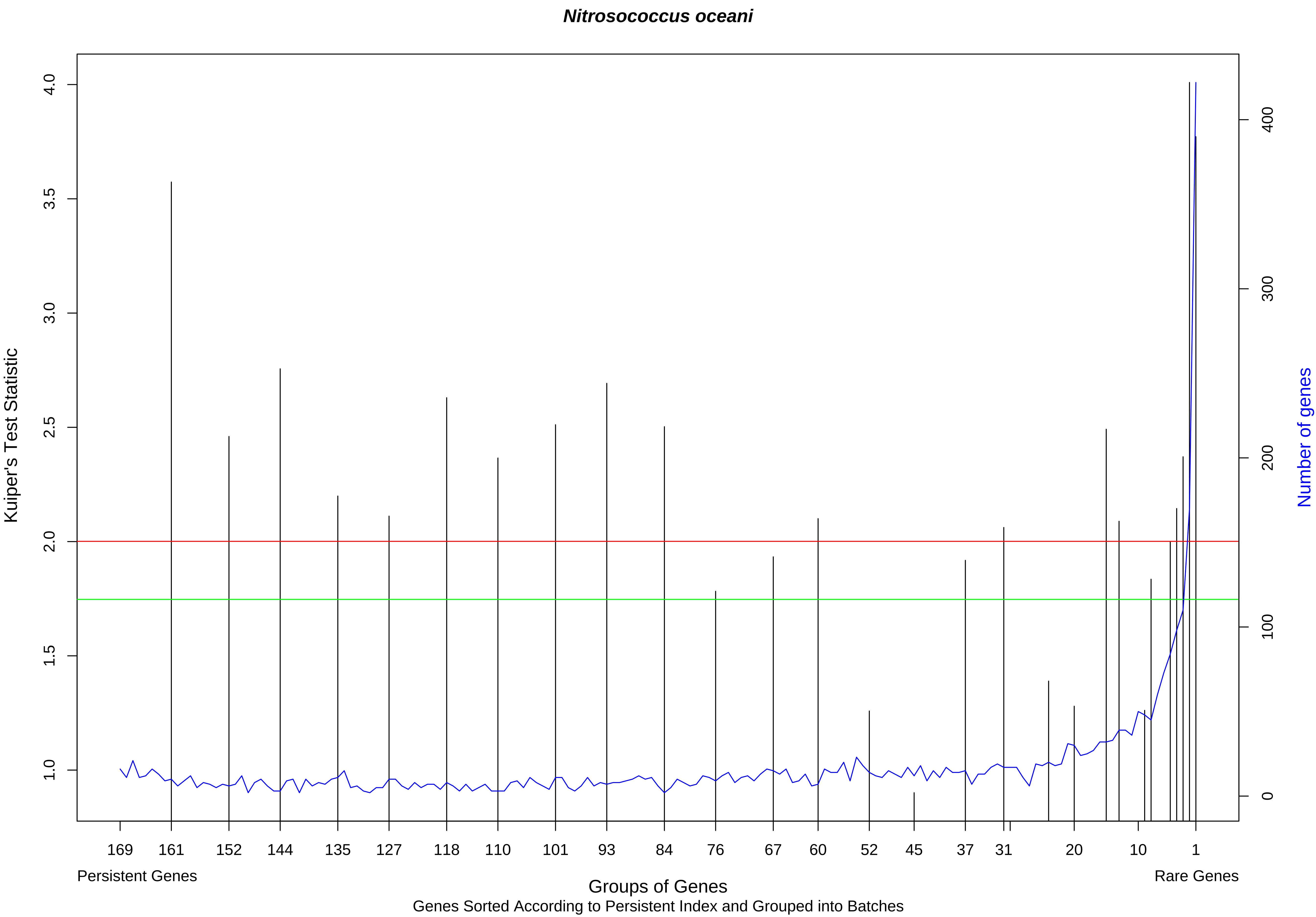
Nitrosococcus oceani
Kuiper's Test Statistic
1.0
1.5
2.0
2.5
3.0
3.5
4.0
Number of genes
0
100
200
300
400
169
161
152
144
135
127
118
110
101
93
84
76
67
60
52
45
37
31
20
10
1
Persistent Genes
Rare Genes
Groups of Genes
Genes Sorted According to Persistent Index and Grouped into Batches

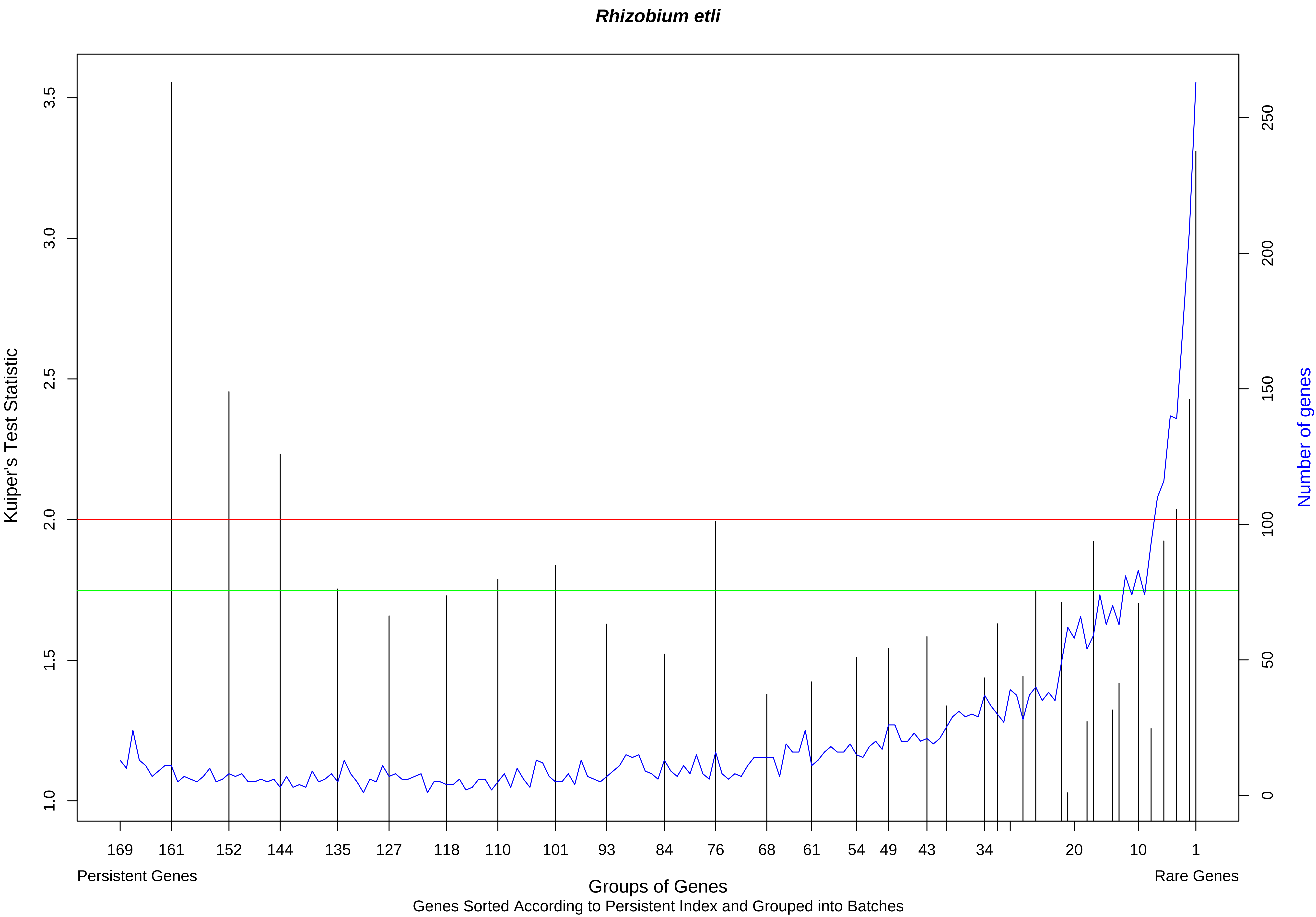

Rhizobium etli
Kuiper's Test Statistic
Number of genes
1.0
1.5
2.0
2.5
3.0
3.5
0
50
100
150
200
250
169
161
152
144
135
127
118
110
101
93
84
76
68
61
54
49
43
34
20
10
1
Persistent Genes
Rare Genes
Groups of Genes
Genes Sorted According to Persistent Index and Grouped into Batches

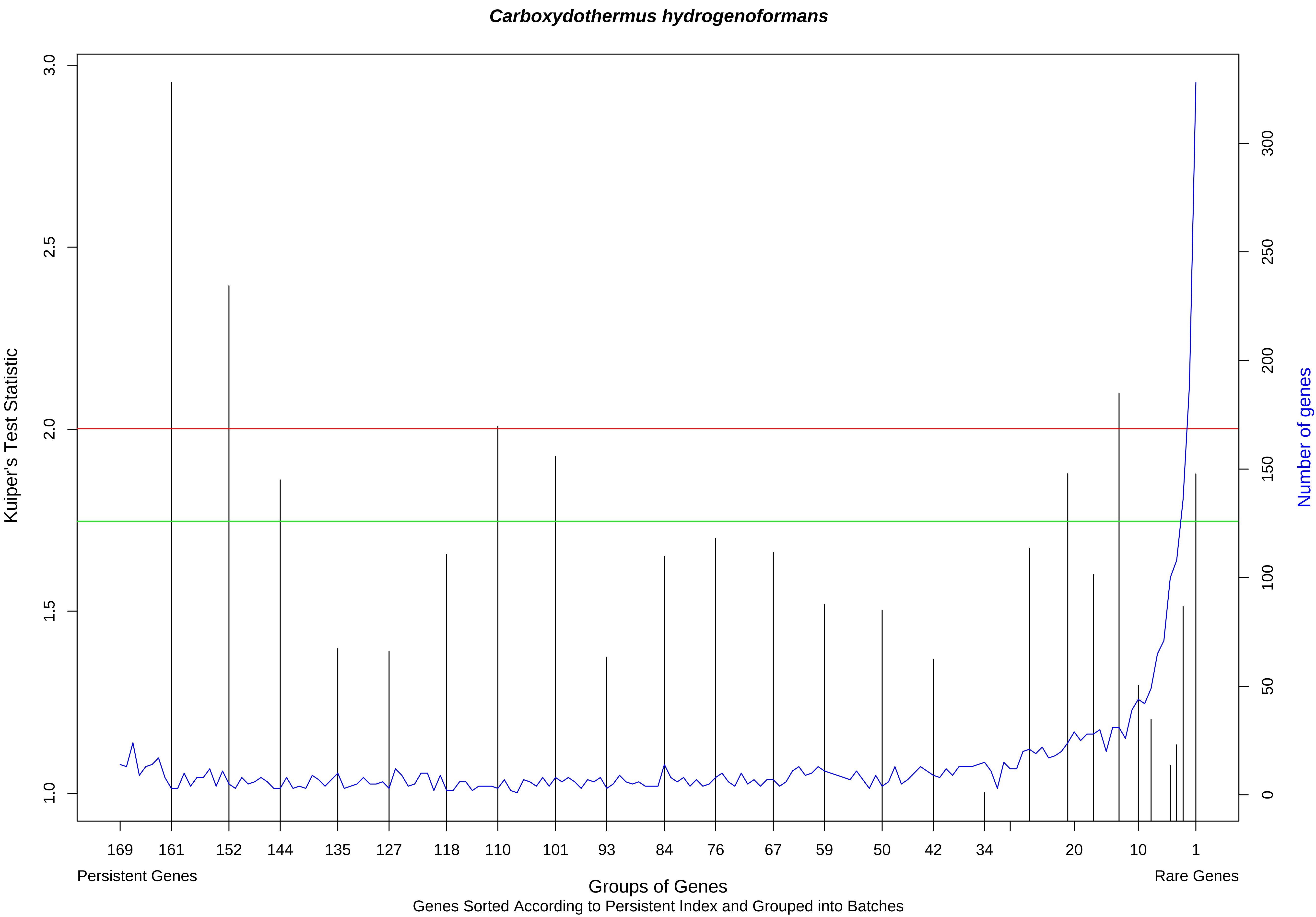
Carboxydothermus hydrogenoformans
Kuiper's Test Statistic
3.0
2.5
2.0
1.5
1.0
Number of genes
300
250
200
150
100
50
0
169
161
152
144
135
127
118
110
101
93
84
76
67
59
50
42
34
20
10
1
Persistent Genes
Rare Genes
Groups of Genes
Genes Sorted According to Persistent Index and Grouped into Batches

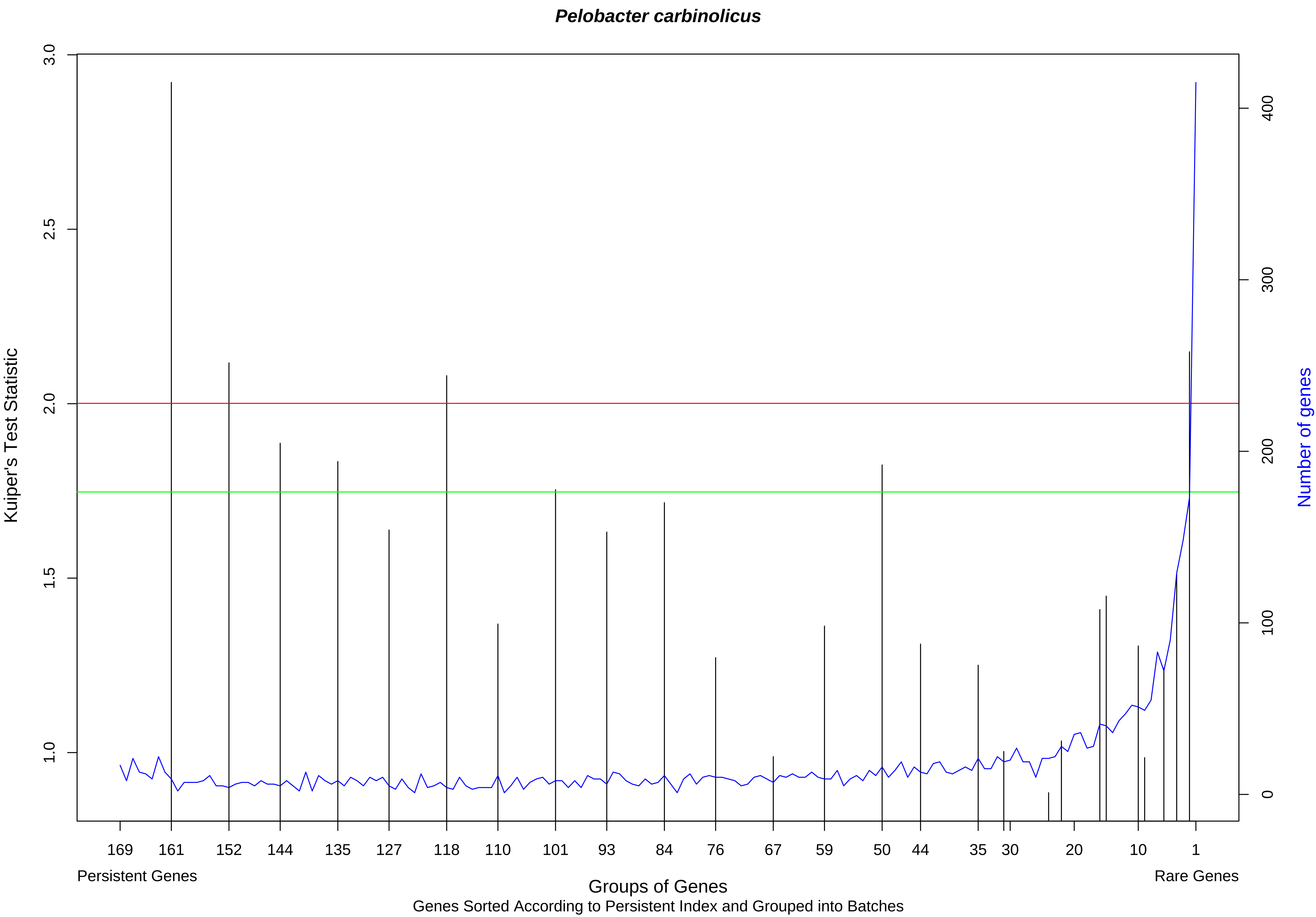
Pelobacter carbinolicus
Kuiper's Test Statistic
3.0
2.5
2.0
1.5
1.0
Number of genes
400
300
200
100
0
169
161
152
144
135
127
118
110
101
93
84
76
67
59
50
44
35
30
20
10
1
Persistent Genes
Rare Genes
Groups of Genes
Genes Sorted According to Persistent Index and Grouped into Batches

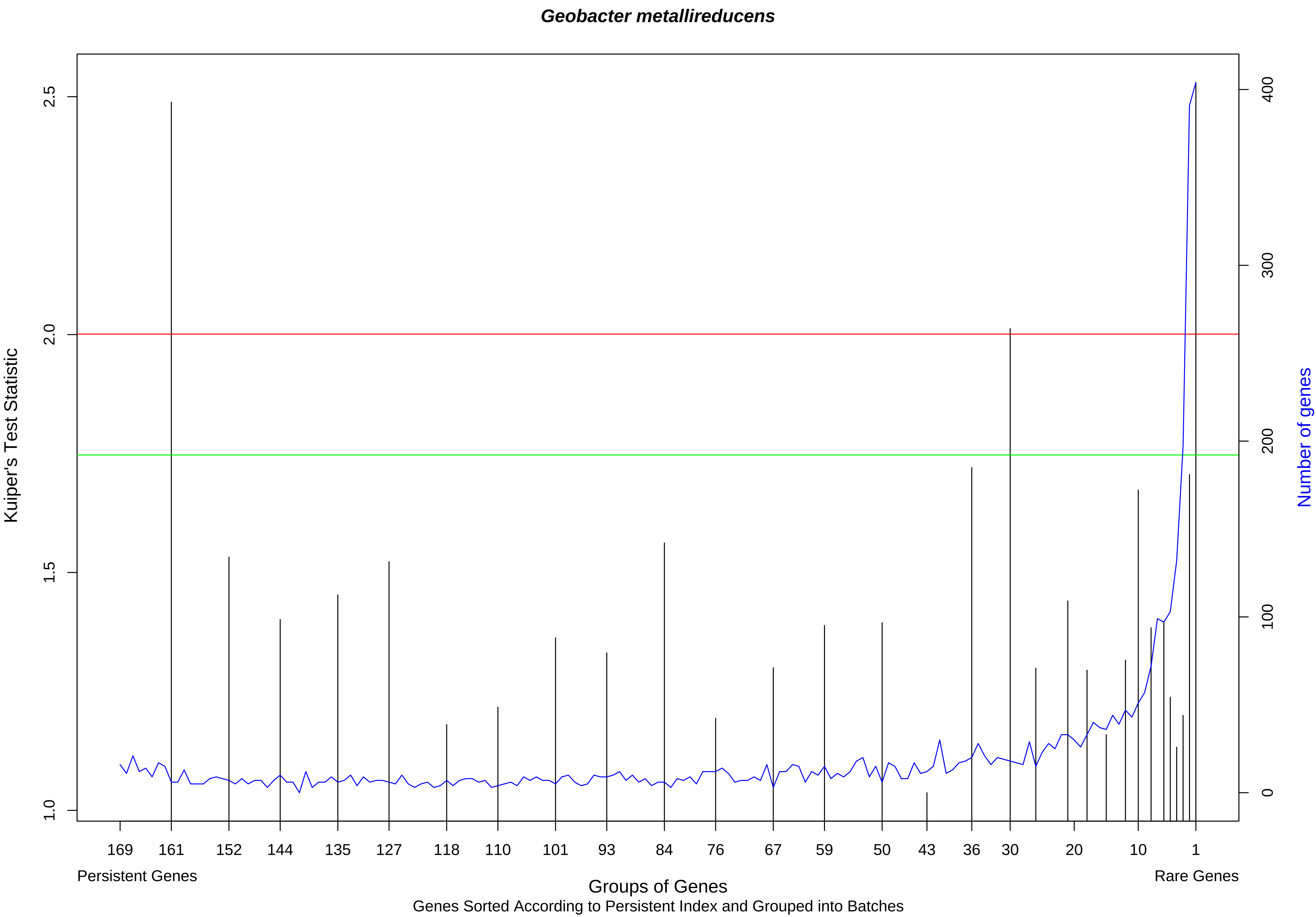

Geobacter metallireducens
Kuiper's Test Statistic
1.0
1.5
2.0
2.5
Number of genes
0
100
200
300
400
169
161
152
144
135
127
118
110
101
93
84
76
67
59
50
43
36
30
20
10
1
Persistent Genes
Rare Genes
Groups of Genes
Genes Sorted According to Persistent Index and Grouped into Batches

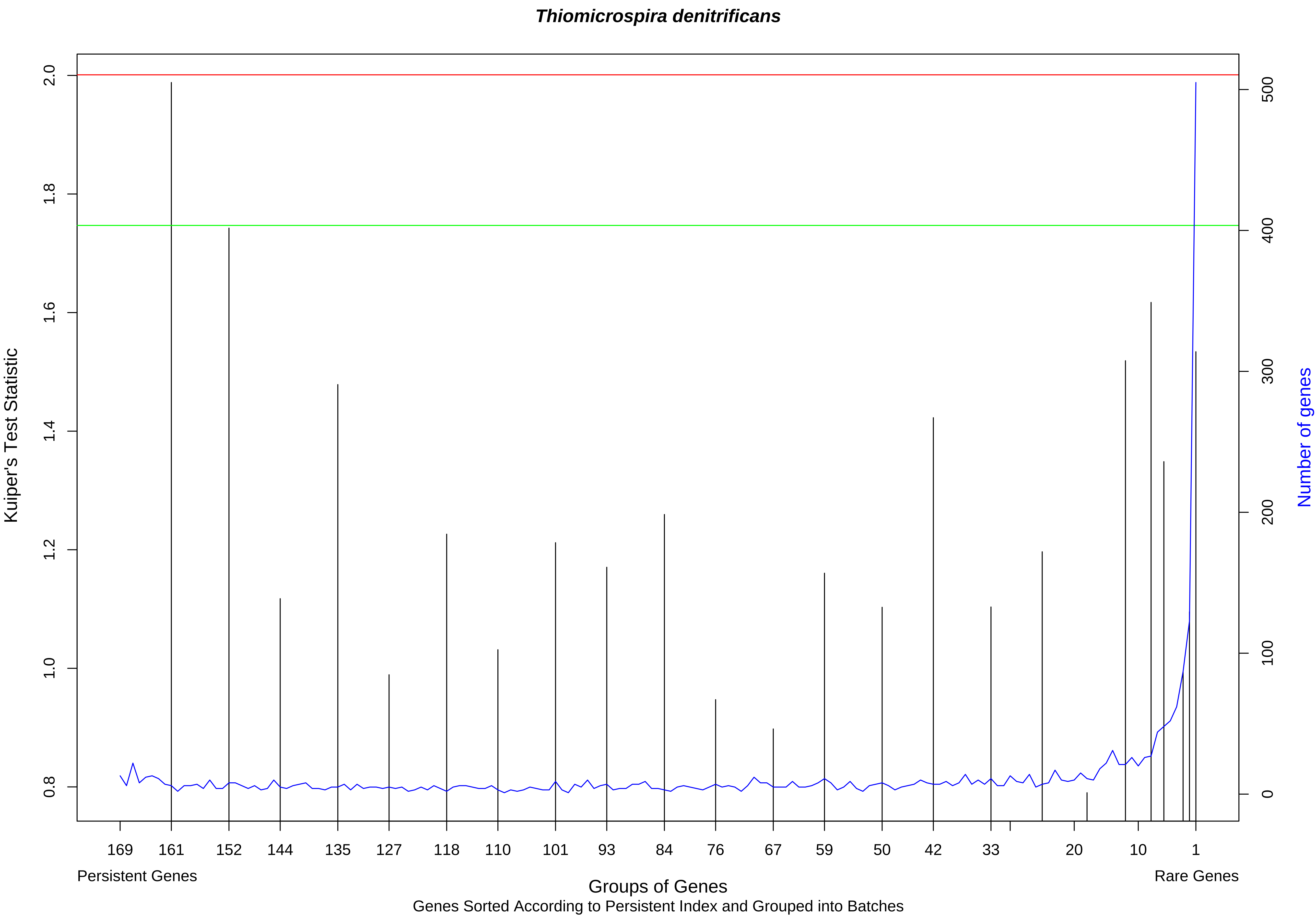

Thiomicrospira denitrificans
Kuiper's Test Statistic
Number of genes
2.0
1.8
1.6
1.4
1.2
1.0
0.8
500
400
300
200
100
0
169
161
152
144
135
127
118
110
101
93
84
76
67
59
50
42
33
20
10
1
Persistent Genes
Rare Genes
Groups of Genes
Genes Sorted According to Persistent Index and Grouped into Batches

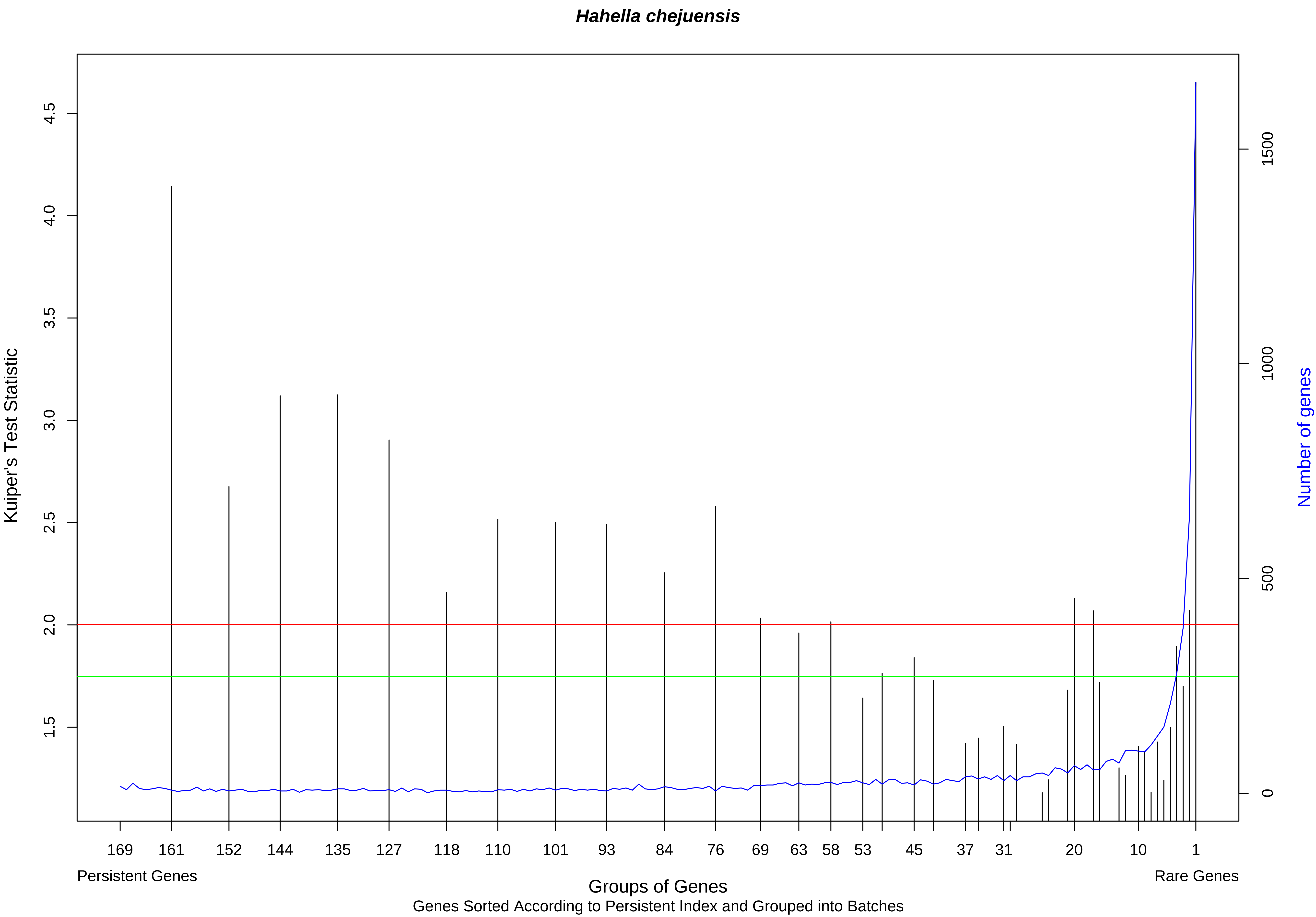
Hahella chejuensis
Kuiper's Test Statistic
Number of genes
4.5
4.0
3.5
3.0
2.5
2.0
1.5
1500
1000
500
0
169
161
152
144
135
127
118
110
101
93
84
76
69
63
58
53
45
37
31
20
10
1
Persistent Genes
Rare Genes
Groups of Genes
Genes Sorted According to Persistent Index and Grouped into Batches

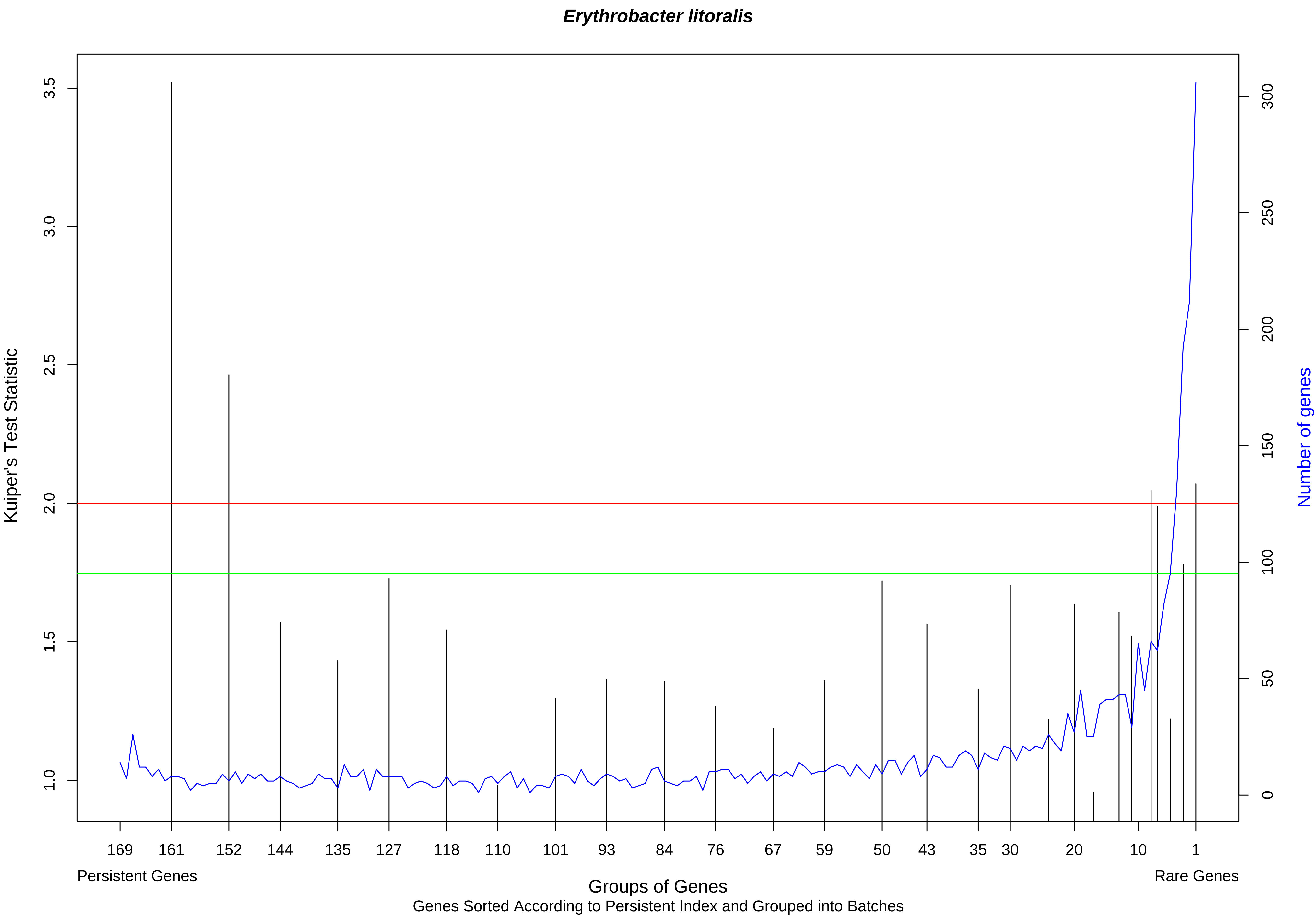

Erythrobacter litoralis
Kuiper's Test Statistic
3.5
3.0
2.5
2.0
1.5
1.0
Number of genes
300
250
200
150
100
50
0
169
161
152
144
135
127
118
110
101
93
84
76
67
59
50
43
35
30
20
10
1
Persistent Genes
Rare Genes
Groups of Genes
Genes Sorted According to Persistent Index and Grouped into Batches

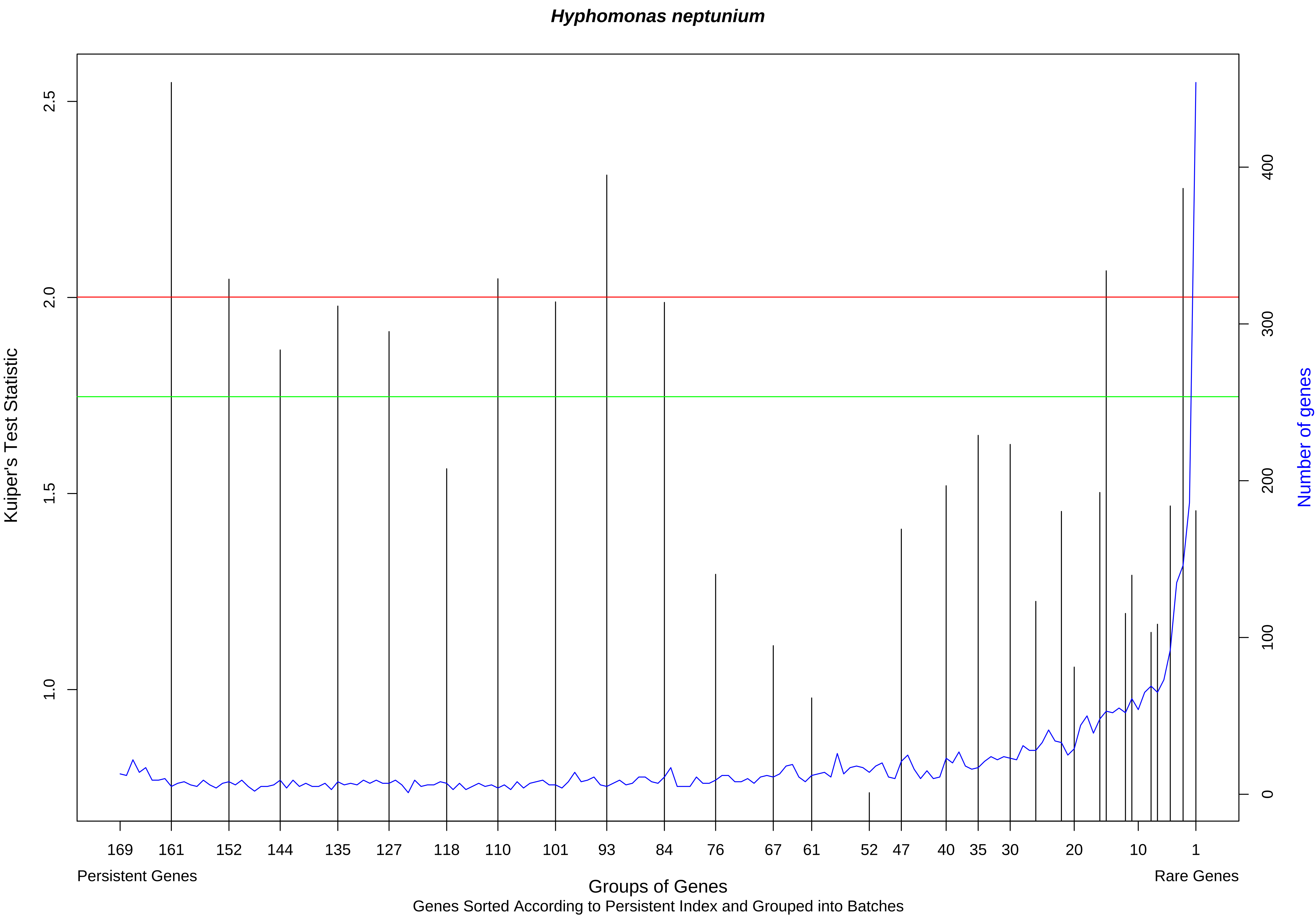

Hyphomonas neptunium
Kuiper's Test Statistic
1.0
1.5
2.0
2.5
Number of genes
0
100
200
300
400
169
161
152
144
135
127
118
110
101
93
84
76
67
61
52
47
40
35
30
20
10
1
Persistent Genes
Rare Genes
Groups of Genes
Genes Sorted According to Persistent Index and Grouped into Batches

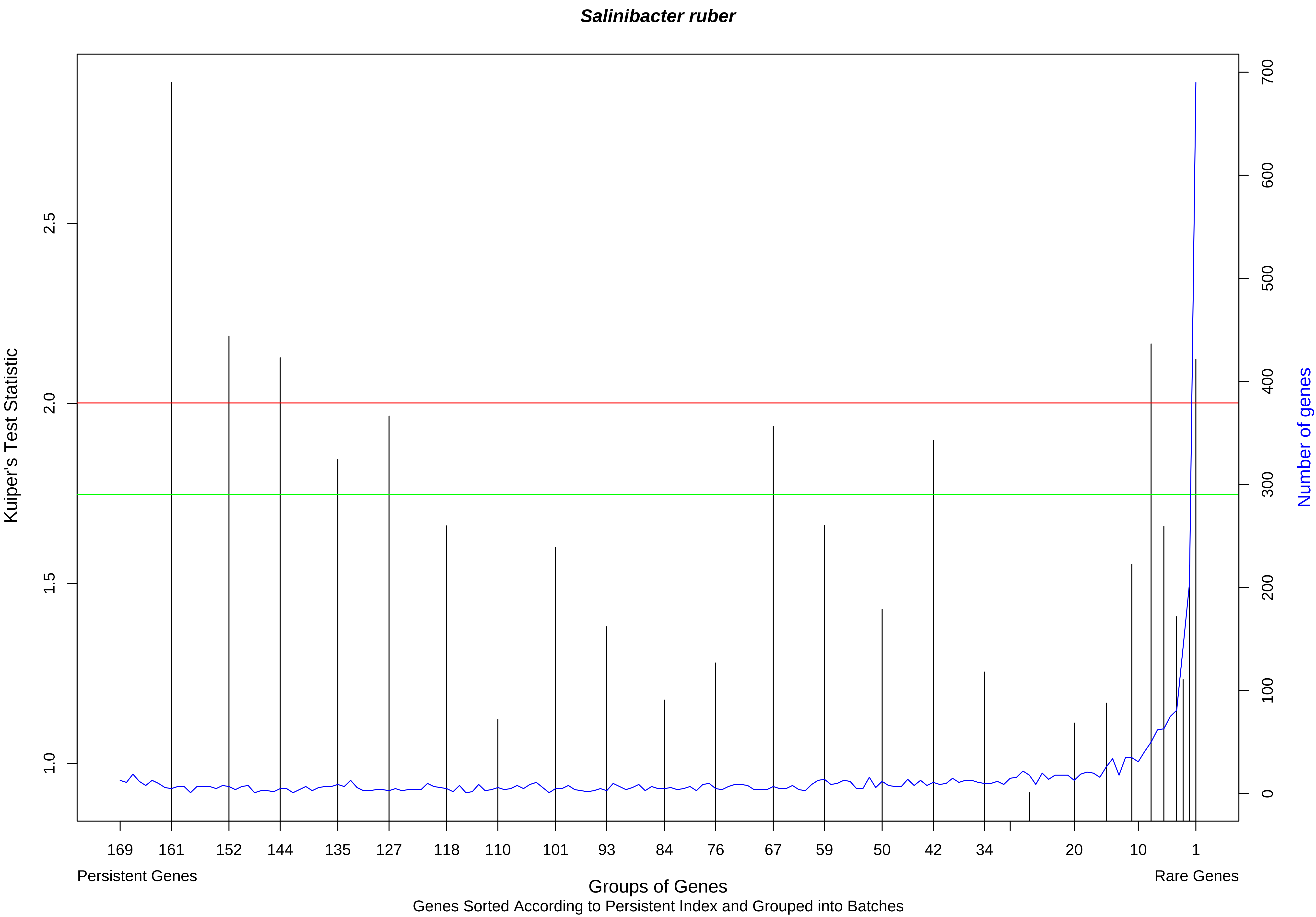

Salinibacter ruber
Kuiper's Test Statistic
Number of genes
1.0
1.5
2.0
2.5
0
100
200
300
400
500
600
700
169
161
152
144
135
127
118
110
101
93
84
76
67
59
50
42
34
20
10
1
Persistent Genes
Rare Genes
Groups of Genes
Genes Sorted According to Persistent Index and Grouped into Batches

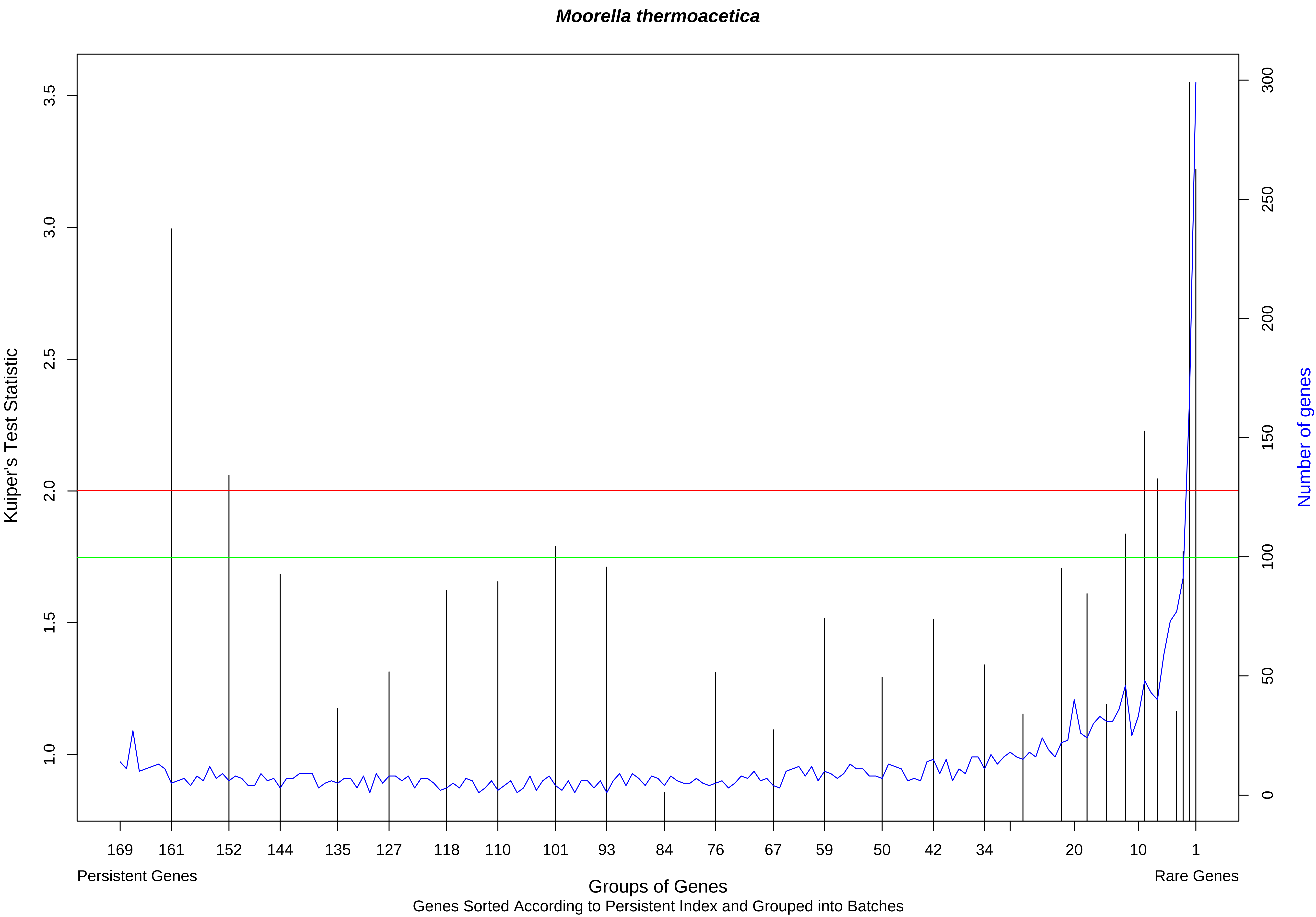

Moorella thermoacetica
Kuiper's Test Statistic
1.0
1.5
2.0
2.5
3.0
3.5
Number of genes
0
50
100
150
200
250
300
169
161
152
144
135
127
118
110
101
93
84
76
67
59
50
42
34
20
10
1
Persistent Genes
Rare Genes
Groups of Genes
Genes Sorted According to Persistent Index and Grouped into Batches

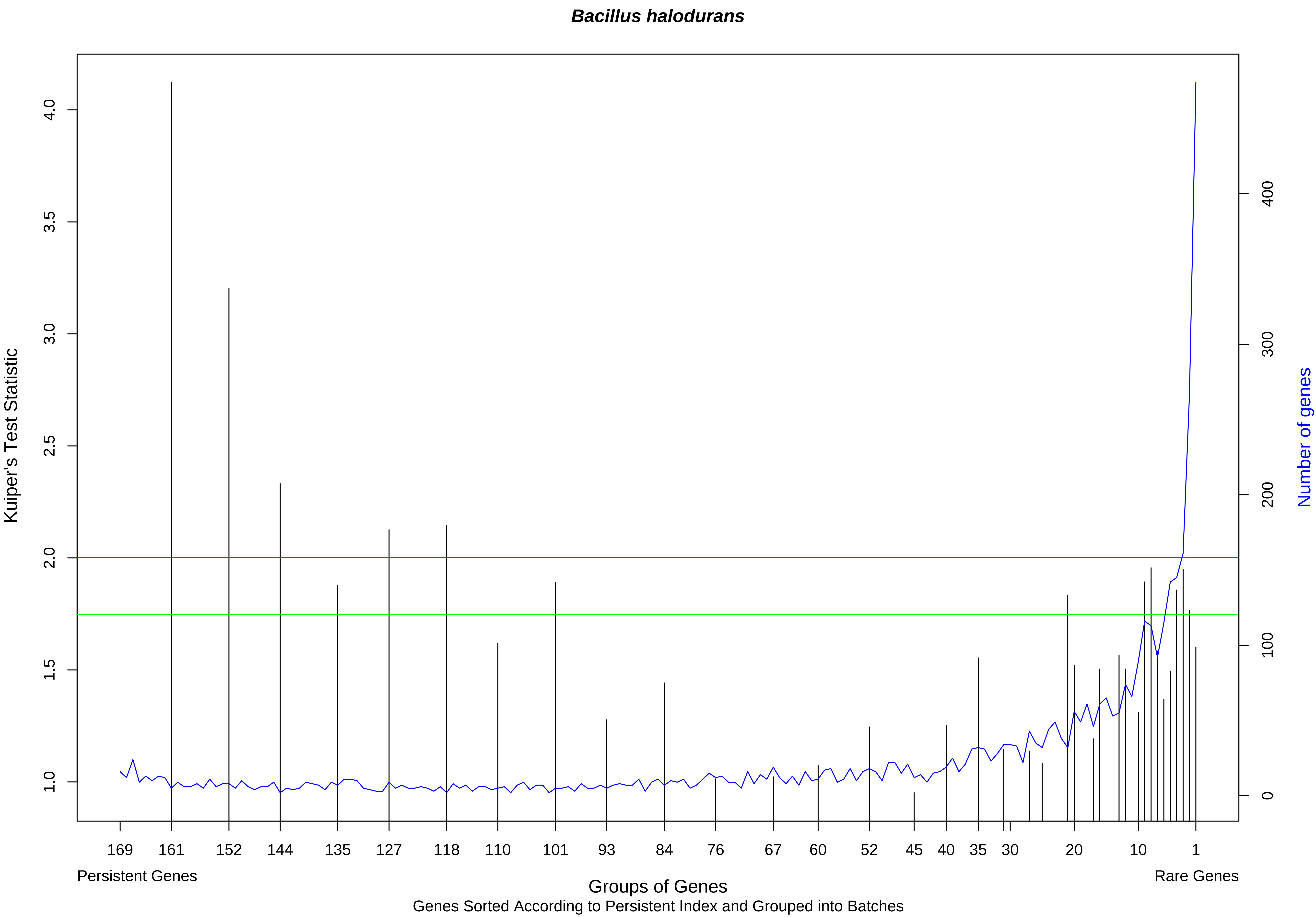
Bacillus halodurans
Kuiper's Test Statistic
1.0
1.5
2.0
2.5
3.0
3.5
4.0
Number of genes
0
100
200
300
400
169
161
152
144
135
127
118
110
101
93
84
76
67
60
52
45
40
35
30
20
10
1
Persistent Genes
Rare Genes
Groups of Genes
Genes Sorted According to Persistent Index and Grouped into Batches

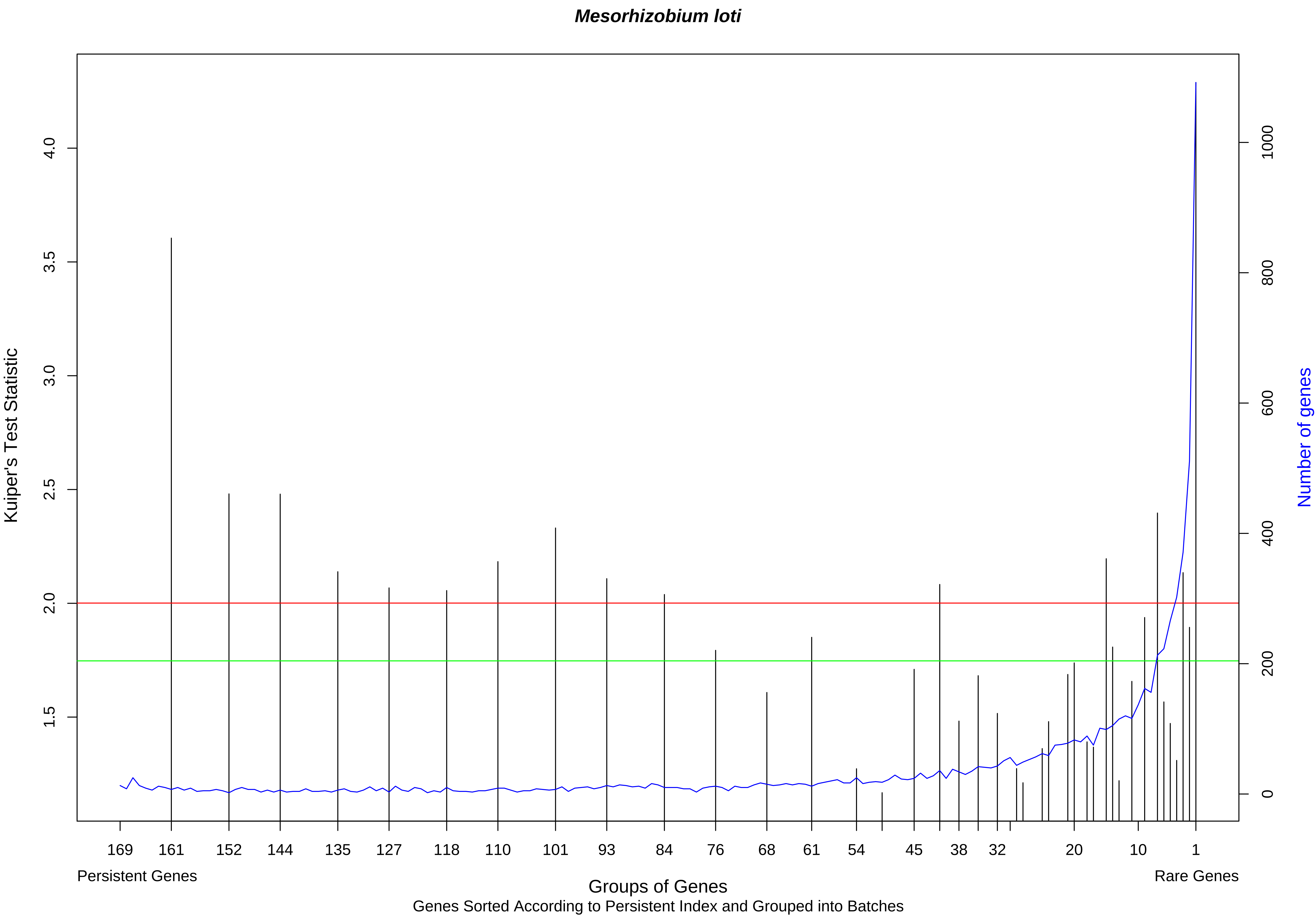

Mesorhizobium loti
Kuiper's Test Statistic
Number of genes
1.5
2.0
2.5
3.0
3.5
4.0
0
200
400
600
800
1000
169
161
152
144
135
127
118
110
101
93
84
76
68
61
54
45
38
32
20
10
1
Persistent Genes
Rare Genes
Groups of Genes
Genes Sorted According to Persistent Index and Grouped into Batches

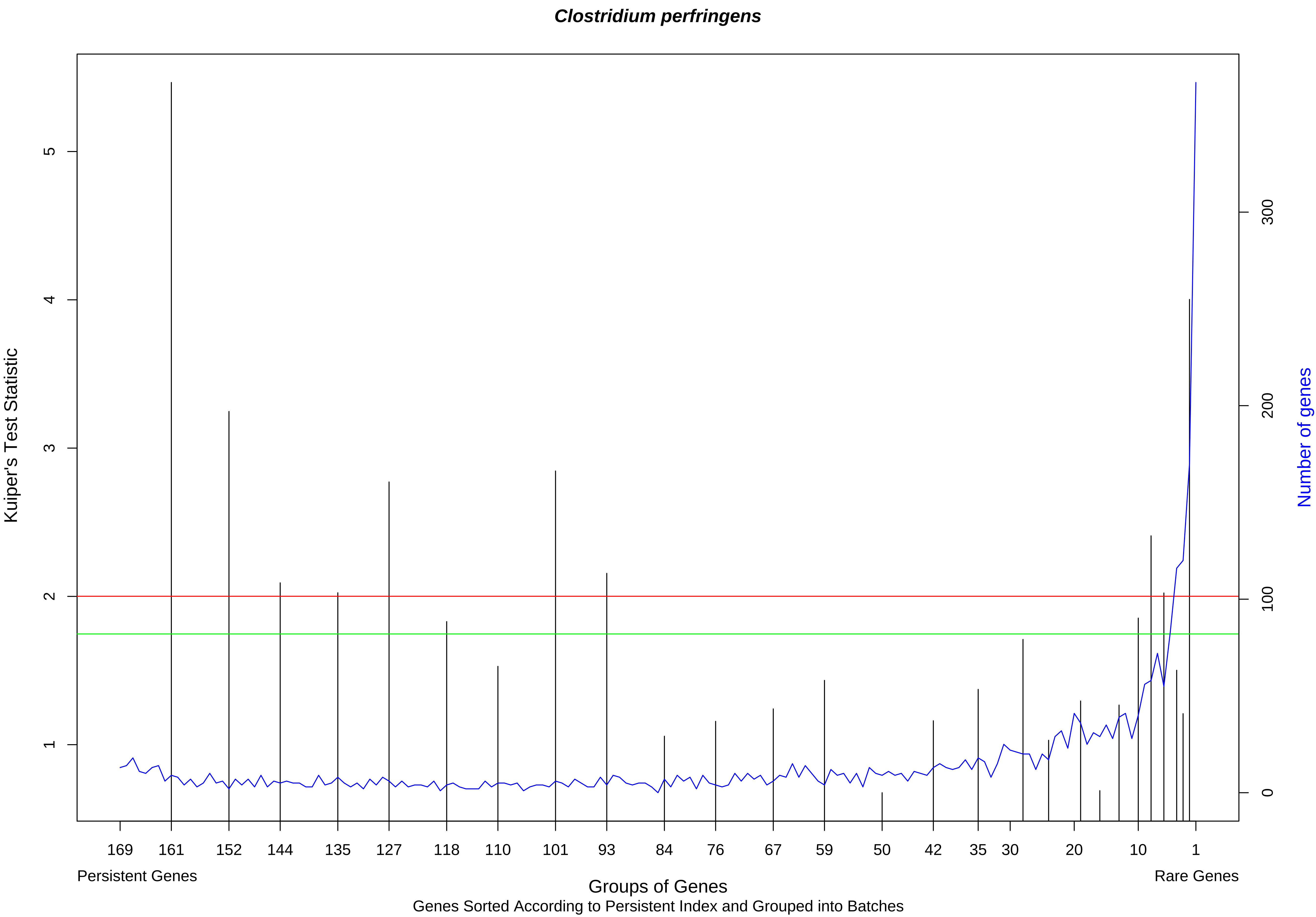

Clostridium perfringens
Kuiper's Test Statistic
1
2
3
4
5
Number of genes
0
100
200
300
169
161
152
144
135
127
118
110
101
93
84
76
67
59
50
42
35
30
20
10
1
Persistent Genes
Rare Genes
Groups of Genes
Genes Sorted According to Persistent Index and Grouped into Batches

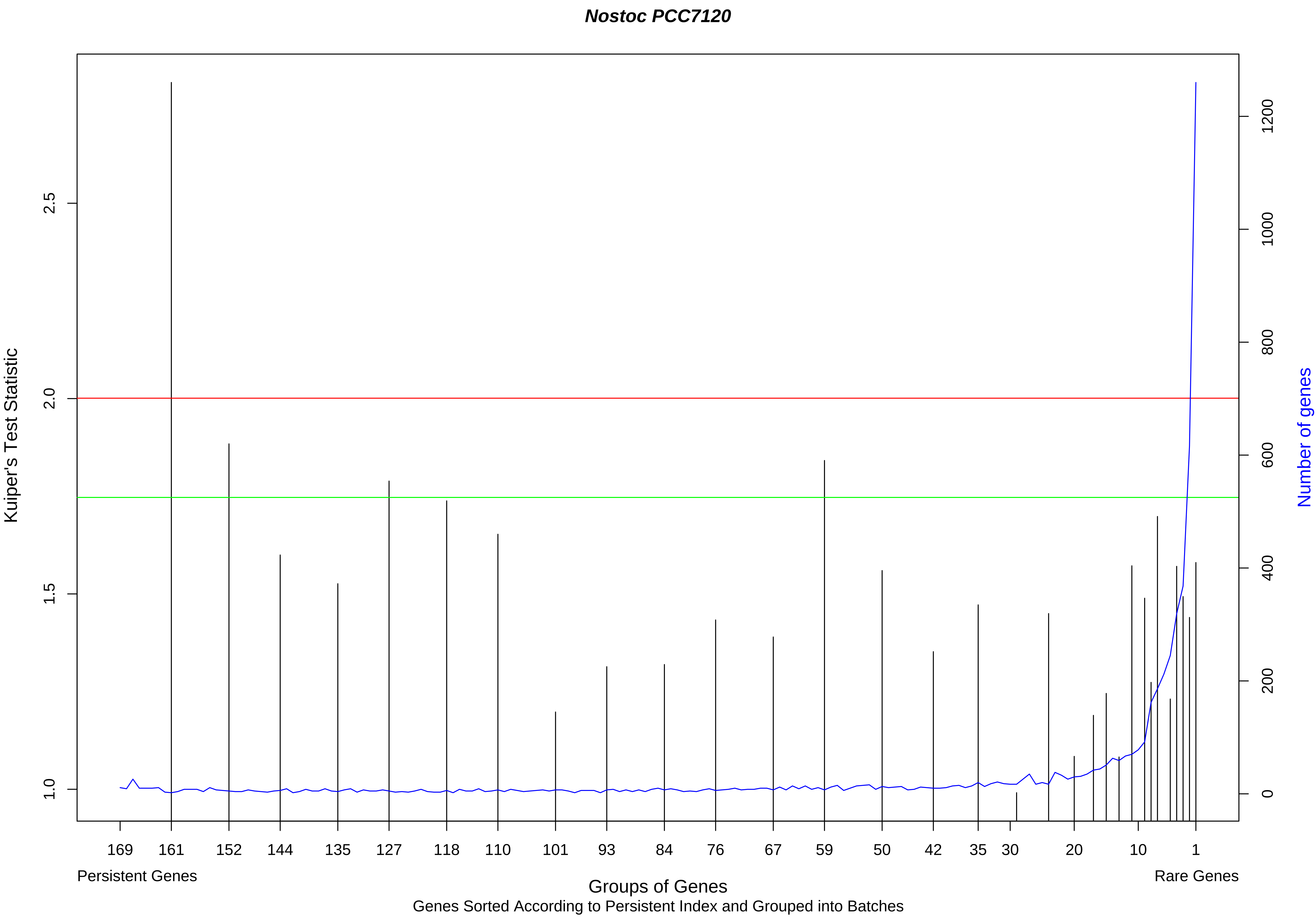

Nostoc PCC7120
Kuiper's Test Statistic
1.0
1.5
2.0
2.5
Number of genes
0
200
400
600
800
1000
1200
169
161
152
144
135
127
118
110
101
93
84
76
67
59
50
42
35
30
20
10
1
Persistent Genes
Rare Genes
Groups of Genes
Genes Sorted According to Persistent Index and Grouped into Batches

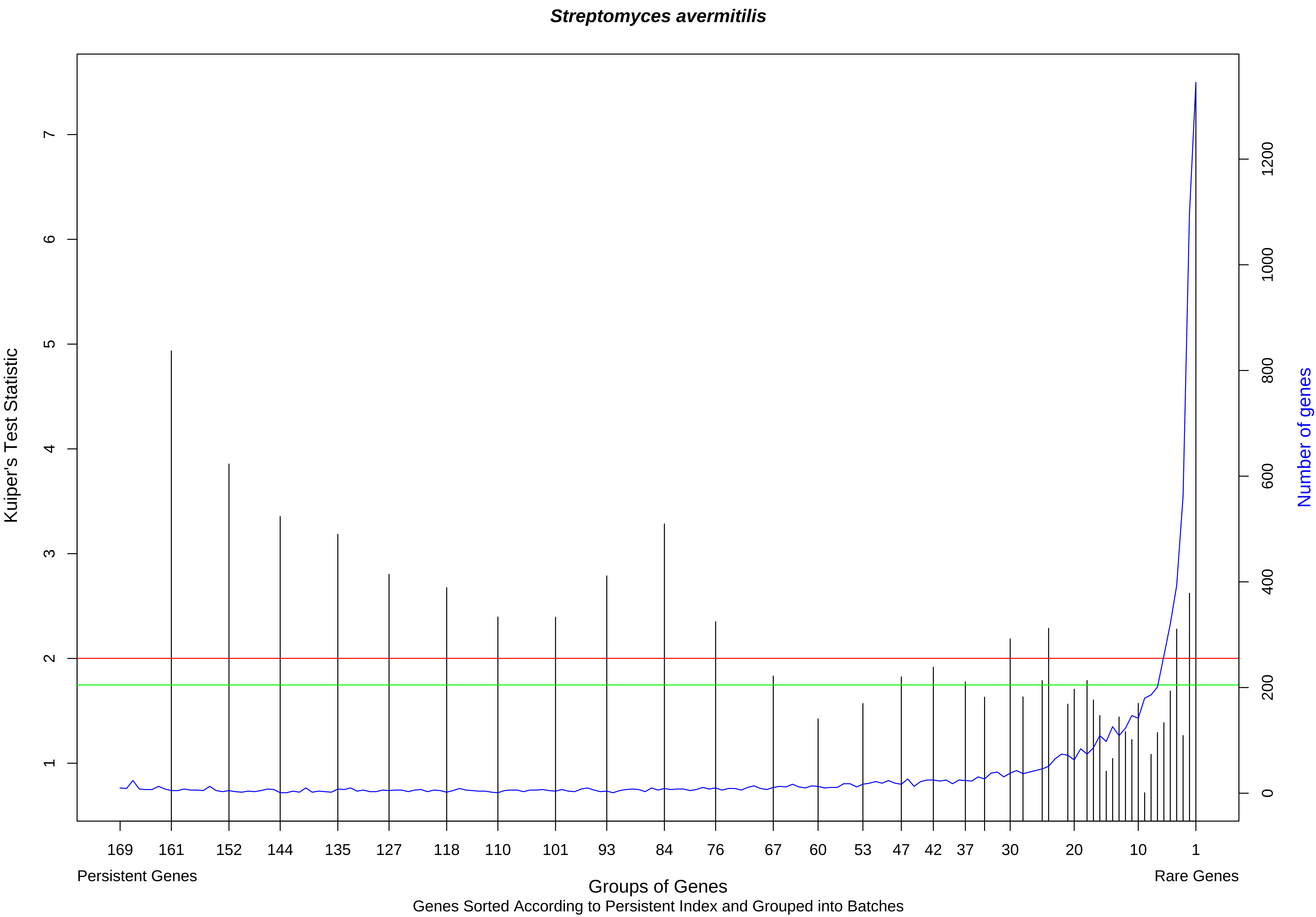
Streptomyces avermitilis
Kuiper's Test Statistic
Number of genes
1
2
3
4
5
6
7
0
200
400
600
800
1000
1200
169
161
152
144
135
127
118
110
101
93
84
76
67
60
53
47
42
37
30
20
10
1
Persistent Genes
Rare Genes
Groups of Genes
Genes Sorted According to Persistent Index and Grouped into Batches

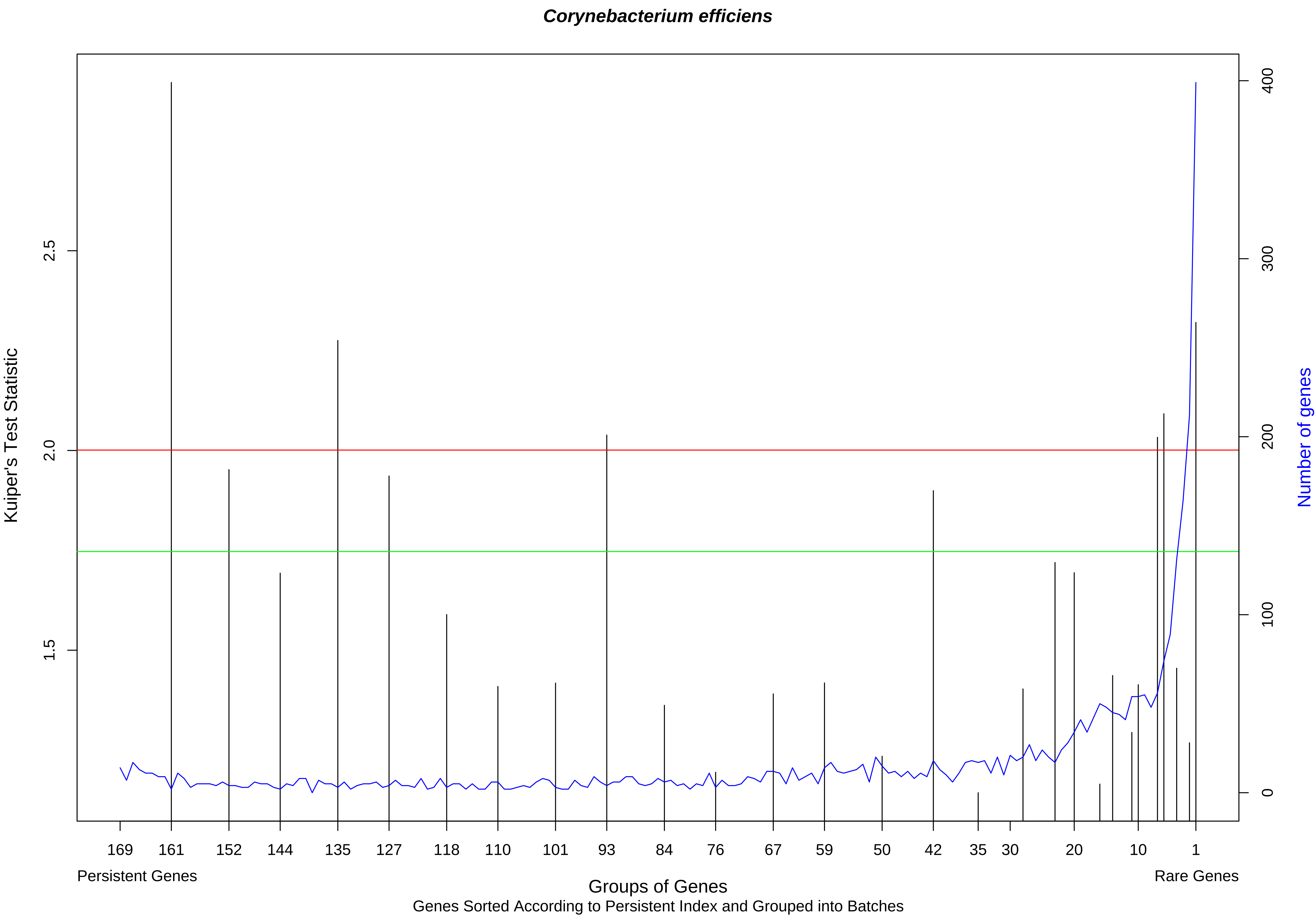
Corynebacterium efficiens
Kuiper's Test Statistic
2.5
2.0
1.5
Number of genes
400
300
200
100
0
169
161
152
144
135
127
118
110
101
93
84
76
67
59
50
42
35
30
20
10
1
Persistent Genes
Rare Genes
Groups of Genes
Genes Sorted According to Persistent Index and Grouped into Batches

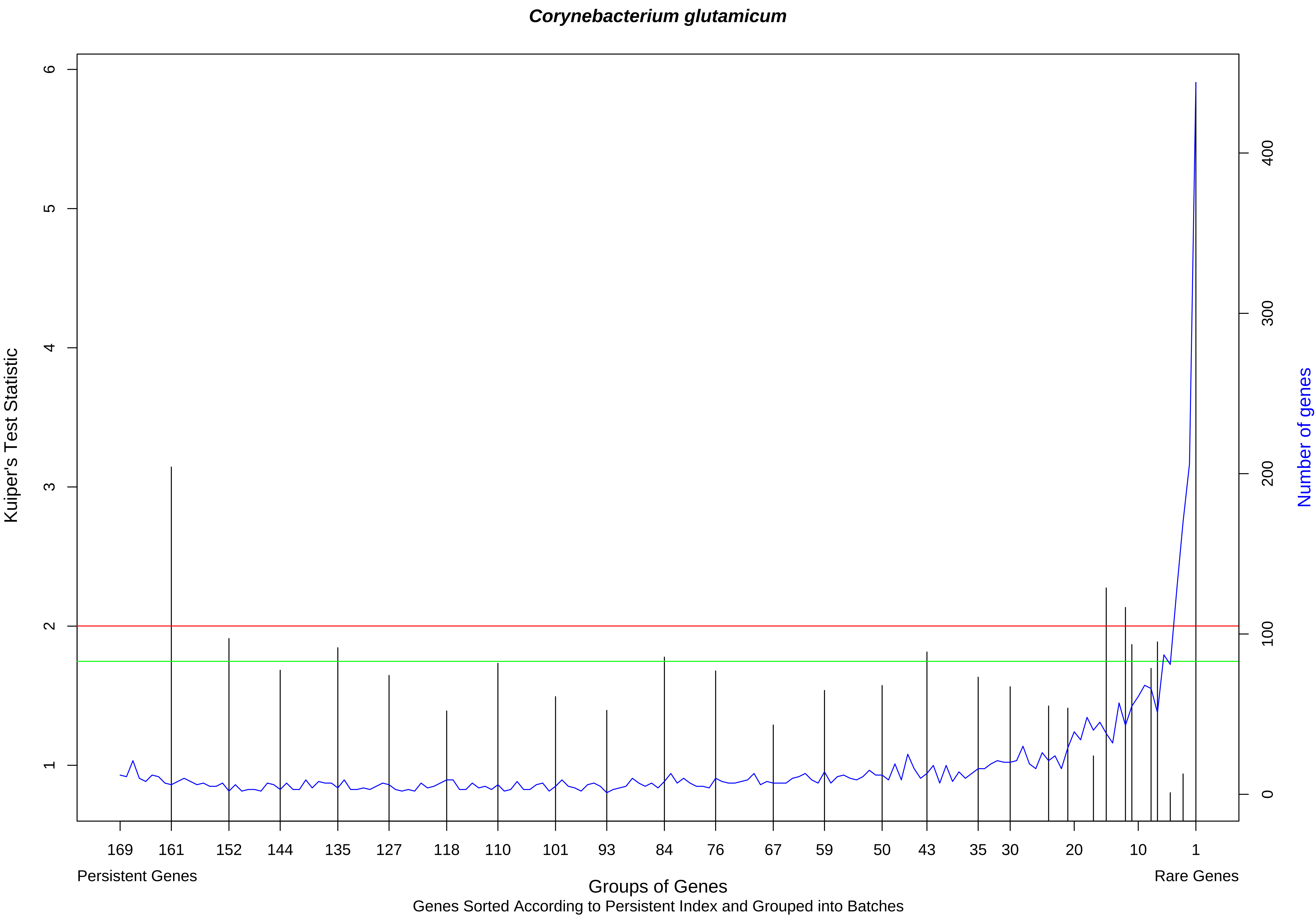
Corynebacterium glutamicum
Kuiper's Test Statistic
Number of genes
1
2
3
4
5
6
0
100
200
300
400
169
161
152
144
135
127
118
110
101
93
84
76
67
59
50
43
35
30
20
10
1
Persistent Genes
Rare Genes
Groups of Genes
Genes Sorted According to Persistent Index and Grouped into Batches

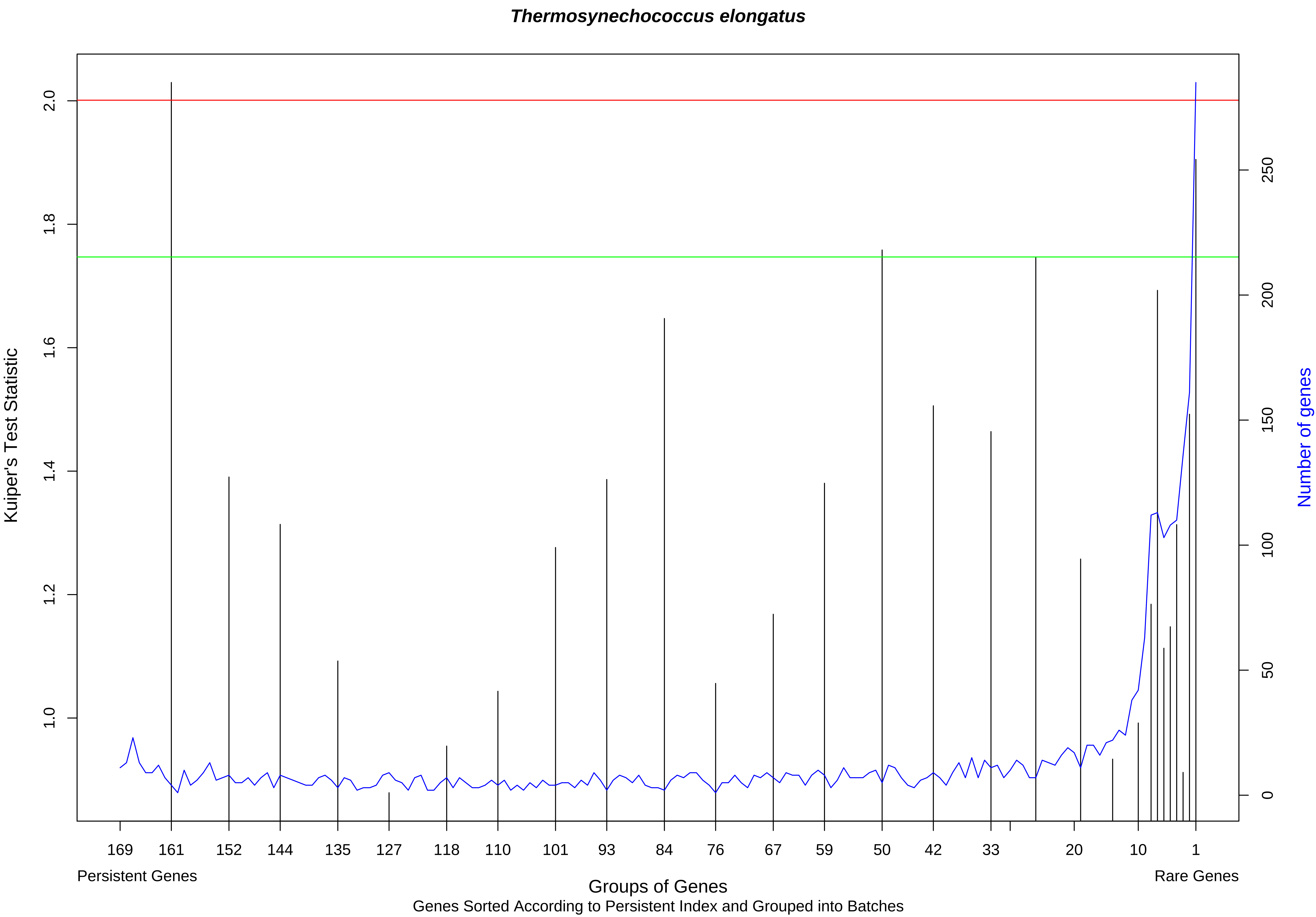
Thermosynechococcus elongatus
Kuiper's Test Statistic
Number of genes
2.0
1.8
1.6
1.4
1.2
1.0
250
200
150
100
50
0
169
161
152
144
135
127
118
110
101
93
84
76
67
59
50
42
33
20
10
1
Persistent Genes
Rare Genes
Groups of Genes
Genes Sorted According to Persistent Index and Grouped into Batches

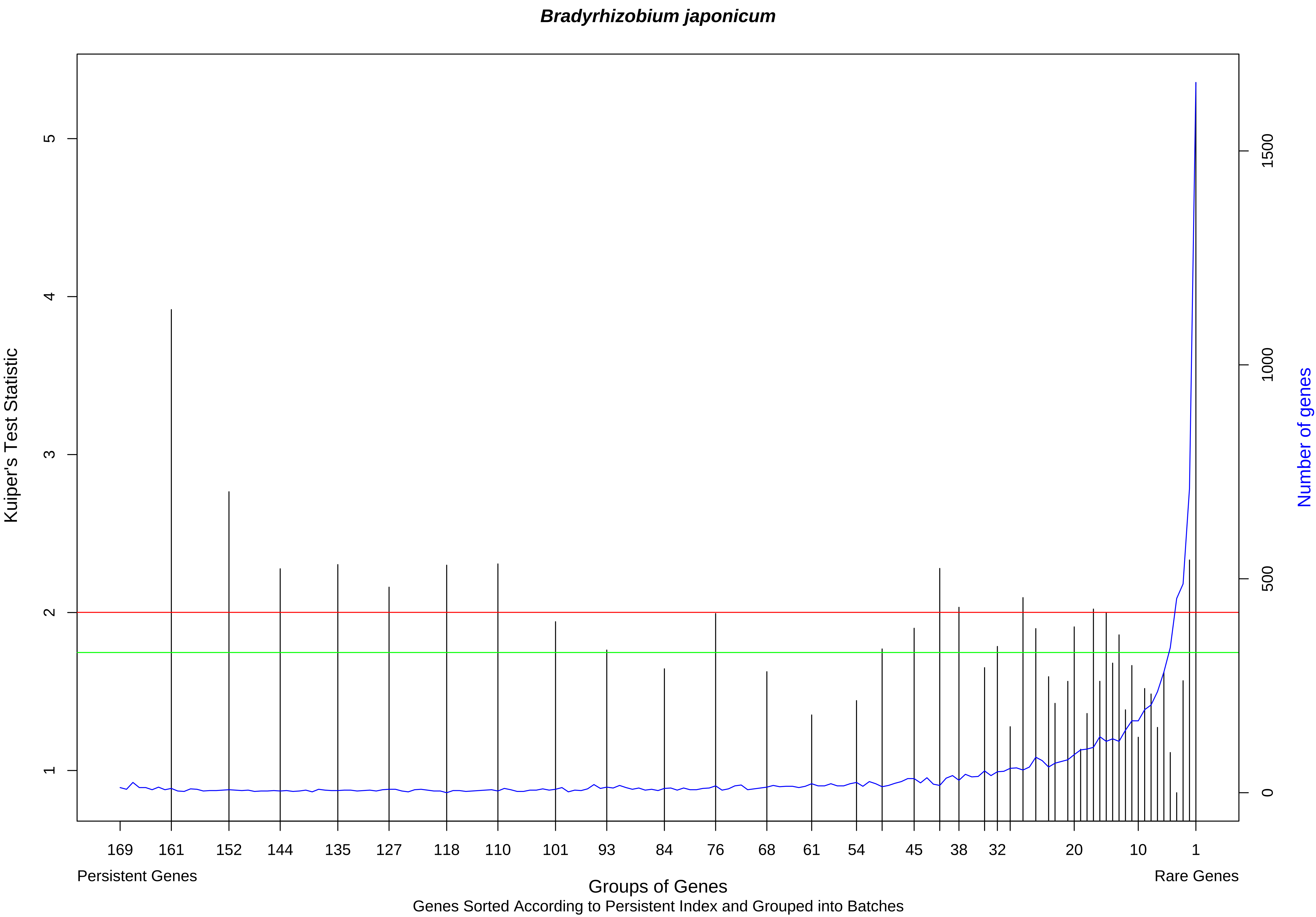
Bradyrhizobium japonicum
Kuiper's Test Statistic
Number of genes
1
2
3
4
5
0
500
1000
1500
169
161
152
144
135
127
118
110
101
93
84
76
68
61
54
45
38
32
20
10
1
Persistent Genes
Rare Genes
Groups of Genes
Genes Sorted According to Persistent Index and Grouped into Batches

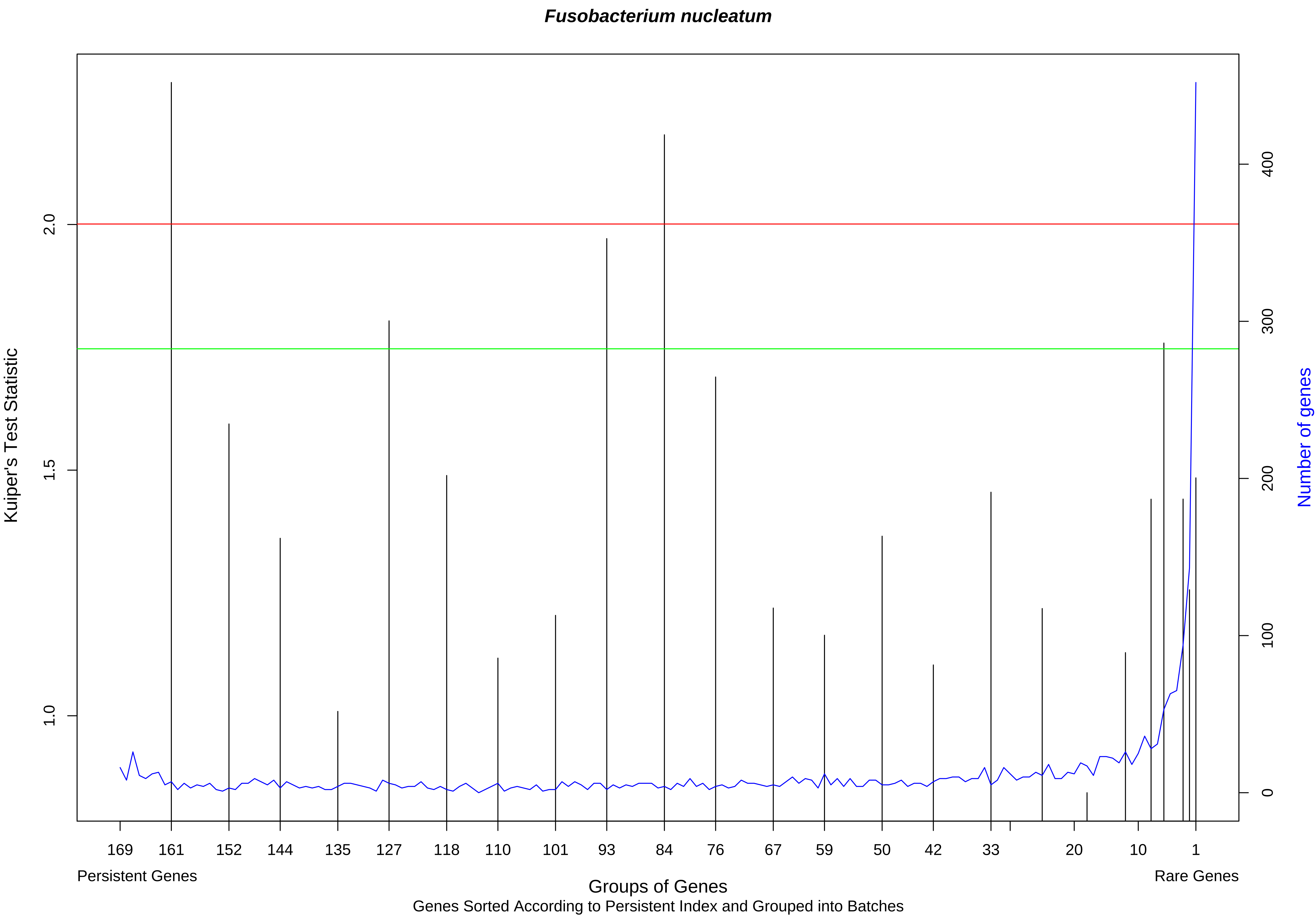

Fusobacterium nucleatum
Kuiper's Test Statistic
1.0
1.5
2.0
Number of genes
0
100
200
300
400
169
161
152
144
135
127
118
110
101
93
84
76
67
59
50
42
33
20
10
1
Persistent Genes
Rare Genes
Groups of Genes
Genes Sorted According to Persistent Index and Grouped into Batches

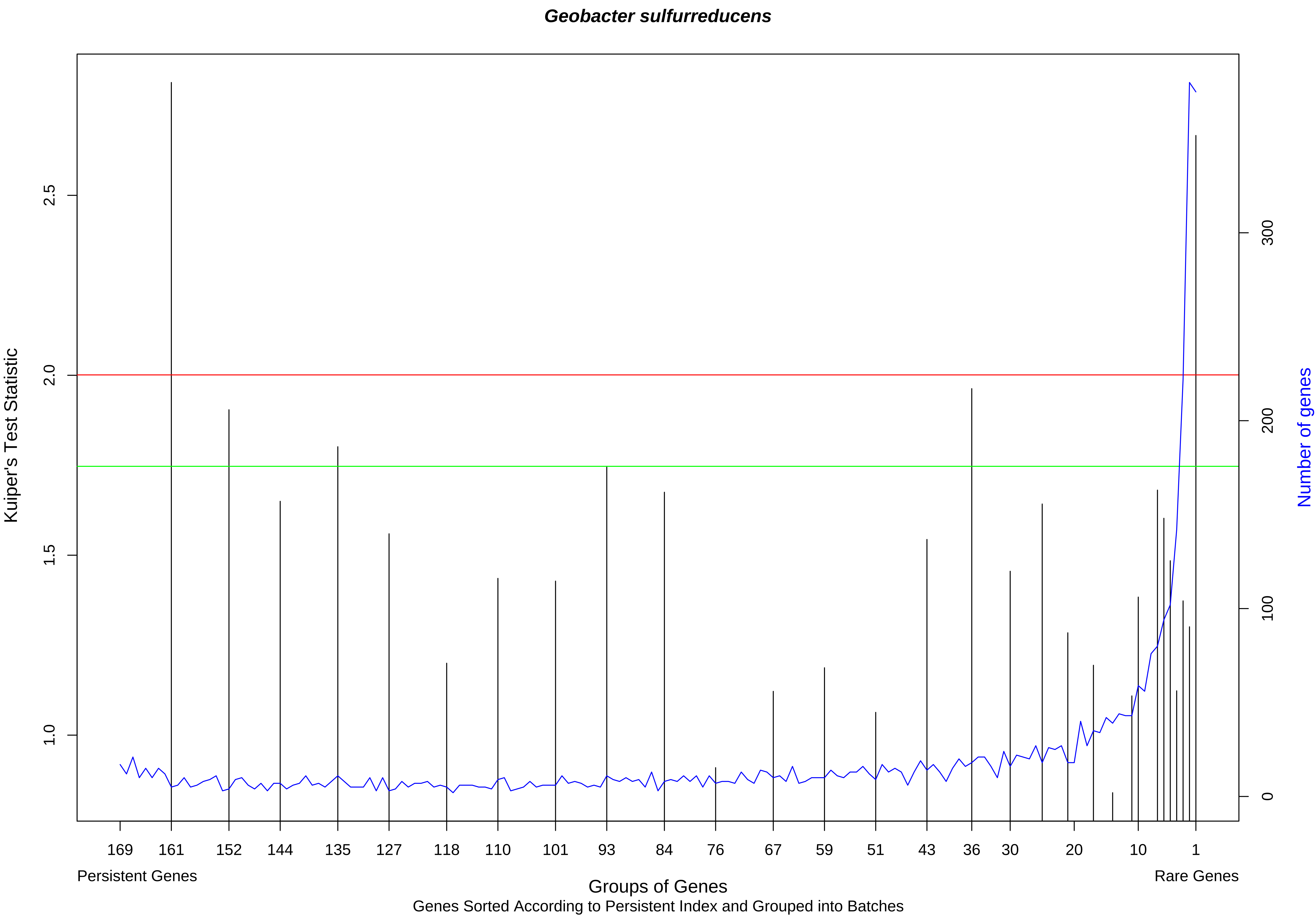
Geobacter sulfurreducens
Kuiper's Test Statistic
Number of genes
1.0
1.5
2.0
2.5
0
100
200
300
169 161 152 144 135 127 118 110 101 93 84 76 67 59 51 43 36 30 20 10 1
Persistent Genes
Rare Genes
Groups of Genes
Genes Sorted According to Persistent Index and Grouped into Batches

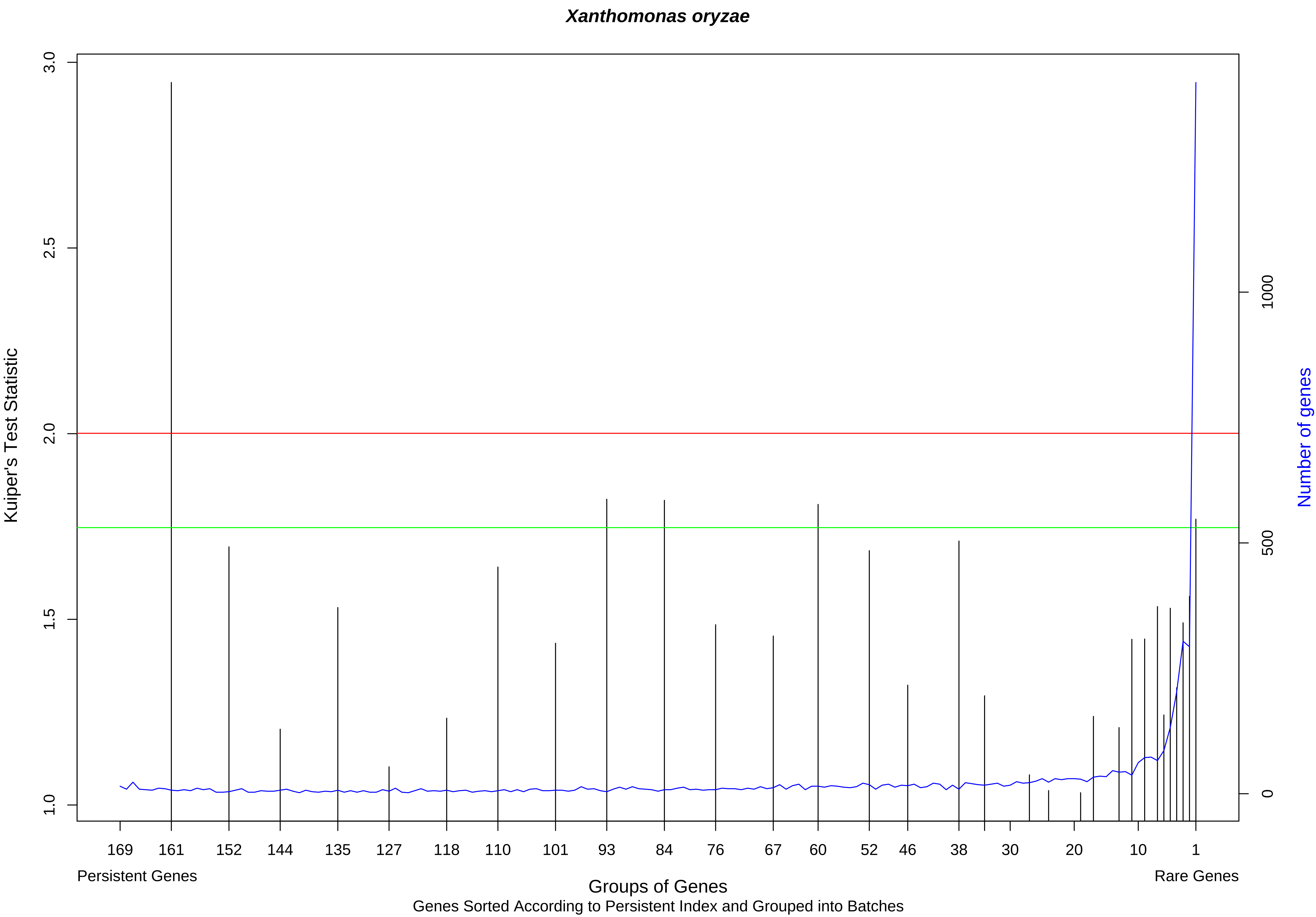

Xanthomonas oryzae
Kuiper's Test Statistic
Number of genes
3.0
2.5
2.0
1.5
1.0
1000
500
0
169
161
152
144
135
127
118
110
101
93
84
76
67
60
52
46
38
30
20
10
1
Persistent Genes
Rare Genes
Groups of Genes
Genes Sorted According to Persistent Index and Grouped into Batches

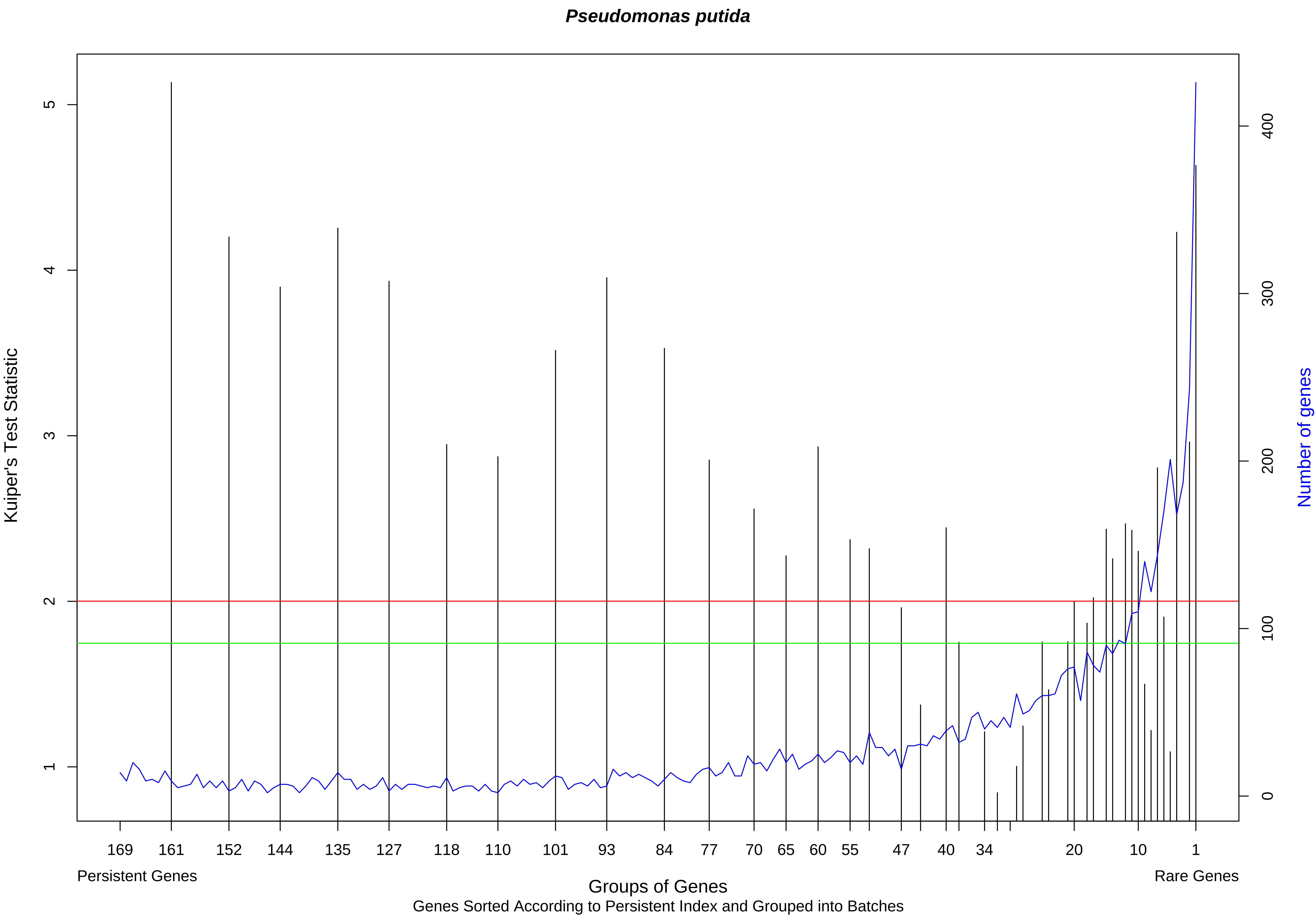

Pseudomonas putida
Kuiper's Test Statistic
1
2
3
4
5
Number of genes
0
100
200
300
400
169
161
152
144
135
127
118
110
101
93
84
77
70
65
60
55
47
40
34
20
10
1
Persistent Genes
Rare Genes
Groups of Genes
Genes Sorted According to Persistent Index and Grouped into Batches

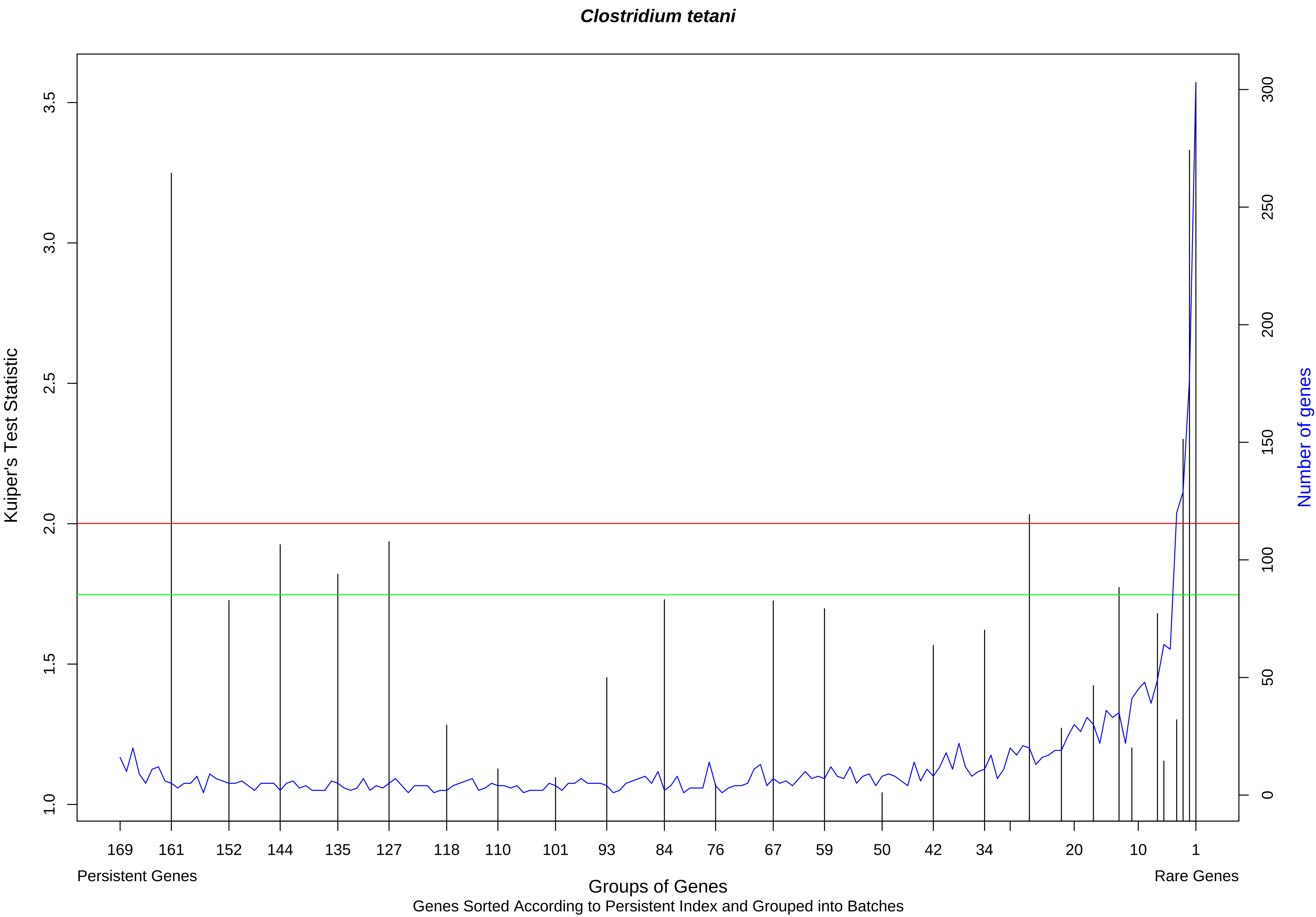
Clostridium tetani
Kuiper's Test Statistic
Number of genes
3.5
3.0
2.5
2.0
1.5
1.0
300
250
200
150
100
50
0
169
161
152
144
135
127
118
110
101
93
84
76
67
59
50
42
34
20
10
1
Persistent Genes
Rare Genes
Groups of Genes
Genes Sorted According to Persistent Index and Grouped into Batches

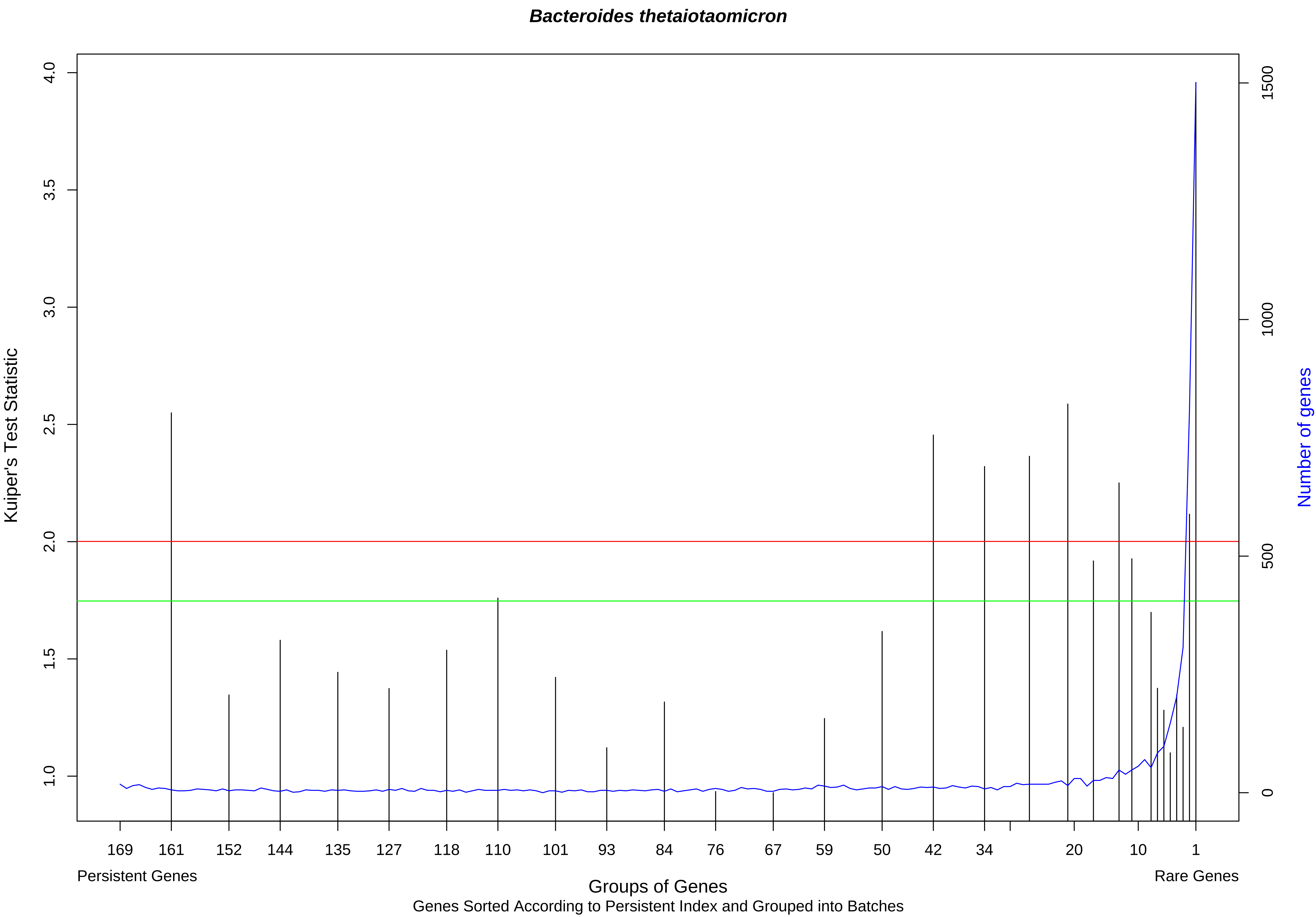

Bacteroides thetaiotaomicron
Kuiper's Test Statistic
4.0
3.5
3.0
2.5
2.0
1.5
1.0
Number of genes
1500
1000
500
0
169
161
152
144
135
127
118
110
101
93
84
76
67
59
50
42
34
20
10
1
Persistent Genes
Rare Genes
Groups of Genes
Genes Sorted According to Persistent Index and Grouped into Batches

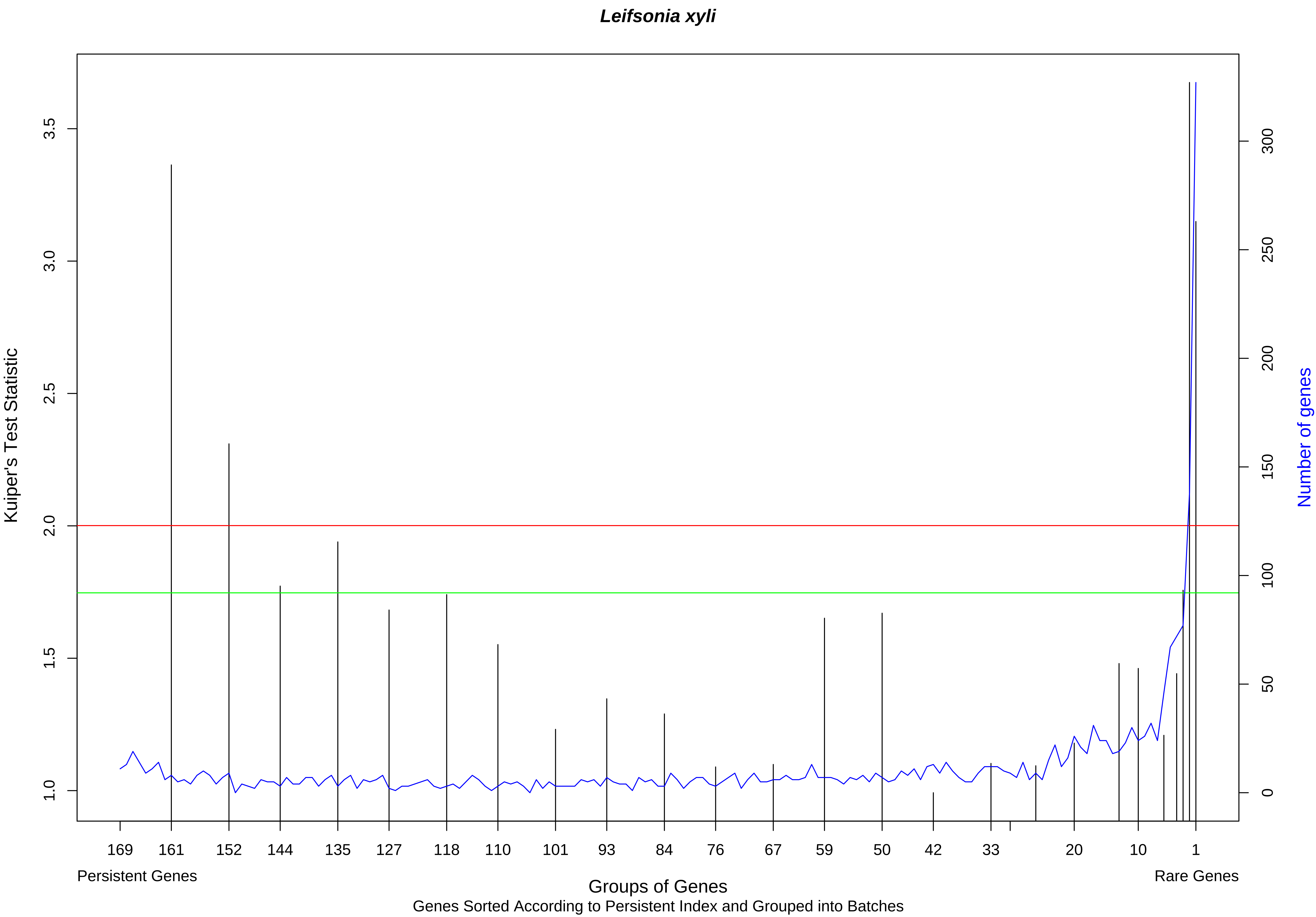
Leifsonia xyli
Kuiper's Test Statistic
Number of genes
1.0
1.5
2.0
2.5
3.0
3.5
0
50
100
150
200
250
300
169
161
152
144
135
127
118
110
101
93
84
76
67
59
50
42
33
20
10
1
Persistent Genes
Rare Genes
Groups of Genes
Genes Sorted According to Persistent Index and Grouped into Batches

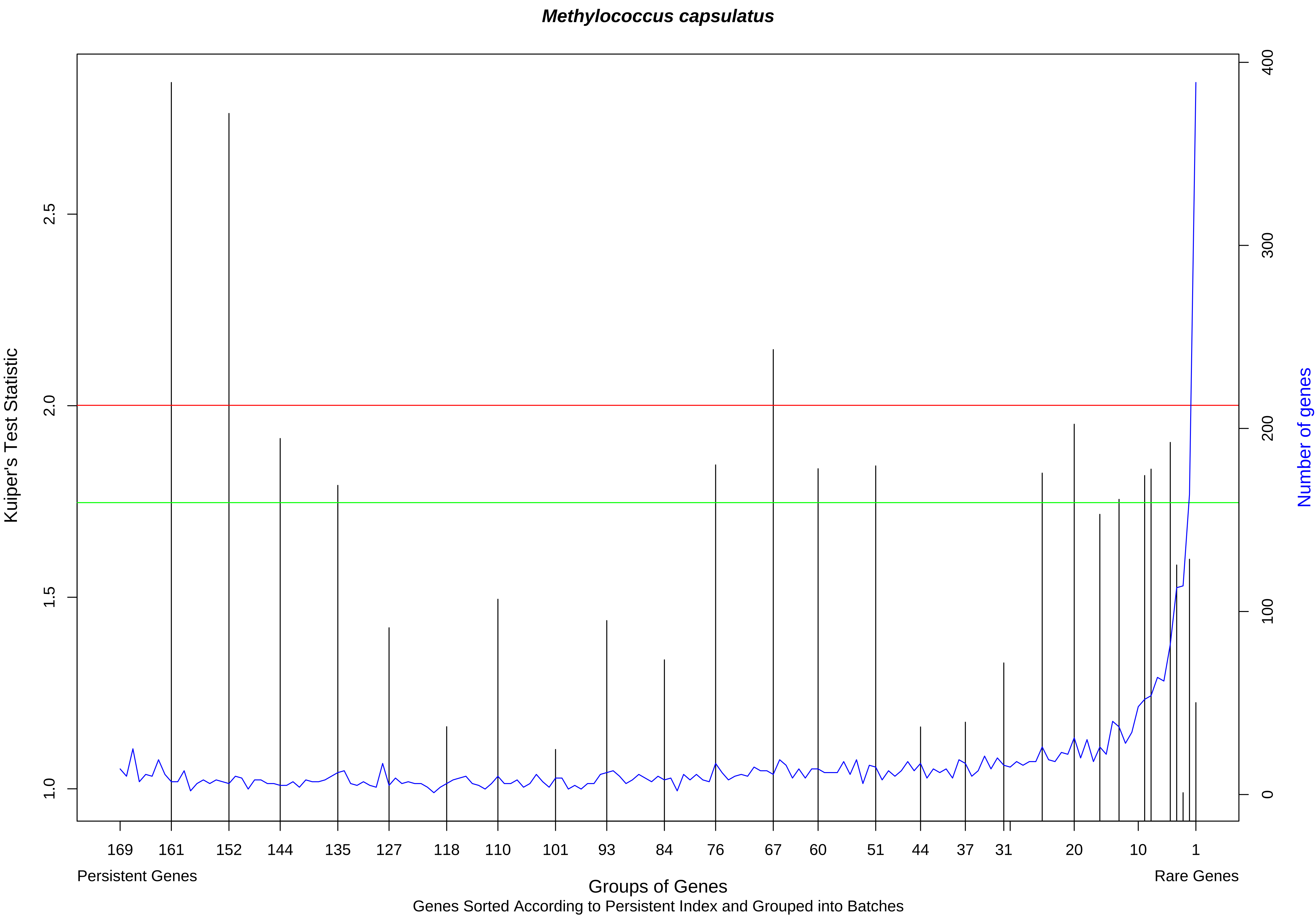

Methylococcus capsulatus
Kuiper's Test Statistic
Number of genes
1.0
1.5
2.0
2.5
0
100
200
300
400
169
161
152
144
135
127
118
110
101
93
84
76
67
60
51
44
37
31
20
10
1
Persistent Genes
Rare Genes
Groups of Genes
Genes Sorted According to Persistent Index and Grouped into Batches

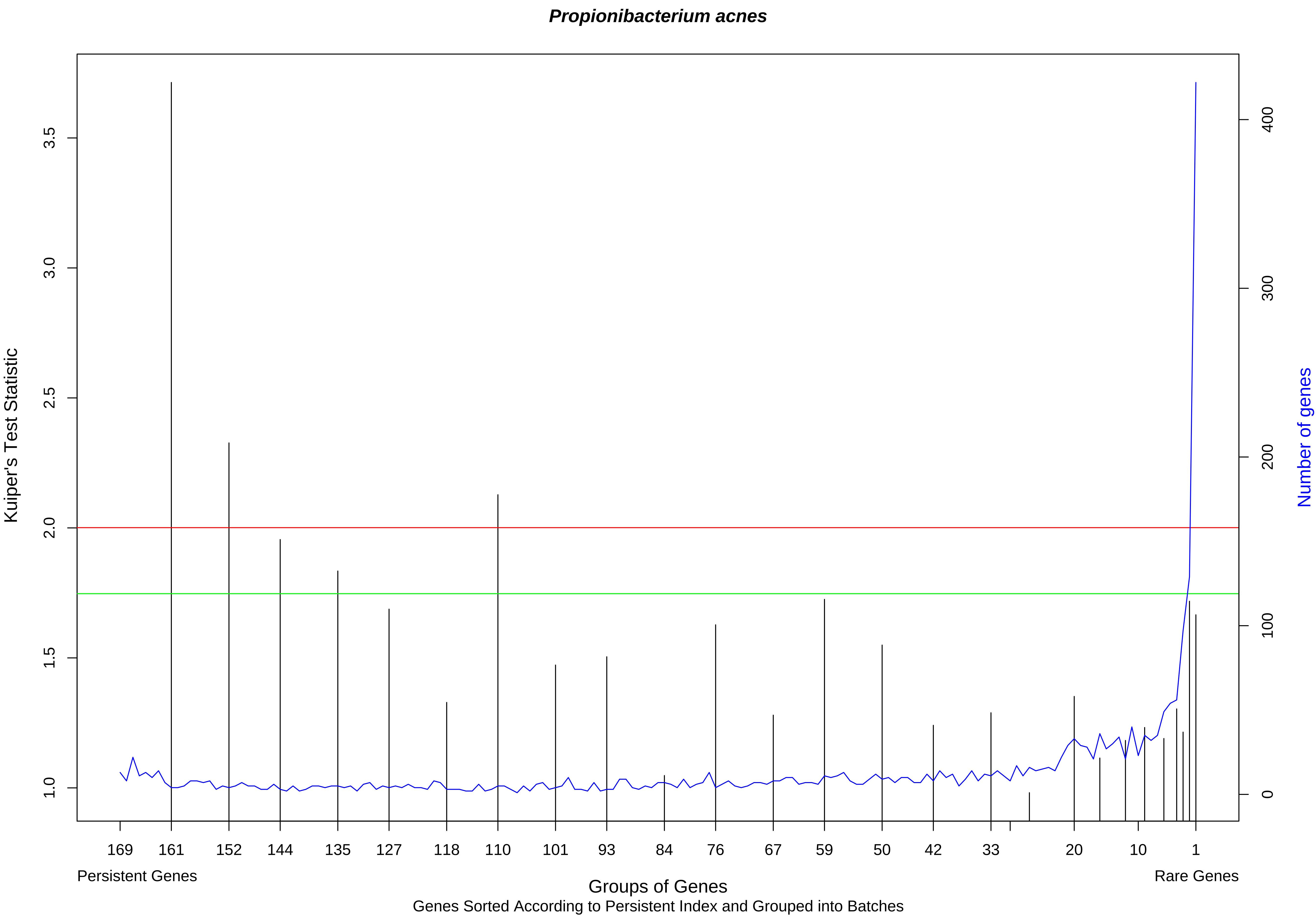

Propionibacterium acnes
Kuiper's Test Statistic
Number of genes
1.0
1.5
2.0
2.5
3.0
3.5
0
100
200
300
400
169
161
152
144
135
127
118
110
101
93
84
76
67
59
50
42
33
20
10
1
Persistent Genes
Rare Genes
Groups of Genes
Genes Sorted According to Persistent Index and Grouped into Batches

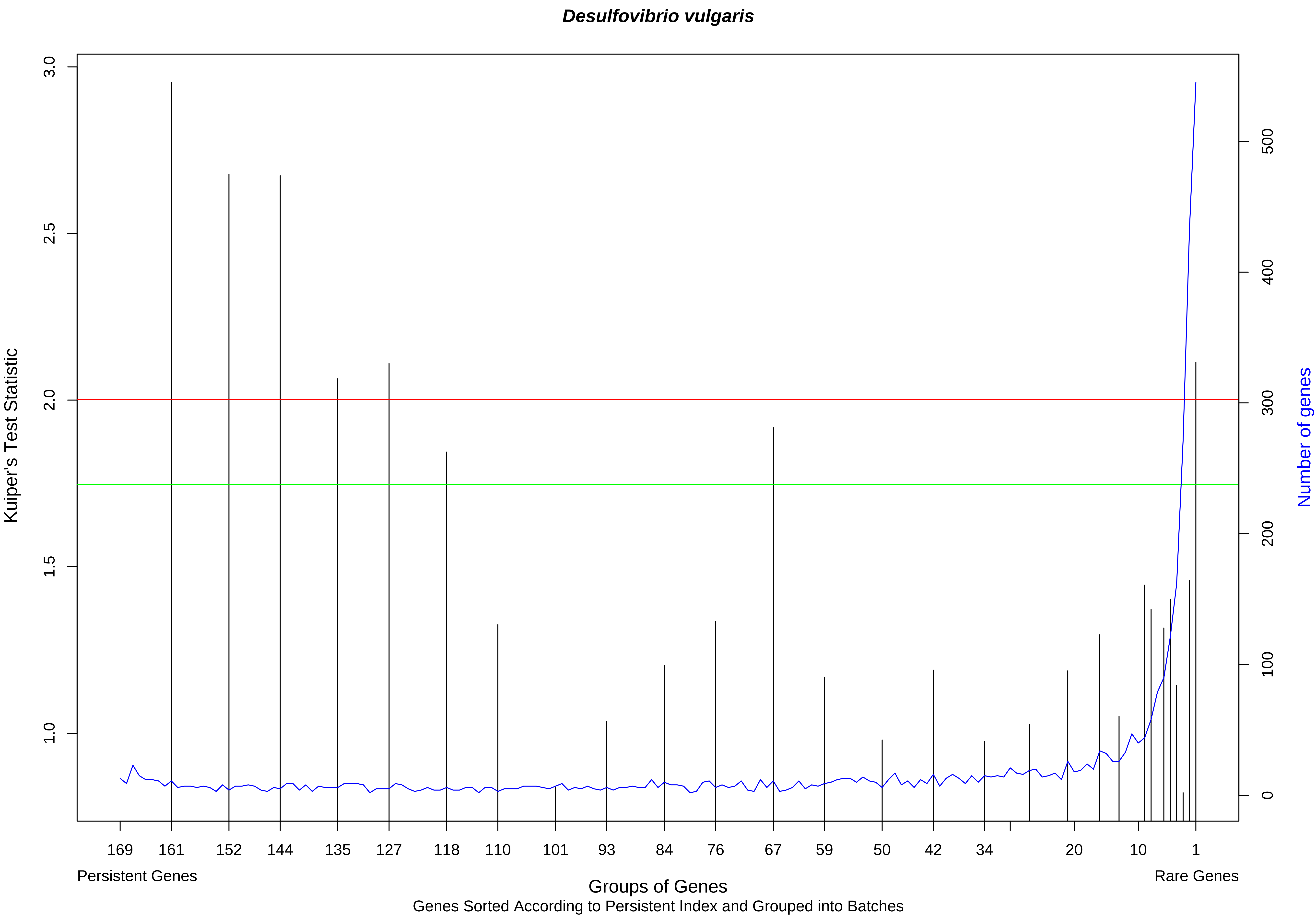
Desulfovibrio vulgaris
Kuiper's Test Statistic
Number of genes
3.0
2.5
2.0
1.5
1.0
500
400
300
200
100
0
169
161
152
144
135
127
118
110
101
93
84
76
67
59
50
42
34
20
10
1
Persistent Genes
Rare Genes
Groups of Genes
Genes Sorted According to Persistent Index and Grouped into Batches

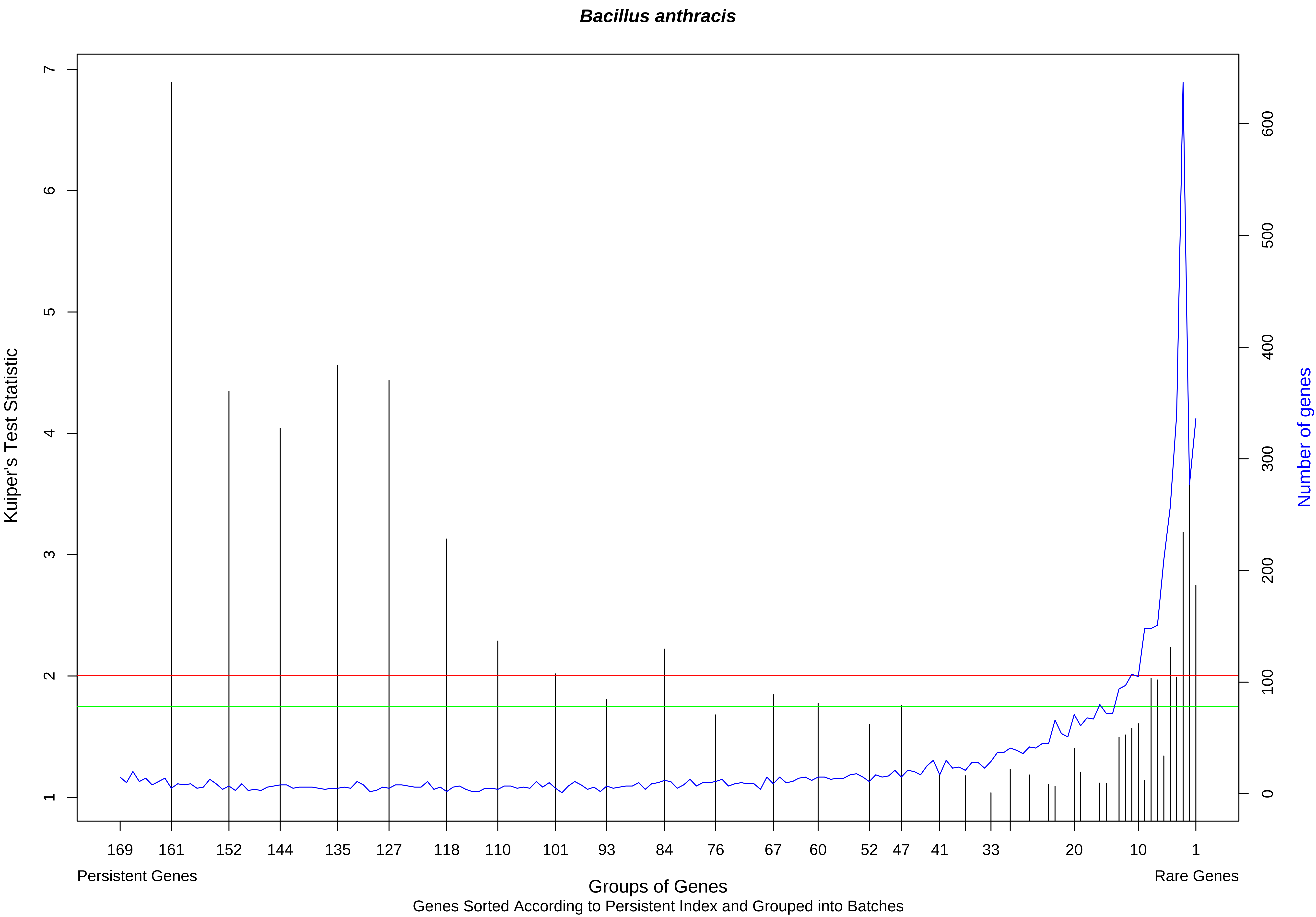

Bacillus anthracis
Kuiper's Test Statistic
1
2
3
4
5
6
7
Number of genes
0
100
200
300
400
500
600
169
161
152
144
135
127
118
110
101
93
84
76
67
60
52
47
41
33
20
10
1
Persistent Genes
Rare Genes
Groups of Genes
Genes Sorted According to Persistent Index and Grouped into Batches

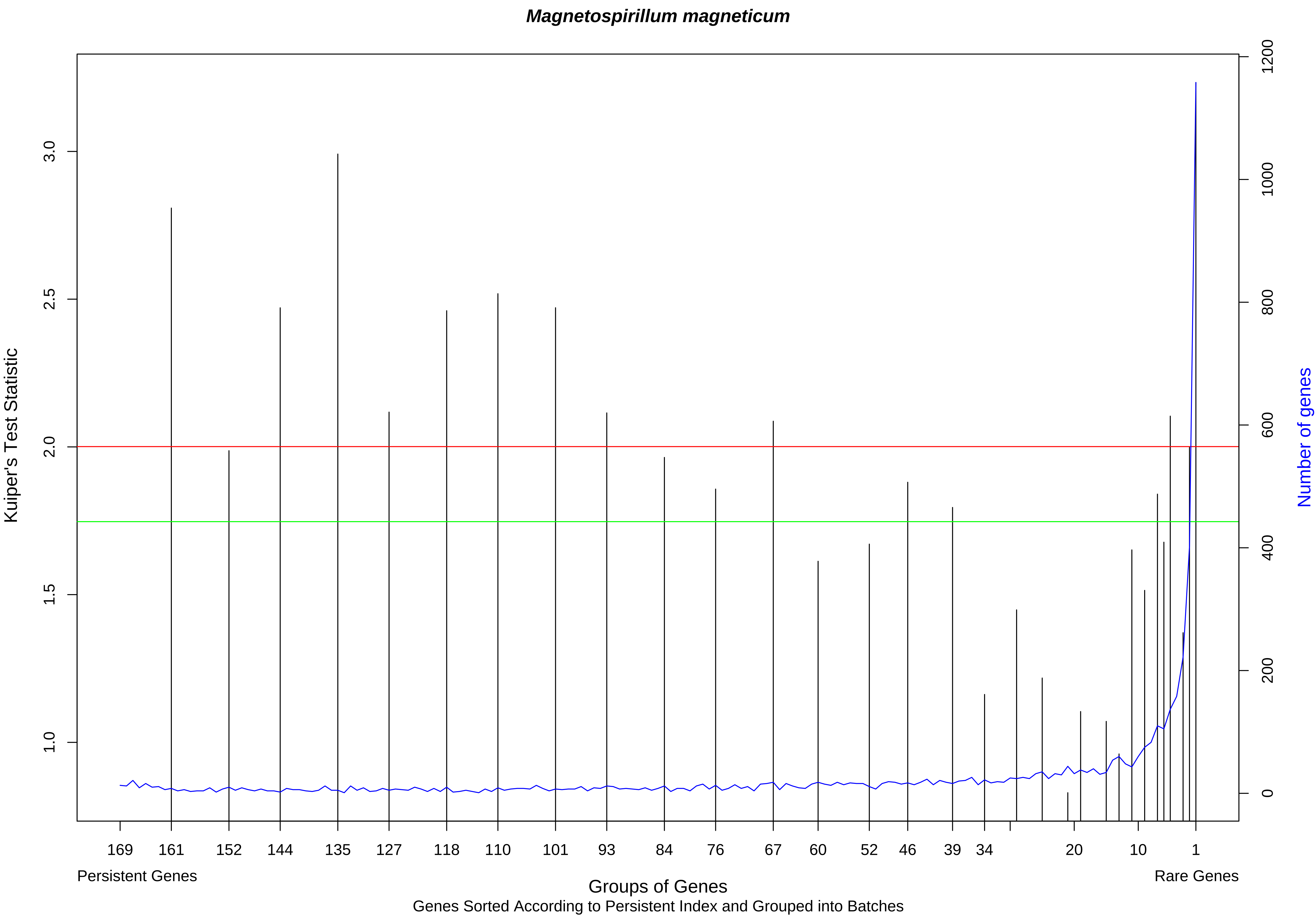

Magnetospirillum magneticum
Kuiper's Test Statistic
1.0
1.5
2.0
2.5
3.0
Number of genes
0
200
400
600
800
1000
1200
169
161
152
144
135
127
118
110
101
93
84
76
67
60
52
46
39
34
20
10
1
Persistent Genes
Rare Genes
Groups of Genes
Genes Sorted According to Persistent Index and Grouped into Batches

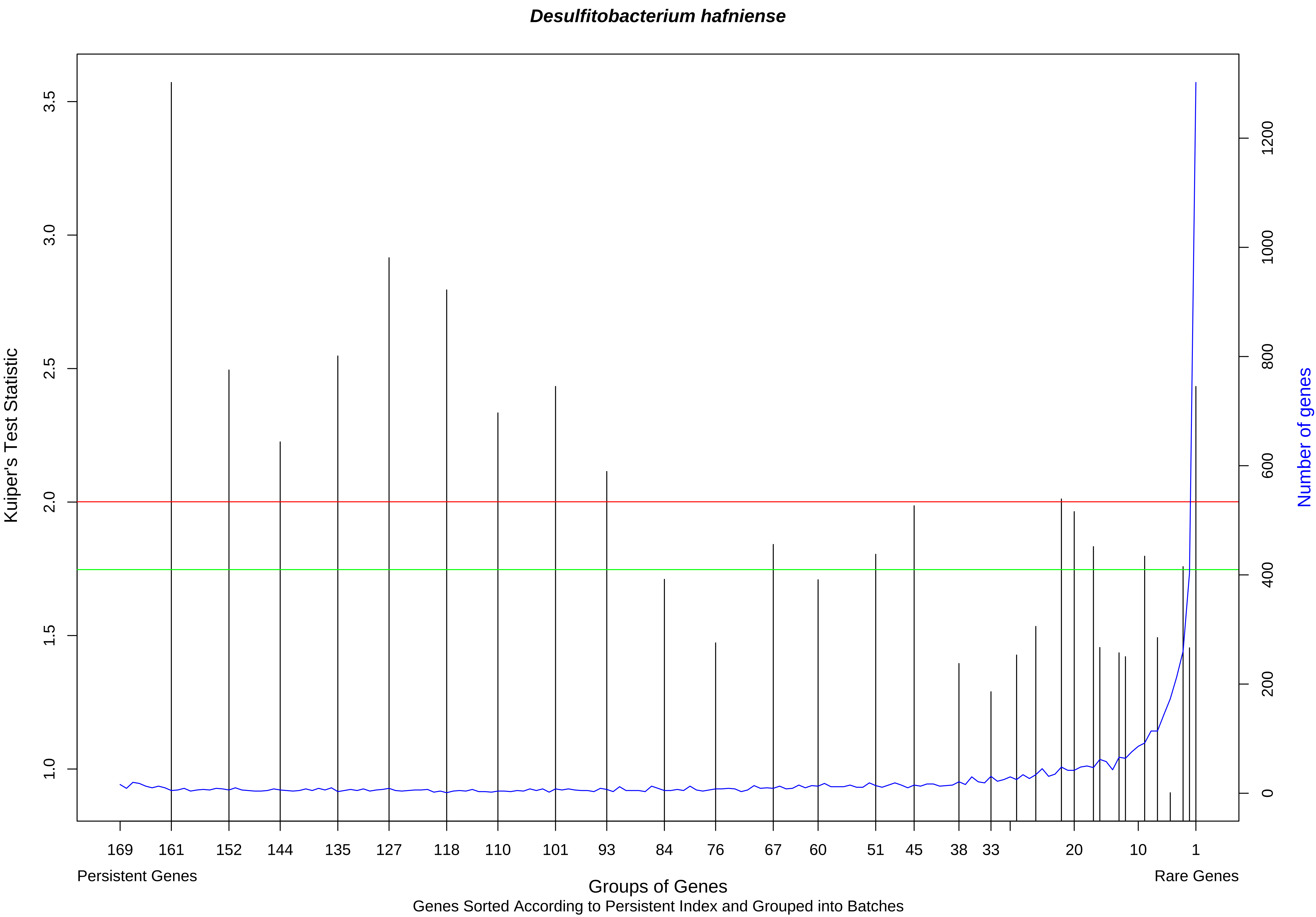
Desulfitobacterium hafniense
Kuiper's Test Statistic
Number of genes
3.5
3.0
2.5
2.0
1.5
1.0
1200
1000
800
600
400
200
0
169 161 152 144 135 127 118 110 101 93 84 76 67 60 51 45 38 33 20 10 1
Persistent Genes
Rare Genes
Groups of Genes
Genes Sorted According to Persistent Index and Grouped into Batches

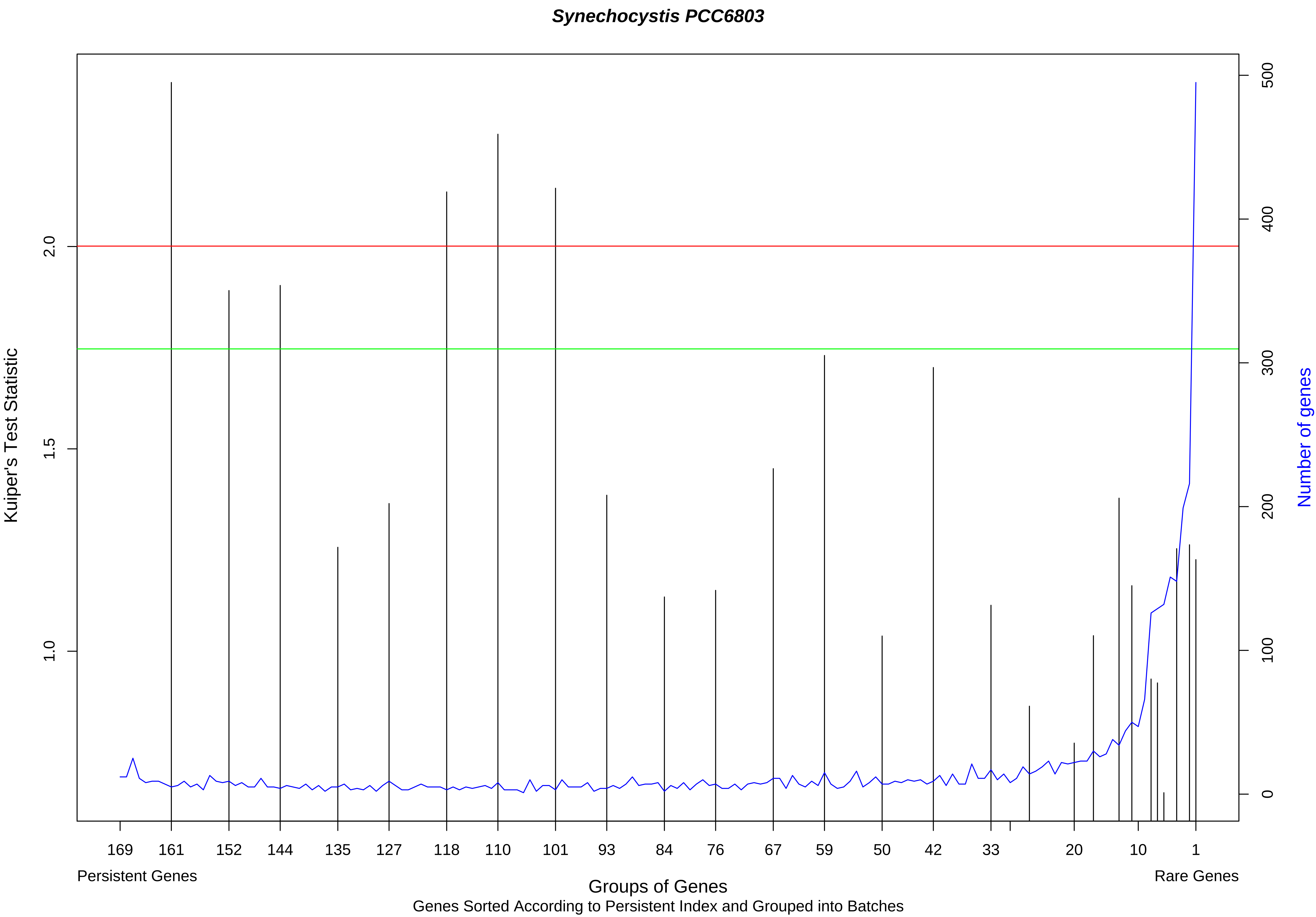

Synechocystis PCC6803
Kuiper's Test Statistic
2.0
1.5
1.0
Number of genes
500
400
300
200
100
0
169
161
152
144
135
127
118
110
101
93
84
76
67
59
50
42
33
20
10
1
Persistent Genes
Rare Genes
Groups of Genes
Genes Sorted According to Persistent Index and Grouped into Batches

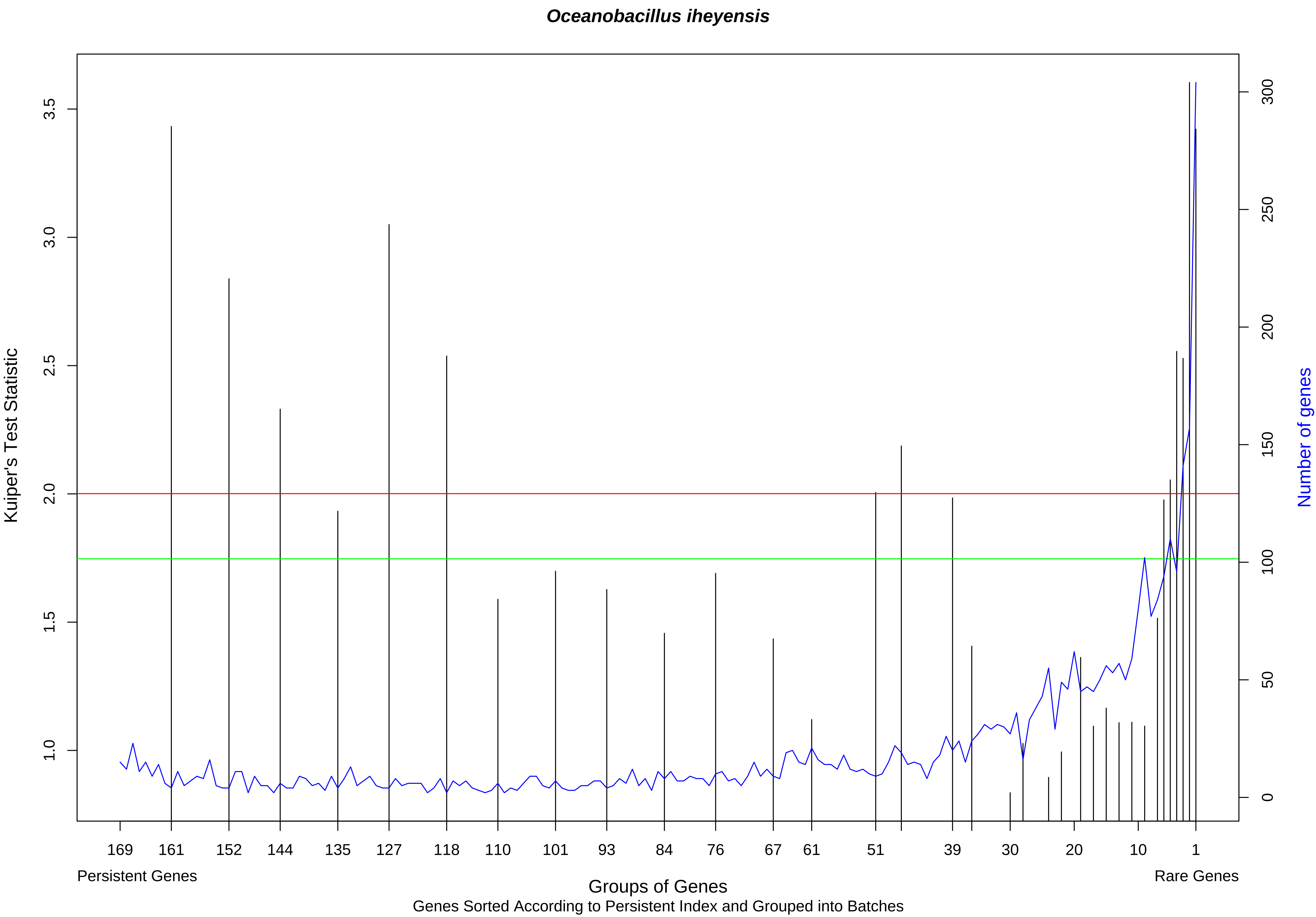

Oceanobacillus iheyensis
Kuiper's Test Statistic
Number of genes
1.0
1.5
2.0
2.5
3.0
3.5
0
50
100
150
200
250
300
169
161
152
144
135
127
118
110
101
93
84
76
67
61
51
39
30
20
10
1
Persistent Genes
Rare Genes
Groups of Genes
Genes Sorted According to Persistent Index and Grouped into Batches

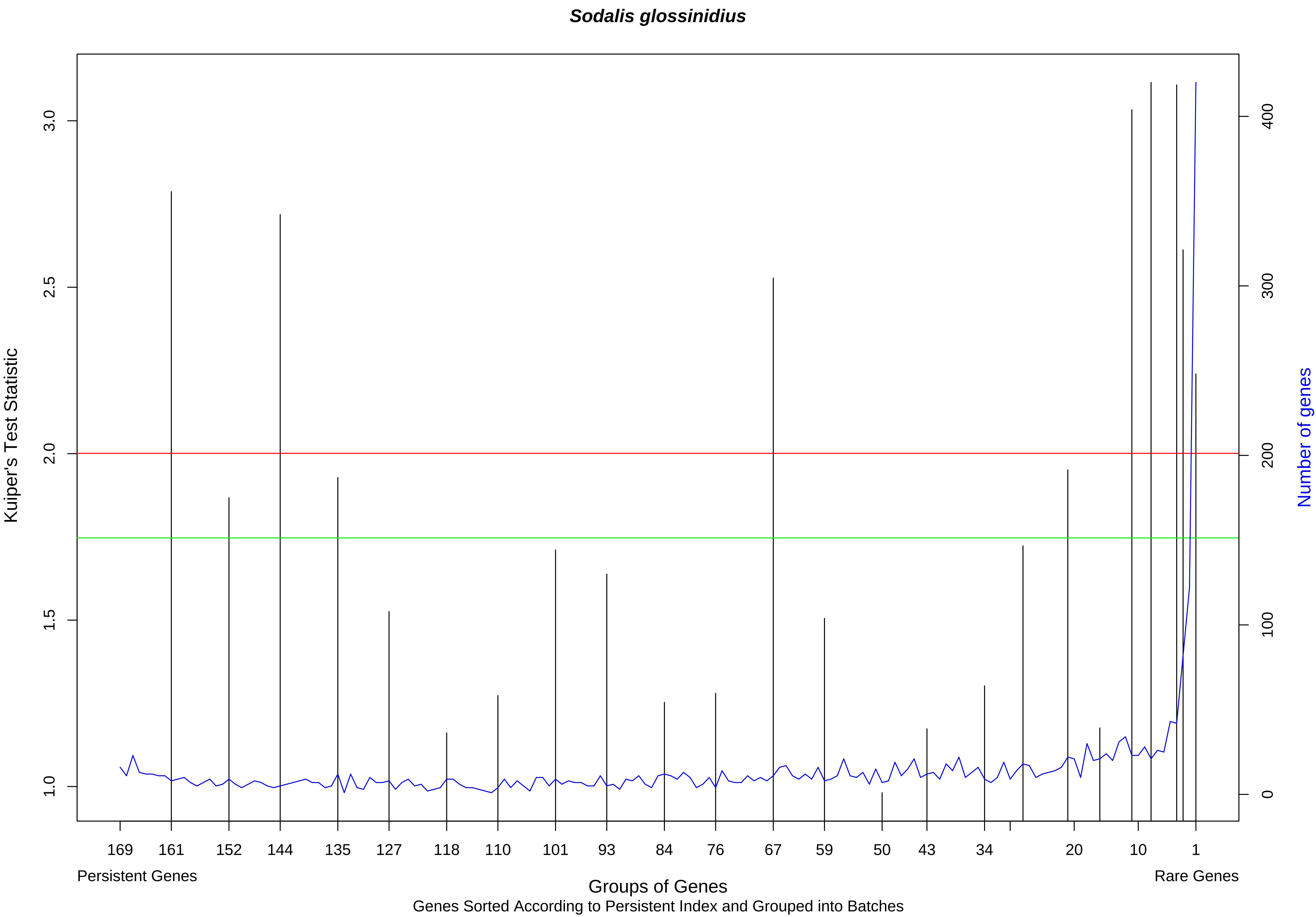
Sodalis glossinidius
Kuiper's Test Statistic
Number of genes
3.0
2.5
2.0
1.5
1.0
400
300
200
100
0
169
161
152
144
135
127
118
110
101
93
84
76
67
59
50
43
34
20
10
1
Persistent Genes
Rare Genes
Groups of Genes
Genes Sorted According to Persistent Index and Grouped into Batches

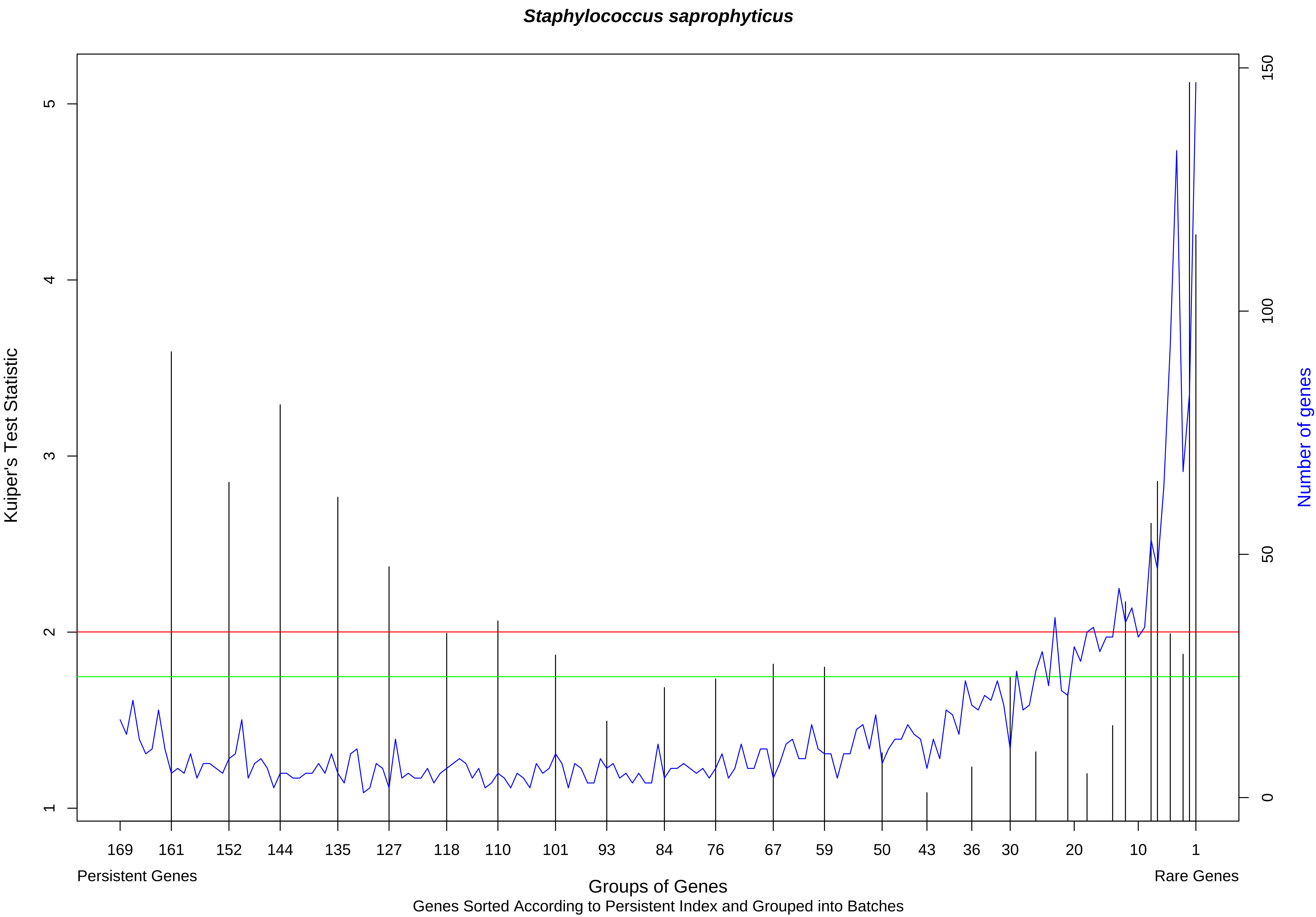
Staphylococcus saprophyticus
Kuiper's Test Statistic
Number of genes
1
2
3
4
5
0
50
100
150
169
161
152
144
135
127
118
110
101
93
84
76
67
59
50
43
36
30
20
10
1
Persistent Genes
Rare Genes
Groups of Genes
Genes Sorted According to Persistent Index and Grouped into Batches

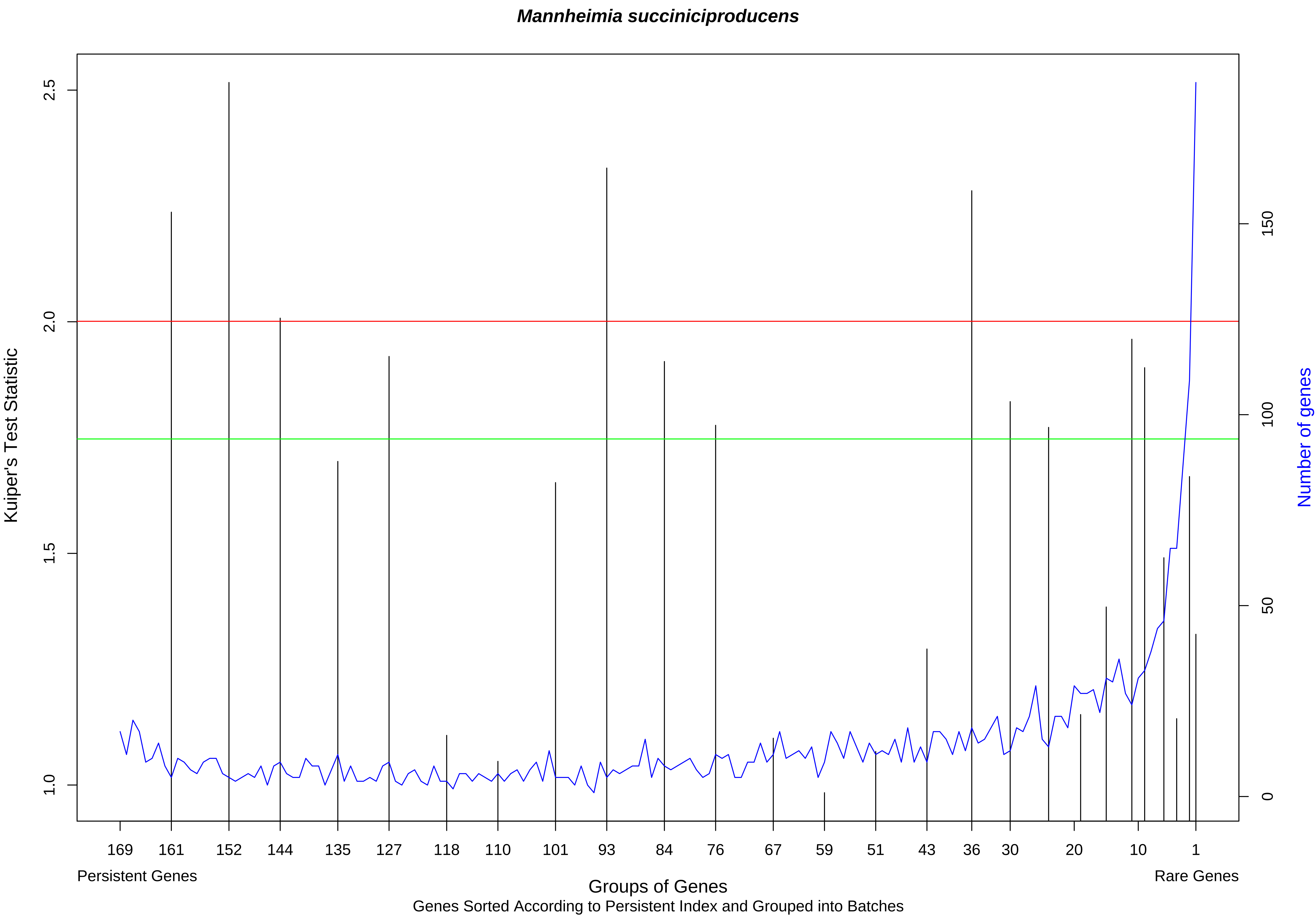

Mannheimia succiniciproducens
Kuiper's Test Statistic
Number of genes
2.5
2.0
1.5
1.0
150
100
50
0
169
161
152
144
135
127
118
110
101
93
84
76
67
59
51
43
36
30
20
10
1
Persistent Genes
Rare Genes
Groups of Genes
Genes Sorted According to Persistent Index and Grouped into Batches

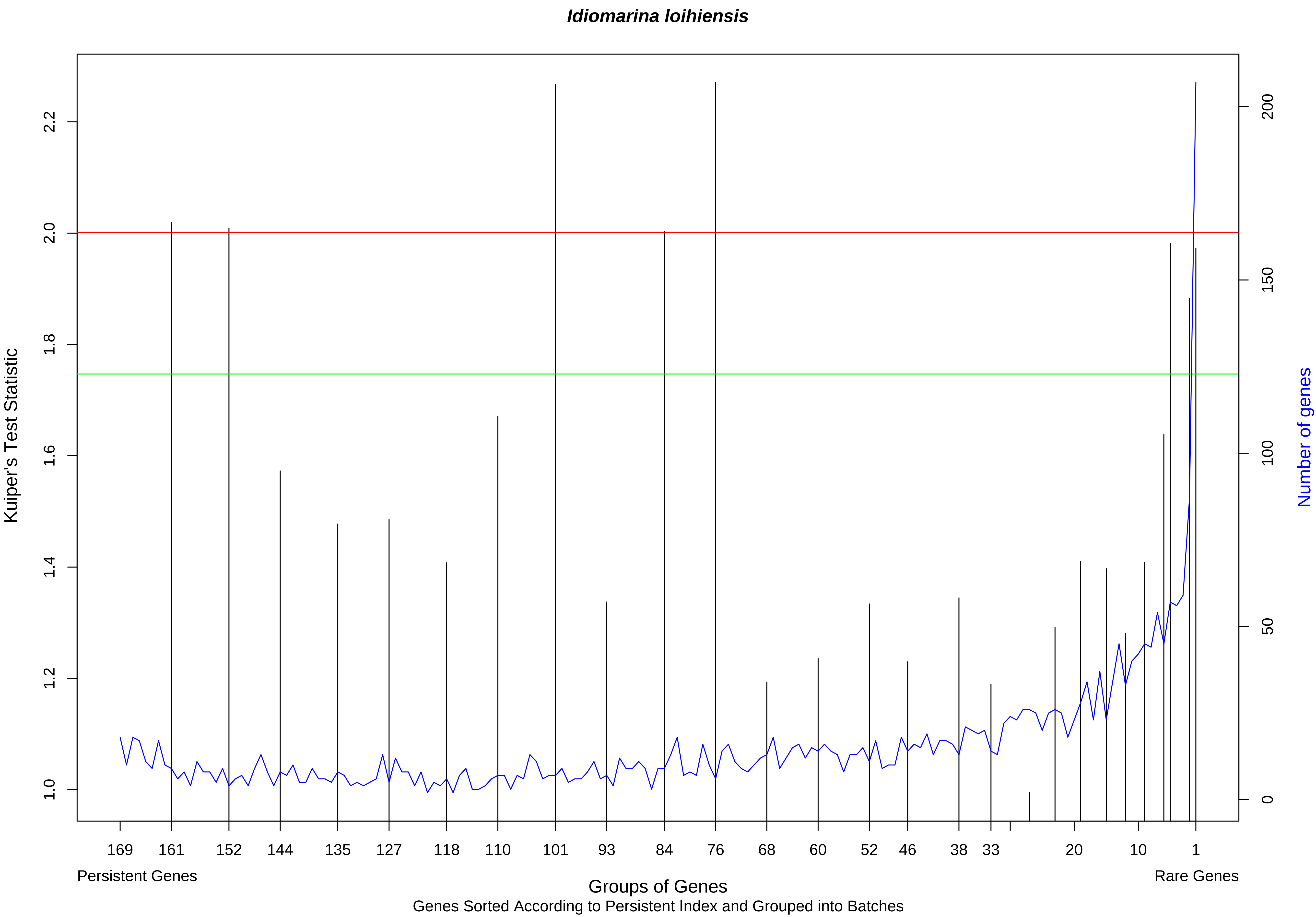
Idiomarina loihiensis
Kuiper's Test Statistic
Number of genes
2.2
2.0
1.8
1.6
1.4
1.2
1.0
200
150
100
50
0
169
161
152
144
135
127
118
110
101
93
84
76
68
60
52
46
38
33
20
10
1
Persistent Genes
Rare Genes
Groups of Genes
Genes Sorted According to Persistent Index and Grouped into Batches

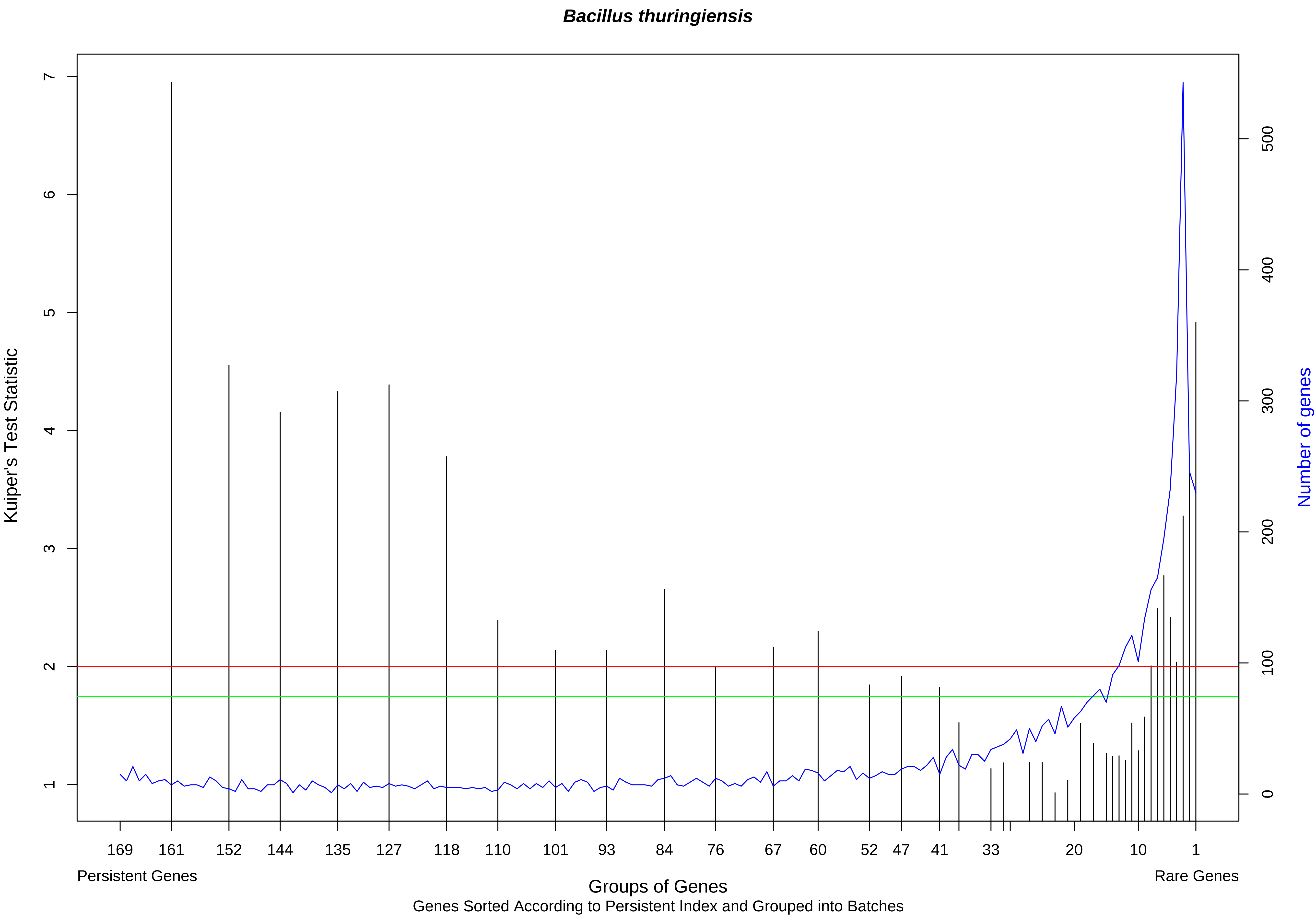
Bacillus thuringiensis
Kuiper's Test Statistic
Number of genes
1
2
3
4
5
6
7
0
100
200
300
400
500
169
161
152
144
135
127
118
110
101
93
84
76
67
60
52
47
41
33
20
10
1
Persistent Genes
Rare Genes
Groups of Genes
Genes Sorted According to Persistent Index and Grouped into Batches

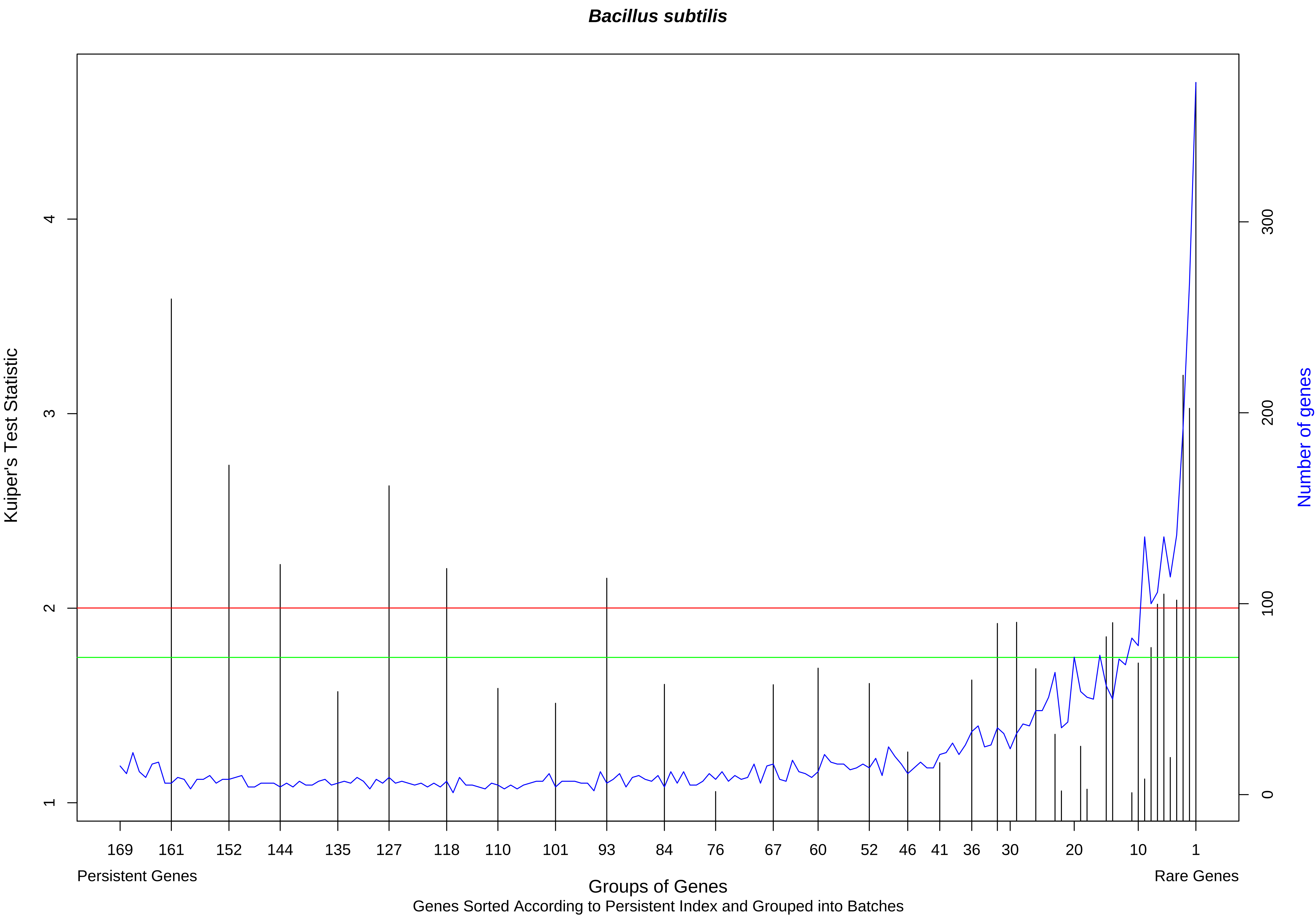
Bacillus subtilis
Kuiper's Test Statistic
1
2
3
4
Number of genes
0
100
200
300
169
161
152
144
135
127
118
110
101
93
84
76
67
60
52
46
41
36
30
20
10
1
Persistent Genes
Rare Genes
Groups of Genes
Genes Sorted According to Persistent Index and Grouped into Batches

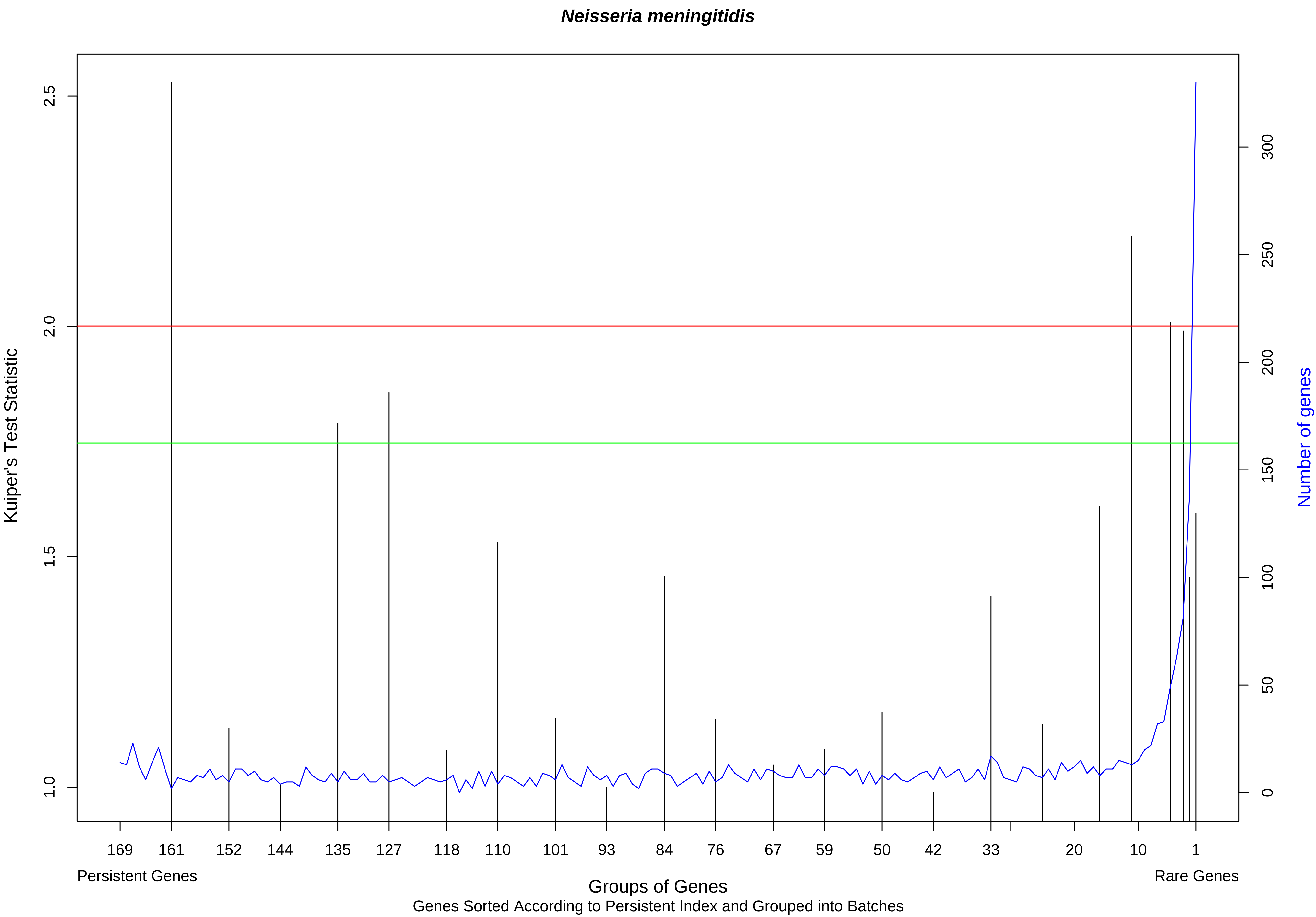

Neisseria meningitidis
Kuiper's Test Statistic
Number of genes
2.5
2.0
1.5
1.0
300
250
200
150
100
50
0
169
161
152
144
135
127
118
110
101
93
84
76
67
59
50
42
33
20
10
1
Persistent Genes
Rare Genes
Groups of Genes
Genes Sorted According to Persistent Index and Grouped into Batches

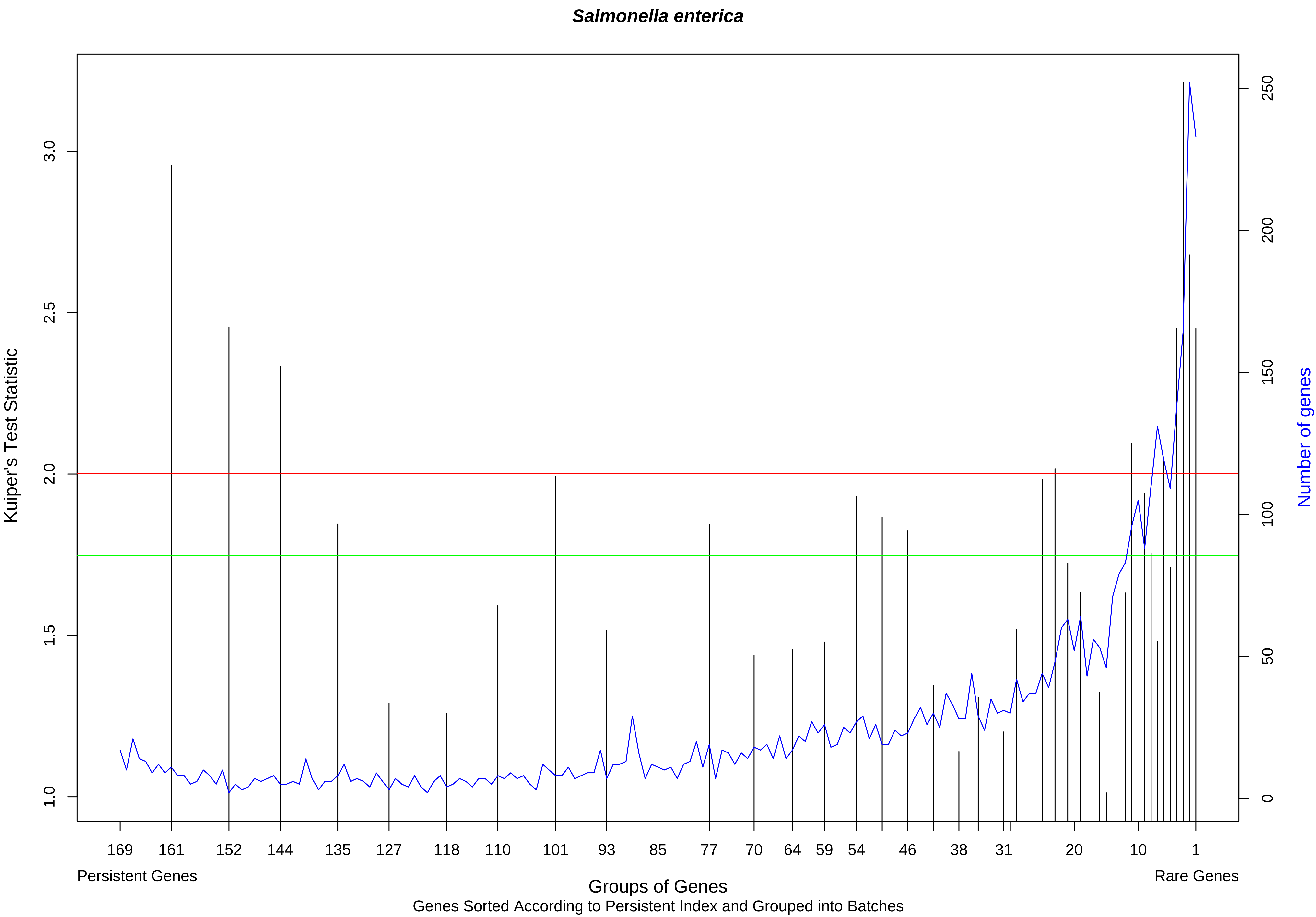

Salmonella enterica
Kuiper's Test Statistic
1.0
1.5
2.0
2.5
3.0
Number of genes
0
50
100
150
200
250
169
161
152
144
135
127
118
110
101
93
85
77
70
64
59
54
46
38
31
20
10
1
Persistent Genes
Rare Genes
Groups of Genes
Genes Sorted According to Persistent Index and Grouped into Batches

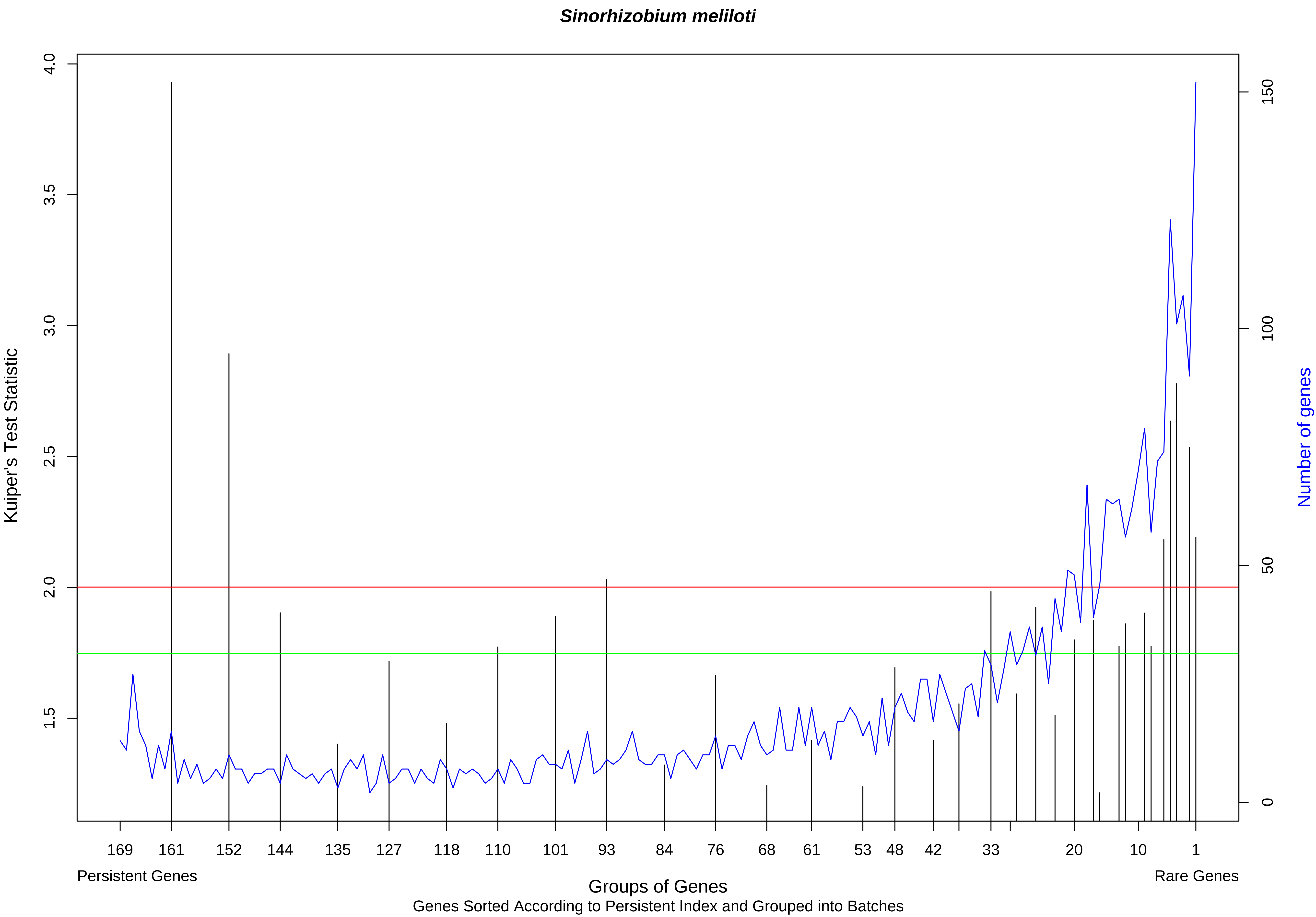

Sinorhizobium meliloti
Kuiper's Test Statistic
1.5
2.0
2.5
3.0
3.5
4.0
Number of genes
0
50
100
150
169
161
152
144
135
127
118
110
101
93
84
76
68
61
53
48
42
33
20
10
1
Persistent Genes
Rare Genes
Groups of Genes
Genes Sorted According to Persistent Index and Grouped into Batches

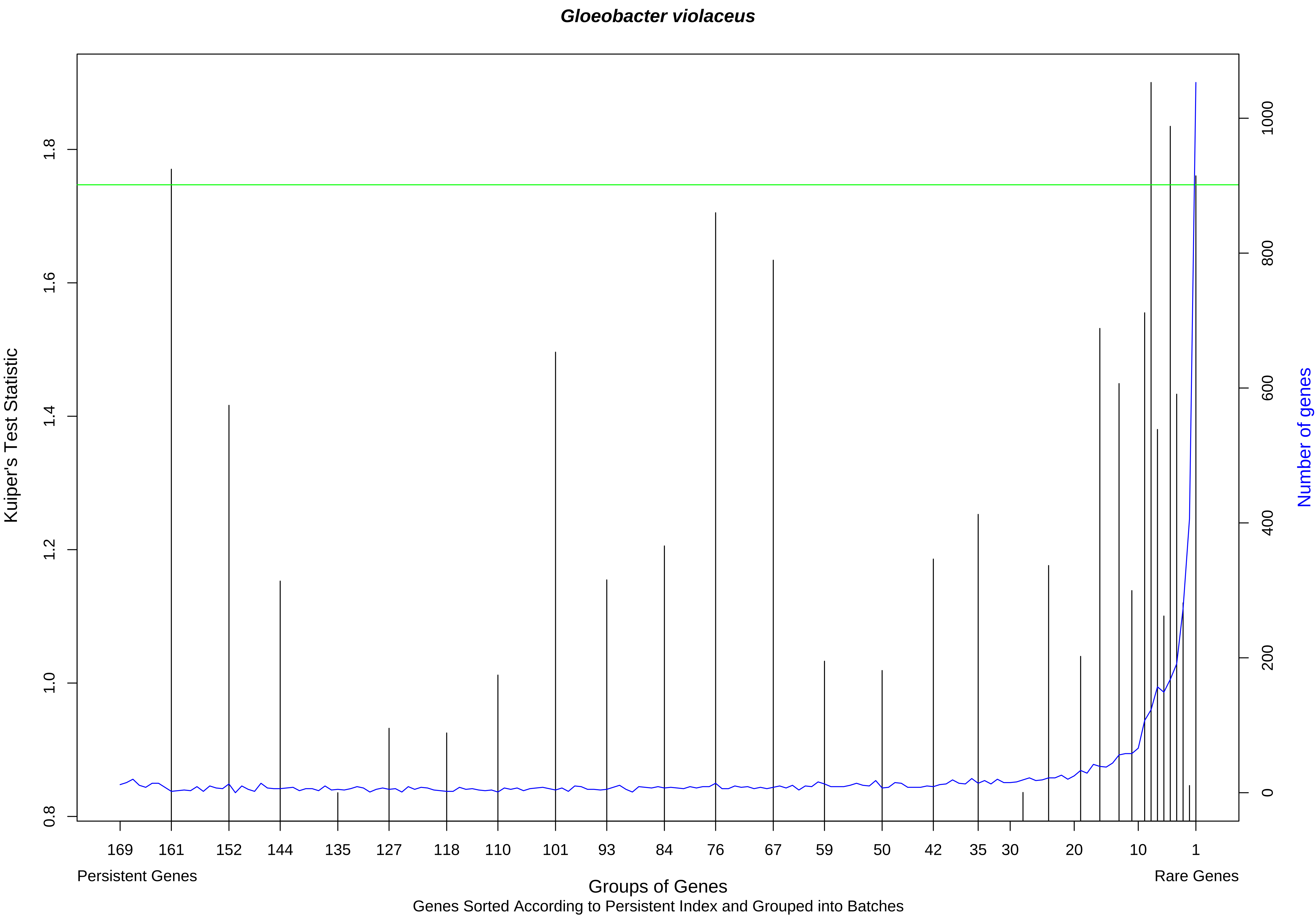

Gloeobacter violaceus
Kuiper's Test Statistic
0.8
1.0
1.2
1.4
1.6
1.8
Number of genes
0
200
400
600
800
1000
169
161
152
144
135
127
118
110
101
93
84
76
67
59
50
42
35
30
20
10
1
Persistent Genes
Rare Genes
Groups of Genes
Genes Sorted According to Persistent Index and Grouped into Batches

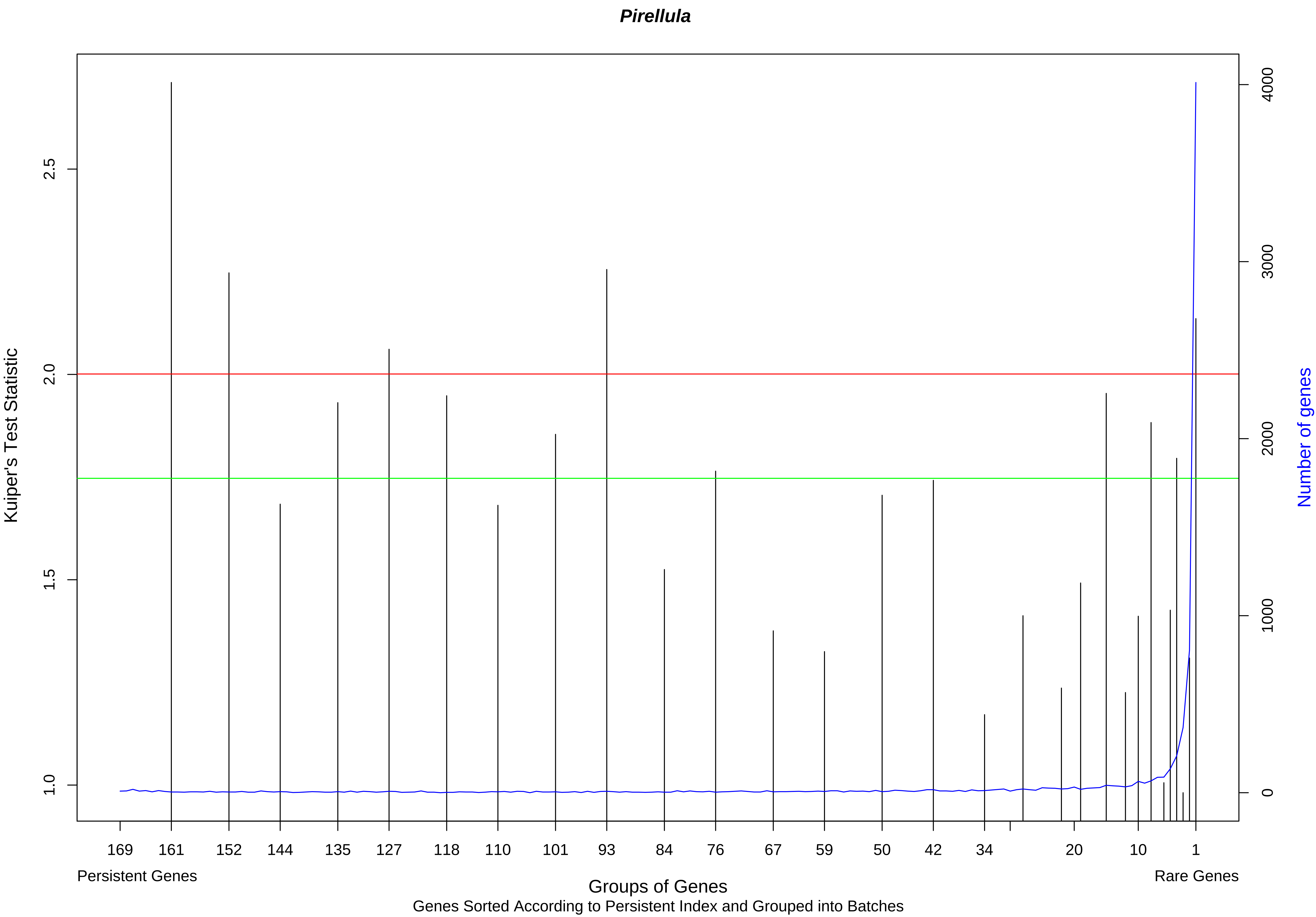

Pirellula
Kuiper's Test Statistic
Number of genes
1.0
1.5
2.0
2.5
0
1000
2000
3000
4000
169
161
152
144
135
127
118
110
101
93
84
76
67
59
50
42
34
20
10
1
Persistent Genes
Rare Genes
Groups of Genes
Genes Sorted According to Persistent Index and Grouped into Batches

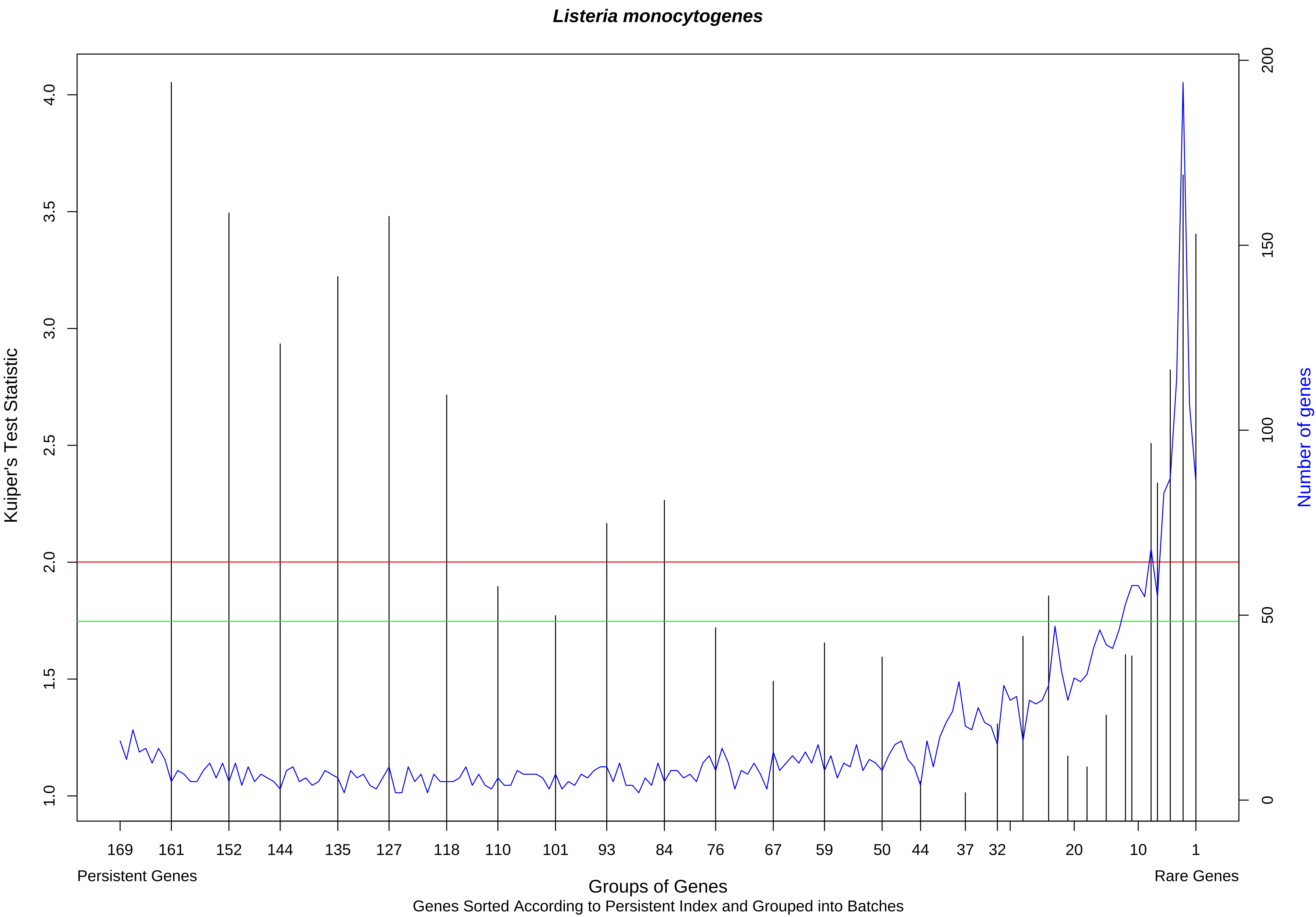

Listeria monocytogenes
Kuiper's Test Statistic
1.0
1.5
2.0
2.5
3.0
3.5
4.0
Number of genes
0
50
100
150
200
169
161
152
144
135
127
118
110
101
93
84
76
67
59
50
44
37
32
20
10
1
Persistent Genes
Rare Genes
Groups of Genes
Genes Sorted According to Persistent Index and Grouped into Batches

Listeria innocua

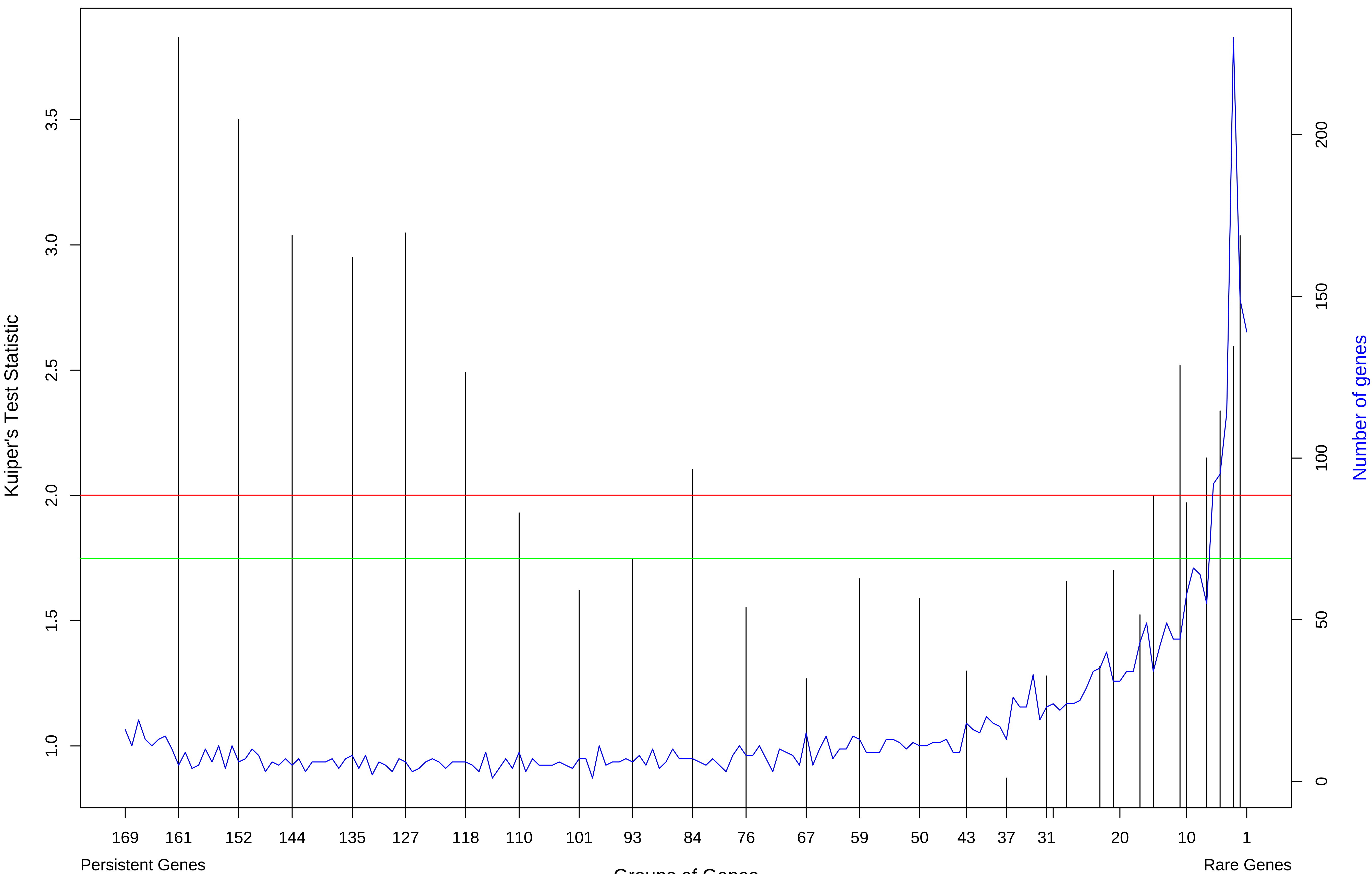

Groups of Genes
Genes Sorted According to Persistent Index and Grouped into Batches

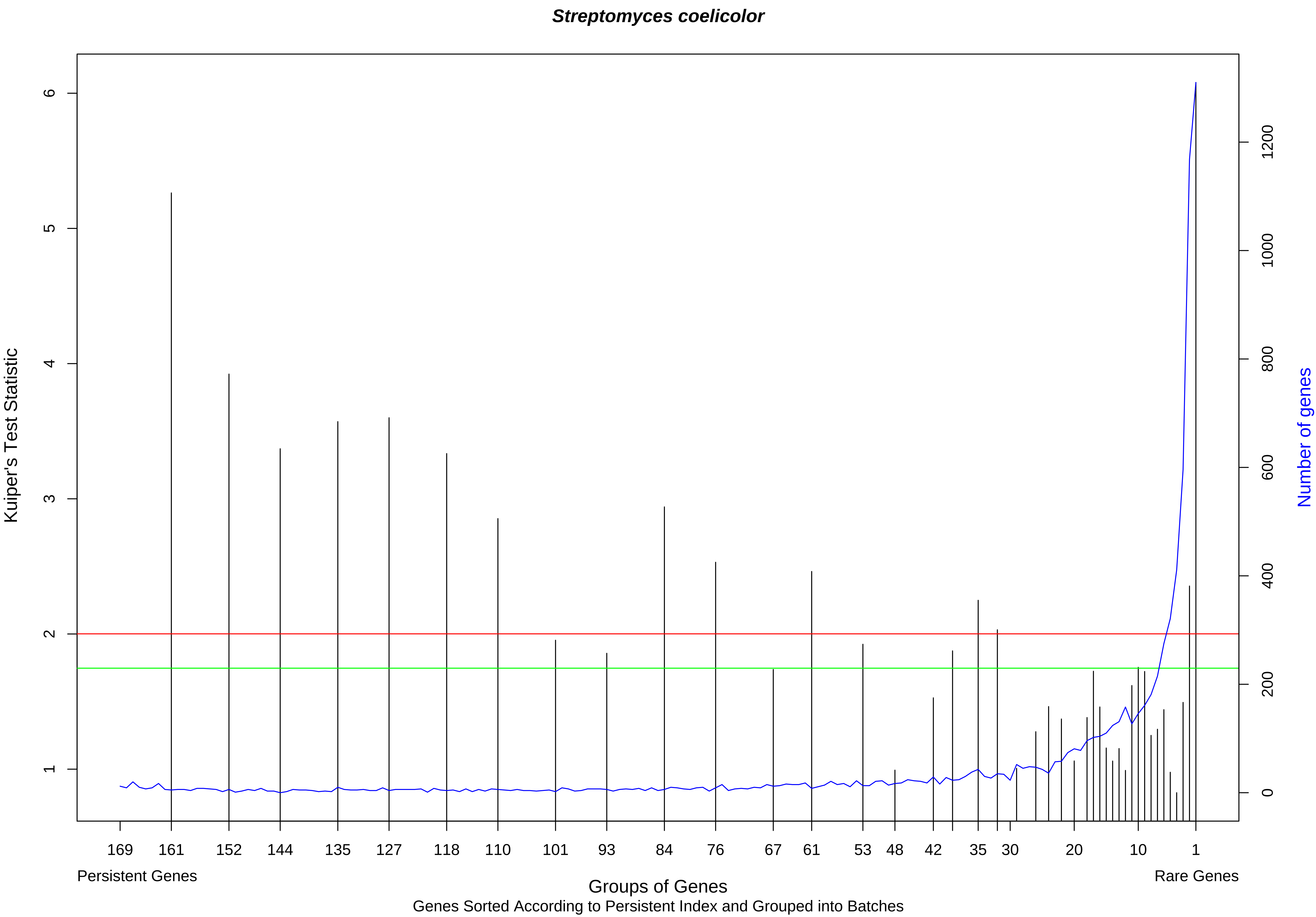

Streptomyces coelicolor
Kuiper's Test Statistic
Number of genes
1
2
3
4
5
6
0
200
400
600
800
1000
1200
169 161 152 144 135 127 118 110 101 93 84 76 67 61 53 48 42 35 30 20 10 1
Persistent Genes
Rare Genes
Groups of Genes
Genes Sorted According to Persistent Index and Grouped into Batches

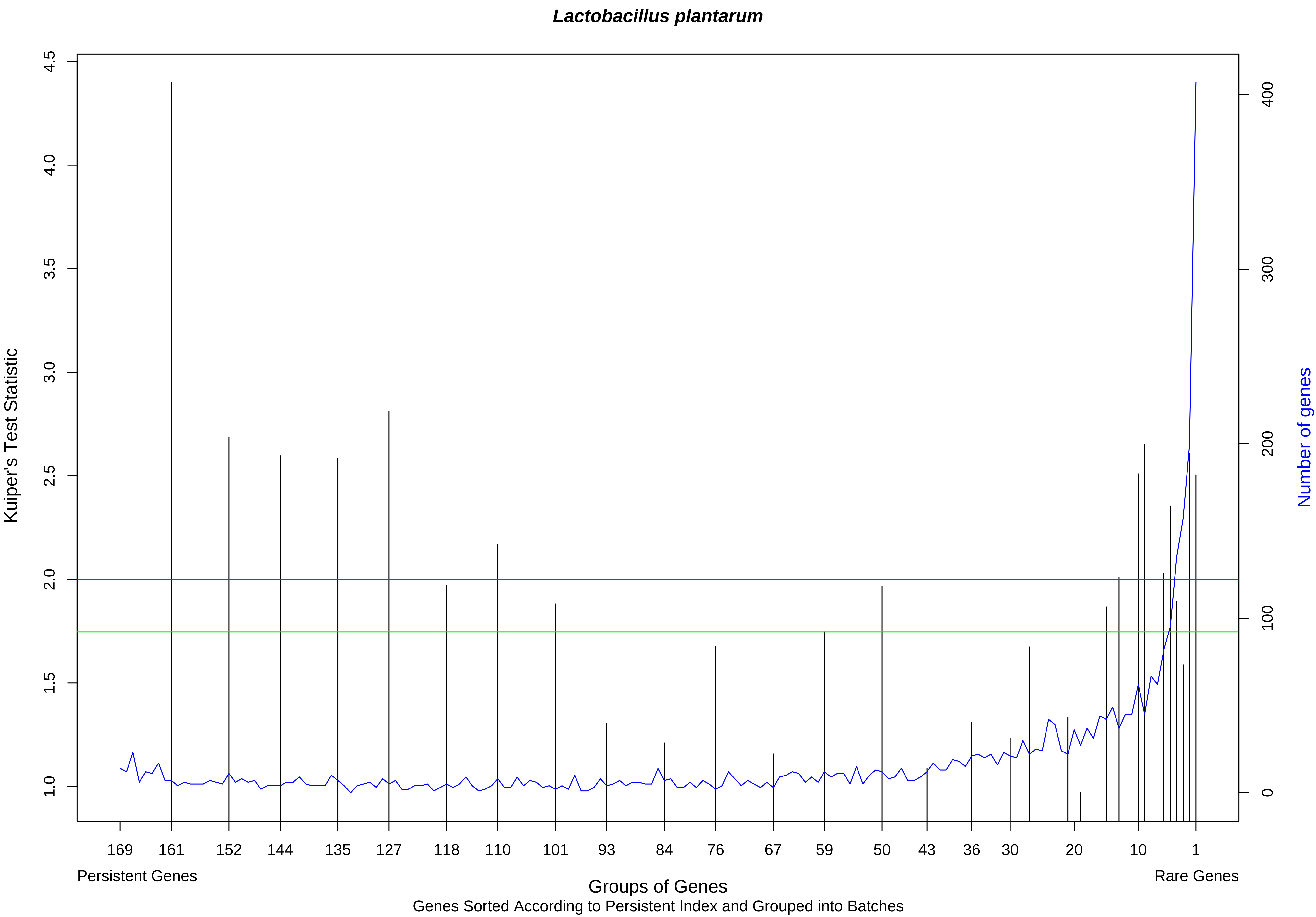

Lactobacillus plantarum
Kuiper's Test Statistic
Number of genes
1.0
1.5
2.0
2.5
3.0
3.5
4.0
4.5
0
100
200
300
400
169
161
152
144
135
127
118
110
101
93
84
76
67
59
50
43
36
30
20
10
1
Persistent Genes
Rare Genes
Groups of Genes
Genes Sorted According to Persistent Index and Grouped into Batches

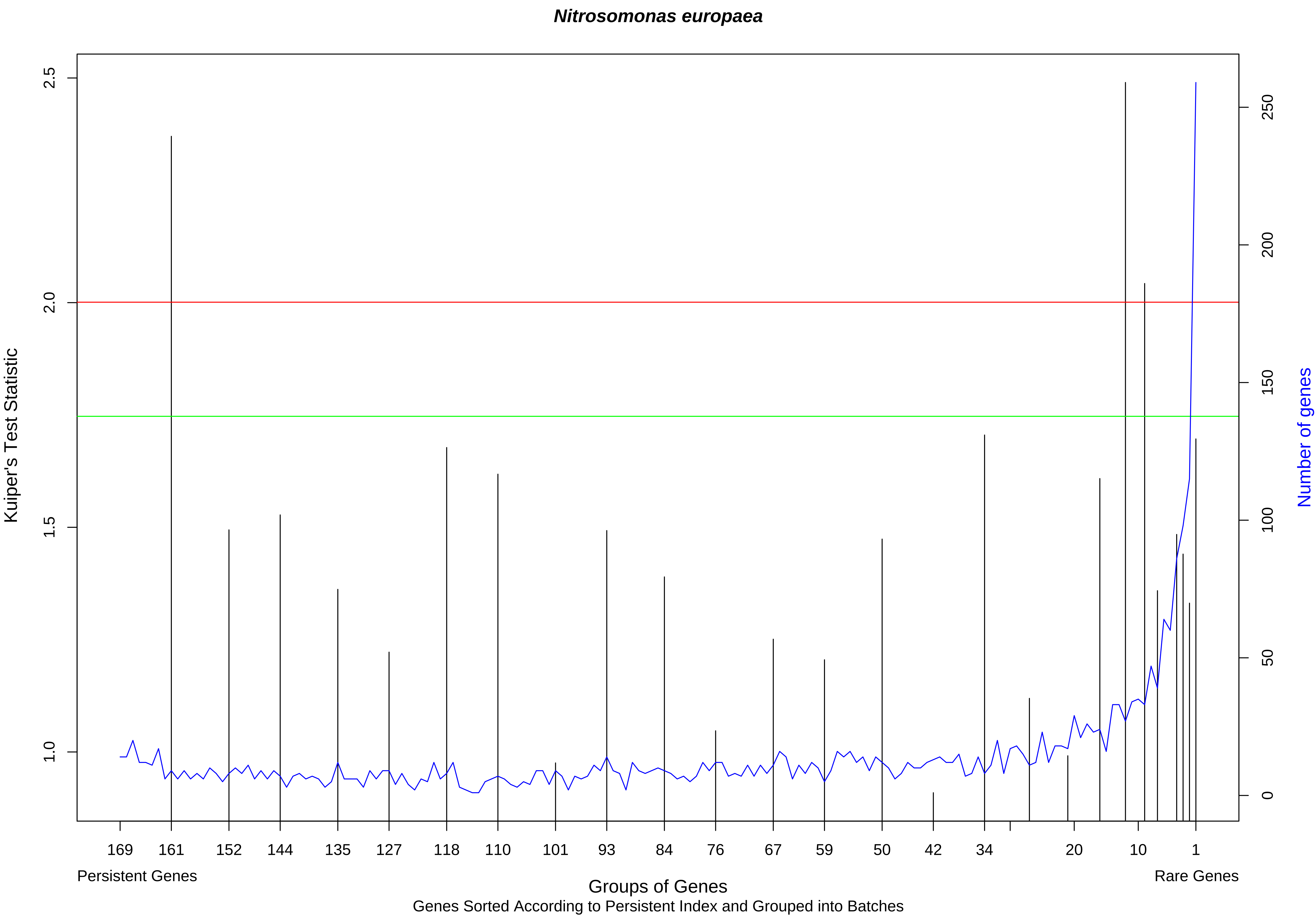

Nitrosomonas europaea
Kuiper's Test Statistic
2.5
2.0
1.5
1.0
Number of genes
250
200
150
100
50
0
169
161
152
144
135
127
118
110
101
93
84
76
67
59
50
42
34
20
10
1
Persistent Genes
Rare Genes
Groups of Genes
Genes Sorted According to Persistent Index and Grouped into Batches

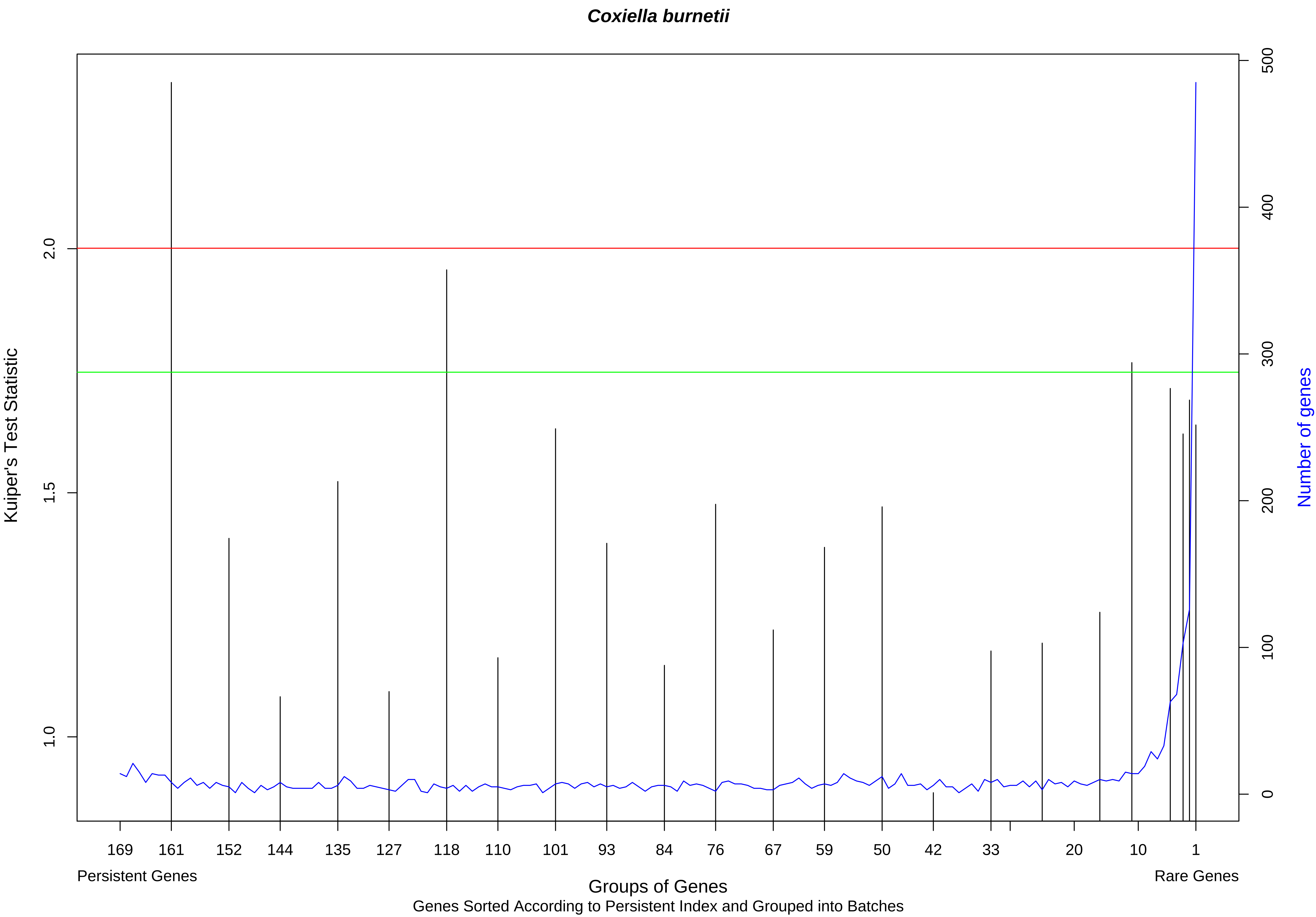
Coxiella burnetii
Kuiper's Test Statistic
Number of genes
2.0
1.5
1.0
500
400
300
200
100
0
169
161
152
144
135
127
118
110
101
93
84
76
67
59
50
42
33
20
10
1
Persistent Genes
Rare Genes
Groups of Genes
Genes Sorted According to Persistent Index and Grouped into Batches

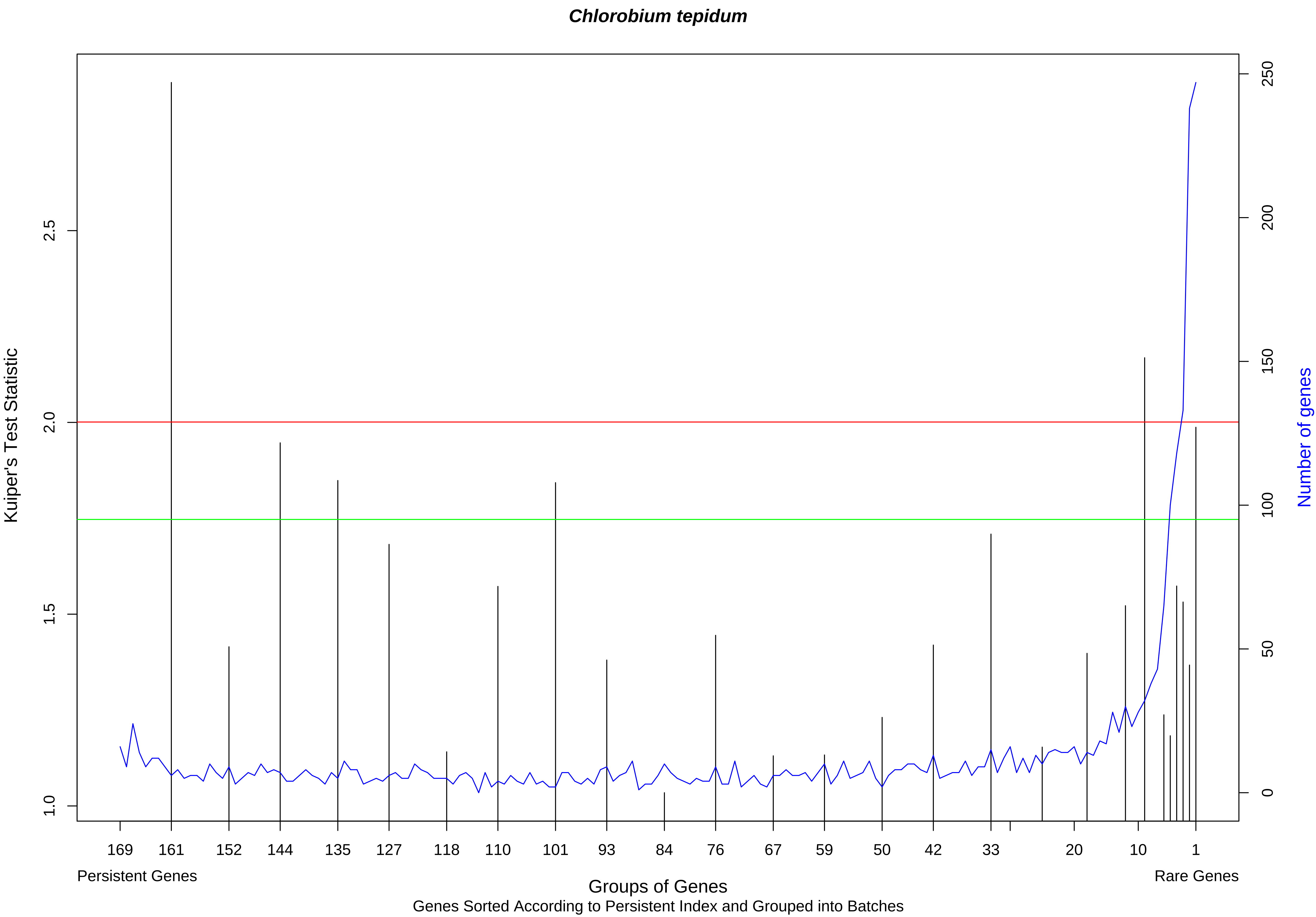

Chlorobium tepidum
Kuiper's Test Statistic
Number of genes
1.0
1.5
2.0
2.5
0
50
100
150
200
250
169
161
152
144
135
127
118
110
101
93
84
76
67
59
50
42
33
20
10
1
Persistent Genes
Rare Genes
Groups of Genes
Genes Sorted According to Persistent Index and Grouped into Batches

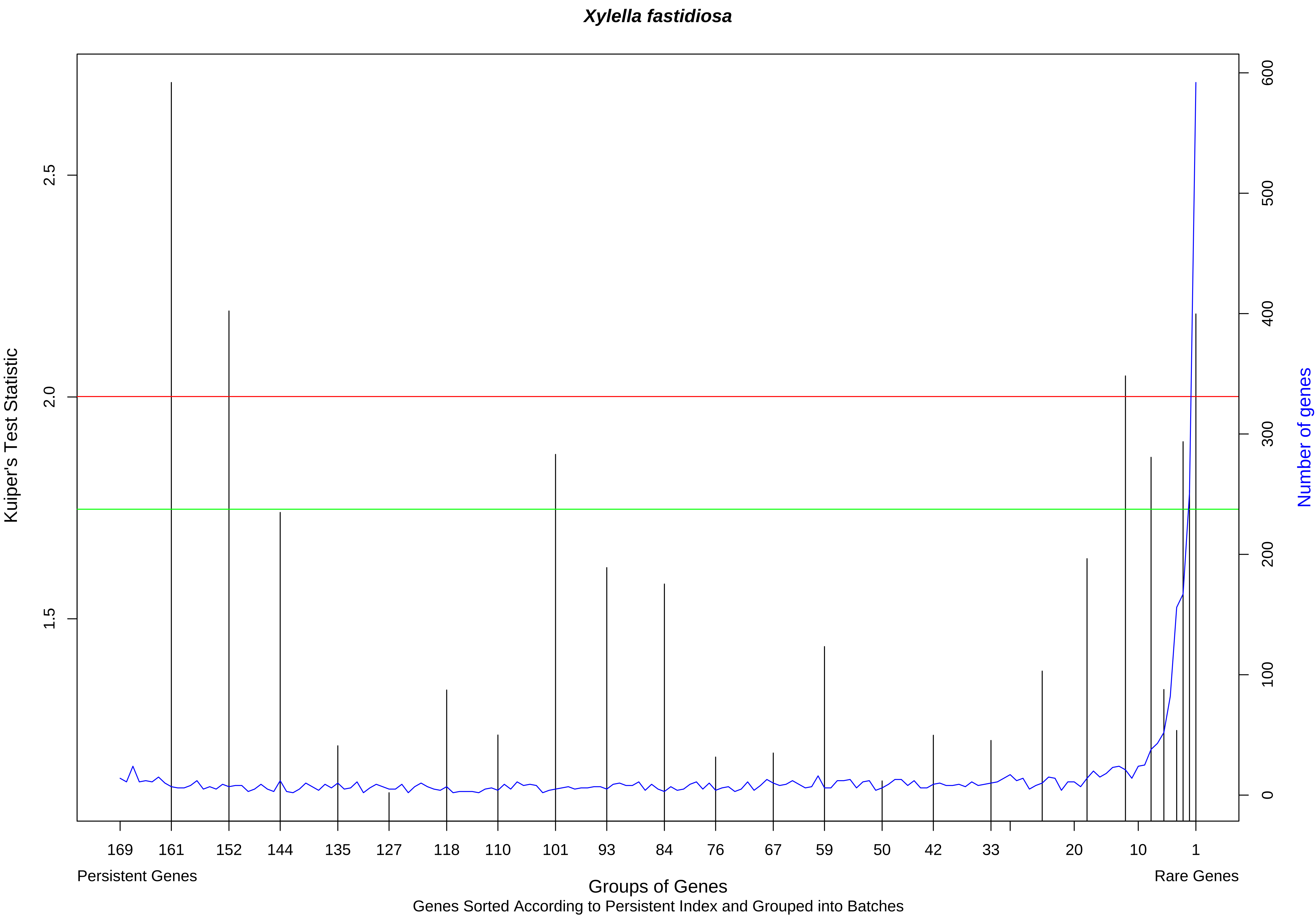

Xylella fastidiosa
Kuiper's Test Statistic
2.5
2.0
1.5
Number of genes
600
500
400
300
200
100
0
169
161
152
144
135
127
118
110
101
93
84
76
67
59
50
42
33
20
10
1
Persistent Genes
Rare Genes
Groups of Genes
Genes Sorted According to Persistent Index and Grouped into Batches

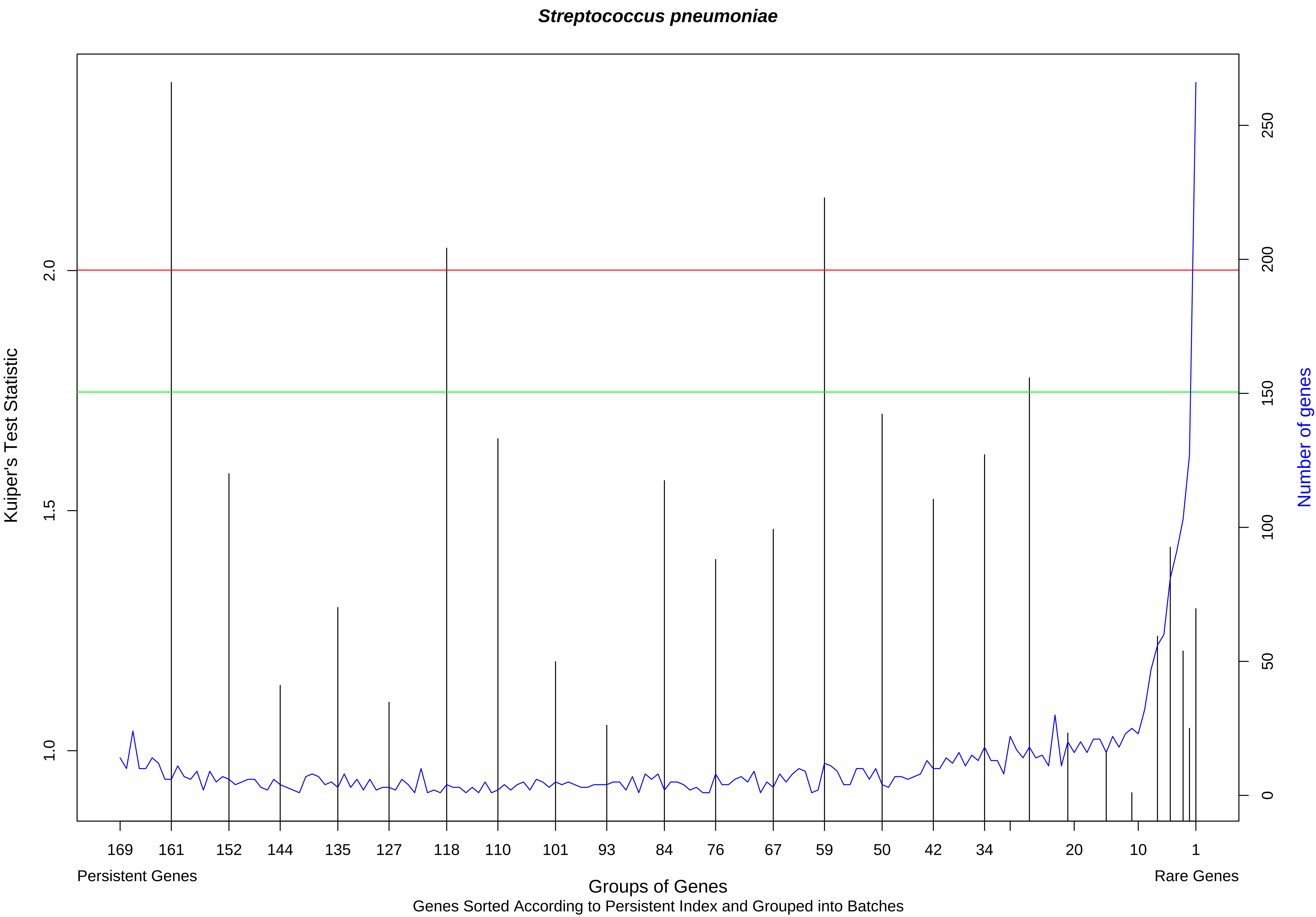

Streptococcus pneumoniae
Kuiper's Test Statistic
2.0
1.5
1.0
Number of genes
250
200
150
100
50
0
169
161
152
144
135
127
118
110
101
93
84
76
67
59
50
42
34
20
10
1
Persistent Genes
Rare Genes
Groups of Genes
Genes Sorted According to Persistent Index and Grouped into Batches

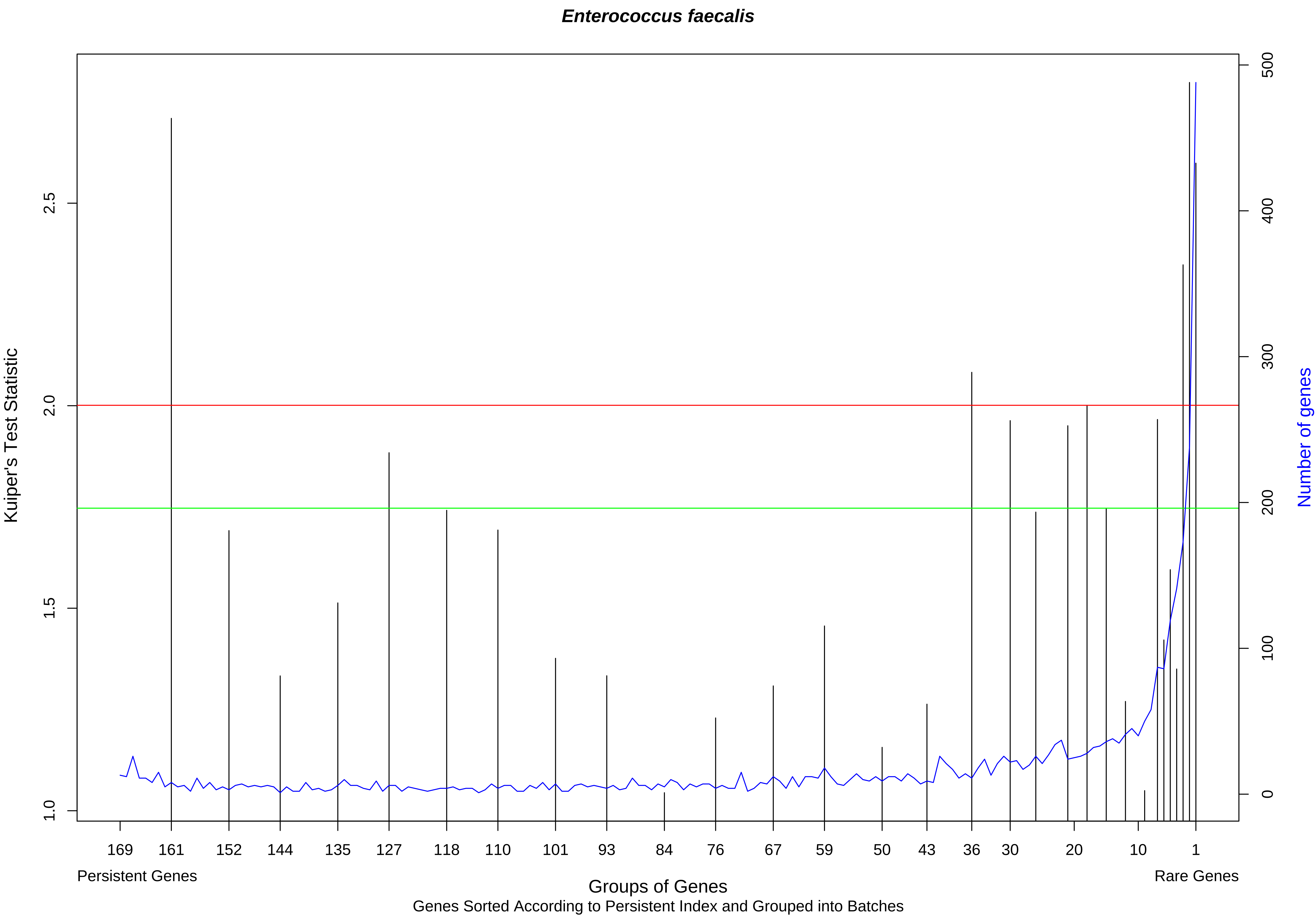

Enterococcus faecalis
Kuiper's Test Statistic
1.0
1.5
2.0
2.5
Number of genes
0
100
200
300
400
500
169
161
152
144
135
127
118
110
101
93
84
76
67
59
50
43
36
30
20
10
1
Persistent Genes
Rare Genes
Groups of Genes
Genes Sorted According to Persistent Index and Grouped into Batches

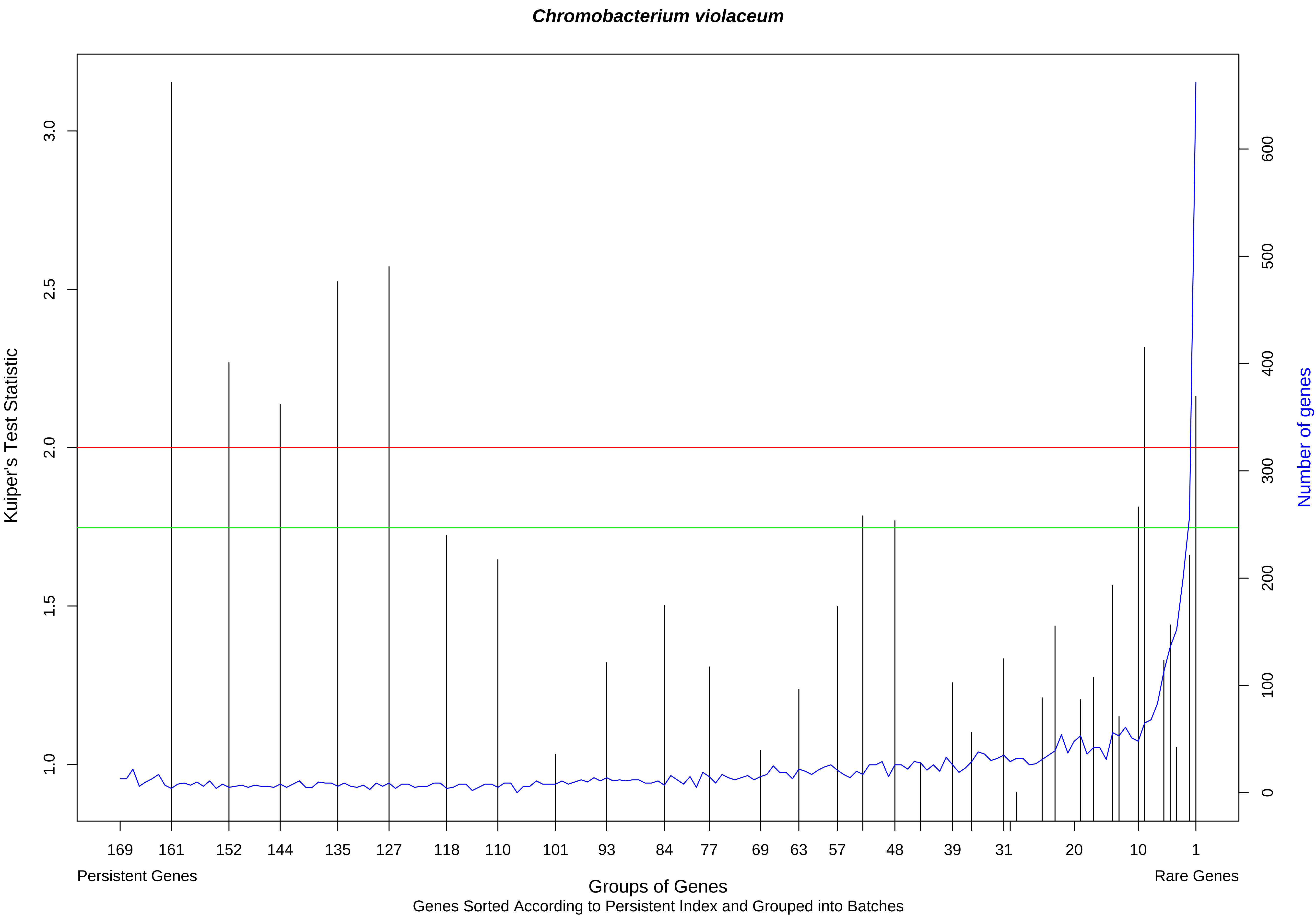

Chromobacterium violaceum
Kuiper's Test Statistic
Number of genes
3.0
2.5
2.0
1.5
1.0
600
500
400
300
200
100
0
169
161
152
144
135
127
118
110
101
93
84
77
69
63
57
48
39
31
20
10
1
Persistent Genes
Rare Genes
Groups of Genes
Genes Sorted According to Persistent Index and Grouped into Batches

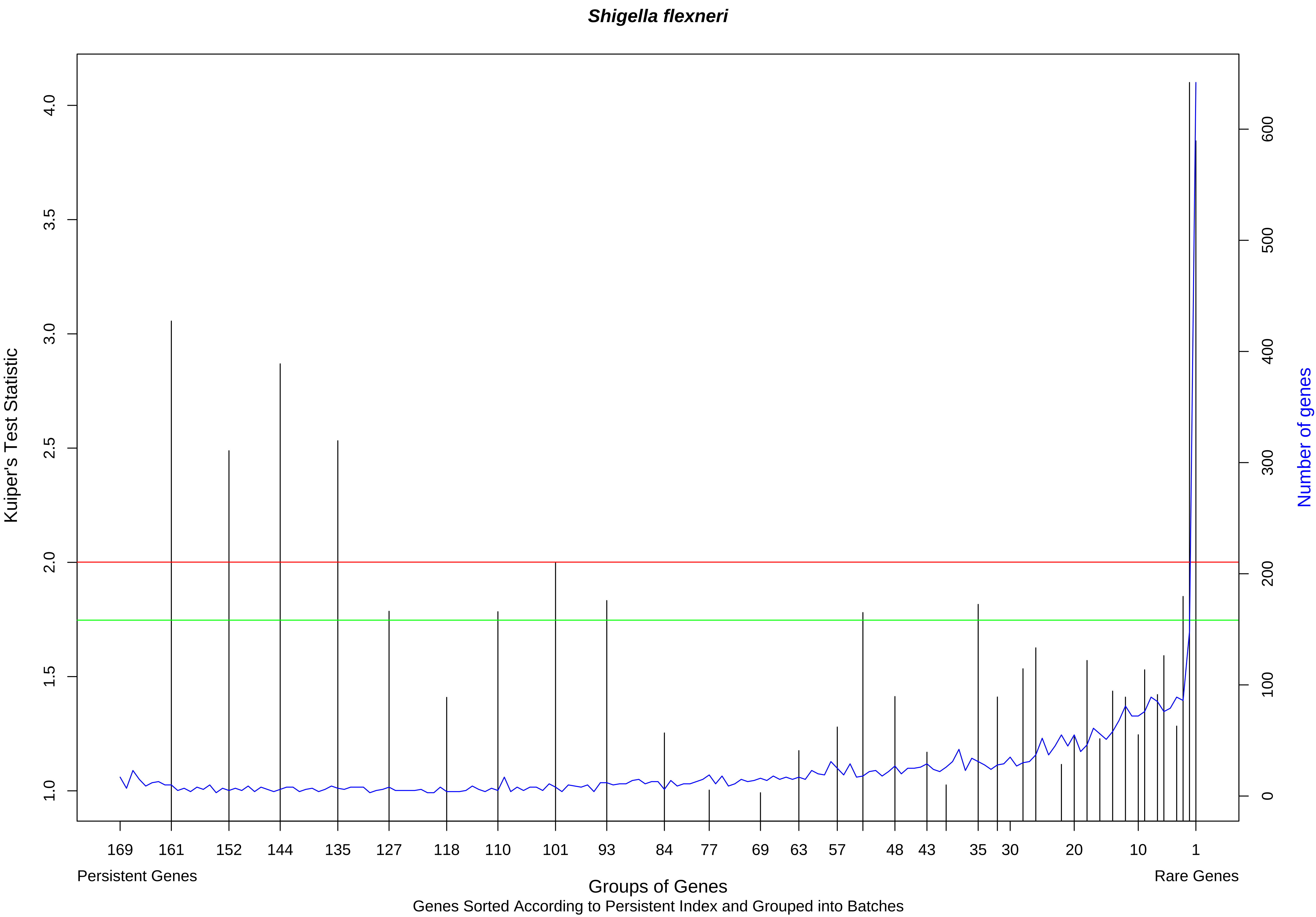
Shigella flexneri
Kuiper's Test Statistic
Number of genes
1.0
1.5
2.0
2.5
3.0
3.5
4.0
0
100
200
300
400
500
600
169
161
152
144
135
127
118
110
101
93
84
77
69
63
57
48
43
35
30
20
10
1
Persistent Genes
Rare Genes
Groups of Genes
Genes Sorted According to Persistent Index and Grouped into Batches

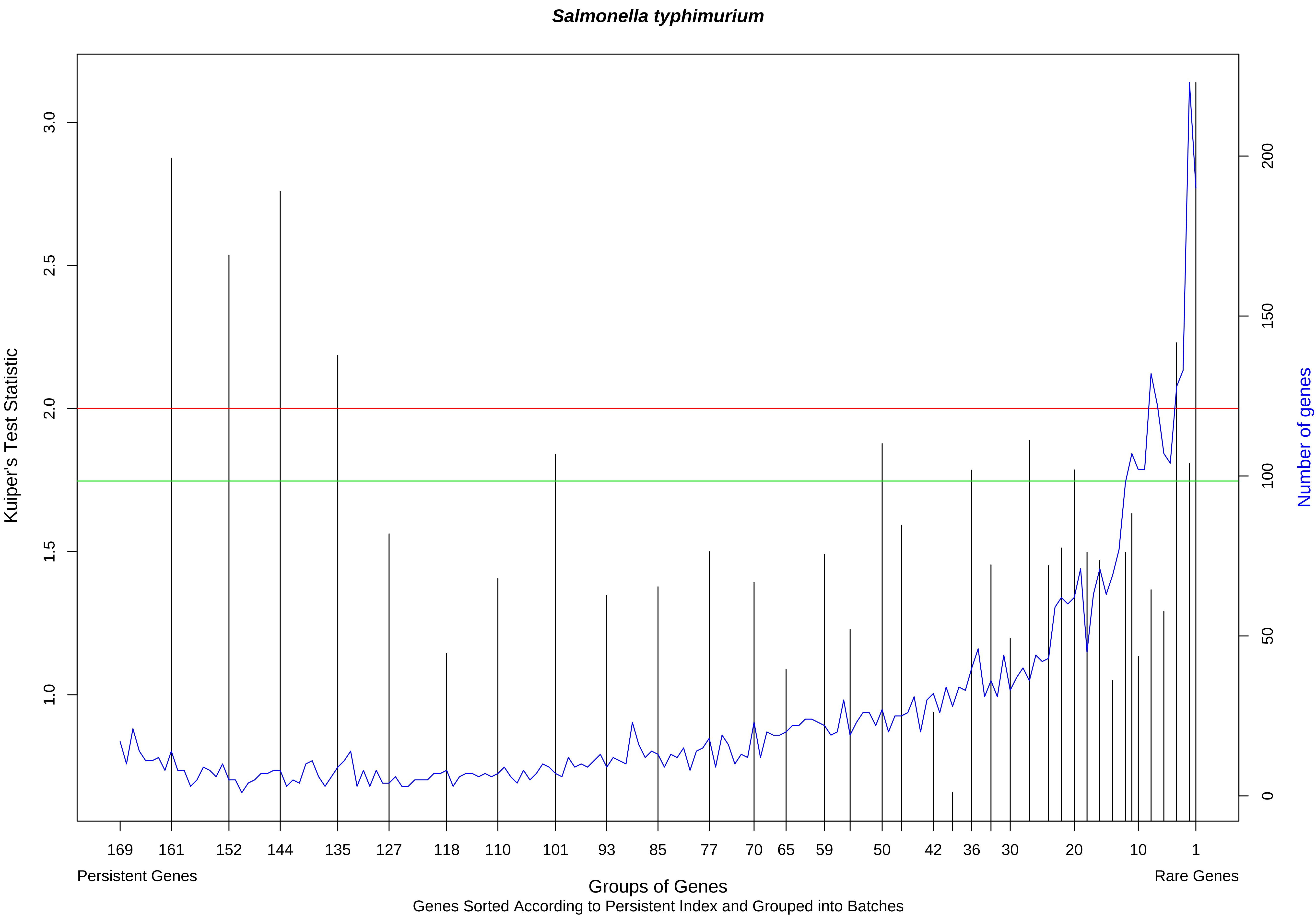
Salmonella typhimurium
Kuiper's Test Statistic
3.0
2.5
2.0
1.5
1.0
Number of genes
200
150
100
50
0
169
161
152
144
135
127
118
110
101
93
85
77
70
65
59
50
42
36
30
20
10
1
Persistent Genes
Rare Genes
Groups of Genes
Genes Sorted According to Persistent Index and Grouped into Batches

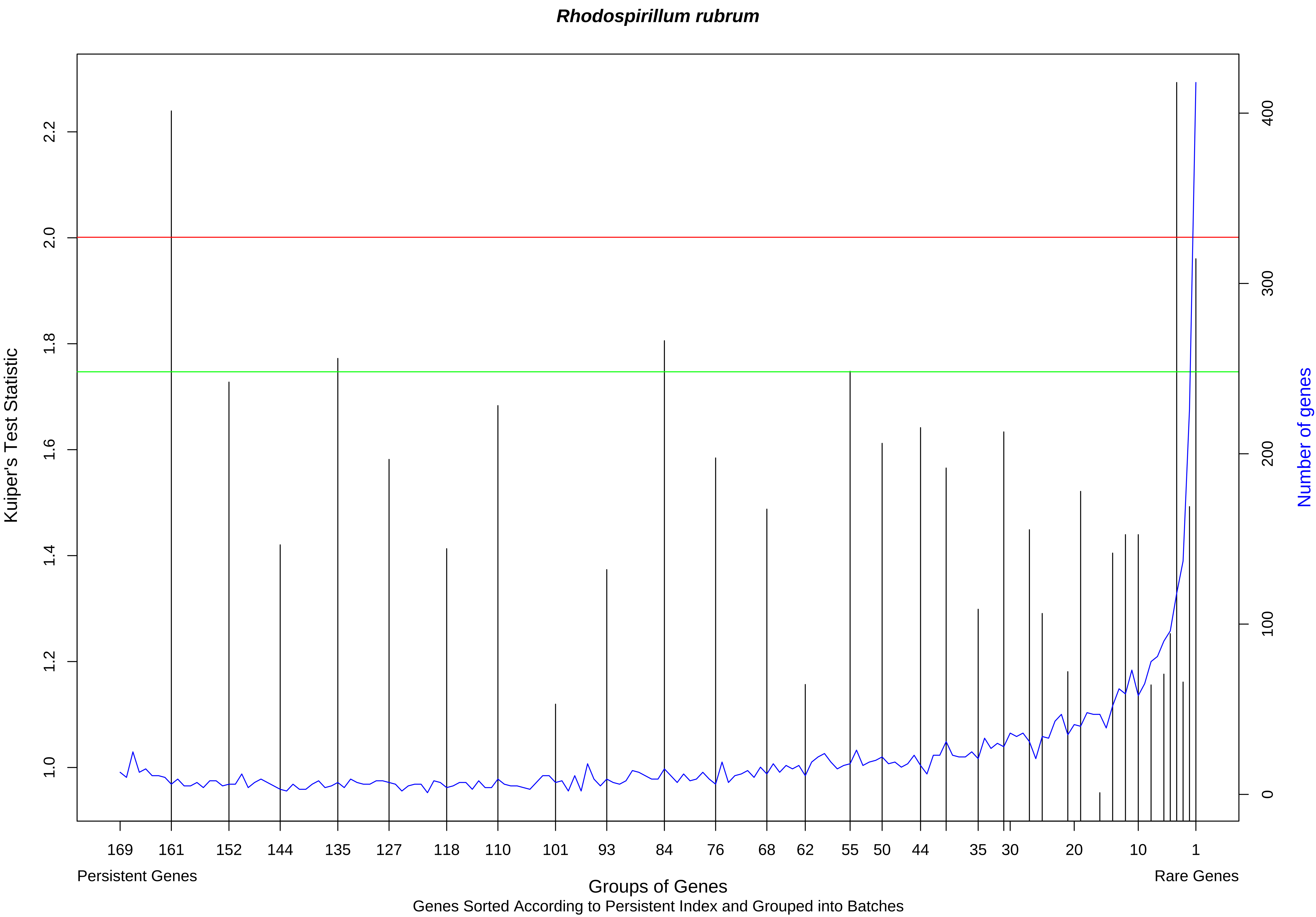

Rhodospirillum rubrum
Kuiper's Test Statistic
Number of genes
1.0
1.2
1.4
1.6
1.8
2.0
2.2
0
100
200
300
400
169
161
152
144
135
127
118
110
101
93
84
76
68
62
55
50
44
35
30
20
10
1
Persistent Genes
Rare Genes
Groups of Genes
Genes Sorted According to Persistent Index and Grouped into Batches

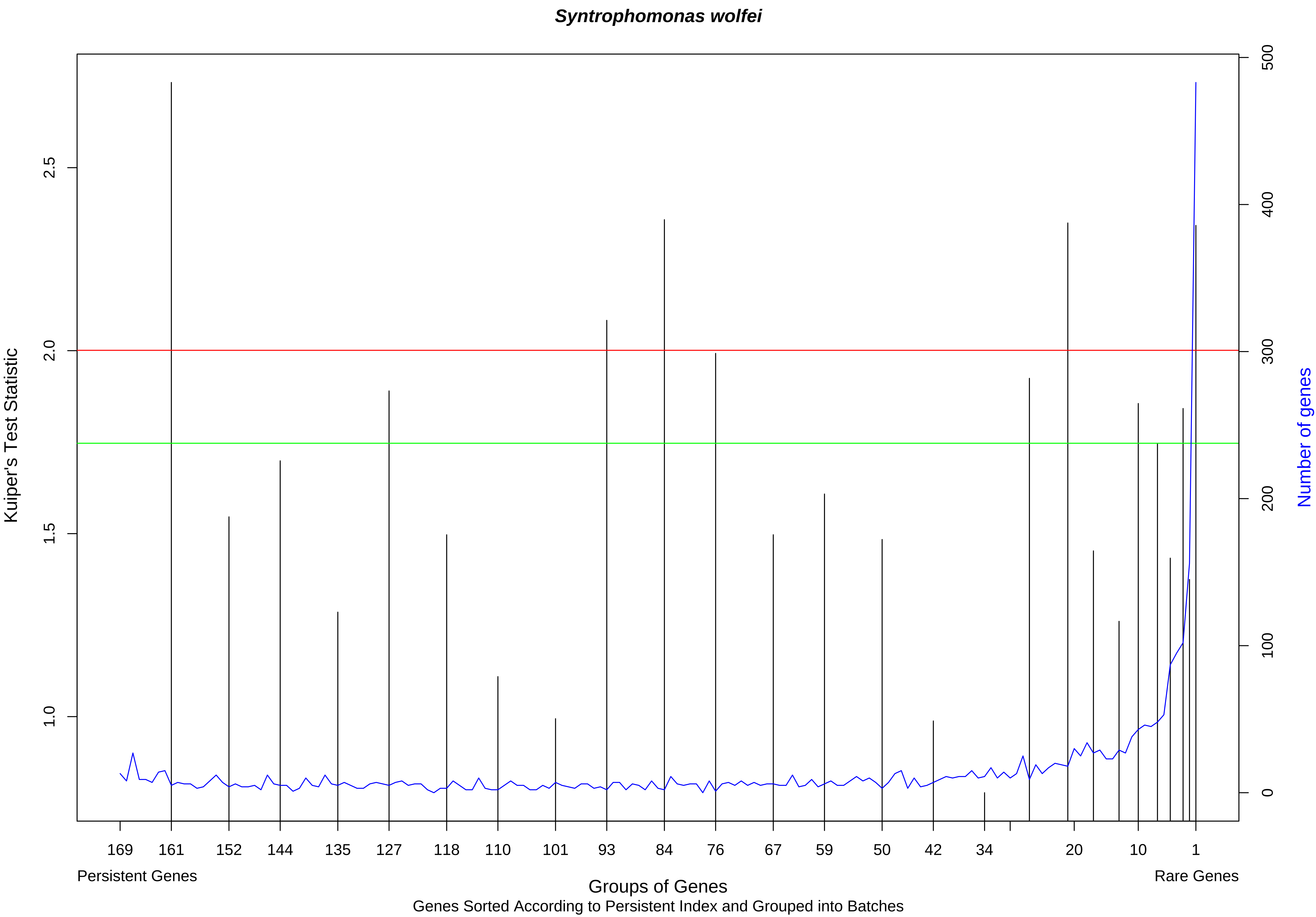

Syntrophomonas wolfei
Kuiper's Test Statistic
Number of genes
2.5
2.0
1.5
1.0
500
400
300
200
100
0
169
161
152
144
135
127
118
110
101
93
84
76
67
59
50
42
34
20
10
1
Persistent Genes
Rare Genes
Groups of Genes
Genes Sorted According to Persistent Index and Grouped into Batches

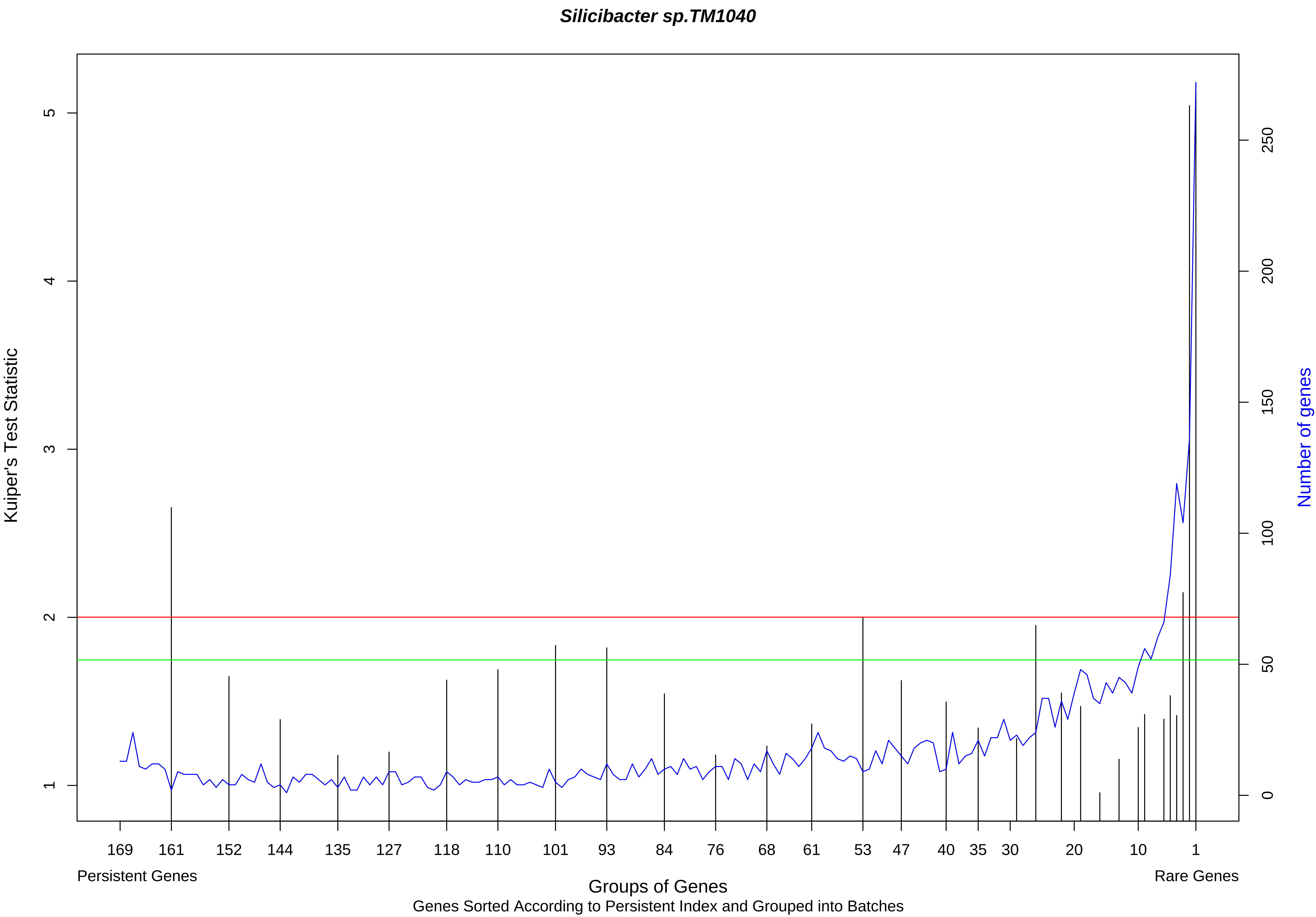

Silicibacter sp.TM1040
Kuiper's Test Statistic
Number of genes
1
2
3
4
5
0
50
100
150
200
250
169
161
152
144
135
127
118
110
101
93
84
76
68
61
53
47
40
35
30
20
10
1
Persistent Genes
Rare Genes
Groups of Genes
Genes Sorted According to Persistent Index and Grouped into Batches

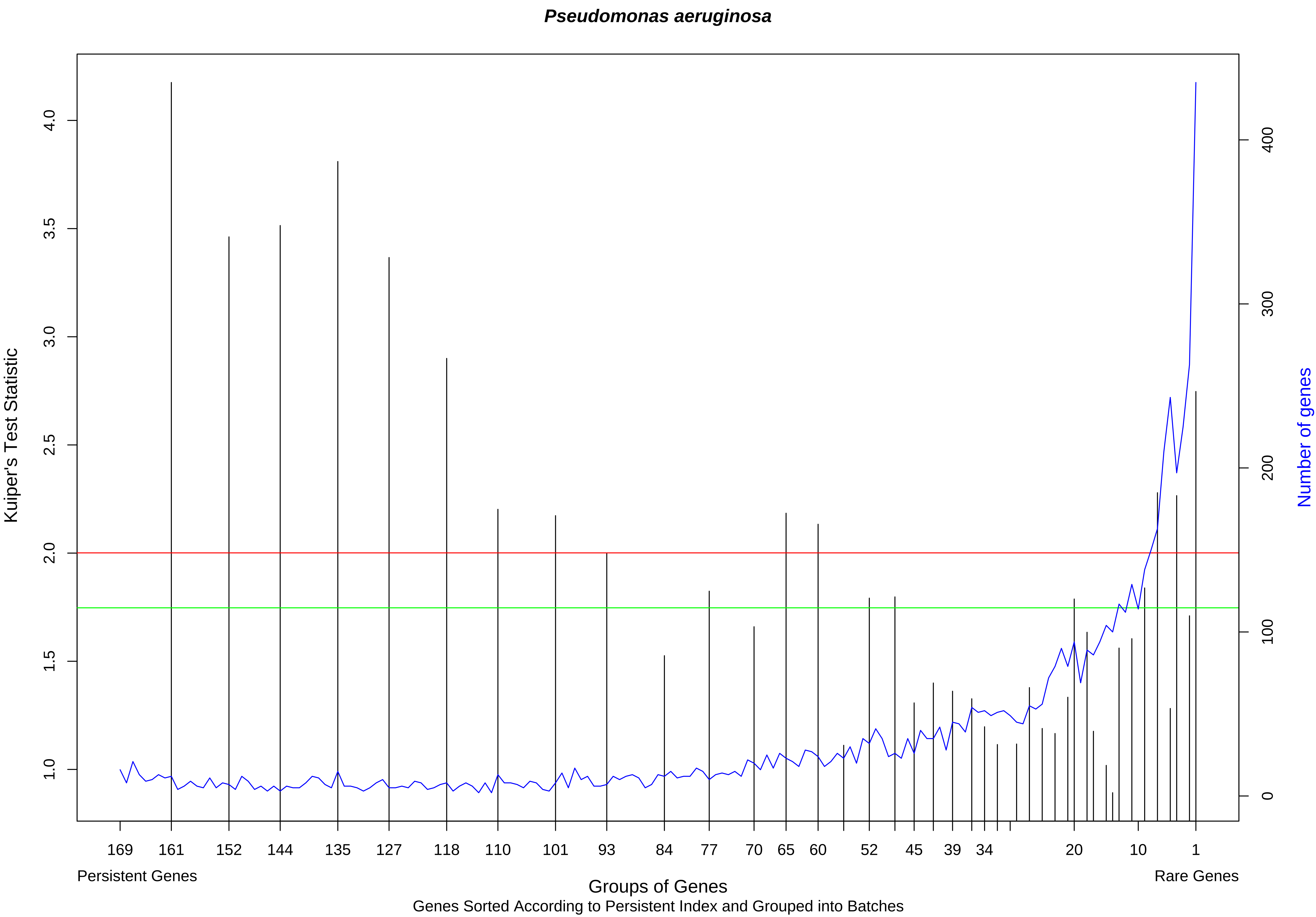

Pseudomonas aeruginosa
Kuiper's Test Statistic
4.0
3.5
3.0
2.5
2.0
1.5
1.0
Number of genes
400
300
200
100
0
169
161
152
144
135
127
118
110
101
93
84
77
70
65
60
52
45
39
34
20
10
1
Persistent Genes
Rare Genes
Groups of Genes
Genes Sorted According to Persistent Index and Grouped into Batches

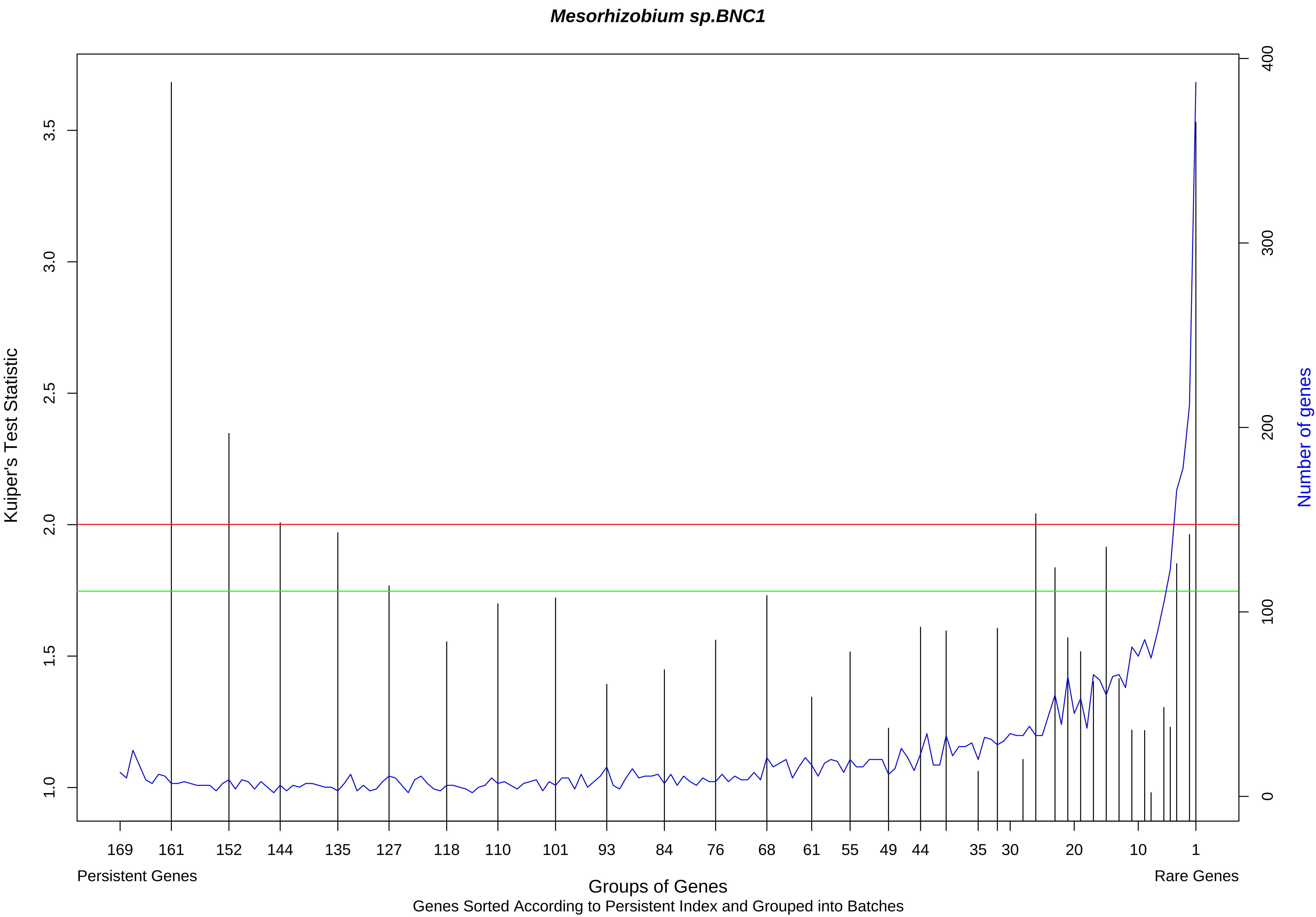

Mesorhizobium sp.BNC1
Kuiper's Test Statistic
1.0
1.5
2.0
2.5
3.0
3.5
Number of genes
0
100
200
300
400
169
161
152
144
135
127
118
110
101
93
84
76
68
61
55
49
44
35
30
20
10
1
Persistent Genes
Rare Genes
Groups of Genes
Genes Sorted According to Persistent Index and Grouped into Batches

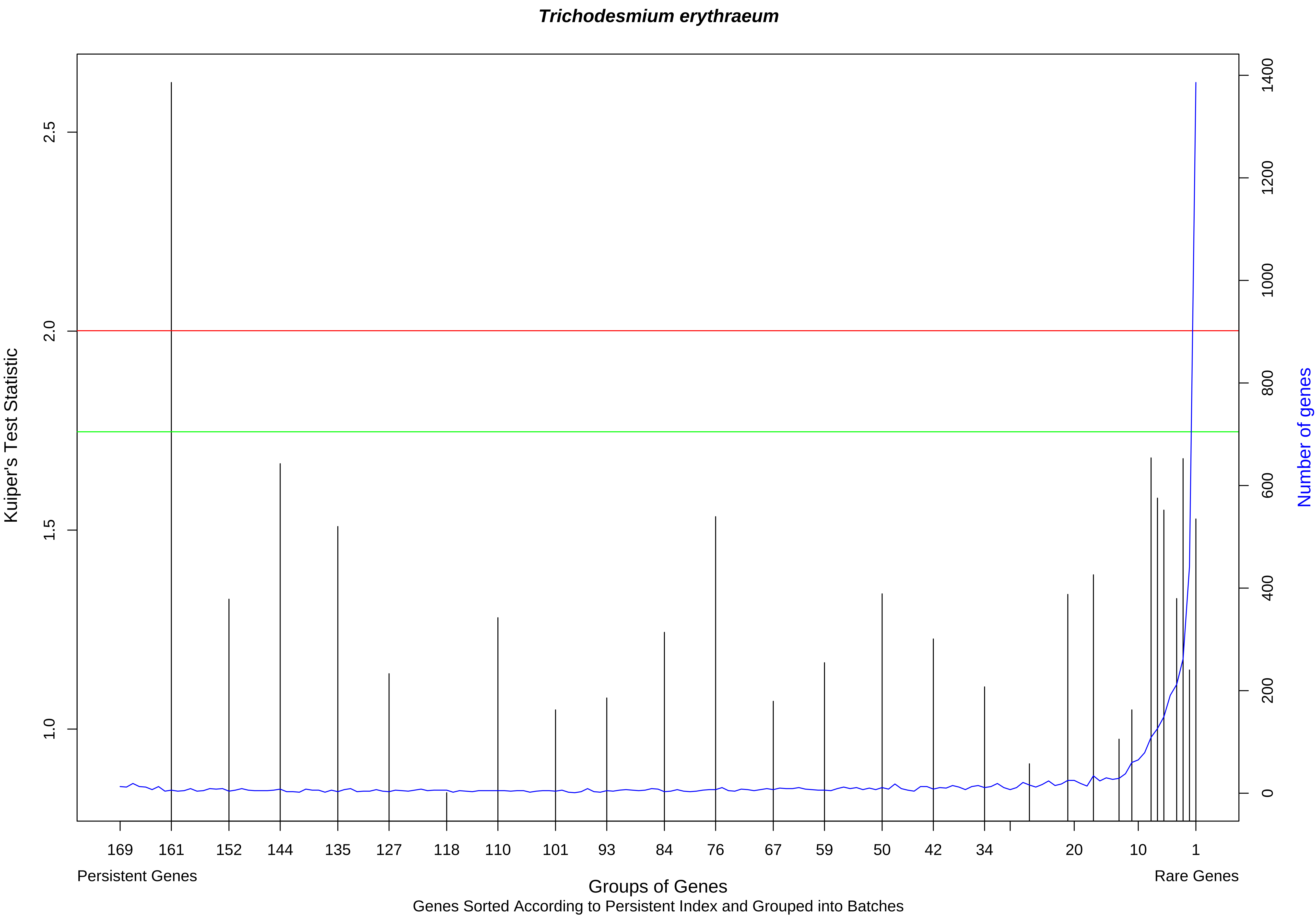
Trichodesmium erythraeum
Kuiper's Test Statistic
Number of genes
2.5
2.0
1.5
1.0
1400
1200
1000
800
600
400
200
0
169 161 152 144 135 127 118 110 101 93 84 76 67 59 50 42 34 20 10 1
Persistent Genes
Rare Genes
Groups of Genes
Genes Sorted According to Persistent Index and Grouped into Batches

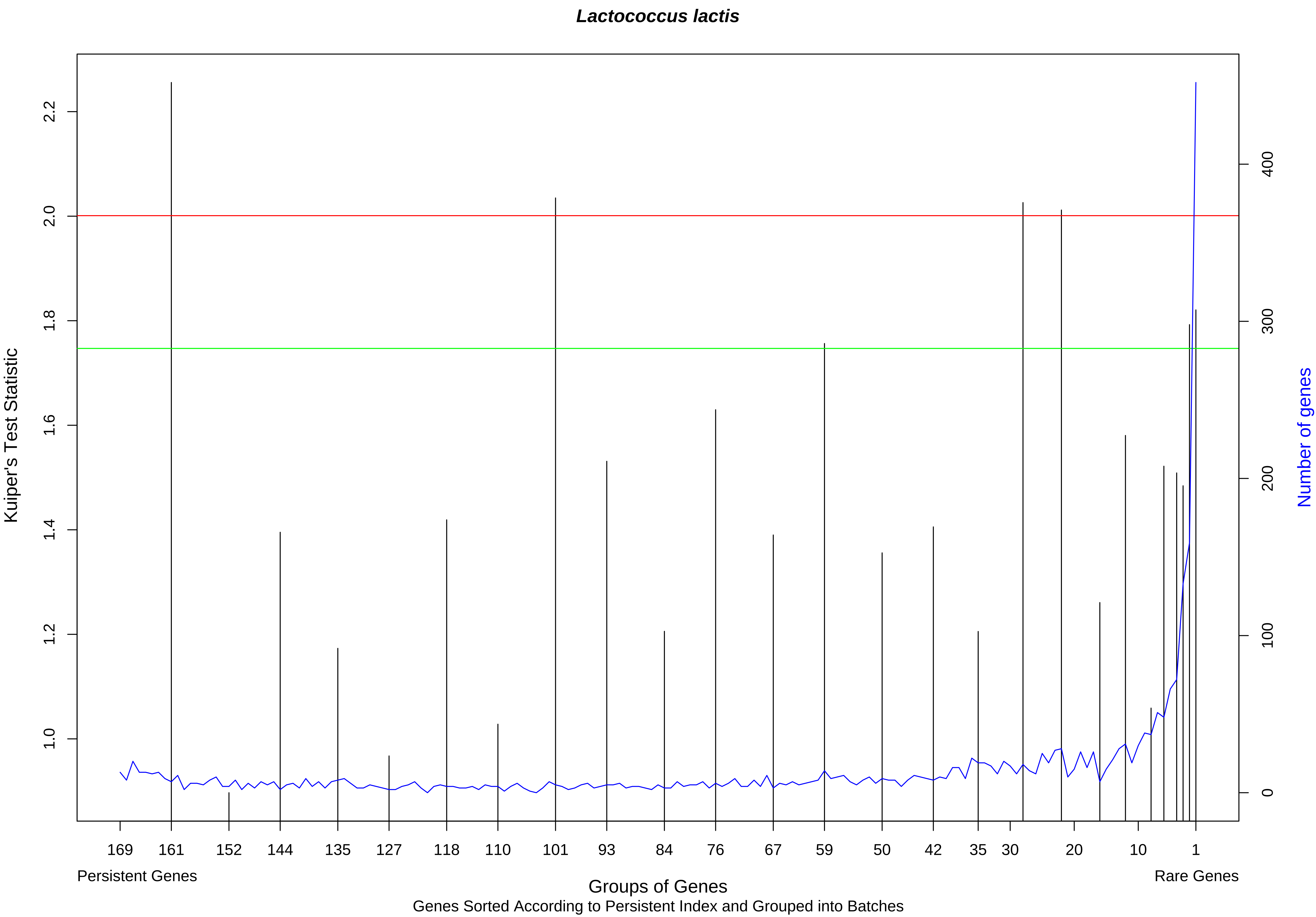

Lactococcus lactis
Kuiper's Test Statistic
1.0
1.2
1.4
1.6
1.8
2.0
2.2
Number of genes
0
100
200
300
400
169
161
152
144
135
127
118
110
101
93
84
76
67
59
50
42
35
30
20
10
1
Persistent Genes
Rare Genes
Groups of Genes
Genes Sorted According to Persistent Index and Grouped into Batches

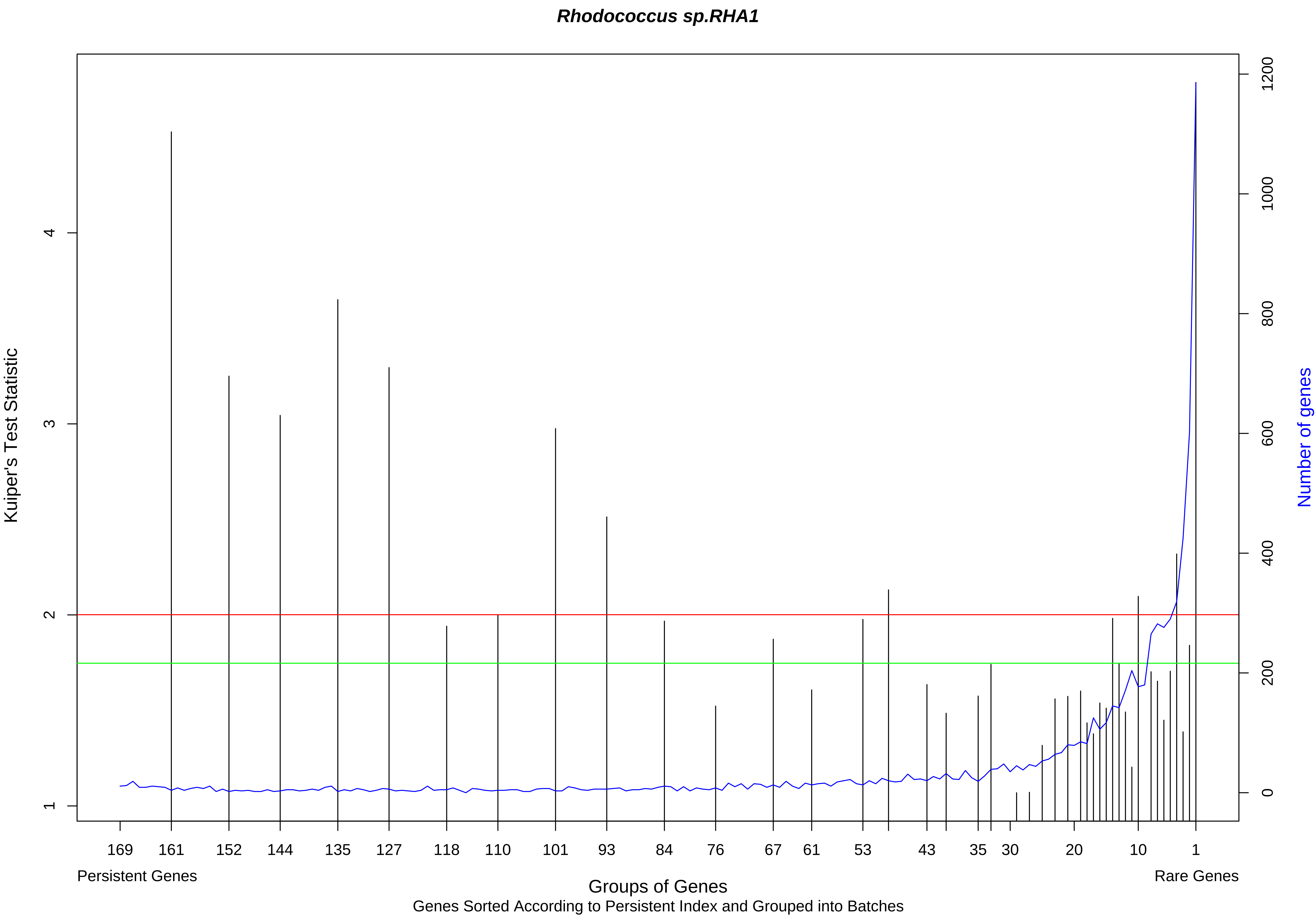

Rhodococcus sp.RHA1
Kuiper's Test Statistic
1
2
3
4
Number of genes
0
200
400
600
800
1000
1200
169
161
152
144
135
127
118
110
101
93
84
76
67
61
53
43
35
30
20
10
1
Persistent Genes
Rare Genes
Groups of Genes
Genes Sorted According to Persistent Index and Grouped into Batches

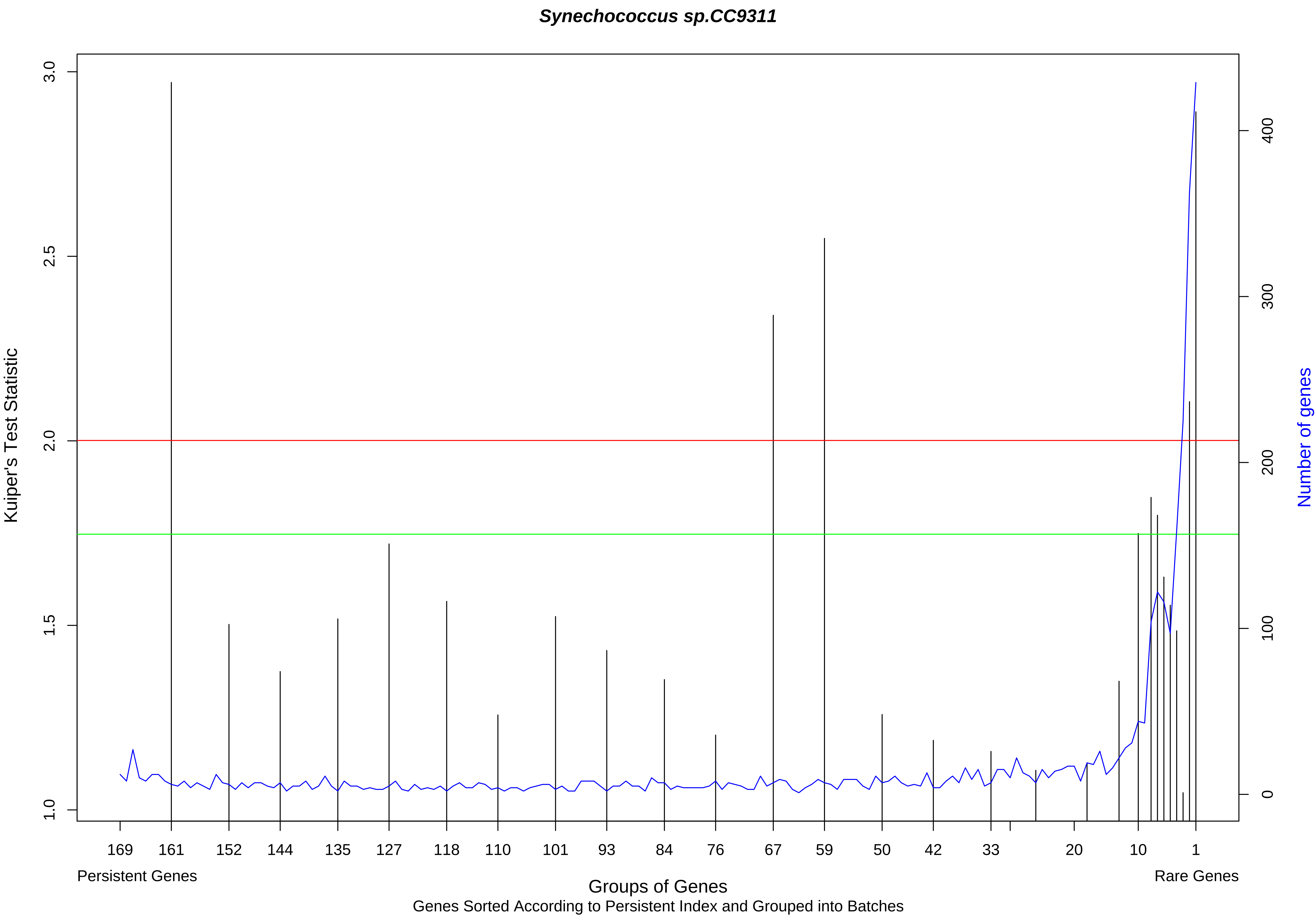
Synechococcus sp.CC9311
Kuiper's Test Statistic
1.0
1.5
2.0
2.5
3.0
Number of genes
0
100
200
300
400
169
161
152
144
135
127
118
110
101
93
84
76
67
59
50
42
33
20
10
1
Persistent Genes
Rare Genes
Groups of Genes
Genes Sorted According to Persistent Index and Grouped into Batches

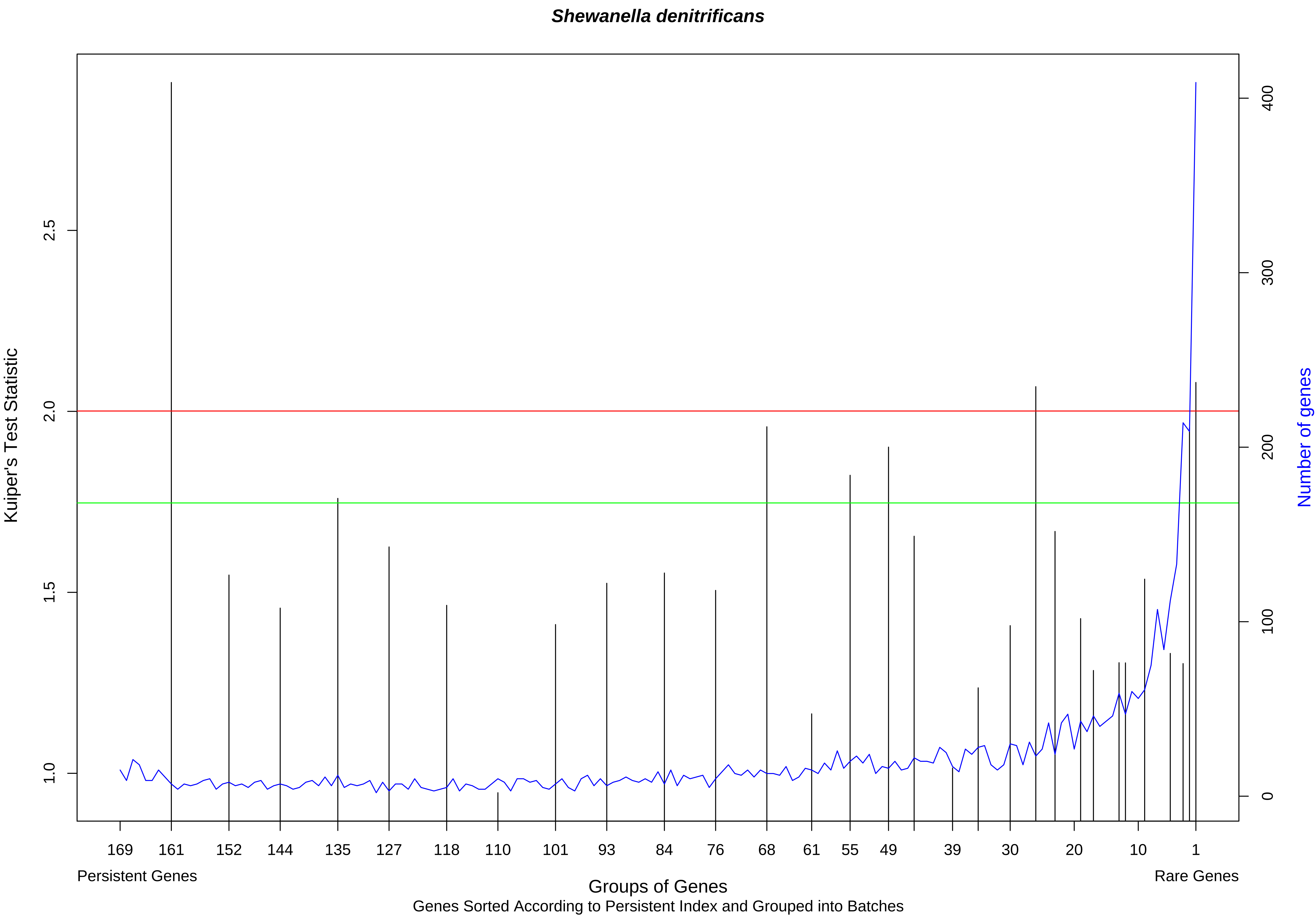

Shewanella denitrificans
Kuiper's Test Statistic
1.0
1.5
2.0
2.5
Number of genes
0
100
200
300
400
169
161
152
144
135
127
118
110
101
93
84
76
68
61
55
49
39
30
20
10
1
Persistent Genes
Rare Genes
Groups of Genes
Genes Sorted According to Persistent Index and Grouped into Batches

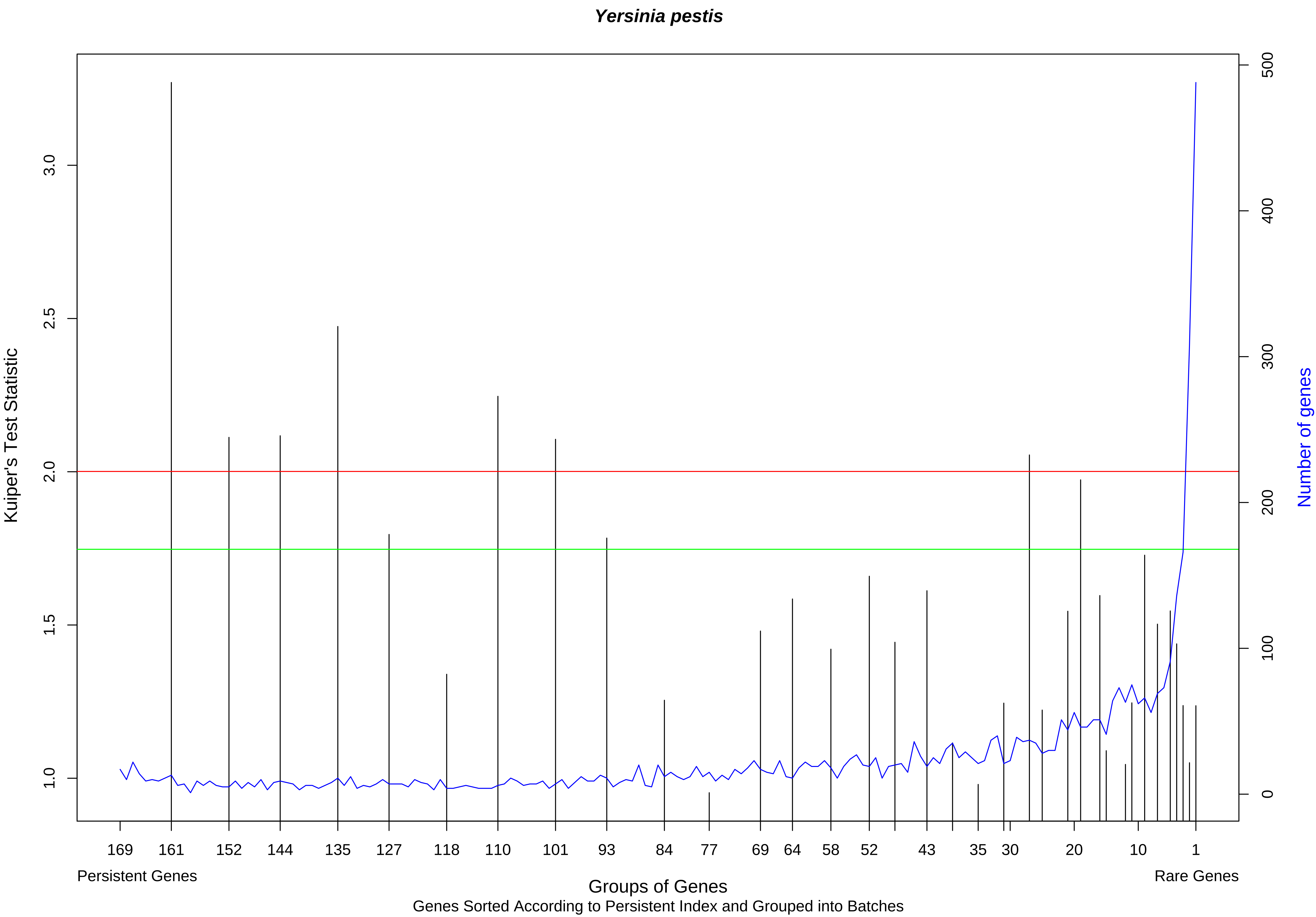

Yersinia pestis
Kuiper's Test Statistic
Number of genes
1.0
1.5
2.0
2.5
3.0
0
100
200
300
400
500
169
161
152
144
135
127
118
110
101
93
84
77
69
64
58
52
43
35
30
20
10
1
Persistent Genes
Rare Genes
Groups of Genes
Genes Sorted According to Persistent Index and Grouped into Batches

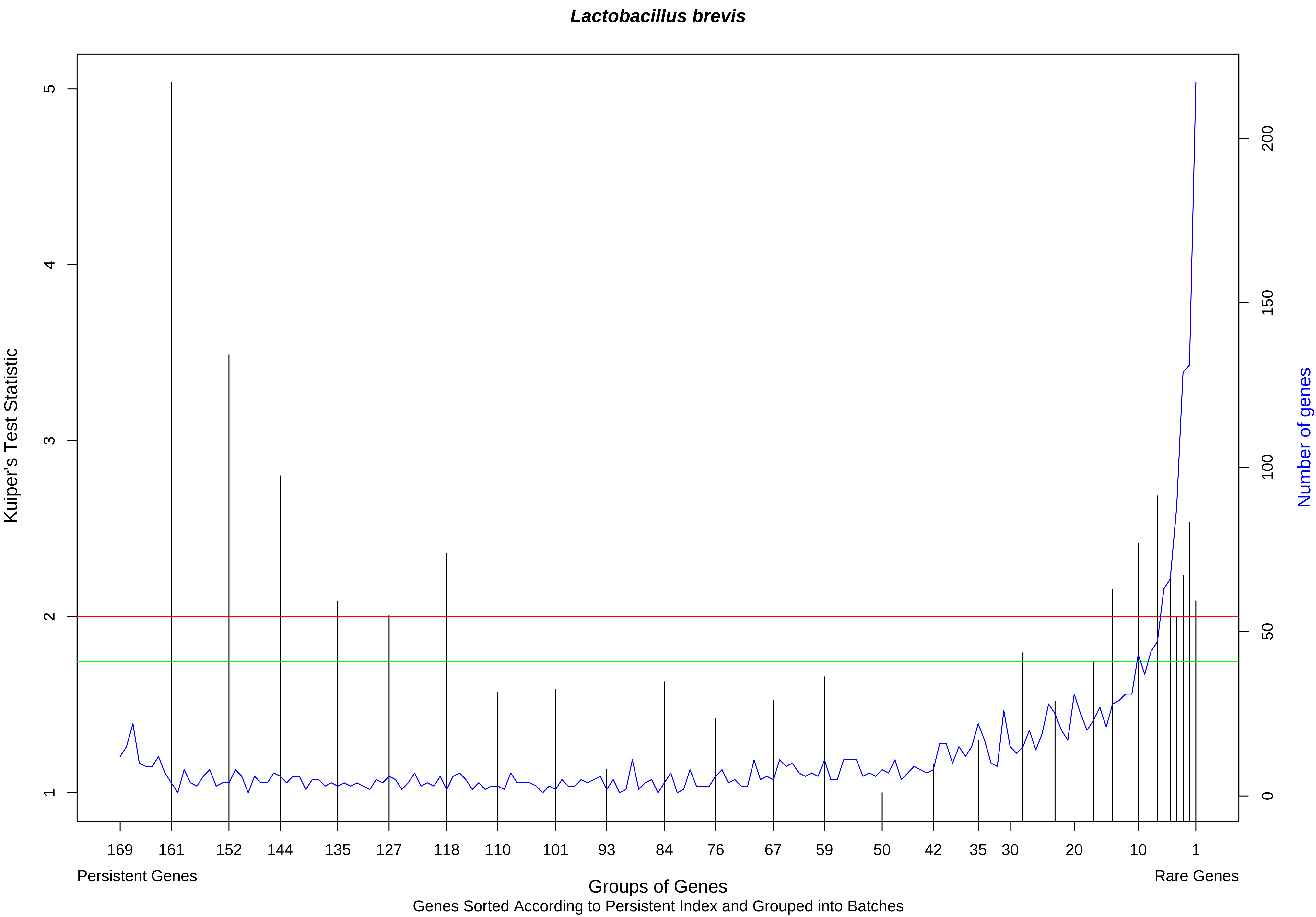

Lactobacillus brevis
Kuiper's Test Statistic
Number of genes
1
2
3
4
5
0
50
100
150
200
169
161
152
144
135
127
118
110
101
93
84
76
67
59
50
42
35
30
20
10
1
Persistent Genes
Rare Genes
Groups of Genes
Genes Sorted According to Persistent Index and Grouped into Batches

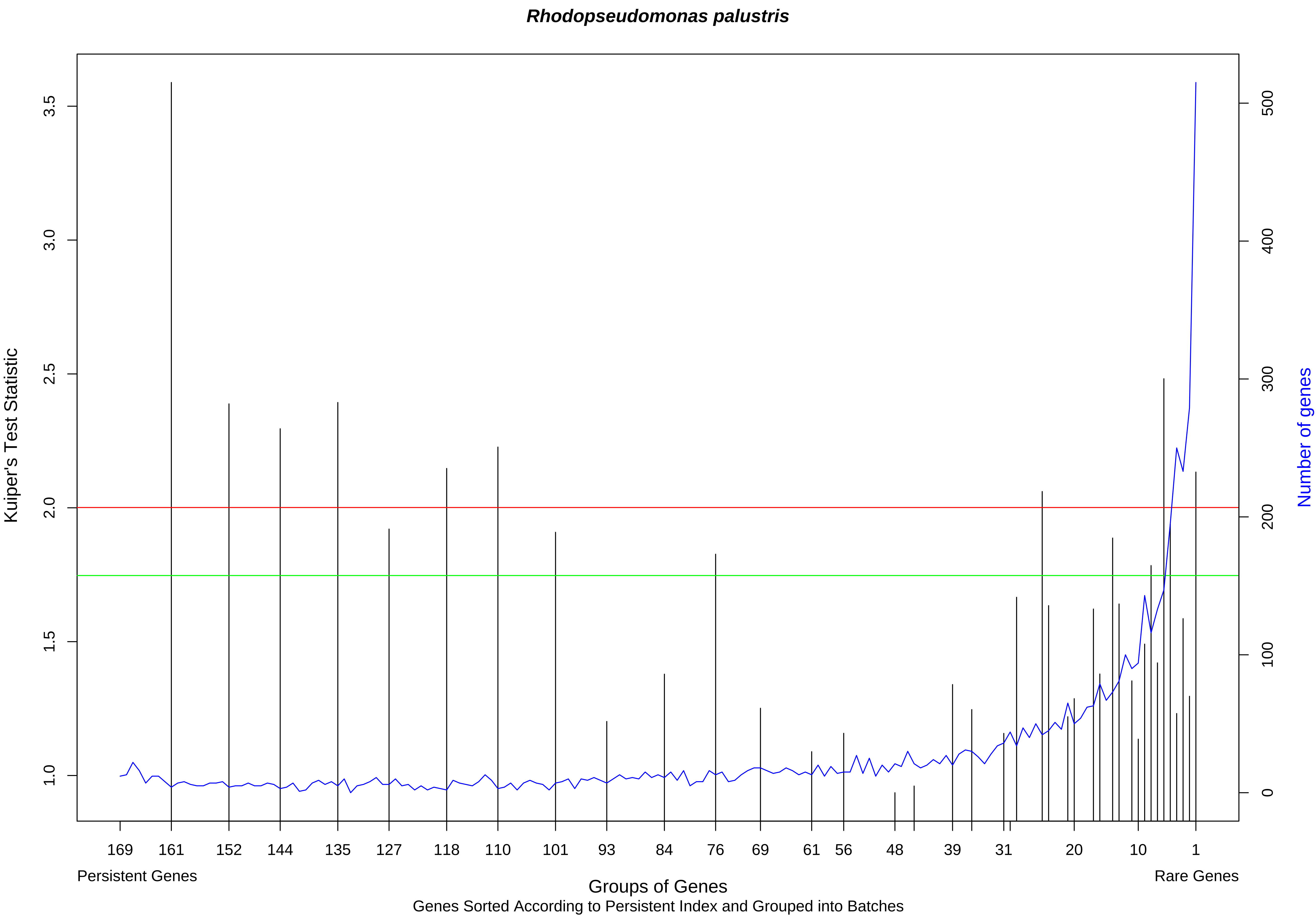

Rhodopseudomonas palustris
Kuiper's Test Statistic
Number of genes
1.0
1.5
2.0
2.5
3.0
3.5
0
100
200
300
400
500
169
161
152
144
135
127
118
110
101
93
84
76
69
61
56
48
39
31
20
10
1
Persistent Genes
Rare Genes
Groups of Genes
Genes Sorted According to Persistent Index and Grouped into Batches

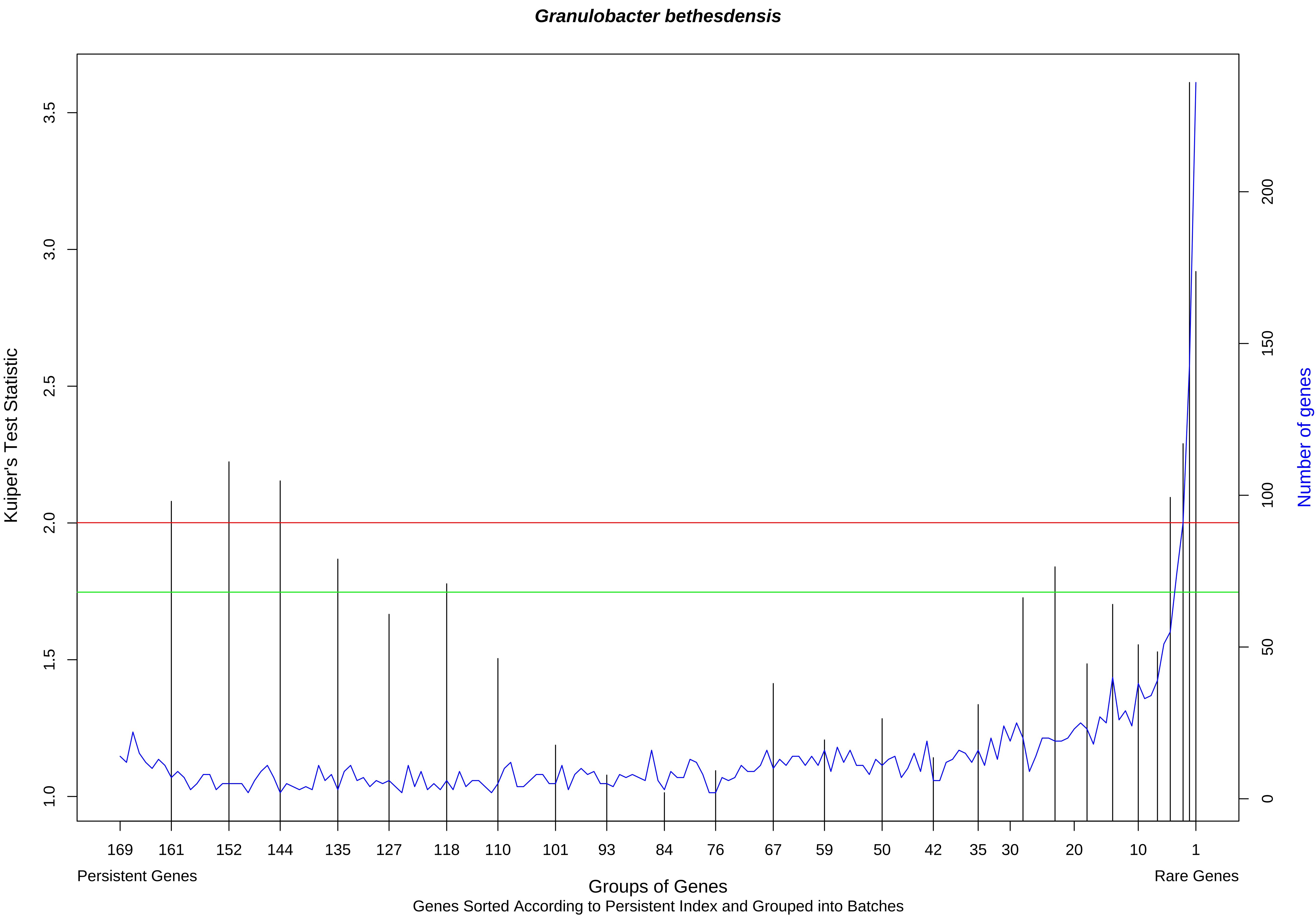
Granulobacter bethesdensis
Kuiper's Test Statistic
1.0
1.5
2.0
2.5
3.0
3.5
Number of genes
0
50
100
150
200
169
161
152
144
135
127
118
110
101
93
84
76
67
59
50
42
35
30
20
10
1
Persistent Genes
Rare Genes
Groups of Genes
Genes Sorted According to Persistent Index and Grouped into Batches

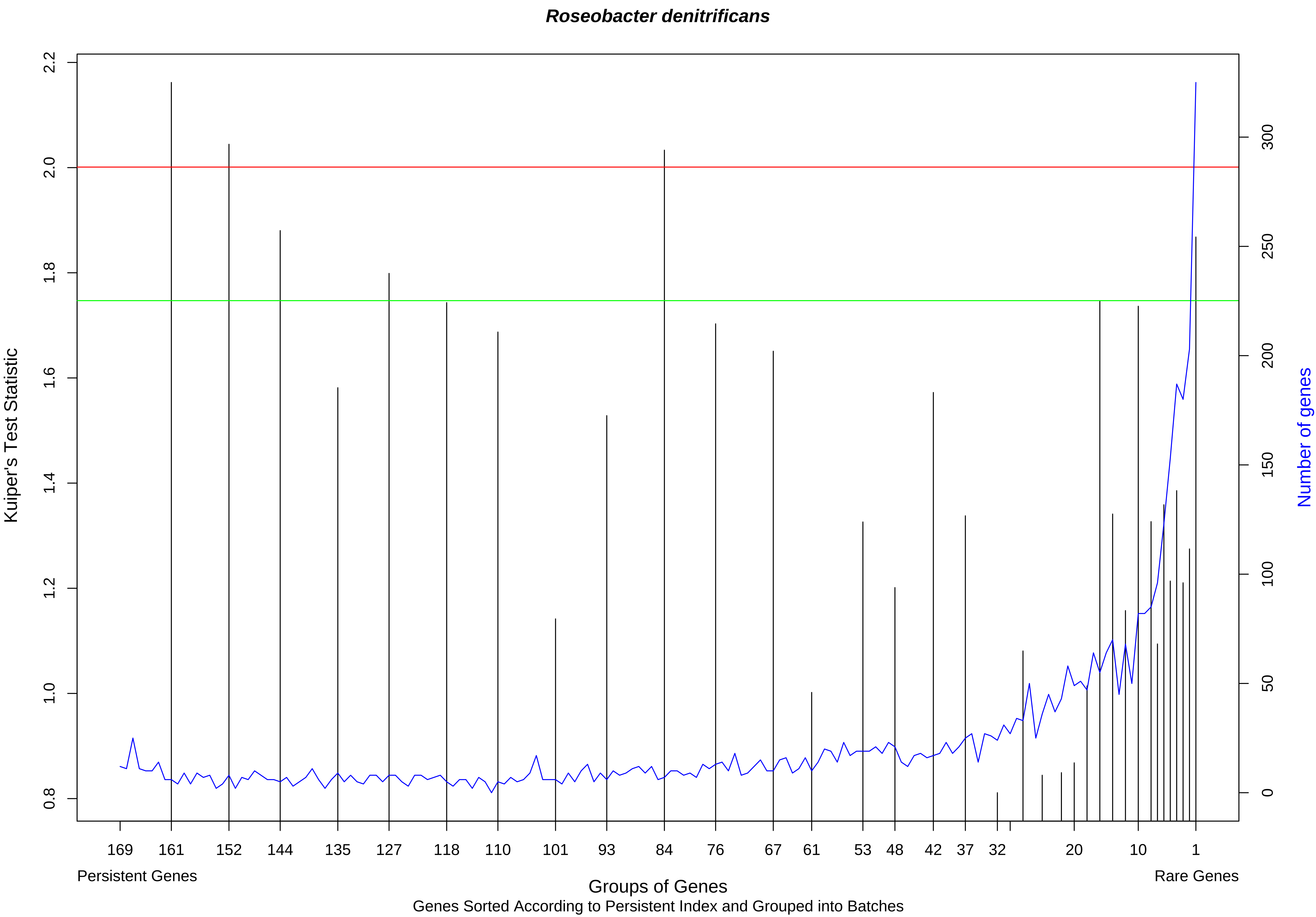

Roseobacter denitrificans
Kuiper's Test Statistic
Number of genes
0.8
1.0
1.2
1.4
1.6
1.8
2.0
2.2
0
50
100
150
200
250
300
169
161
152
144
135
127
118
110
101
93
84
76
67
61
53
48
42
37
32
20
10
1
Persistent Genes
Rare Genes
Groups of Genes
Genes Sorted According to Persistent Index and Grouped into Batches
