# Supplementary figures and images for: Persistence drives gene clustering in bacterial genomes
Source: BMC Genomics. 2008 Jan 7;9:4. doi: 10.1186/1471-2164-9-4 (PMC2234087; doi:10.1186/1471-2164-9-4)

a

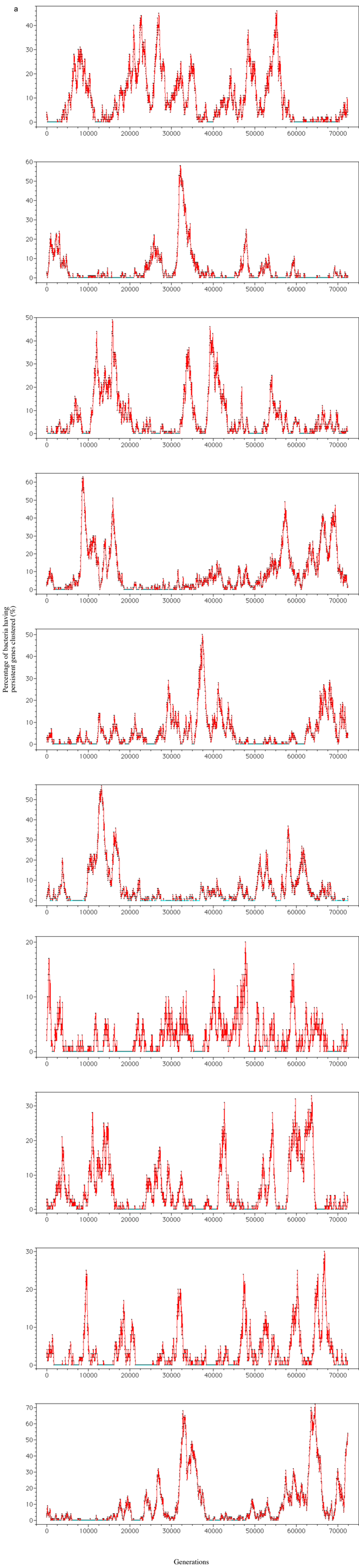

b

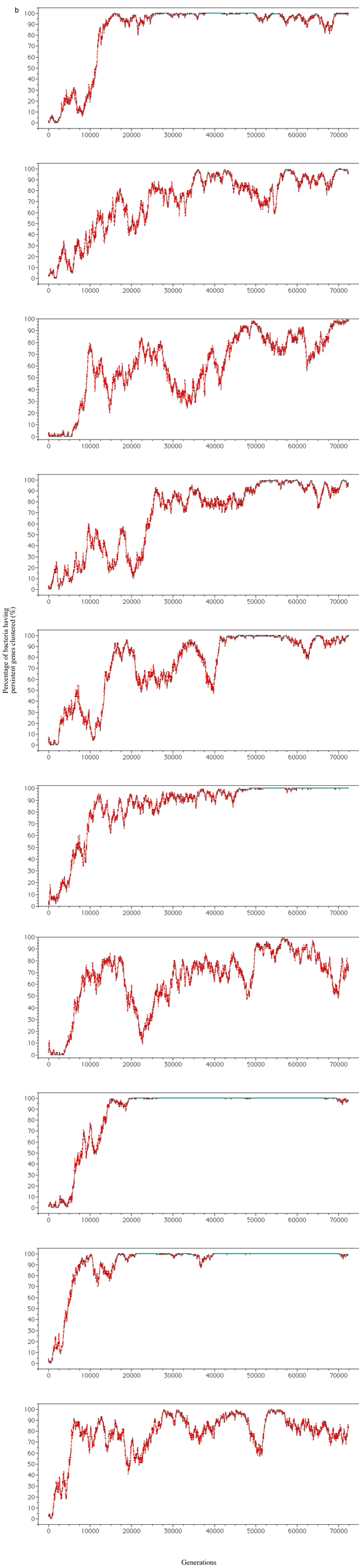

Supplement: Additional file 5 — a: Simulation without considering stabilization forces. b: Simulation with stabilization forces [file 1471-2164-9-4-S5.pdf]

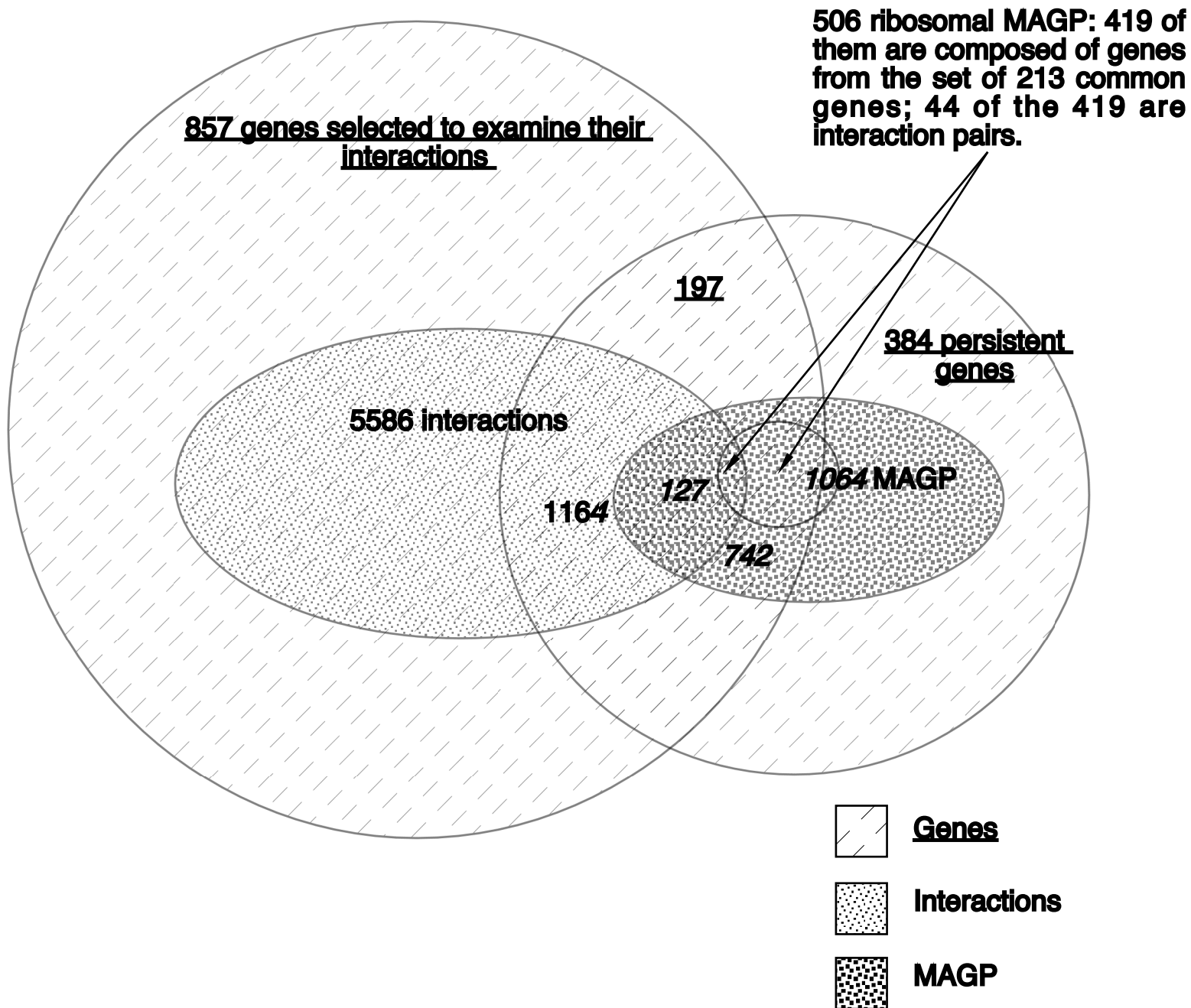

Supplement: Additional file 7 — Venn diagram showing the intersections between the datasets of protein interactions and MAGP [file 1471-2164-9-4-S7.pdf]
